# Aquaponics using a fish farm effluent shifts bacterial communities profile in halophytes rhizosphere and endosphere

**DOI:** 10.1038/s41598-020-66093-8

**Published:** 2020-06-22

**Authors:** Vanessa Oliveira, Patrícia Martins, Bruna Marques, Daniel F. R. Cleary, Ana I. Lillebø, Ricardo Calado

**Affiliations:** 1 CESAM - Centre for Environmental and Marine Studies, Departament of Biology, University of Aveiro, Campus Universitário de Santiago, 3810-193 Aveiro, Portugal; 2 ECOMARE, CESAM - Centre for Environmental and Marine Studies, Departament of Biology, University of Aveiro, Campus Universitário de Santiago, 3810-193 Aveiro, Portugal

**Keywords:** Metagenomics, Plant domestication

## Abstract

The intensification of marine aquaculture raises multiple sustainability issues, namely the handling of nutrient-rich effluents that can adversely impact ecosystems. As integrated multi-trophic aquaculture (IMTA) gains momentum, the use of halophyte plants to phytoremediate aquaculture effluents has received growing attention, particularly in aquaponics. It is, therefore, important to obtain a more in-depth knowledge of the microbial communities present in the root systems of these plants, both in their natural environment (sediment) and in aquaponics, in order to understand their nutrient removal potential. The present study used denaturing gradient gel electrophoresis (DGGE) and barcoded pyrosequencing to assess the bacterial community present in the endosphere and rhizosphere of three halophyte plants: *Halimione portulacoides*, *Salicornia ramosissima* and *Sarcocornia perennis*. Species-specific effects were recorded in the profile and diversity of the bacterial communities present in halophyte roots, with significant differences also recorded for the same halophyte species grown in contrasting environments (sediment *vs*. aquaponics). In aquaponics the most abundant groups belonged to the orders *Rhodocyclales*, *Campylobacterales*, *Rhodobacterales* and *Desulfobacterales*, while in the natural environment (sediment) the most abundant groups belonged to the orders *Rhizobiales*, *Sphingomonadales* and *Alteromonadales*. An overall enrichment in bacterial taxa involved in nutrient cycling was recorded in the roots of halophytes grown in aquaponics (such as *Denitromonas*, *Mesorhizobium*, *Colwellia*, *Dokdonella* and *Arcobacter*), thereby highlighting their potential to reduce the nutrient loads from aquaculture effluents.

## Introduction

The ever-growing demand for seafood has led to an increase in marine aquaculture production and an unprecedented level of intensification^1^. However, marine aquaculture has yet to develop a sustainable paradigm that will minimize environmental risks, interactions and impacts^2^. One potential pathway towards an environmentally sustainable marine aquaculture entails the use of land-based facilities operating recirculating aquaculture systems (RAS)^3,4^.

While RAS technology and conceptual designs continue to improve and minimize the use of water to grow aquatic organisms^5,6^, these systems will continue to generate particulate organic matter (POM) and dissolved organic matter (DOM), as well as dissolved inorganic nutrients, such as nitrogen (DIN = NO_x_-N + NH_4_-N) and phosphorus (DIP = PO_4_-P), that are essential to support life in aquatic ecosystems^7^. The growth of primary producers with nutrient rich RAS effluents may help to develop more balanced productive systems through a diversification of cultured species and by minimizing potential environmental impacts due to effluent discharge (e.g., eutrophication of water bodies and turbidity)^8^. The integration of fish-rearing systems with the hydroponic production of vegetables commonly farmed in agriculture is already well-established in freshwater aquaculture, an approach that has become known as aquaponics^9,10^. This productive framework is aligned with the principles of integrated multi-trophic aquaculture (IMTA) and when operated in marine and/or coastal waters the extractive species most commonly cultured to up-take dissolved inorganic nutrients are seaweeds rather than plants^11,12^.

However, in recent years, a group of macrophytes, namely, salt-tolerant or halophyte plants, has received growing attention in the bioremediation of aquaculture effluents generated by facilities operating in brackish water. The potential use of halophytes as biofilters for marine and brackish water aquaculture has already been highlighted^13–16^, namely within an IMTA framework^17^. Moreover, the ability to successfully bioremediate brackish water aquaculture effluents through the production of halophytes in aquaponics and add value to these crops has been recently demonstrated^18^.

Apart from incorporating inorganic dissolved nutrients, plant roots act as a trap for suspended solids and are important for microbial nutrient transformation processes^19^. Nonetheless, knowledge of the overall diversity and profile of bacterial communities present in the rhizosphere and endosphere of halophytes used for the bioremediation of aquaculture effluents has only recently started to be unraveled, although so far has been limited to the genus *Salicornia*^20^. According to Xiaona *et al*.^20^, in a study acessing the microbial communities in pilot-scale constructed wetlands with *Salicornia* for treatment of marine aquaculture effluents, the most common groups present in the root systems are: *Proteobacteria*, *Bacteriodetes*, *Cyanobacteria* and *Firmicutes*.

In the present study we compared the bacterial communities in the rhizosphere and endosphere of three halophyte species grown in aquaponics and in their natural environment. The halophyte species selected were sea purslane *Halimione portulacoides* (L.) Aellen (also known as *Atriplex portulacoides* L.), perennial marsh samphire *Sarcocornia perennis* (Mill.) A. J. Scott and purple glasswort or marsh samphire *Salicornia ramosissima* J. Woods. The rationale supporting the selection of these three species is their recognized potential to grow in aquaponics and bioremediate the effluents from marine fish farms^17,18^.

The following two null hypotheses were tested: (1) bacterial communities present in the rhizosphere or endosphere do not differ between halophyte species grown in the same environment (sediment or aquaponics); and (2) bacterial communities present in the rhizosphere or endosphere do not differ if the same halophyte species is grown in contrasting environments (sediment *vs*. aquaponics).

## Material and Methods

### Experimental design

A total of 12 tanks (6 m long × 1 m wide × 0.3 m deep) were used to perform phytoremediation trials of the effluent water generated by a super-intensive marine fish farm producing Senegalese sole (*Solea senegalensis*) at Torreira, Aveiro, Portugal (40°47′40.59“N, 8°41′47.69“W). Tanks were assembled in parallel, with each one being supplied effluent water previously filtered by a polychaete assisted sand filter (1 m long × 1 m wide × 0.7 m deep) stocked with ragworms (*Hediste diversicolor*) to remove excess organic particulate matter from the water. All sets of polychaete assisted sand filters and phytoremediation tanks were gravity fed by two interconnected header tanks (2 m long × 1 m wide × 0.7 m deep). The header tanks were stocked with bio-blocs where effluent water pumped from the fish farm settling basin was trickled and strongly aerated before being distributed to the 18 sets of polychaete assisted sand filters and phytoremediation tanks. Water inflow to each set was controlled manually through the use of ball-valves and set at 180 L h^−1^ of effluent water (thus allowing 2 full water renewals per day for each phytoremediation tank). A schematic representation of the culture system and further technical details can be found in Marques *et al*.^18^. The physico-chemical parameters of effluent water were as follows: salinity: 20.0 ± 1.0, water temperature: 19.7 ± 1.8 °C, pH: 7.9 ± 0.2, dissolved oxygen: 8.8 ± 0.7 mg L^−1^, dissolved inorganic nitrogen (DIN = NO_x_-N + NH_4_-N): 8.9 ± 1.3 mg L^−1^ and dissolved inorganic phosphorus (DIP): 0.32 ± 0.11 mg L^−1^. Each phytoremediation tank was stocked with 12 floating Styrofoam seed trays (0.9 m long and 0.6 m wide) with 100 pyramid-shaped cells, thus operating as a raft hydroponic tank. Every other cell of the tray was stocked with a halophyte plant transplanted from the wild, for a total of 800 halophytes per tank grown on aquaponics (for further details on plants and stocking conditions please see^18^).

### Halophytes sampling

For this study, due to tank dimensions and inter-variability regarding nutrient dynamics^18^, one single tank stocked with each of the halophyte species referred above was selected and composite samples covering the 6 m long × 1 m wide tank were collected. Halophytes were sampled from different floating Styrofoam trays within the selected *H. portulacoides, S. perennis* and *S. ramosissima* tank. After 5 months of culture in aquaponics, four composite samples per halophyte species were collected (4 composite samples × 3 halophyte species in aquaponics = 12 composite samples). Each composite sample was assembled by pooling 4 plants per tray within the selected phytoremediation tank. Additionally, 4 composite samples per halophyte species were also collected at the same location where halophytes were initially sampled to stock the phytoremediation tanks (4 composite samples × 3 halophyte species from their natural habitat = 12 composite samples). Each composite sample (for a total of 24 composite samples) was stored separately in sterile plastic bags and processed on the same day of sampling.

### Rhizosphere and endosphere sampling

Roots were manually shaken to remove loosely bound sediment, which was discarded. The endophytic community was recovered from roots. Root material was vigorously washed in distilled water (5 min) and the root surface was disinfected by sequential washing with 95% ethanol and 1% sodium hypochlorite supplemented with one droplet of Tween 80^®^ (Sigma-Aldrich) and rinsed three times using sterile distilled water.

### DNA extraction

Total community DNA (TC-DNA) was extracted from rhizosphere (0.25 g) and endosphere (0.25 g) samples using the PowerSoil^®^ DNA Isolation kit (Cambio) following manufacturer’s instructions.

### 16S rRNA gene denaturing gradient gel electrophoresis (DGGE) profiling of bacterial communities

A nested PCR approach was used to amplify 16S rRNA gene sequences from rhizosphere and endosphere samples^21^, as this approach is more efficient for amplification of 16S rRNA gene fragments suitable for DGGE analyses. Briefly, in the first PCR the universal bacterial primers 27 F (5′- AGAGTTTGATCMTGGCTCAG-3′) and 1494R^22^ (5′-CTACGGRTACCTTGTTACGAC-3′) were used. Reaction mixtures (25 µL) contained 12.5 μl DreamTaq™ PCR Master Mix (Fisher Scientific), 0.1 µM of each primer, 0.08 mg mL^−1^ bovine serum albumin (BSA) and 1 µL of template DNA. The amplification conditions were as follows: initial denaturation (94 °C for 5 min); 20 cycles of denaturation (94 °C for 45S), annealing (56 °C for 45S), and extension (72 °C for 1.5 min), and a final extension (72 °C for 10 min). The amplicons obtained from the first PCR were used as a template for a second PCR with bacterial DGGE primers 984F-GC (5′-CGCCCGGGGCGCGCCCCGGGCGGGGCGGGGGCACGGGGGGAACGCGAAGAACCTTAC-3′) and 1401 R (5′-CGGTGTGTACAAGGCCCGGGAACG-3′). The PCR reaction mixtures (25 µL) consisted of 12.5 μl DreamTaq^®^ PCR Master Mix (Fisher Scientific), 0.1 µM of each primer, 1% (v/v) dimethyl sulfoxide (DMSO) and 1 µL of template DNA. After 4 min of denaturation at 94 °C, 25 thermal cycles of 1 min at 95 °C, 1 min at 53 °C, and 1.5 min at 72 °C, the PCR was finished by an extension step at 72 °C for 7 min. Five µL of PCR products were analyzed by electrophoresis on a 1% agarose gel and stained with GelRed^®^ (Biotium). The DGGE of amplified 16S rRNA gene fragments was performed using DCode System (Universal Mutation Detection System, Bio-Rad). PCR products containing approximately equal amounts of DNA (estimated based on band intensity detected in the agarose gel electrophoresis) were loaded onto 6–10% (w/v) polyacrylamide gel in 1 × TAE buffer (0.04 M Tris-Acetate, 0.001 M EDTA; pH 8.0). The 6–10% polyacrylamide gel was made with a denaturing gradient ranging from 40 to 58%. A DGGE marker was used for internal normalization and as an indication of the quality of the analysis. Electrophoresis was performed for 16 h at 80 V at 60 °C in 1 × TAE buffer. Following electrophoresis, gels were silver-stained according to Heuer *et al*.^23^. Scanned DGGE gels (Figs. S1 and S2) were processed using the Bionumerics software 6.6 (Applied Maths, Sint-Martens-Latem, Belgium). The matrix constructed by the program incorporated both band position and intensity of each band that was processed in a spreadsheet. The intensity of each DGGE band was then normalized by total sample intensity to obtain relative abundances.

### Barcoded pyrosequencing

A barcoded pyrosequencing approach was used for compositional analysis of bacterial communities. Before pyrosequencing, TC-DNA of all four replicates per experimental treatment was combined, forming one DNA library for each. The V3-V4 region was amplified using barcoded fusion primer V3 Forward (5′ -ACTCCTACGGGAGGCAG-3′) and V4 Reverse (5′ -TACNVRRGTHTCTAATYC-3′) with Roche 454 titanium sequencing adapters (see Oliveira *et al*.^24^, for a detailed description). Sequences generated in this study can be downloaded from the NCBI SRA: SRP155695. A detailed description of barcoded pyrosequencing analysis using QIIME and UPARSE (http://qiime.org/; https://www.drive5.com/uparse/) can be found in Cleary *et al*.^25,26^ The taxonomic affiliation of all bacterial OTUs was determined using Ribosomal Database Project (RDP) classifier.

### Data analysis

Two square matrices were imported into R (version 3.1.2; http//www.r-project.org/) using the read.table() function: (1) containing band ‘abundance’ based on band intensity and position on the DGGE gel and (2) containing the presence and raw abundance of all operational taxonomic units (OTUs) per sample generated in the pyrosequencing analysis. The distribution of OTUs in samples was assessed using a Venn diagram with the venn() function in the gplots package^27^ in R. In the OTU abundance matrix, sequences not classified as bacteria or classified as chloroplasts or mitochondria were removed prior to statistical analysis. Both matrices were log_e_(*x* + 1) transformed and a distance matrix was constructed using the Bray-Curtis index with the vegdist() function, in the VEGAN package^28^ in R. Variation in composition was visualized with principal coordinates analysis (PCO) using the cmdscale() function in R with the Bray–Curtis distance matrix as input. Selected sequences from dominant OTUs (≥50) and their closest relatives were downloaded using the NCBI Basic Local Alignment Search Tool (BLAST) command line ‘blastn’ tool with the _db argument set to nt^29^. A heatmap was constructed to visualize the distribution of the dominant OTUs (≥50 sequence reads). The heatmap was generated using the heatmap2() function in the R package gplots. A two-way permutational multivariate analysis of variance (PERMANOVA) was performed to test for the existence of statistically significant differences between DGGE profiles performed for the rhizosphere and the endosphere of the halophytes grown in the different environments using PRIMER v6 with the PERMANOVA + add-on software. Halophyte species (*H. portulacoides* vs. *Sar. perennis* vs. *Sal. ramosissima*) and environment (sediment vs. aquaponics) were used as categorical factors.

## Results and Discussion

### Bacterial communities profiles

**Figure 1:**
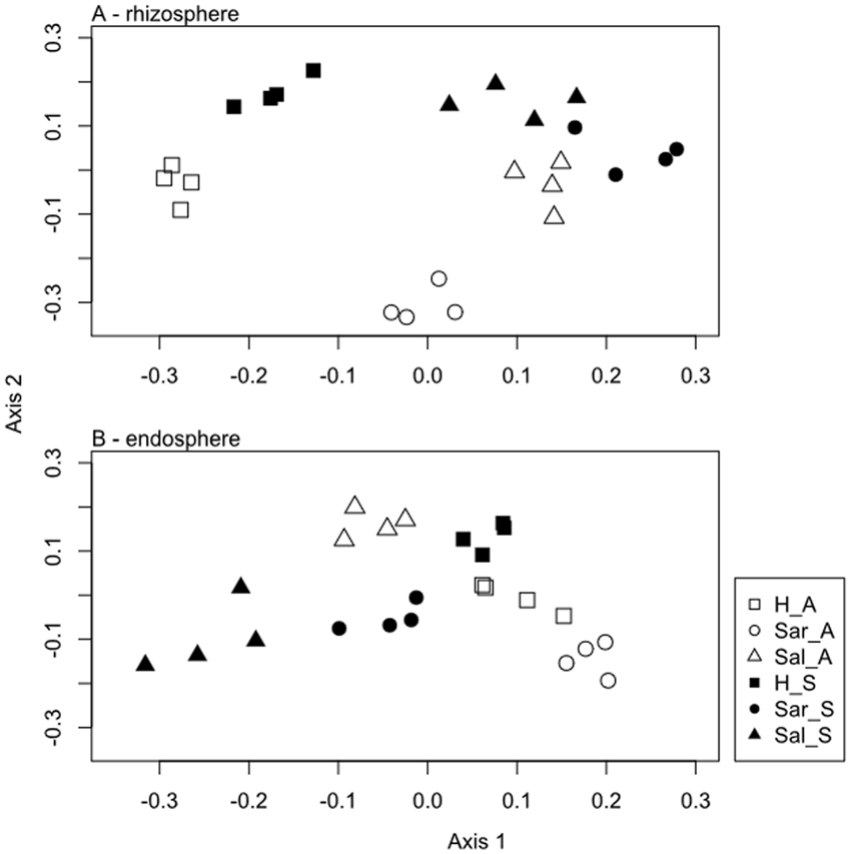
Ordination diagrams (PCO) of DGGE profiles of the bacterial communities present on the: (**A**) rhizosphere of plants grown in aquaponics (A) or sediment (S); (**B**) endosphere of plants grown in aquaponics (A) or sediment (S). H: *Halimione portulacoides*, Sal: *Salicornia ramosissima* and Sar: *Sarcocornia perennis*.

PCO ordination analysis of bacterial DGGE profiles showed distinct bacterial communities in halophyte growth environments (sediment *vs*. aquaponics) (Fig. 1). The first two PCO axes explained 48.9% and 40.0% of the compositional variation in rhizosphere data (PCO axis 1: 26.8%, PCO axis 2: 22.0%) and endosphere data (PCO axis 1: 23.4%, PCO axis 2: 16.7%), respectively. PERMANOVA analysis of bacterial DGGE profiles revealed a significant interaction (*P* = 0.0001) between plant species and the type of growth environment (sediment *vs*. aquaponics) (Table S1 and S2). Moreover plant species specific effects were observed on the profile of root bacterial communities (*P* = 0.0001). These results were somehow expected, as oxygen and nutrient availability, along with other factors, play a relevant role in wetland performance^13^ and consequently may affect the composition of bacterial communities^30^. *Salicornia ramosissima* grown in its natural environment (sediment) displayed the most distinct microbial community in the endosphere and rhizosphere. *Salicornia ramosissima* plants were collected close to the area where effluent from the super-intensive marine fish farm is discharged into the environment, whereas samples of the other two species were collected in Ria de Aveiro channels solely subject to tidal nutrient inputs. This may explain the distinct composition differences among plant species. Our findings are in line with those described by several authors^30–33^ that revealed that composition and temporal variation of root-inhabiting bacterial communities were associated with habitat characteristics and vegetation type, such as the influence of root exudates.

### OTU compositional analysis

Barcoded pyrosequencing analysis yielded 12,924 sequences clustered into 1859 bacterial OTUs after quality control, OTU picking and removal of chimeras and sequences not assigned to the domain Bacteria (Table S3).

**Figure 2:**
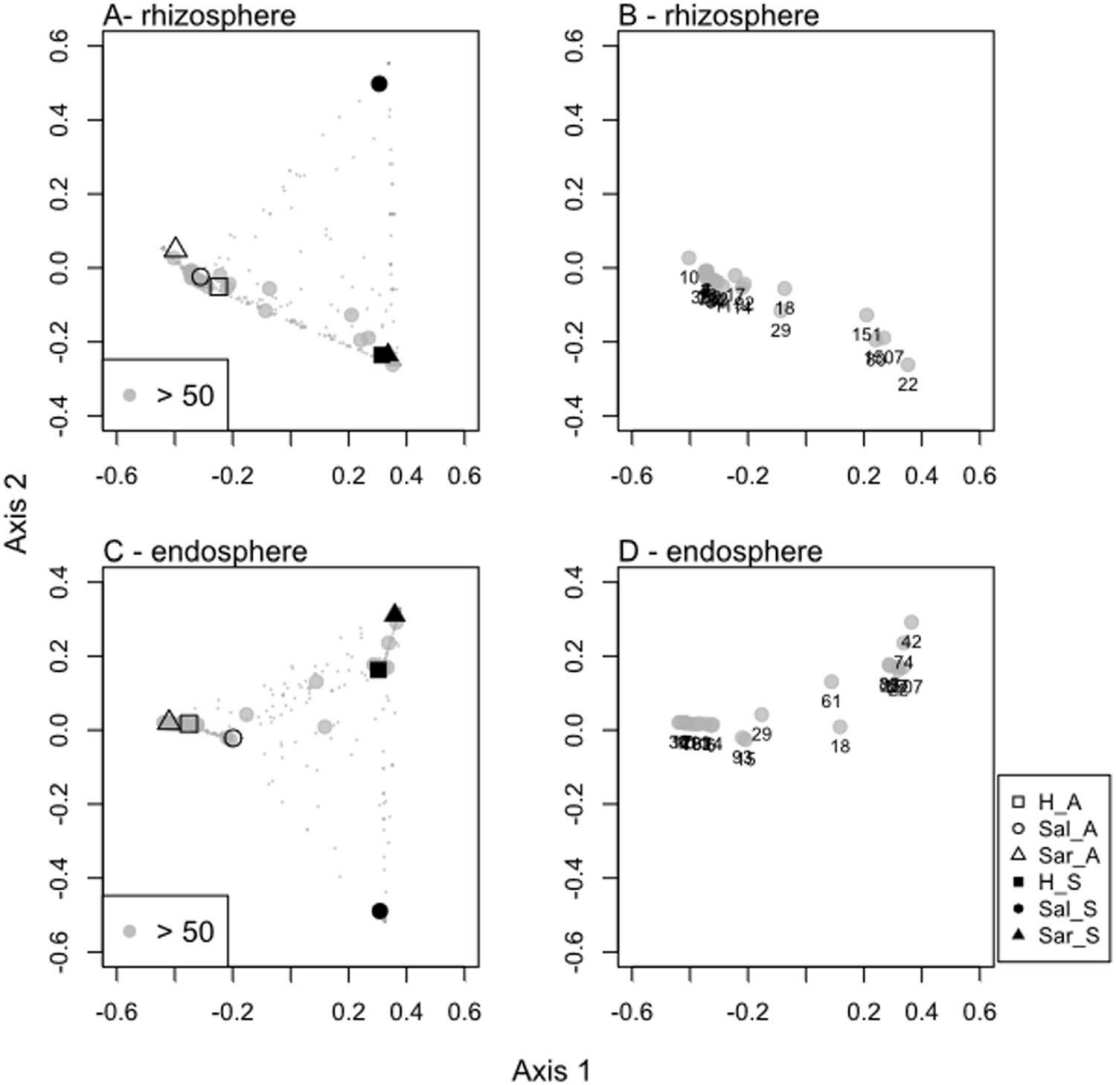
Ordination showing the first two axes of PCO analysis of the bacterial community present: (**A**) in the rhizosphere of plants grown in aquaponics (A) or sediment (S); (**B**) numbers represent dominant OTUs associated to the rhizosphere; (**C**) in the endosphere of plants grown in aquaponics (A) or sediment (S); (**D**) numbers represent dominant OTUs associated to the endosphere. H: *Halimione portulacoides*, Sal: *Salicornia ramosissima* and Sar: *Sarcocornia perennis*.

**Figure 3:**
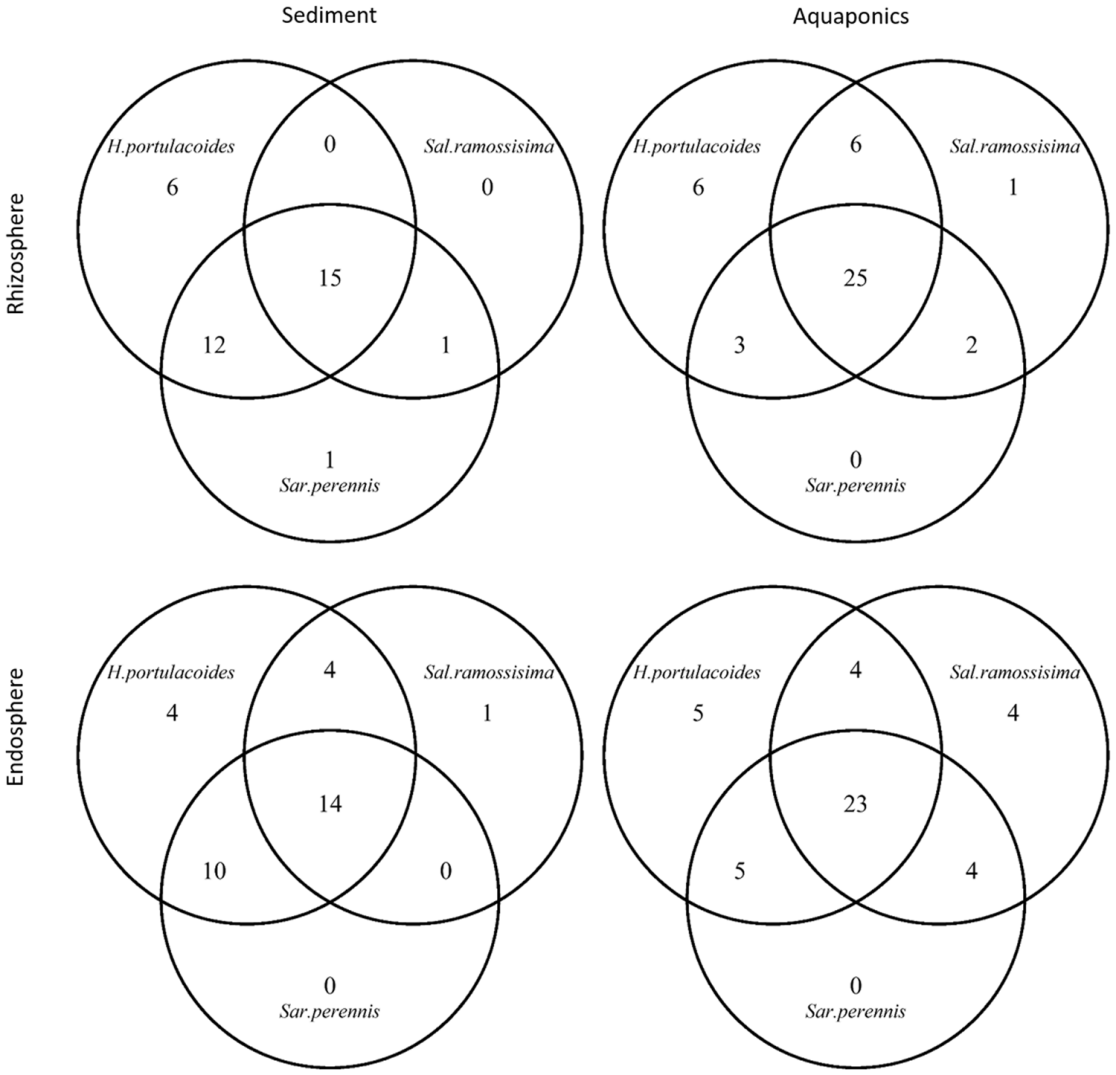
Venn diagrams showing the amount of most abundant bacterial OTUs (≥50 sequences) shared between halophyte plant species grown in aquaponics or sediment.

**Figure 4:**
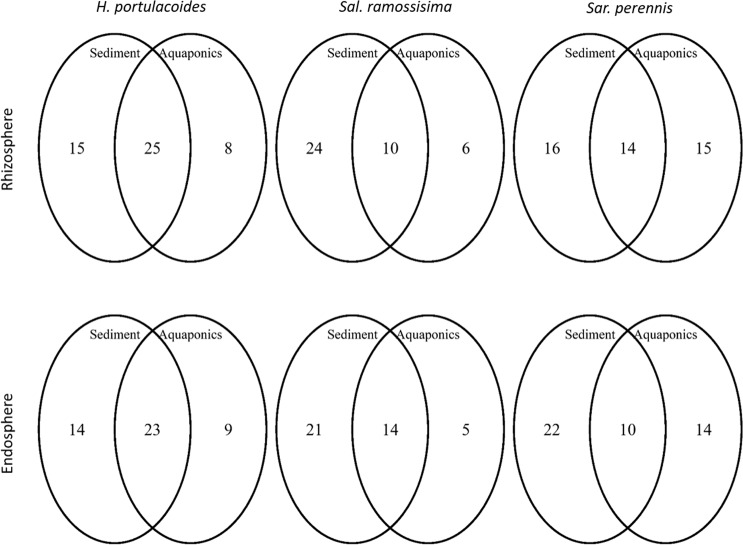
Venn diagrams showing the number of most abundant bacterial OTUs (≥50 sequences) shared by each halophyte plant species grown in aquaponics or sediment.

In line with DGGE profiles, the PCO ordination revealed compositional differences among rhizosphere and endosphere bacterial communities recorded in aquaponics and sediment (Fig. 2). The first PCO axis separated aquaponics samples from sediment samples. There were three dominant bacterial OTUs (OTUs 18, 29 and 61), which were almost equally distributed in both growth environments (Fig. 2B and D) and were classified as belonging to the order *Rhizobiales* (Table S3). The distribution of most abundant OTUs (≥50 sequences) among halophyte species in the same growth environment showed that the largest component consisted of abundant OTUs that were found in all three plants (Fig. 3). The halophyte *H. portulacoides* was the plant where the highest number of exclusive OTUs was recorded, while in the *S. perennis* rhizosphere sediment there was only one restricted OTU (OTU 27, Fig. 3). The most abundant OTUs (≥50 sequences) were only recorded in a certain growth environment (Fig. 4), with exception of *H. portulacoides* rhizosphere and endosphere where the largest component consisted of abundant OTUs that were shared between sediment and aquaponics. The highest number of restricted OTUs was recorded in the rhizosphere and endosphere of plants grown in sediment (Table S4 to S9). OTUs were assigned to 33 phyla, 82 classes and 117 orders and only 13 OTUs remained unclassified at the phylum level (Table S3). The two most abundant phyla were *Bacteroidetes* and *Proteobacteria*. The majority of bacterial OTUs in all samples were assigned to *Proteobacteria* and all five major classes were detected, representing on average more than 82% of all sequences. The phylum *Proteobacteria* consists of a diverse group of bacteria recovered from different hosts and environments, and is an important player in nutrient cycling^34^. *Bacteroidetes* was the second most abundant phyla, with relative abundance ranging from 0.7% to 25%. Members of the *Bacteroidetes* were more abundant in sediment and are known degraders of plant polysaccharides^35^.

### Phylotype-level 16S rRNA gene analysis

**Figure 5:**
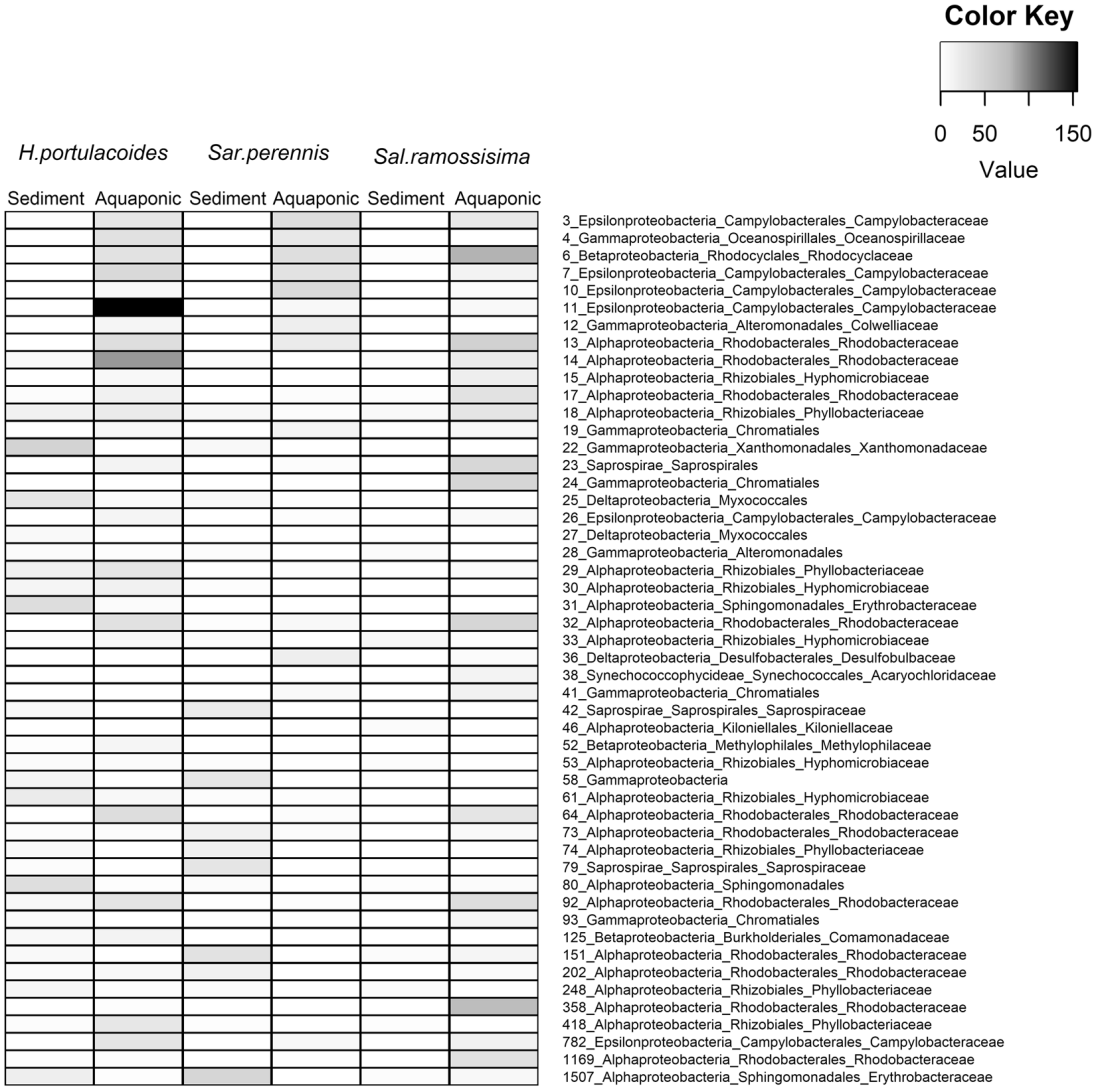
Heatmap showing the abundance of dominant 16S rRNA sequence reads (≥50 sequences) in the rhizosphere of each halophyte plant species grown in aquaponics or sediment.

**Figure 6:**
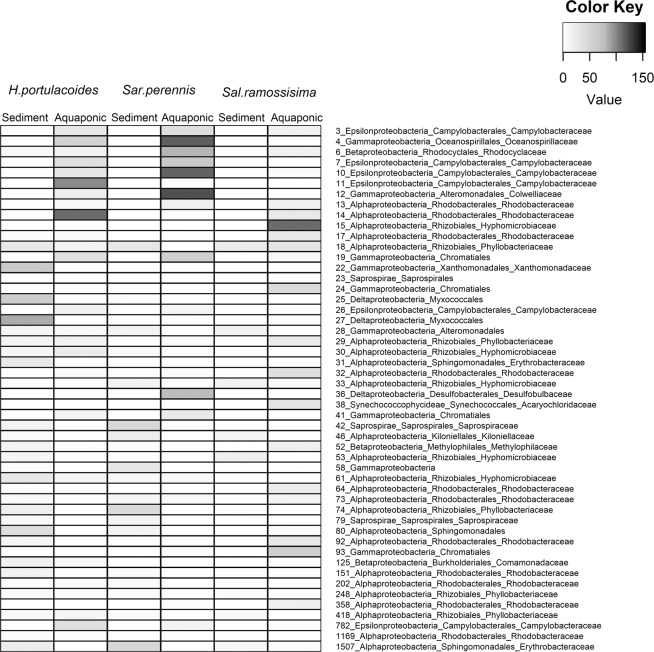
Heatmap showing the abundance of dominant 16S rRNA sequence reads (≥50 sequences) in the endosphere of each halophyte plant species grown in aquaponics or sediment.

The in-depth bacterial composition analysis detected fifty abundant OTUs (≥50 sequences, Figs. 5 and 6) that were assigned to three phyla: *Bacteroidetes*, *Cyanobacteria* and *Proteobacteria*. The taxonomic assignment of these abundant OTUs and their known ecophysiological traits are given in Table 1. The most abundant OTU overall was OTU 6, which was enriched in aquaponic samples, and was assigned to the family *Rhodocyclaceae* (*Betaproteobacteria*) and closely related to the genus *Denitromonas* that was isolated from a marine aquaculture (GI: 115334100, Table S10). Members of this genus have been associated with denitrifying processes in a biofilm developed in a recirculating marine aquaculture system aerated filter^36^. Only one abundant OTU (OTU 38), recorded in *S. ramosissima* aquaponic biotopes, was assigned to the phylum *Cyanobacteria*; this OTU was closely related to an *Acaryochloris* strain isolated from a red alga^37^. The fact that this OTU was only detected in Salicornia tank may be explained by higher water temperature recorded in this tank (23 °C) compared to the other tanks (21 °C). The availability of nutrient-rich effluents from aquaculture combined with higher water temperatures may have promoted the growth of cyanobacteria in *Salicornia* rhizosphere and endosphere^38,39^. Of the three abundant OTUs assigned to phylum *Bacteroidetes*, two were restricted to sediment samples from *H. portulacoides* and *S. ramossisima* (OTU 42 and 79) and were related to a *Lewinella* sp. strain isolated from seawater (GI: 530549991, Table S12). Members of this genus are aerobic chemo-organotrophic that require NaCl for growth^40^. The restricted presence of members of the phylum *Bacteroidetes* in the sediments samples could be explain by the fact that they are specialized in degrading complex carbon sources common found in this type of sediments^41^. Furthermore, they have shown to have a clear preference for growth attached to surfaces or particles (e.g. sediments)^41^. All the main proteobacterial classes were well represented in the present study (*Alphaproteobacteria*, *Deltaproteobacteria*, *Epsilonproteobacteria*, *Gammaproteobacteria* and *Betaproteobacteria*; Table 1) with the majority of dominant OTUs assigned to *Alphaproteobacteria*. This class includes several plant symbionts^42^. Within the class *Alphaproetobacteria*, several dominant OTUs were closely related to members assigned to the Hyphomicrobiaceae family, which includes strains known to be involved in denitrification^43^. Another group of dominant OTUs (18, 29, 74, 248 and 418), assigned to family *Phyllobacteriaceae*, were enriched in the root endophytic community, and were closely related to *Mesorhizobium* sp. that was isolated from a root endophytic microbiome (GI: 725096748, Table S12) and an endophytic bacterium isolated from the roots of the salt marsh plant *Spartina alterniflora*^44^. The genus *Mesorhizobium* is known to include root-nodule bacteria that can establish nitrogen-fixing symbiosis with plants^45^. *Gammaproteobacteria* were the second most abundant proteobacterial class. The phylogenetic analysis showed that these OTUs were closely related to uncultured *Gammaproteobacteria* or species within the genera *Colwellia* or *Dokdonella* (SI Table S12). These genera include bacterial guilds often associated with nitrate reduction^46,47^.

**Table 1:** Taxonomic assignment of partial 16S rRNA gene sequences of dominant bacterial populations (operational taxonomic units > 50 reads) and their known putative ecophysiological traits.

OTU code/number of reads	Sequence classification	Known traits
	**Bacteroidetes**	
**23**/68 **42**/87, **79**/59	Saprospirales *Lewinella*	The Saprospiraceae family includes stains isolated from aquatic environments and members of this family have the ability for the hydrolysis and utilization of complex carbon sources. The genus *Lewinella* includes a few isolates retrieved from marine sources which were aerobic chemo-organotrophic that require NaCl to growth.
	**Cyanobacteria**	
**38**/50	Acaryochloridaceae *Acaryochloris*	Members of the *Acaryochloris* genus (Cyanobacteria) contain chlorophyll d as a major photopigment and are found widely distributed in both aquatic and terrestrial ecological systems.
	**Proteobacteria**	
	**Alphaproteobacteria**	
**80**/114 **31**/75, **1507**/164	Sphingomonadales Erythrobacteraceae	Members of Erythrobacteraceae family (Sphingomonadales) consist of aerobic chemoorganotrophs that are mainly isolated from aquatic environments, but there are also isolation reports from sediment, sand, and rice.
**33**/72 **30**/74, **53**/52, **61**/92 **15**/143	Hyphomicrobiaceae *Devosia* *Hyphomicrobium*	The family Hyphomicrobiaceae is phenotypically, metabolically, and ecologically diverse. *Devosia* genus comprises non-photosynthetic bacteria which have been mainly isolated from terrestrial soil sources. Members of *Hyphomicrobium* genus are anerobic facultative methylotrophs, reproduce by budding and are ubiquitous in water and soils, but can also be found in sewage treatment plants. Some strains are characterized by their denitrification capacities.
**46**/52 **18**/219, **29**/115, **248**/51, **418**/69	Kiloniellaceae Thalassospira Phyllobacteriaceae *Mezorhizobium*	Members from Kiloniellales order (Kiloniellaceae family) may be involved in nutrient cycle, possible in denitrification. Several OTU’s assigned to order Rhizobiales were related to the family Phyllobacteriaceae. Members of this family are versatile environmental bacteria that occur in diverse habitats that often are polluted or nutritionally rather rich. *Mesorhizobium* spp. are well known as root-nodule bacteria, which fix atmospheric nitrogen.
**74**/94 **13**/161, **64**/99, **151**/86, **202**/60, **358**/100, **1169**/53 **73**/78 **14**/271 **17**/103 **32**/142 **92**/127	Rhodobacteraceae *Jannaschia* *Loktanella* *Marivita* *Octadecabacter* *Phaeobacter*	Rhodobacteraceae are, fundamentally, aquatic bacteria that frequently thrive in marine environments, and are deeply involved in sulfur and carbon biogeochemical cycling. The *Jannaschia* genus are isolated from marine environments (seawater, seashore sand, tidal flat sediment and solar saltern water). The genus *Loktanella* have been isolated from Antarctic lakes and marine environments. *Marivita* genus consist in six species, one of theme isolated from estuarine water, that contain genes for bacteriochlorophyll a synthesis (although some of them do not produce the pigment). Only two species of *Octadecabacter* are described and both isolated from polar regions. The genus *Phaeobacter* includes a few isolates, and are candidates to be used as probiotics in marine aquaculture because their ability to efficiently adhere and grow on surfaces and to secrete inhibitors compounds that enable them to antagonize invertebrate settlement and algal or microbial growth
	**Betaproteobacteria**	
**6**/283	Rhodocyclaceae	Members of Rhodocyclaceae family have been isolated from diverse environments: soil; sewage treatment plants; polluted and unpolluted waters of ponds, rivers, aquifers and plant roots. They are capable to degrade a wide range of carbon sources including many aromatic compounds and fix nitrogen in association with plants.
**125**/55	Comamonadaceae *Hydrogenophaga*	Hydrogenophaga comprises hydrogen-oxidizing bacteria found in aquatic or soil habitats and activated sludge.
**52**/55	Methylophilaceae *Methylotenera*	The family Methylophilaceae includes obligate or restricted facultative methylotrophs such as *Methylotenera* spp. This family belong to a group of methylobacteria, which play an important role for the aerobic conversion of C1 compounds in different ecological niches.
	**Deltaproteobacteria**	
**25**/107, **27**/93 **36**/105	Myxococcales Desulfobulbaceae	Myxococcales members are known for their complex life cycle and the ability to produce natural products with unique structures and bioactivities. Most members of Desulfobulbaceae family are mesophilic sulfate-reducing bacteria found in marine, brackish or freshwater habitats and are important players in the process of anoxic mineralization of organic matter.
	**Epsilonproteobacteria**	
**3**/179, **7**/195, **10**/2 07, **11**/272, **26**/53, **782**/112	Campylobacteraceae *Arcobacter*	*Arcobacter* species have been previously recovered from different hosts and environments. Some members are considered as potential water and food-borne pathogens. Members of this genus are involved in the fixation of nitrogen.
**58**/71	**Gammaproteobacteria**	
**28**/69 **12**/174	Alteromonadales Colwelliaceae	The class Gammaproteobacteria comprise a diverse group of bacteria that exhibits enormous variety in terms of their phenotype and metabolic capabilities. Many species of Alteromonadales order are involved in important functions in the environment, such as carbon and sulfur cycling, degradation of hydrocarbons and production of inhibitory molecules. Members of Colwelliaceae family are strictly marine secondary producers and are involved in organic material decomposition (hydrocarbons, lipids, proteins, polysaccharides).
**19**/136, **24**/90, **41**/66, **93**/81 **4**/227 **22**/122	Chromatiales Oceanospirillaceae Xanthomonadaceae *Dokdonella*	The order Chromatiales includes the phototrophic purple sulfur bacteria able to perform photosynthesis under anoxic conditions without oxygen production. Almost Oceanospirillaceae members are aerobic, halotolerant or halophilic marine bacteria, which are able to utilize various carbohydrate and amino acid compounds, as a sole carbon and energy source. Members of *Dokdonella* (Xanthomonadaceae) genus include few isolates retrieved, mostly, from soil samples.
The codes in bold refer to the OTU’s code (≥ 50 sequences) followed by the number of sequences reads assigned to each OTU.		

The codes in bold refer to the OTU’s code (≥50 sequences) followed by the number of sequences reads assigned to each OTU.

Fourteen abundant OTUs were restricted to plants grown in aquaponics (OTUs 4, 7, 10, 11, 12, 13, 19, 23, 36, 38, 41, 64, 358 and 782) (Table S11), while five OTUs were restricted to plants grown in sediment (OTUs 22, 27, 42, 58 and 79) (Table S12). In aquaponics, the order *Campylobacterales* was the most abundant detected, mainly in *H. portulacoides* and *S. perennis* samples and assigned to the genus *Arcobacter*. Members of this genus isolated from the roots of *Spartina alterniflora*, a salt marsh plant, have been shown to be capable of nitrogen fixation^48^. *Rhodobacterales* was the second most abundant order of *Proteobacteria* recorded in samples from aquaponics. Within this order, family *Rhodobacteraceae* was the most prevalent in this study and is characterized by highly diverse metabolisms that include sulfur-oxidizing species in freshwater and marine environments^49^. *Desulfobacterales* were markedly more abundant in the endophytic community of *S. perennis* in aquaponics. This order includes sulfate-reducing bacteria which are important players in anoxic mineralization of organic matter^50^. However, the reasons why members of this order was markedly more abundant in *S. perennis* endophytic community need further investigation.

Results from the present study showed that specific proteobacterial groups involved in nutrient cycling, namely, nitrogen fixation, denitrification and mineralization of organic matter, were enriched in root associated bacterial communities in aquaponics. The more prevalent bacterial groups in the roots of plants grown in sediment included the *Rhizobiales* and the *Sphingomonadales*. While the first is well-known for its ability to fix atmospheric nitrogen in association with plants, the second includes aerobic chemeorganotrophs isolated from diverse environments, such as seawater, tidal flats and marine sediment^51^. The endophytic community of *S. ramosissima* in the sediment was enriched with *Alteromonadales*, which are a relatively common marine group of chemoheterotrophs that are known to be important player in carbon and sulfur cycling^52^.

## Concluding Remarks

In this study we applied a combined DGGE and pyrosequencing approach to assess the bacterial community present in the root system of three halophyte plants: *Halimione portulacoides*, *Salicornia ramosissima* and *Sarcocornia perennis*.

Overall, our study revealed the existence of a plant species-specific effect on the profile and diversity of bacterial communities present in the rhizosphere and endosphere. Moreover, significant differences were also revealed in the bacterial composition present in the rhizosphere and endosphere of conspecific plants grown in contrasting environments (aquaponics *vs*. sediment). The composition analysis performed showed an enrichment in the bacterial taxa of halophytes produced in aquaponics, namely those involved in nutrient cycling, such as sulfur, carbon cycles nitrogen fixation and denitrification processes.

## Supplementary information

Supplementary Dataset 1.

## References

[1] Fisheries, F. Aquaculture Department (2010) The state of world fisheries and aquaculture. Food and Agriculture Organization of the United Nations, Rome 2016 (2010).

[2] Tal Y (2009). Environmentally sustainable land-based marine aquaculture. Aquaculture.

[3] Piedrahita RH (2003). Reducing the potential environmental impact of tank aquaculture effluents through intensification and recirculation. Aquaculture.

[4] van Rijn J (2013). Waste treatment in recirculating aquaculture systems. Aquacult. Eng..

[5] Martins C (2010). New developments in recirculating aquaculture systems in Europe: A perspective on environmental sustainability. Aquacult. Eng..

[6] Chen X (2017). A novel combined recirculating treatment system for intensive marine aquaculture. Aquacult. Res..

[7] Worsfold PJ (2008). Characterisation and quantification of organic phosphorus and organic nitrogen components in aquatic systems: a review. Anal. Chim. Acta.

[8] Chopin, T. *et al*. Ecological engineering: Multi-trophic integration for sustainable marine aquaculture. (Elsevier, Oxford (2008).

[9] Somerville, C., Cohen, M., Pantanella, E., Stankus, A. & Lovatelli, A. Small-scale aquaponic food production: integrated fish and plant farming. FAO Fisheries and Aquaculture Technical Paper, I (2014).

[10] dos Santos MJPL (2016). Smart cities and urban areas—Aquaponics as innovative urban agriculture. Urban For Urban. Green.

[11] Troell M (2009). Ecological engineering in aquaculture—potential for integrated multi-trophic aquaculture (IMTA) in marine offshore systems. Aquaculture.

[12] Chopin T, Cooper JA, Reid G, Cross S, Moore C (2012). Open‐water integrated multi-trophic aquaculture: environmental biomitigation and economic diversification of fed aquaculture by extractive aquaculture. Rev. Aquacult..

[13] Buhmann AK, Waller U, Wecker B, Papenbrock J (2015). Optimization of culturing conditions and selection of species for the use of halophytes as biofilter for nutrient-rich saline water. Agric. Water Manag..

[14] Buhmann A, Papenbrock J (2013). Biofiltering of aquaculture effluents by halophytic plants: Basic principles, current uses and future perspectives. Environ. Exp. Bot..

[15] De Lange H, Paulissen M (2016). Efficiency of three halophyte species in removing nutrients from saline water: a pilot study. Wetl. Ecol. Manag..

[16] De Lange H, Paulissen M, Slim P (2013). ‘Halophyte filters’: the potential of constructed wetlands for application in saline aquaculture. Int. J. Phytoremediation.

[17] Custódio M, Villasante S, Cremades J, Calado R, Lillebø AI (2017). Unravelling the potential of halophytes for marine integrated multi-trophic aquaculture (IMTA) a perspective on performance, opportunities and challenges. Aquac. Environ. Interact..

[18] Marques B, Calado R, Lillebø AI (2017). New species for the biomitigation of a super-intensive marine fish farm effluent: Combined use of polychaete-assisted sand filters and halophyte aquaponics. Sci. Total Environ..

[19] Münch, C., Neu, T., Kuschk, P. & Röske, I. The root surface as the definitive detail for microbial transformation processes in constructed wetlands–a biofilm characteristic. *Water Sci. Technol*. **56** (2007). 10.2166/wst.2007.527 17802865

[20] Xiaona, M., *et al*. Characterization of microbial communities in pilot-scale constructed wetlands with Salicornia for treatment of marine aquaculture effluents. *Archaea* (2018). 10.1155/2018/7819840 PMC5949191 29853796

[21] Gomes NCM (2008). Exploring the diversity of bacterial communities in sediments of urban mangrove forests. FEMS Microbial. Ecol..

[22] Weisburg WG, Barns SM, Pelletier DA, Lane DJ (1991). 16S ribosomal DNA amplification for phylogenetic study. J. Bacteriol..

[23] Heuer H (2001). Bacterial community profiling using DGGE or TGGE analysis. Environmental molecular microbiology: Protocols and applications.

[24] Oliveira V (2014). Halophyte plant colonization as a driver of the composition of bacterial communities in salt marshes chronically exposed to oil hydrocarbons. FEMS Microbiol. Ecol..

[25] Cleary DF (2013). Habitat-and host-related variation in sponge bacterial symbiont communities in Indonesian waters. FEMS Microbiol. Ecol..

[26] Cleary DF, de Voogd NJ, Polónia AR, Freitas R, Gomes NC (2015). Composition and predictive functional analysis of bacterial communities in seawater, sediment and sponges in the Spermonde Archipelago, Indonesia. Microb. Ecol..

[27] Warnes, G. R. *et al*. gplots: various R programming tools for plotting data. R package version 2.17. 0. Computer software]. Available online at: http://CRAN.R-project.org/package= gplots (2015).

[28] Oksanen J (2013). vegan: Community Ecology Package. R package version.

[29] Zhang Z, Schwartz S, Wagner L, Miller W (2000). A greedy algorithm for aligning DNA sequences. J. Comput. Biol..

[30] Calheiros CS (2009). Changes in the bacterial community structure in two-stage constructed wetlands with different plants for industrial wastewater treatment. Bioresour. Technol.

[31] Bulgarelli D (2012). Revealing structure and assembly cues for Arabidopsis root-inhabiting bacterial microbiota. Nature.

[32] Jiang X-T (2013). Illumina sequencing of 16S rRNA tag revealed spatial variations of bacterial communities in a mangrove wetland. Microb. Ecol..

[33] Lau JA, Lennon JT (2012). Rapid responses of soil microorganisms improve plant fitness in novel environments. Proceedings of the National Academy of Sciences.

[34] Kersters, K. *et al*. In The prokaryotes. 3-37 (Springer (2006).

[35] Xu J (2003). A genomic view of the human-Bacteroides thetaiotaomicron symbiosis. Science.

[36] Gao X-Y, Xu Y, Liu Y, Liu Z-P (2012). Bacterial diversity, community structure and function associated with biofilm development in a biological aerated filter in a recirculating marine aquaculture system. Mar. Biodivers..

[37] Murakami A, Miyashita H, Iseki M, Adachi K, Mimuro M (2004). Chlorophyll d in an epiphytic cyanobacterium of red algae. Science.

[38] Nogueira SMS, Souza Junior J, Maia HD, Saboya JPS, Farias WRL (2018). Use of Spirulina platensis in treatment of fish farming wastewater. Revista Ciência Agronômica.

[39] Srimongkol P, Thongchul N, Phunpruch S, Karnchanatat A (2019). Ability of marine cyanobacterium Synechococcus sp. VDW to remove ammonium from brackish aquaculture wastewater. Agric. Water Manag..

[40] Khan ST, Fukunaga Y, Nakagawa Y, Harayama S (2007). Emended descriptions of the genus Lewinella and of Lewinella cohaerens, Lewinella nigricans and Lewinella persica, and description of Lewinella lutea sp. nov. and Lewinella marina sp. nov. Anglais.

[41] Fernández-Gomez B (2013). Ecology of marine Bacteroidetes: a comparative genomics approach. The. ISME journal.

[42] Garrity, G. M., Bell, J. A. & Lilburn, T. Alphaproteobacteria class. nov. In *Bergey’s Manual® of Systematic Bacteriology* 1-574 (Springer (2005).

[43] Mills HJ (2008). Characterization of nitrifying, denitrifying, and overall bacterial communities in permeable marine sediments of the northeastern Gulf of Mexico. Appl. Environ. Microbiol..

[44] Kandalepas D, Blum MJ, Van Bael SA (2015). Shifts in symbiotic endophyte communities of a foundational salt marsh grass following oil exposure from the Deepwater Horizon oil spill. PloS one.

[45] Willems, A., The Family Phyllobacteriaceae. *The Prokaryotes: Alphaproteobacteria and Betaproteobacteria*. 355-418 (2014).

[46] Jung S-Y, Oh T-K, Yoon J-H (2006). Colwellia aestuarii sp. nov., isolated from a tidal flat sediment in Korea. Anglais.

[47] Yoo S-H (2009). Dokdonella soli sp. nov., a gammaproteobacterium isolated from soil. Anglais.

[48] Pati A (2010). Complete genome sequence of Arcobacter nitrofigilis type strain (CIT). Stand. Genomic Sci..

[49] Pujalte, M. J., Lucena, T., Ruvira, M. A., Arahal, D. R. & Macián, M. C. The family Rhodobacteraceae. *The Prokaryotes: Alphaproteobacteria and Betaproteobacteria*, 439-512 (2014).

[50] Gomes NC (2010). Taking root: enduring effect of rhizosphere bacterial colonization in mangroves. Plos one.

[51] Tonon, L. A. C., Moreira, A. P. B. & Thompson, F. In The Prokaryotes. 213-235 (Springer (2014).

[52] Bowman, J. P. & McMeekin, T. A. In Bergey’s Manual® of Systematic Bacteriology. 443-491 (Springer 2005).

