# Author Correction: Aquaponics using a fish farm effluent shifts bacterial communities profile in halophytes rhizosphere and endosphere

**DOI:** 10.1038/s41598-020-77251-3

**Published:** 2020-11-18

**Authors:** Vanessa Oliveira, Patrícia Martins, Bruna Marques, Daniel F. R. Cleary, Ana I. Lillebø, Ricardo Calado

**Affiliations:** 1 CESAM - Centre for Environmental and Marine Studies, Departament of Biology, University of Aveiro, Campus Universitário de Santiago, 3810-193 Aveiro, Portugal; 2 ECOMARE, CESAM - Centre for Environmental and Marine Studies, Departament of Biology, University of Aveiro, Campus Universitário de Santiago, 3810-193 Aveiro, Portugal

Correction to: *Scientific Reports* 10.1038/s41598-020-66093-8, published online 22 June 2020

This Article contains errors in the accompanying Supplementary Dataset, where Supplementary Tables S1 to S12 were omitted.

The corrected Supplementary Dataset 1 file is linked to this correction notice.

## Supplementary information

Supplementary Dataset 1.

