# Supplementary material for: Aquaponics using a fish farm effluent shifts bacterial communities profile in halophytes rhizosphere and endosphere
Source: Sci Rep. 2020 Jun 22;10:10023. doi: 10.1038/s41598-020-66093-8 (PMC7308282; doi:10.1038/s41598-020-66093-8)
Supplement: Supplementary file 1 — Supplementary Dataset 1. [file 41598_2020_66093_MOESM1_ESM.pdf]

Aquaponics using a fish farm effluent shifts bacterial communities profile in halophytes rhizosphere and endosphere

Vanessa Oliveira[1], Patrícia Martins[1], Bruna Marques[2], Daniel F. R. Cleary[1], Ana I. Lillebø[2*] & Ricardo Calado[2*]

[1]Departamento de Biologia & CESAM, Campus Universitário de Santiago, Universidade de Aveiro, 3810-193 Aveiro Portugal

[2]Departamento de Biologia & CESAM & ECOMARE, Campus Universitário de Santiago, Universidade de Aveiro, 3810-193 Aveiro Portugal

Correspondence and requests for materials should be addressed to A.I.L (email: lillebo@ua.pt) or R.C. (email: rjcalado@ua.pt)

Supplementary information

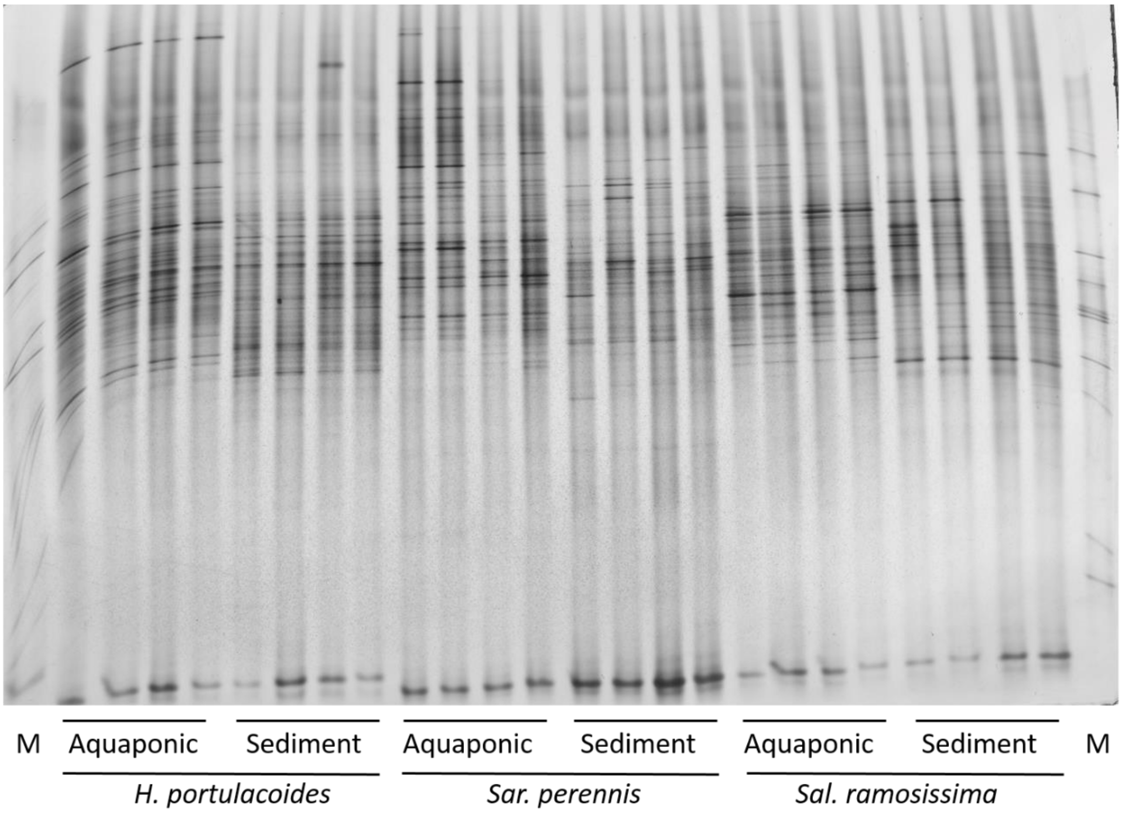

Figure S1. DGGE profiles of halophytes rhizosphere community between grow-out environments (sediment *vs.* aquaponics) (full-length gel).

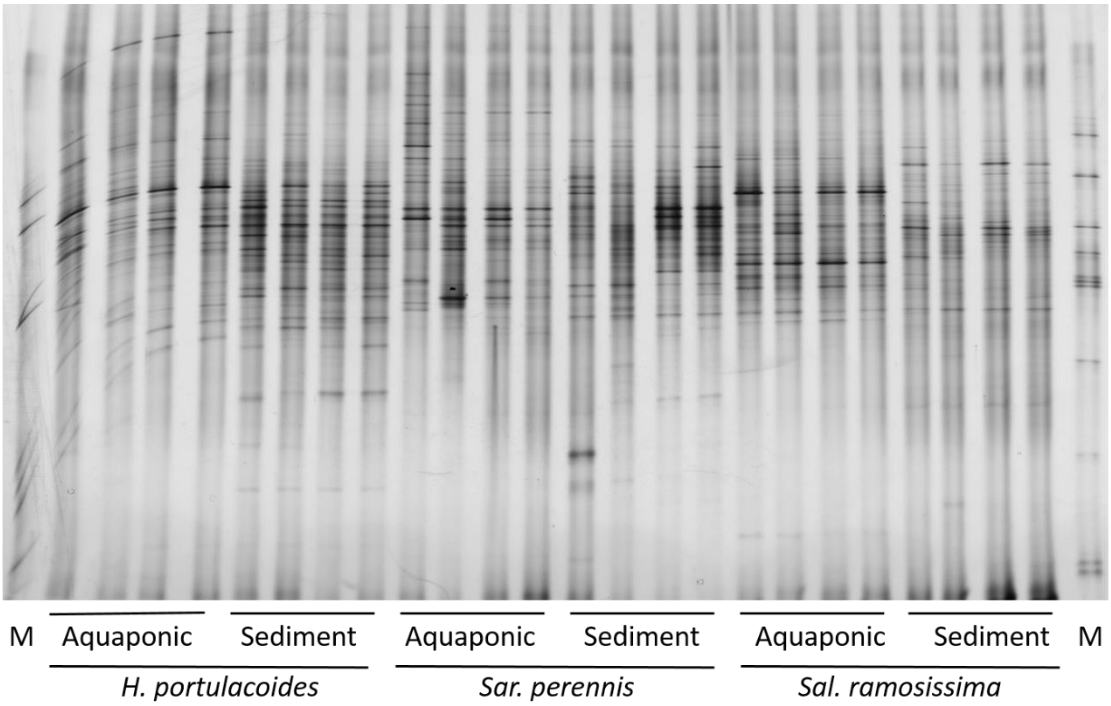

Figure S2. DGGE profiles of halophytes endosphere community between grow-out environments (sediment *vs.* aquaponics) (full-length gel).
