# Supplementary material for: Author Correction: Aquaponics using a fish farm effluent shifts bacterial communities profile in halophytes rhizosphere and endosphere
Source: Sci Rep. 2020 Nov 18;10:20356. doi: 10.1038/s41598-020-77251-3 (PMC7672088; doi:10.1038/s41598-020-77251-3)
Supplement: Supplementary file 1 — Supplementary Dataset 1. [file 41598_2020_77251_MOESM1_ESM.pdf]

Aquaponics using a fish farm effluent shifts the bacterial communities of halophytes rhizosphere and endosphere

Vanessa Oliveira[1], Patrícia Martins[1], Bruna Marques[2], Daniel F. R. Cleary[1], Ana I. Lillebø[2*] & Ricardo Calado[2*]

[1]Departamento de Biologia & CESAM, Campus Universitário de Santiago, Universidade de Aveiro, 3810-193 Aveiro Portugal

[2]Departamento de Biologia & CESAM & ECOMARE, Campus Universitário de Santiago, Universidade de Aveiro, 3810-193 Aveiro Portugal

Correspondence and requests for materials should be addressed to A.I.L (email: lillebo@ua.pt) or R.C. (email: rjcalado@ua.pt)

Supplementary information

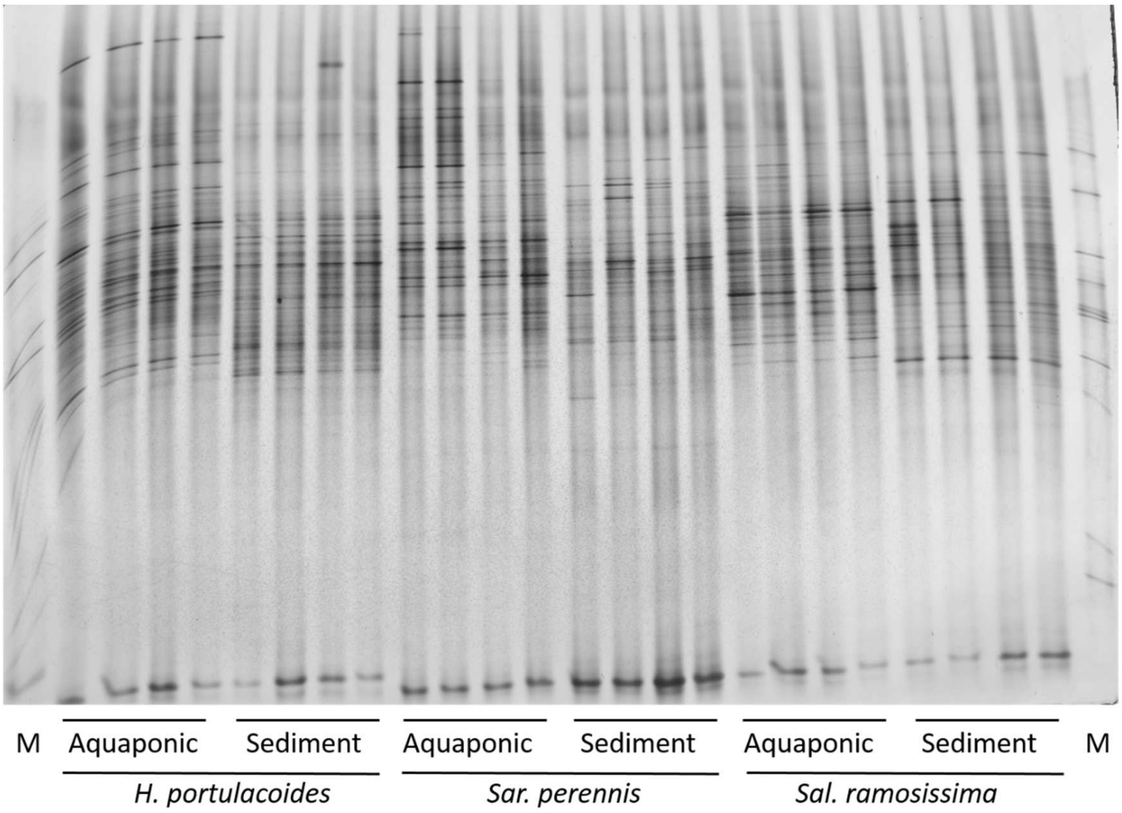

Figure S1. DGGE profiles of halophytes rhizosphere community between grow-out environments (sediment *vs.* aquaponics) (full-length gel).

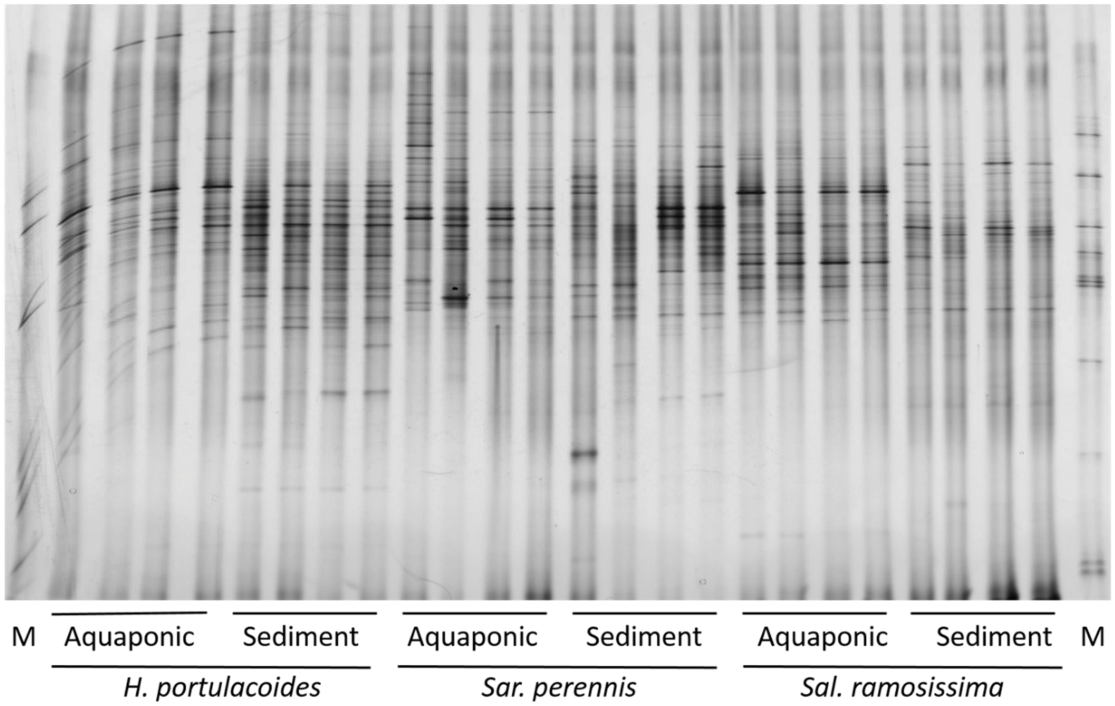

Figure S2. DGGE profiles of halophytes endosphere community between grow-out environments (sediment *vs.* aquaponics) (full-length gel).

Table S1. PERMANOVA analysis resume and pair-wise tests for bacterial DGGE profiles recorded for rhizosphere.

**Source**	**df**	**SS**	**MS**	**Pseudo-F**	**P(perm)**	**Unique perms**
Plant species	2	13669	6834.6	9.7012	0.0001	9904
Environment	1	6982.1	6982.1	9.9105	0.0001	9927
Plant speciesxEnvironment	2	10980	5490.2	7.7929	0.0001	9922
Residuals	18	12681	704.51			
Total	23	44313				

PAIR-WISE TESTS

Term “Plant speciesxEnvironment“ for pairs of levels of factor 'Environment' - Within level '*H.potulacoides*' of factor 'Plant species'	Sediment, Aquaponics			3.1453	0.0264	35
Term “Plant speciesxEnvironment“ for pairs of levels of factor 'Environment' - Within level '*Sar. perennis*' of factor 'Plant species'	Sediment, Aquaponics			2.6237	0.0274	35
Term “Plant speciesxEnvironment“ for pairs of levels of factor 'Environment' - Within level '*Sal. ramosissima*' of factor 'Plant species'	Sediment, Aquaponics			3.1043	0.0287	35
Term “Plant speciesxEnvironment“ for pairs of levels of factor 'Plant species' - Within level '*Sediment*' of factor 'Environment'	H, Sar			2.9937	0.0285	35
	H, Sal			3.3765	0.0302	35
	Sar, Sal			2.3204	0.0305	35
Term “Plant speciesxEnvironment“ for pairs of levels of factor 'Plant species' - Within level '*Aquaponics*' of factor 'Environment'	H, Sar			3.0377	0.0285	35
	H, Sal			3.3548	0.0264	35
	Sar, Sal			2.8513	0.0276	35

Table S2. PERMANOVA analysis resume and pair-wise tests for bacterial DGGE profiles recorded for endosphere.

**Source**	**df**	**SS**	**MS**	**Pseudo-F**	**P(perm)**	**Unique perms**
Plant species	2	10051	5025.6	6.8799	0.0001	9912
Environment	1	4250.7	4250.7	5.8191	0.0001	9919
Plant speciesxEnvironment	2	8826.3	4413.1	6.0415	0.0001	9923
Residuals	18	13149	730.47			
Total	23	36277				

PAIR-WISE TESTS						
Term " EnvironmentxPlant species " for pairs of levels of factor 'Environment' - Within level '*H.potulacoides*' of factor 'Plant species'	Sediment, Aquaponics			2.2821	0.0282	35
Term "EnvironmentxPlant species" for pairs of levels of factor 'Environment' - Within level '*Sar. perennis*' of factor 'Plant species'	Sediment, Aquaponics			1.879	0.0298	35
Term "EnvironmentxPlant species" for pairs of levels of factor 'Environment' - Within level '*Sal. ramosissima*' of factor 'Plant species'	Sediment, Aquaponics			3.4042	0.0263	35
Term " EnvironmentxPlant species " for pairs of levels of factor 'Plant species' - Within level '*Sediment*' of factor 'Environment'	H, Sar			1.9503	0.0305	35
	H, Sal			2.8165	0.0293	35
	Sar, Sal			1.991	0.0306	35
Term " EnvironmentxPlant species " for pairs of levels of factor 'Plant species' - Within level '*Aquaponics*' of factor 'Environment'	H, Sar			2.2065	0.0302	35
	H, Sal			3.1964	0.0263	35
	Sar, Sal			3.2162	0.0332	35

Table S3. OTU table with taxonomic assignment generated by QIIME (Rhiz: Rhizosphere; End: Endosphere).

		H. portulacoides				***Sal.ramossisima***				***Sar. Perennis***									
		**Aquaponic**		**Sediment**		**Aquaponic**		**Sediment**		**Aquaponic**		**Sediment**							
**OTU**	**SUM**	**Rhiz**	**End**	**Rhiz**	**End**	**Rhiz**	**End**	**Rhiz**	**End**	**Rhiz**	**End**	**Rhiz**	**End**	**Phylum**	**Class**	**Order**	**Family**	**Genus**	**Species**
3	179	29	27	2	3	25	17	0	0	38	38	0	0	Proteobacteria	Epsilonproteobacteria	Campylobacterales	Campylobacteraceae	Arcobacter	Unassigned
11	272	155	98	0	0	7	1	0	0	9	2	0	0	Proteobacteria	Epsilonproteobacteria	Campylobacterales	Campylobacteraceae	Arcobacter	Unassigned
14	271	92	111	7	4	22	26	3	0	1	1	4	0	Proteobacteria	Alphaproteobacteria	Rhodobacterales	Rhodobacteraceae	Loktanella	Unassigned
7	195	44	37	0	0	15	1	0	0	33	65	0	0	Proteobacteria	Epsilonproteobacteria	Campylobacterales	Campylobacteraceae	Arcobacter	Unassigned
6	283	33	16	0	9	82	18	2	3	38	81	1	0	Proteobacteria	Betaproteobacteria	Rhodocyclales	Rhodocyclaceae	Unassigned	Unassigned
4	227	35	51	0	0	1	0	0	0	25	115	0	0	Proteobacteria	Gammaproteobacteria	Oceanospirillales	Oceanospirillaceae	Unassigned	Unassigned
10	207	9	31	0	0	6	6	0	0	41	114	0	0	Proteobacteria	Epsilonproteobacteria	Campylobacterales	Campylobacteraceae	Arcobacter	Unassigned
5	12	1	1	0	0	2	1	0	0	2	5	0	0	Proteobacteria	Alphaproteobacteria	BD7-3	Unassigned	Unassigned	Unassigned
18	219	23	14	17	28	31	32	7	24	4	1	10	28	Proteobacteria	Alphaproteobacteria	Rhizobiales	Phyllobacteriaceae	Unassigned	Unassigned
9	9	6	0	0	0	3	0	0	0	0	0	0	0	Proteobacteria	Gammaproteobacteria	Alteromonadales	Alteromonadaceae	Glaciecola	punicea
12	174	11	21	0	0	0	0	0	0	20	122	0	0	Proteobacteria	Gammaproteobacteria	Alteromonadales	Colwelliaceae	Unassigned	Unassigned
1169	53	5	3	2	4	35	1	0	0	2	0	0	1	Proteobacteria	Alphaproteobacteria	Rhodobacterales	Rhodobacteraceae	Unassigned	Unassigned
32	142	35	5	0	1	47	37	0	0	9	5	3	0	Proteobacteria	Alphaproteobacteria	Rhodobacterales	Rhodobacteraceae	Octadecabacter	Unassigned
17	103	16	7	4	4	34	27	3	1	4	2	1	0	Proteobacteria	Alphaproteobacteria	Rhodobacterales	Rhodobacteraceae	Marivita	Unassigned
358	100	2	0	0	0	76	19	0	0	1	2	0	0	Proteobacteria	Alphaproteobacteria	Rhodobacterales	Rhodobacteraceae	Unassigned	Unassigned
1507	164	2	0	19	23	8	3	4	10	1	0	47	47	Proteobacteria	Alphaproteobacteria	Sphingomonadales	Erythrobacteraceae	Unassigned	Unassigned
13	161	38	9	0	0	53	18	0	0	23	20	0	0	Proteobacteria	Alphaproteobacteria	Rhodobacterales	Rhodobacteraceae	Unassigned	Unassigned
26	53	12	16	1	0	7	3	0	0	2	11	1	0	Proteobacteria	Epsilonproteobacteria	Campylobacterales	Campylobacteraceae	Arcobacter	Unassigned
92	127	31	1	8	11	38	25	3	1	4	2	3	0	Proteobacteria	Alphaproteobacteria	Rhodobacterales	Rhodobacteraceae	Phaeobacter	Unassigned
782	112	31	38	0	0	17	4	0	0	8	14	0	0	Proteobacteria	Epsilonproteobacteria	Campylobacterales	Campylobacteraceae	Arcobacter	Unassigned
19	136	10	37	0	0	10	9	0	0	13	57	0	0	Proteobacteria	Gammaproteobacteria	Chromatiales	Unassigned	Unassigned	Unassigned
15	143	8	3	0	0	20	111	0	1	0	0	0	0	Proteobacteria	Alphaproteobacteria	Rhizobiales	Hyphomicrobiaceae	Hyphomicrobium	Unassigned
64	99	39	3	0	0	30	22	0	0	4	1	0	0	Proteobacteria	Alphaproteobacteria	Rhodobacterales	Rhodobacteraceae	Unassigned	Unassigned
22	122	0	0	50	62	0	0	0	1	0	0	8	1	Proteobacteria	Gammaproteobacteria	Xanthomonadales	Xanthomonadaceae	Dokdonella	Unassigned
29	115	31	17	18	13	5	22	0	0	2	6	0	1	Proteobacteria	Alphaproteobacteria	Rhizobiales	Phyllobacteriaceae	Unassigned	Unassigned
80	114	3	0	38	40	6	5	2	1	0	0	6	13	Proteobacteria	Alphaproteobacteria	Sphingomonadales	Unassigned	Unassigned	Unassigned
21	0	0	0	0	0	0	0	0	0	0	0	0	0	TM6	SJA-4	Unassigned	Unassigned	Unassigned	Unassigned
20	31	6	2	0	0	8	12	0	0	2	1	0	0	Proteobacteria	Alphaproteobacteria	Rhizobiales	Cohaesibacteraceae	Unassigned	Unassigned
23	68	14	0	0	0	45	4	0	0	4	1	0	0	Bacteroidetes	[Saprospirae]	[Saprospirales]	Unassigned	Unassigned	Unassigned
25	107	6	3	28	59	0	0	0	0	0	0	7	4	Proteobacteria	Deltaproteobacteria	Myxococcales	Unassigned	Unassigned	Unassigned
36	105	0	3	0	0	5	0	0	0	19	78	0	0	Proteobacteria	Deltaproteobacteria	Desulfobacterales	Desulfobulbaceae	Unassigned	Unassigned
151	86	1	0	7	16	12	2	6	2	0	1	33	6	Proteobacteria	Alphaproteobacteria	Rhodobacterales	Rhodobacteraceae	Unassigned	Unassigned
74	94	0	0	8	20	0	1	1	3	0	1	16	44	Proteobacteria	Alphaproteobacteria	Rhizobiales	Phyllobacteriaceae	Mesorhizobium	Unassigned
27	93	0	0	7	85	0	0	0	0	0	0	1	0	Proteobacteria	Deltaproteobacteria	Myxococcales	Unassigned	Unassigned	Unassigned
24	90	0	0	1	0	47	42	0	0	0	0	0	0	Proteobacteria	Gammaproteobacteria	Chromatiales	Unassigned	Unassigned	Unassigned
61	92	9	11	22	27	0	0	0	1	3	7	4	8	Proteobacteria	Alphaproteobacteria	Rhizobiales	Hyphomicrobiaceae	Devosia	Unassigned
42	87	0	0	7	13	0	0	0	0	0	0	22	45	Bacteroidetes	[Saprospirae]	[Saprospirales]	Saprospiraceae	Lewinella	Unassigned
1341	13	9	2	0	0	2	0	0	0	0	0	0	0	Proteobacteria	Epsilonproteobacteria	Campylobacterales	Campylobacteraceae	Arcobacter	Unassigned
73	78	5	1	4	7	7	14	0	0	5	8	16	11	Proteobacteria	Alphaproteobacteria	Rhodobacterales	Rhodobacteraceae	Jannaschia	Unassigned

93	81	1	1	5	0	11	56	0	0	3	2	2	0	Proteobacteria	Gammaproteobacteria	Chromatiales	Unassigned	Unassigned	Unassigned
45	29	9	14	0	0	5	0	0	0	0	1	0	0	Proteobacteria	Gammaproteobacteria	Oceanospirillales	Oceanospirillaceae	Unassigned	Unassigned
33	72	8	3	2	3	2	7	10	16	0	0	6	15	Proteobacteria	Alphaproteobacteria	Rhizobiales	Hyphomicrobiaceae	Unassigned	Unassigned
31	75	5	2	39	26	0	0	0	0	0	0	0	3	Proteobacteria	Alphaproteobacteria	Sphingomonadales	Erythrobacteraceae	Unassigned	Unassigned
39	10	0	1	0	0	0	0	0	0	0	9	0	0	GN02	BD1-5	Unassigned	Unassigned	Unassigned	Unassigned
202	60	8	6	6	5	5	3	1	1	1	0	18	6	Proteobacteria	Alphaproteobacteria	Rhodobacterales	Rhodobacteraceae	Unassigned	Unassigned
30	74	17	22	12	11	0	0	0	0	0	2	3	7	Proteobacteria	Alphaproteobacteria	Rhizobiales	Hyphomicrobiaceae	Devosia	Unassigned
58	71	0	0	11	4	0	0	0	0	0	0	27	29	Proteobacteria	Gammaproteobacteria	Unassigned	Unassigned	Unassigned	Unassigned
418	69	27	11	4	7	0	0	3	7	1	0	3	6	Proteobacteria	Alphaproteobacteria	Rhizobiales	Phyllobacteriaceae	Unassigned	Unassigned
28	69	3	8	6	11	0	0	6	16	0	0	4	15	Proteobacteria	Gammaproteobacteria	Alteromonadales	Unassigned	Unassigned	Unassigned
41	66	0	15	0	0	17	1	0	0	7	26	0	0	Proteobacteria	Gammaproteobacteria	Chromatiales	Unassigned	Unassigned	Unassigned
1549	1	1	0	0	0	0	0	0	0	0	0	0	0	Proteobacteria	Epsilonproteobacteria	Campylobacterales	Campylobacteraceae	Arcobacter	Unassigned
54	46	5	6	0	3	9	17	0	0	2	3	1	0	Proteobacteria	Alphaproteobacteria	Rhodobacterales	Rhodobacteraceae	Phaeobacter	Unassigned
37	12	0	3	0	0	0	0	0	0	4	5	0	0	Proteobacteria	Gammaproteobacteria	Alteromonadales	Colwelliaceae	Unassigned	Unassigned
1576	44	5	1	0	0	15	9	0	0	0	1	13	0	Proteobacteria	Alphaproteobacteria	Rhodobacterales	Rhodobacteraceae	Unassigned	Unassigned
34	31	11	0	1	0	4	2	1	1	9	1	0	1	Proteobacteria	Gammaproteobacteria	Chromatiales	Chromatiaceae	Halochromatium	Unassigned
79	59	0	0	4	10	0	0	0	0	0	0	31	14	Bacteroidetes	[Saprospirae]	[Saprospirales]	Saprospiraceae	Lewinella	Unassigned
275	48	8	0	2	0	19	3	1	0	1	0	10	4	Proteobacteria	Alphaproteobacteria	Rhodobacterales	Rhodobacteraceae	Unassigned	Unassigned
520	30	9	1	0	0	18	2	0	0	0	0	0	0	Proteobacteria	Gammaproteobacteria	Chromatiales	Chromatiaceae	Unassigned	Unassigned
52	55	11	3	3	13	1	19	0	0	1	1	1	2	Proteobacteria	Betaproteobacteria	Methylophilales	Methylophilaceae	Methylotenera	mobilis
125	55	15	8	8	18	1	3	0	1	0	1	0	0	Proteobacteria	Betaproteobacteria	Burkholderiales	Comamonadaceae	Hydrogenophaga	Unassigned
40	5	2	0	0	0	0	3	0	0	0	0	0	0	Proteobacteria	Deltaproteobacteria	Spirobacillales	Unassigned	Unassigned	Unassigned
46	52	0	1	1	7	0	2	5	13	0	1	1	21	Proteobacteria	Alphaproteobacteria	Kiloniellales	Kiloniellaceae	Thalassospira	Unassigned
53	52	5	1	5	11	0	0	4	19	0	0	4	3	Proteobacteria	Alphaproteobacteria	Rhizobiales	Hyphomicrobiaceae	Devosia	Unassigned
248	51	3	4	12	7	0	2	6	6	0	1	6	4	Proteobacteria	Alphaproteobacteria	Rhizobiales	Phyllobacteriaceae	Unassigned	Unassigned
38	50	1	0	0	0	15	34	0	0	0	0	0	0	Cyanobacteria	Synechococcophycideae	Synechococcales	Acaryochloridaceae	Acaryochloris	Unassigned
160	44	7	0	9	1	15	0	1	1	0	0	1	9	Proteobacteria	Alphaproteobacteria	Kiloniellales	Kiloniellaceae	Unassigned	Unassigned
106	46	18	17	0	0	0	0	0	0	5	6	0	0	Proteobacteria	Gammaproteobacteria	Oceanospirillales	Oceanospirillaceae	Unassigned	Unassigned
47	48	1	0	0	0	1	0	0	0	22	24	0	0	Proteobacteria	Gammaproteobacteria	Unassigned	Unassigned	Unassigned	Unassigned
72	48	15	3	1	1	4	23	0	0	1	0	0	0	Proteobacteria	Alphaproteobacteria	Rhizobiales	Unassigned	Unassigned	Unassigned
91	47	0	0	0	3	0	0	0	0	0	0	7	37	Bacteroidetes	Flavobacteriia	Flavobacteriales	Flavobacteriaceae	Leeuwenhoekiella	marinoflava
183	47	2	3	1	1	6	9	9	10	3	3	0	0	Proteobacteria	Betaproteobacteria	Methylophilales	Methylophilaceae	Methylotenera	mobilis
686	33	7	9	0	0	7	0	0	0	1	9	0	0	Proteobacteria	Epsilonproteobacteria	Campylobacterales	Campylobacteraceae	Arcobacter	Unassigned
50	46	0	0	3	38	0	0	0	0	0	0	0	5	Proteobacteria	Betaproteobacteria	Rhodocyclales	Rhodocyclaceae	Unassigned	Unassigned
59	45	0	3	0	0	3	4	0	0	5	30	0	0	Proteobacteria	Deltaproteobacteria	Desulfobacterales	Desulfobulbaceae	Unassigned	Unassigned
825	37	12	1	5	2	4	4	0	0	0	0	4	5	Proteobacteria	Alphaproteobacteria	Rhodobacterales	Rhodobacteraceae	Phaeobacter	Unassigned
936	36	6	0	0	0	15	11	0	0	0	0	1	3	Proteobacteria	Alphaproteobacteria	Rhodobacterales	Rhodobacteraceae	Octadecabacter	Unassigned
49	41	2	1	0	1	7	19	3	7	0	0	1	0	Proteobacteria	Gammaproteobacteria	[Marinicellales]	[Marinicellaceae]	Marinicella	Unassigned
65	44	0	0	1	1	0	0	11	30	0	0	1	0	Proteobacteria	Gammaproteobacteria	Oceanospirillales	Halomonadaceae	Unassigned	Unassigned
68	42	14	1	2	0	13	5	4	0	0	0	3	0	Proteobacteria	Alphaproteobacteria	Rhodobacterales	Rhodobacteraceae	Amaricoccus	Unassigned
71	44	0	0	12	32	0	0	0	0	0	0	0	0	Proteobacteria	Gammaproteobacteria	Unassigned	Unassigned	Unassigned	Unassigned
640	4	0	1	0	0	1	0	0	0	0	0	2	0	Proteobacteria	Alphaproteobacteria	Rhodobacterales	Rhodobacteraceae	Octadecabacter	Unassigned
898	43	1	0	5	9	1	1	1	3	0	0	8	14	Proteobacteria	Alphaproteobacteria	Sphingomonadales	Erythrobacteraceae	Lutibacterium	Unassigned
48	41	10	1	0	0	10	19	0	0	1	0	0	0	Actinobacteria	Acidimicrobiia	Acidimicrobiales	JdFBGBact	Unassigned	Unassigned
44	40	0	0	1	1	4	1	6	26	1	0	0	0	Proteobacteria	Gammaproteobacteria	Alteromonadales	Unassigned	Unassigned	Unassigned

56	40	4	4	2	1	1	21	0	0	3	3	1	0	Proteobacteria	Deltaproteobacteria	Myxococcales	Unassigned	Unassigned	Unassigned
75	40	1	0	28	2	0	0	6	3	0	0	0	0	Firmicutes	Bacilli	Bacillales	Planococcaceae	Planococcus	Unassigned
541	38	11	8	3	0	4	3	0	0	0	2	0	7	Proteobacteria	Alphaproteobacteria	Rhodobacterales	Rhodobacteraceae	Phaeobacter	Unassigned
60	31	13	0	0	0	6	12	0	0	0	0	0	0	Chloroflexi	Anaerolineae	Ardenscatenales	Ardenscatenaceae	Ardenscatena	Unassigned
109	3	0	0	0	0	1	1	0	0	1	0	0	0	Proteobacteria	Alphaproteobacteria	Rhodobacterales	Hyphomonadaceae	Unassigned	Unassigned
155	32	4	0	9	3	0	0	4	0	0	0	10	2	Actinobacteria	Acidimicrobiia	Acidimicrobiales	C111	Unassigned	Unassigned
792	36	0	0	0	0	5	1	0	0	22	8	0	0	Proteobacteria	Epsilonproteobacteria	Campylobacterales	Campylobacteraceae	Unassigned	Unassigned
55	21	6	5	0	0	1	8	0	0	0	1	0	0	TM6	SJA-4	Unassigned	Unassigned	Unassigned	Unassigned
57	1	0	0	0	0	1	0	0	0	0	0	0	0	Proteobacteria	Deltaproteobacteria	Bdellovibrionales	Bacteriovoracaceae	Unassigned	Unassigned
62	31	14	2	0	0	7	8	0	0	0	0	0	0	Proteobacteria	Gammaproteobacteria	Chromatiales	Chromatiaceae	Halochromatium	Unassigned
114	35	0	0	10	24	0	0	0	0	0	0	1	0	Proteobacteria	Gammaproteobacteria	Chromatiales	Unassigned	Unassigned	Unassigned
300	27	4	1	12	0	1	5	0	0	0	0	4	0	Proteobacteria	Gammaproteobacteria	Alteromonadales	OM60	Congregibacter	Unassigned
533	26	0	0	0	0	23	0	0	0	1	0	0	2	Proteobacteria	Alphaproteobacteria	Sphingomonadales	Unassigned	Unassigned	Unassigned
1297	32	3	3	0	0	4	0	0	0	5	17	0	0	Proteobacteria	Gammaproteobacteria	Chromatiales	Unassigned	Unassigned	Unassigned
1401	36	8	2	7	3	2	7	1	0	2	2	2	0	Proteobacteria	Alphaproteobacteria	Rhodobacterales	Rhodobacteraceae	Anaerospora	Unassigned
104	35	0	1	0	0	0	0	0	0	6	28	0	0	Proteobacteria	Deltaproteobacteria	Desulfobacterales	Desulfobulbaceae	Unassigned	Unassigned
84	34	3	9	0	0	2	6	0	0	4	10	0	0	Proteobacteria	Betaproteobacteria	Rhodocyclales	Rhodocyclaceae	Unassigned	Unassigned
103	33	2	1	8	3	2	4	2	3	0	0	4	4	Proteobacteria	Alphaproteobacteria	Rhizobiales	Hyphomicrobiaceae	Unassigned	Unassigned
1543	27	10	8	0	0	6	2	0	0	1	0	0	0	Proteobacteria	Epsilonproteobacteria	Campylobacterales	Campylobacteraceae	Arcobacter	Unassigned
1770	4	0	0	0	0	1	1	0	0	2	0	0	0	Proteobacteria	Epsilonproteobacteria	Campylobacterales	Campylobacteraceae	Arcobacter	Unassigned
51	32	0	0	2	30	0	0	0	0	0	0	0	0	Proteobacteria	Deltaproteobacteria	Desulfuromonadales	Pelobacteraceae	Pelobacter	Unassigned
348	23	2	0	1	1	5	3	6	3	1	0	1	0	Proteobacteria	Alphaproteobacteria	Rhodobacterales	Rhodobacteraceae	Unassigned	Unassigned
66	31	0	0	0	0	0	0	0	0	0	0	4	27	Proteobacteria	Deltaproteobacteria	Myxococcales	Nannocystaceae	Plesiocystis	Unassigned
69	31	0	0	8	23	0	0	0	0	0	0	0	0	Proteobacteria	Alphaproteobacteria	Rhizobiales	Hyphomicrobiaceae	Unassigned	Unassigned
85	31	6	0	2	0	6	16	0	0	1	0	0	0	Gemmatimonadetes	Gemm-2	Unassigned	Unassigned	Unassigned	Unassigned
87	0	0	0	0	0	0	0	0	0	0	0	0	0	Cyanobacteria	Oscillatoriophycideae	Oscillatoriales	Phormidiaceae	Planktothricoides	Unassigned
154	31	1	0	13	9	0	0	2	0	0	0	2	4	Actinobacteria	Acidimicrobiia	Acidimicrobiales	Unassigned	Unassigned	Unassigned
682	31	0	0	5	23	0	0	0	0	0	0	0	3	Proteobacteria	Betaproteobacteria	Rhodocyclales	Rhodocyclaceae	Unassigned	Unassigned
1210	20	2	0	9	0	1	5	1	0	0	0	1	1	Proteobacteria	Gammaproteobacteria	Alteromonadales	OM60	Unassigned	Unassigned
1285	25	1	0	1	0	0	0	8	7	0	0	1	7	Proteobacteria	Alphaproteobacteria	Sphingomonadales	Erythrobacteraceae	Unassigned	Unassigned
63	26	2	0	0	0	13	11	0	0	0	0	0	0	Proteobacteria	Gammaproteobacteria	Chromatiales	Chromatiaceae	Unassigned	Unassigned
67	30	0	0	2	28	0	0	0	0	0	0	0	0	Proteobacteria	Gammaproteobacteria	Xanthomonadales	Xanthomonadaceae	Unassigned	Unassigned
82	25	1	0	0	2	13	4	0	0	5	0	0	0	Proteobacteria	Gammaproteobacteria	Thiotrichales	Thiotrichaceae	Thiothrix	Unassigned
108	30	0	0	1	8	0	0	0	0	0	0	6	15	Cyanobacteria	Synechococcophycideae	Pseudanabaenales	Pseudanabaenaceae	Unassigned	Unassigned
113	30	0	0	24	4	0	0	0	0	0	0	2	0	Proteobacteria	Alphaproteobacteria	Rhodospirillales	Rhodospirillaceae	Unassigned	Unassigned
136	29	5	1	0	4	9	9	0	0	1	0	0	0	Proteobacteria	Gammaproteobacteria	Chromatiales	Unassigned	Unassigned	Unassigned
176	28	5	6	1	1	2	6	0	0	1	3	1	2	Proteobacteria	Gammaproteobacteria	Chromatiales	Unassigned	Unassigned	Unassigned
112	12	2	4	0	0	5	0	0	0	1	0	0	0	Proteobacteria	Alphaproteobacteria	Rickettsiales	Unassigned	Unassigned	Unassigned
212	29	1	0	10	10	0	0	2	2	0	0	4	0	Proteobacteria	Alphaproteobacteria	Rhodobacterales	Unassigned	Unassigned	Unassigned
222	21	2	0	1	0	12	3	2	0	0	0	0	1	Proteobacteria	Alphaproteobacteria	Sphingomonadales	Erythrobacteraceae	Erythrobacter	Unassigned
1454	29	0	0	3	6	0	0	1	0	0	0	14	5	Proteobacteria	Alphaproteobacteria	Rhodobacterales	Rhodobacteraceae	Anaerospora	Unassigned
78	28	2	2	1	0	8	13	1	0	1	0	0	0	Proteobacteria	Alphaproteobacteria	Rhizobiales	Rhizobiaceae	Unassigned	Unassigned
173	28	0	0	1	11	0	0	6	3	0	0	3	4	Proteobacteria	Alphaproteobacteria	Kiloniellales	Unassigned	Unassigned	Unassigned
258	27	2	0	2	0	1	0	1	2	0	0	4	15	Proteobacteria	Alphaproteobacteria	Sphingomonadales	Erythrobacteraceae	Unassigned	Unassigned
599	4	0	0	0	0	2	1	0	0	1	0	0	0	Proteobacteria	Gammaproteobacteria	Alteromonadales	Colwelliaceae	Unassigned	Unassigned

89	26	0	2	0	0	2	2	0	0	0	0	4	16	Bacteroidetes	Flavobacteriia	Flavobacteriales	Flavobacteriaceae	Maribacter	Unassigned
111	23	2	0	7	1	8	2	1	0	1	0	0	1	Proteobacteria	Alphaproteobacteria	Rhodobacterales	Rhodobacteraceae	Paracoccus	Unassigned
115	27	5	0	0	2	6	5	1	3	0	0	0	5	Proteobacteria	Alphaproteobacteria	Rhizobiales	Hyphomicrobiaceae	Hyphomicrobium	Unassigned
175	27	3	0	5	1	3	0	0	0	0	0	8	7	Actinobacteria	Acidimicrobiia	Acidimicrobiales	C111	Unassigned	Unassigned
193	27	0	0	21	2	0	0	0	0	0	0	3	1	Proteobacteria	Gammaproteobacteria	[Marinicellales]	[Marinicellaceae]	Unassigned	Unassigned
204	27	0	0	3	5	0	0	14	2	0	0	0	3	Proteobacteria	Alphaproteobacteria	Rhodobacterales	Rhodobacteraceae	Unassigned	Unassigned
293	27	0	0	7	6	0	0	6	7	0	0	1	0	Proteobacteria	Alphaproteobacteria	Rhodobacterales	Rhodobacteraceae	Unassigned	Unassigned
681	27	3	0	5	2	8	6	1	1	0	0	1	0	Proteobacteria	Alphaproteobacteria	Rhizobiales	Hyphomicrobiaceae	Devosia	Unassigned
76	26	2	2	5	17	0	0	0	0	0	0	0	0	Proteobacteria	Gammaproteobacteria	Alteromonadales	HTCC2188	Unassigned	Unassigned
99	26	0	0	0	23	0	3	0	0	0	0	0	0	Acidobacteria	Sva0725	Sva0725	Unassigned	Unassigned	Unassigned
105	20	1	0	0	0	9	5	0	0	0	0	1	4	Proteobacteria	Alphaproteobacteria	Rhodobacterales	Rhodobacteraceae	Octadecabacter	Unassigned
132	24	0	3	0	0	7	12	0	0	0	2	0	0	Proteobacteria	Gammaproteobacteria	Alteromonadales	Ferrimonadaceae	Ferrimonas	Unassigned
157	25	0	0	14	1	0	0	0	0	0	0	7	3	Proteobacteria	Deltaproteobacteria	Myxococcales	Unassigned	Unassigned	Unassigned
165	17	2	0	0	0	8	0	0	0	2	5	0	0	Proteobacteria	Gammaproteobacteria	Alteromonadales	Colwelliaceae	Unassigned	Unassigned
971	22	8	0	1	0	1	1	1	0	0	2	5	3	Proteobacteria	Alphaproteobacteria	Rhodobacterales	Rhodobacteraceae	Dinoroseobacter	Unassigned
128	0	0	0	0	0	0	0	0	0	0	0	0	0	Bacteroidetes	Flavobacteriia	Flavobacteriales	Flavobacteriaceae	Unassigned	Unassigned
70	19	6	1	1	0	2	9	0	0	0	0	0	0	Acidobacteria	Solibacteres	Solibacterales	PAUC26f	Unassigned	Unassigned
707	23	0	0	2	3	0	0	0	0	0	0	11	7	Proteobacteria	Alphaproteobacteria	Rhodobacterales	Rhodobacteraceae	Maribius	salinus
83	22	0	0	2	1	0	0	0	0	0	0	6	13	Bacteroidetes	Cytophagia	Cytophagales	Flammeovirgaceae	Tunicatimonas	pelagia
97	21	6	1	0	0	10	2	2	0	0	0	0	0	Gemmatimonadete:	Gemmatimonadetes	Gemmatimonadales	Gemmatimonadaceae	Gemmatimonas	Unassigned
101	20	4	3	0	0	8	3	0	0	2	0	0	0	Proteobacteria	Alphaproteobacteria	Rhodobacterales	Rhodobacteraceae	Unassigned	Unassigned
139	21	1	0	0	0	0	20	0	0	0	0	0	0	Proteobacteria	Gammaproteobacteria	Alteromonadales	OM60	Unassigned	Unassigned
153	22	0	0	1	3	0	0	6	9	0	0	3	0	Bacteroidetes	Cytophagia	Cytophagales	Flammeovirgaceae	Marinoscillum	furvescens
288	18	4	0	5	3	3	0	0	0	0	0	3	0	Proteobacteria	Alphaproteobacteria	Rhodobacterales	Rhodobacteraceae	Unassigned	Unassigned
100	21	0	1	2	1	3	0	0	0	2	10	2	0	Proteobacteria	Deltaproteobacteria	Desulfobacterales	Desulfobulbaceae	Unassigned	Unassigned
120	21	0	0	0	0	0	0	0	0	0	0	15	6	Cyanobacteria	Oscillatoriophycideae	Chroococcales	Xenococcaceae	Chroococcidiopsis	Unassigned
129	21	10	4	1	0	3	3	0	0	0	0	0	0	Proteobacteria	Alphaproteobacteria	Rhodospirillales	Unassigned	Unassigned	Unassigned
130	20	14	2	0	0	1	0	0	0	0	2	0	1	Proteobacteria	Alphaproteobacteria	Rhizobiales	Hyphomicrobiaceae	Unassigned	Unassigned
141	15	7	1	0	0	5	1	0	0	1	0	0	0	Proteobacteria	Alphaproteobacteria	Rhodobacterales	Rhodobacteraceae	Anaerospora	Unassigned
889	21	0	0	0	0	0	0	10	10	0	0	0	1	Proteobacteria	Alphaproteobacteria	Sphingomonadales	Sphingomonadaceae	Novosphingobium	Unassigned
81	16	2	0	0	0	7	7	0	0	0	0	0	0	Chloroflexi	Anaerolineae	Ardenscatenales	Ardenscatenaceae	Ardenscatena	Unassigned
90	16	1	1	0	0	3	0	0	0	4	6	0	1	Proteobacteria	Deltaproteobacteria	Myxococcales	Unassigned	Unassigned	Unassigned
94	14	2	6	0	0	1	0	0	0	2	3	0	0	Proteobacteria	Gammaproteobacteria	Alteromonadales	Colwelliaceae	Unassigned	Unassigned
95	9	1	1	0	0	3	4	0	0	0	0	0	0	Proteobacteria	Gammaproteobacteria	Alteromonadales	OM60	Unassigned	Unassigned
96	20	0	0	0	0	0	0	0	0	0	0	3	17	Bacteroidetes	Cytophagia	Cytophagales	Flammeovirgaceae	Tunicatimonas	pelagia
117	20	0	0	0	0	1	19	0	0	0	0	0	0	Proteobacteria	Deltaproteobacteria	Myxococcales	Unassigned	Unassigned	Unassigned
131	11	1	0	0	0	4	4	0	0	0	1	1	0	Proteobacteria	Gammaproteobacteria	Alteromonadales	OM60	Unassigned	Unassigned
149	20	2	0	0	0	5	13	0	0	0	0	0	0	Caldithrix	Caldithrixae	Caldithrixales	BA059	Unassigned	Unassigned
383	18	1	0	0	0	5	12	0	0	0	0	0	0	Proteobacteria	Gammaproteobacteria	Alteromonadales	OM60	Congregibacter	Unassigned
1467	20	0	0	4	7	0	0	0	1	0	0	3	5	Proteobacteria	Deltaproteobacteria	Myxococcales	Unassigned	Unassigned	Unassigned
1679	20	0	0	0	0	0	0	0	0	0	0	9	11	Cyanobacteria	Synechococcophycideae	Pseudanabaenales	Pseudanabaenaceae	Unassigned	Unassigned
77	19	0	0	0	0	0	0	0	0	0	0	1	18	Proteobacteria	Deltaproteobacteria	Myxococcales	Unassigned	Unassigned	Unassigned
98	19	0	0	9	10	0	0	0	0	0	0	0	0	Proteobacteria	Deltaproteobacteria	Desulfobacterales	Desulfobulbaceae	Desulfobulbus	Unassigned
102	18	8	3	2	0	1	1	0	0	0	3	0	0	Proteobacteria	Alphaproteobacteria	Rhizobiales	Unassigned	Unassigned	Unassigned
86	18	0	0	2	5	0	0	1	9	0	0	1	0	Bacteroidetes	Flavobacteriia	Flavobacteriales	Flavobacteriaceae	Arenibacter	Unassigned

107	16	1	0	0	0	13	2	0	0	0	0	0	0	Proteobacteria	Deltaproteobacteria	Myxococcales	Nannocystaceae	Plesiocystis	Unassigned
110	16	0	0	0	0	10	6	0	0	0	0	0	0	Proteobacteria	Gammaproteobacteria	Alteromonadales	OM60	Unassigned	Unassigned
145	13	0	0	0	0	11	2	0	0	0	0	0	0	Proteobacteria	Gammaproteobacteria	[Marinicellales]	[Marinicellaceae]	Unassigned	Unassigned
1325	14	2	0	0	0	8	3	0	0	1	0	0	0	Proteobacteria	Alphaproteobacteria	Rhodobacterales	Rhodobacteraceae	Unassigned	Unassigned
119	17	0	0	8	7	1	0	0	0	0	0	1	0	Bacteroidetes	[Saprospirae]	[Saprospirales]	Saprospiraceae	Unassigned	Unassigned
121	17	10	2	0	0	3	1	0	0	1	0	0	0	Actinobacteria	Acidimicrobiia	Acidimicrobiales	TK06	Unassigned	Unassigned
182	17	2	1	0	0	4	5	0	0	4	1	0	0	Bacteroidetes	[Saprospirae]	[Saprospirales]	Saprospiraceae	Unassigned	Unassigned
264	17	0	0	15	2	0	0	0	0	0	0	0	0	Proteobacteria	Gammaproteobacteria	HOC36	Unassigned	Unassigned	Unassigned
1122	16	1	0	7	2	0	0	3	0	0	0	3	0	Proteobacteria	Alphaproteobacteria	Rhodospirillales	Unassigned	Unassigned	Unassigned
1740	17	0	0	3	7	0	0	0	0	0	0	3	4	Proteobacteria	Alphaproteobacteria	Rhodobacterales	Rhodobacteraceae	Unassigned	Unassigned
122	16	1	3	0	0	4	8	0	0	0	0	0	0	Proteobacteria	Gammaproteobacteria	[Marinicellales]	[Marinicellaceae]	Marinicella	Unassigned
126	16	1	9	0	0	0	0	0	0	0	6	0	0	Proteobacteria	Gammaproteobacteria	Chromatiales	Unassigned	Unassigned	Unassigned
138	9	1	0	0	1	4	0	0	0	0	3	0	0	Proteobacteria	Unassigned	Unassigned	Unassigned	Unassigned	Unassigned
150	15	14	1	0	0	0	0	0	0	0	0	0	0	Proteobacteria	Gammaproteobacteria	Legionellales	Unassigned	Unassigned	Unassigned
268	16	1	0	7	1	1	3	0	0	0	0	2	1	Proteobacteria	Gammaproteobacteria	Thiotrichales	Piscirickettsiaceae	Unassigned	Unassigned
273	16	0	0	0	0	0	0	0	0	4	12	0	0	Proteobacteria	Deltaproteobacteria	Desulfobacterales	Desulfobulbaceae	Desulfobulbus	mediterraneus
362	13	3	3	0	0	1	0	0	0	1	5	0	0	Proteobacteria	Gammaproteobacteria	Oceanospirillales	Oceanospirillaceae	Neptuniibacter	caesariensis
118	13	0	0	0	0	3	10	0	0	0	0	0	0	Bacteroidetes	Flavobacteriia	Flavobacteriales	Flavobacteriaceae	Unassigned	Unassigned
133	15	0	0	3	11	0	0	0	0	0	0	0	1	Proteobacteria	Alphaproteobacteria	Rhizobiales	Hyphomicrobiaceae	Unassigned	Unassigned
137	15	0	0	0	0	0	0	14	1	0	0	0	0	Proteobacteria	Alphaproteobacteria	Unassigned	Unassigned	Unassigned	Unassigned
147	0	0	0	0	0	0	0	0	0	0	0	0	0	Bacteroidetes	[Saprospirae]	[Saprospirales]	Saprospiraceae	Unassigned	Unassigned
168	15	3	0	2	2	3	5	0	0	0	0	0	0	Bacteroidetes	[Saprospirae]	[Saprospirales]	Saprospiraceae	Lewinella	nigricans
169	15	0	0	2	0	2	2	7	2	0	0	0	0	Proteobacteria	Gammaproteobacteria	Oceanospirillales	Oceanospirillaceae	Marinomonas	Unassigned
187	12	6	2	1	0	1	2	0	0	0	0	0	0	WS6	B142	Unassigned	Unassigned	Unassigned	Unassigned
246	15	0	0	1	2	0	0	0	0	0	0	11	1	Bacteroidetes	Flavobacteriia	Flavobacteriales	Flavobacteriaceae	Gramella	Unassigned
553	15	0	0	1	4	0	0	0	0	0	0	1	9	Cyanobacteria	Synechococcophycideae	Pseudanabaenales	Pseudanabaenaceae	Unassigned	Unassigned
143	14	0	0	0	0	0	0	3	11	0	0	0	0	Proteobacteria	Gammaproteobacteria	Oceanospirillales	Oceanospirillaceae	Unassigned	Unassigned
144	14	2	0	0	0	6	2	0	0	1	1	0	2	Proteobacteria	Deltaproteobacteria	Unassigned	Unassigned	Unassigned	Unassigned
156	13	1	2	0	0	3	6	0	0	0	1	0	0	Proteobacteria	Alphaproteobacteria	Kiloniellales	Unassigned	Unassigned	Unassigned
162	0	0	0	0	0	0	0	0	0	0	0	0	0	Cyanobacteria	Oscillatoriophycideae	Oscillatoriales	Phormidiaceae	Oscillatoria	Unassigned
198	14	0	0	6	5	1	2	0	0	0	0	0	0	Proteobacteria	Gammaproteobacteria	Unassigned	Unassigned	Unassigned	Unassigned
250	5	0	0	0	0	4	1	0	0	0	0	0	0	Bacteroidetes	Cytophagia	Cytophagales	Cyclobacteriaceae	Unassigned	Unassigned
256	14	3	0	0	0	4	7	0	0	0	0	0	0	Proteobacteria	Betaproteobacteria	Burkholderiales	Unassigned	Unassigned	Unassigned
140	13	0	2	0	0	2	3	0	0	2	4	0	0	Bacteroidetes	[Rhodothermi]	[Rhodothermales]	Rhodothermaceae	Unassigned	Unassigned
152	13	1	1	0	0	5	6	0	0	0	0	0	0	Proteobacteria	Gammaproteobacteria	Unassigned	Unassigned	Unassigned	Unassigned
159	13	0	0	0	2	0	0	3	8	0	0	0	0	Proteobacteria	Gammaproteobacteria	Oceanospirillales	Oceanospirillaceae	Marinomonas	Unassigned
167	7	2	1	0	0	2	2	0	0	0	0	0	0	Proteobacteria	Alphaproteobacteria	BD7-3	Unassigned	Unassigned	Unassigned
267	8	0	1	0	0	0	0	0	0	2	5	0	0	Proteobacteria	Epsilonproteobacteria	Campylobacterales	Campylobacteraceae	Sulfurospirillum	Unassigned
279	13	0	0	4	2	0	0	1	0	0	0	2	4	Actinobacteria	Acidimicrobiia	Acidimicrobiales	C111	Unassigned	Unassigned
332	13	0	0	10	3	0	0	0	0	0	0	0	0	Proteobacteria	Deltaproteobacteria	Desulfobacterales	Desulfobulbaceae	Unassigned	Unassigned
451	12	1	0	6	1	1	0	0	0	0	0	1	2	Actinobacteria	Acidimicrobiia	Acidimicrobiales	koll13	Unassigned	Unassigned
720	13	1	0	3	9	0	0	0	0	0	0	0	0	Proteobacteria	Alphaproteobacteria	Rhizobiales	Hyphomicrobiaceae	Devosia	Unassigned
885	12	0	0	0	6	0	0	0	0	0	2	0	4	Proteobacteria	Gammaproteobacteria	Unassigned	Unassigned	Unassigned	Unassigned
1013	13	0	0	0	1	0	0	0	0	0	0	11	1	Proteobacteria	Gammaproteobacteria	Oceanospirillales	Halomonadaceae	Halomonas	Unassigned
1090	13	0	0	4	1	0	0	0	0	0	0	4	4	Proteobacteria	Alphaproteobacteria	Rhodobacterales	Rhodobacteraceae	Unassigned	Unassigned

1159	10	4	0	0	0	5	0	0	0	0	0	1	0	Proteobacteria	Alphaproteobacteria	Rhodobacterales	Rhodobacteraceae	Anaerospora	Unassigned
1657	13	2	1	2	1	1	1	1	0	0	0	3	1	Proteobacteria	Alphaproteobacteria	Rhodobacterales	Rhodobacteraceae	Unassigned	Unassigned
116	12	0	0	0	0	4	7	1	0	0	0	0	0	Proteobacteria	Gammaproteobacteria	HOC36	Unassigned	Unassigned	Unassigned
127	11	1	0	0	0	2	8	0	0	0	0	0	0	Proteobacteria	Gammaproteobacteria	Thiotrichales	Piscirickettsiaceae	Unassigned	Unassigned
170	12	0	0	0	0	6	6	0	0	0	0	0	0	Cyanobacteria	Synechococcophycideae	Pseudanabaenales	Pseudanabaenaceae	Unassigned	Unassigned
171	12	1	0	0	0	6	1	0	0	1	3	0	0	Proteobacteria	Gammaproteobacteria	Chromatiales	Unassigned	Unassigned	Unassigned
174	12	0	0	2	4	0	0	0	6	0	0	0	0	Bacteroidetes	Cytophagia	Cytophagales	Flammeovirgaceae	Unassigned	Unassigned
177	7	3	0	0	0	1	0	3	0	0	0	0	0	Cyanobacteria	Synechococcophycideae	Synechococcales	Synechococcaceae	Synechococcus	Unassigned
207	6	0	0	0	0	0	0	0	0	0	0	6	0	Proteobacteria	Gammaproteobacteria	Vibrionales	Pseudoalteromonadaceae	Pseudoalteromonas	Unassigned
210	12	0	0	0	0	0	0	0	0	0	0	7	5	Bacteroidetes	[Rhodothermi]	[Rhodothermales]	Rhodothermaceae	Rubricoccus	Unassigned
211	12	2	3	1	5	0	0	0	0	0	0	1	0	Proteobacteria	Gammaproteobacteria	Oceanospirillales	Oceanospirillaceae	Unassigned	Unassigned
223	11	6	0	0	0	4	0	0	0	0	0	0	1	Proteobacteria	Alphaproteobacteria	Rhodospirillales	Unassigned	Unassigned	Unassigned
231	12	0	0	0	0	0	0	2	10	0	0	0	0	Proteobacteria	Gammaproteobacteria	Alteromonadales	Alteromonadaceae	Marinimicrobium	Unassigned
233	11	8	0	2	0	0	0	0	0	1	0	0	0	Chloroflexi	Anaerolineae	SBR1031	A4b	Unassigned	Unassigned
245	11	0	1	2	2	0	1	0	0	0	0	1	4	Bacteroidetes	Flavobacteriia	Flavobacteriales	Flavobacteriaceae	Maribacter	Unassigned
247	12	0	0	0	0	0	0	0	0	0	0	6	6	Proteobacteria	Deltaproteobacteria	Myxococcales	Nannocystaceae	Plesiocystis	Unassigned
272	12	0	0	0	3	0	0	0	0	0	0	2	7	Bacteroidetes	Cytophagia	Cytophagales	Flammeovirgaceae	Unassigned	Unassigned
281	8	1	0	0	0	1	0	0	0	3	3	0	0	Chlorobi	OPB56	Unassigned	Unassigned	Unassigned	Unassigned
286	1	1	0	0	0	0	0	0	0	0	0	0	0	Bacteroidetes	[Saprospirae]	[Saprospirales]	Saprospiraceae	Unassigned	Unassigned
299	4	1	0	0	0	2	0	0	0	0	0	1	0	Proteobacteria	Gammaproteobacteria	[Marinicellales]	[Marinicellaceae]	Unassigned	Unassigned
309	12	3	0	4	1	2	1	0	0	0	0	0	1	Proteobacteria	Alphaproteobacteria	Rhizobiales	Unassigned	Unassigned	Unassigned
399	12	2	1	2	0	2	2	0	0	0	1	2	0	Proteobacteria	Alphaproteobacteria	Rhizobiales	Hyphomicrobiaceae	Unassigned	Unassigned
1005	11	1	0	0	0	4	6	0	0	0	0	0	0	Proteobacteria	Gammaproteobacteria	Alteromonadales	OM60	Congregibacter	Unassigned
124	9	1	1	0	0	1	1	0	0	2	3	0	0	Proteobacteria	Gammaproteobacteria	Oceanospirillales	Oceanospirillaceae	Neptunomonas	Unassigned
134	11	0	0	3	1	0	0	0	0	0	0	4	3	Bacteroidetes	[Saprospirae]	[Saprospirales]	Saprospiraceae	Lewinella	Unassigned
146	11	0	0	3	8	0	0	0	0	0	0	0	0	Cyanobacteria	Oscillatoriophycideae	Chroococcales	Xenococcaceae	Unassigned	Unassigned
158	11	0	0	2	6	0	0	1	0	0	0	1	1	Actinobacteria	Acidimicrobiia	Acidimicrobiales	wb1_P06	Unassigned	Unassigned
164	11	0	0	0	1	0	0	0	6	1	2	1	0	Proteobacteria	Alphaproteobacteria	Kiloniellales	Unassigned	Unassigned	Unassigned
178	11	0	0	0	11	0	0	0	0	0	0	0	0	Acidobacteria	Sva0725	Sva0725	Unassigned	Unassigned	Unassigned
191	11	0	0	0	0	0	0	0	0	0	0	5	6	Bacteroidetes	[Saprospirae]	[Saprospirales]	Saprospiraceae	Lewinella	Unassigned
195	0	0	0	0	0	0	0	0	0	0	0	0	0	SR1	Unassigned	Unassigned	Unassigned	Unassigned	Unassigned
201	11	7	0	0	0	0	4	0	0	0	0	0	0	Proteobacteria	Alphaproteobacteria	Rhodobacterales	Hyphomonadaceae	Unassigned	Unassigned
209	4	0	0	2	0	0	0	1	0	0	0	1	0	Bacteroidetes	Cytophagia	Cytophagales	Flammeovirgaceae	Unassigned	Unassigned
224	10	2	1	0	0	5	2	0	0	0	0	0	0	Proteobacteria	Alphaproteobacteria	Rhizobiales	Hyphomicrobiaceae	Devosia	Unassigned
225	4	0	0	0	0	2	0	0	0	0	0	1	1	Proteobacteria	Deltaproteobacteria	Myxococcales	Nannocystaceae	Unassigned	Unassigned
283	11	1	1	0	1	0	0	0	0	0	0	4	4	Proteobacteria	Alphaproteobacteria	Rhizobiales	Hyphomicrobiaceae	Unassigned	Unassigned
330	11	0	2	0	0	1	0	0	0	0	8	0	0	Proteobacteria	Deltaproteobacteria	Desulfobacterales	Desulfobulbaceae	Desulfobulbus	Unassigned
363	5	2	1	0	0	0	2	0	0	0	0	0	0	Proteobacteria	Alphaproteobacteria	Rhodobacterales	Hyphomonadaceae	Hyphomonas	Unassigned
385	1	0	0	0	0	0	0	0	0	1	0	0	0	Proteobacteria	Gammaproteobacteria	Oceanospirillales	Oleiphilaceae	Unassigned	Unassigned
485	11	0	0	2	1	0	0	0	0	0	0	3	5	Proteobacteria	Alphaproteobacteria	Sphingomonadales	Sphingomonadaceae	Unassigned	Unassigned
967	11	1	0	0	9	0	0	0	0	0	0	1	0	Proteobacteria	Alphaproteobacteria	Rhodobacterales	Rhodobacteraceae	Roseivivax	Unassigned
987	11	0	0	2	2	1	2	1	1	0	1	1	0	Actinobacteria	Acidimicrobiia	Acidimicrobiales	C111	Unassigned	Unassigned
1458	11	0	0	2	9	0	0	0	0	0	0	0	0	Proteobacteria	Alphaproteobacteria	Rhodobacterales	Rhodobacteraceae	Dinoroseobacter	Unassigned
123	10	0	2	1	0	0	0	0	7	0	0	0	0	Cyanobacteria	Oscillatoriophycideae	Oscillatoriales	Phormidiaceae	Oscillatoria	acuminata
135	7	0	0	0	0	7	0	0	0	0	0	0	0	Proteobacteria	Alphaproteobacteria	Rickettsiales	Rickettsiaceae	Unassigned	Unassigned

148	10	0	0	0	0	2	8	0	0	0	0	0	0	Proteobacteria	Gammaproteobacteria	[Marinicellales]	[Marinicellaceae]	Unassigned	Unassigned
161	10	0	0	0	0	0	0	0	0	0	0	0	10	Chloroflexi	Anaerolineae	SBR1031	A4b	Unassigned	Unassigned
180	10	0	0	1	1	0	0	0	0	0	0	0	8	Proteobacteria	Gammaproteobacteria	Oceanospirillales	Saccharospirillaceae	Unassigned	Unassigned
181	10	0	0	2	6	0	0	0	0	0	0	1	1	Proteobacteria	Alphaproteobacteria	Rhizobiales	Hyphomicrobiaceae	Devosia	Unassigned
188	10	0	0	8	2	0	0	0	0	0	0	0	0	Proteobacteria	Deltaproteobacteria	Desulfobacterales	Desulfobulbaceae	Unassigned	Unassigned
190	10	0	0	7	0	0	0	3	0	0	0	0	0	Firmicutes	Bacilli	Bacillales	Unassigned	Unassigned	Unassigned
213	8	0	0	0	1	3	4	0	0	0	0	0	0	Proteobacteria	Gammaproteobacteria	Oceanospirillales	Oceanospirillaceae	Marinomonas	Unassigned
219	8	4	1	0	0	1	2	0	0	0	0	0	0	Proteobacteria	Gammaproteobacteria	Xanthomonadales	Xanthomonadaceae	Unassigned	Unassigned
227	10	0	0	4	4	0	0	1	0	0	0	0	1	Proteobacteria	Deltaproteobacteria	Desulfuromonadales	Desulfuromonadaceae	Unassigned	Unassigned
234	9	0	0	0	0	4	5	0	0	0	0	0	0	Proteobacteria	Gammaproteobacteria	Alteromonadales	OM60	Unassigned	Unassigned
253	10	0	0	1	0	1	8	0	0	0	0	0	0	Proteobacteria	Gammaproteobacteria	HTCC2188	HTCC2089	Unassigned	Unassigned
255	10	0	0	0	0	3	7	0	0	0	0	0	0	Proteobacteria	Gammaproteobacteria	Alteromonadales	OM60	Congregibacter	Unassigned
335	10	2	0	3	5	0	0	0	0	0	0	0	0	Nitrospirae	Nitrospira	Nitrospirales	Nitrospiraceae	Unassigned	Unassigned
351	10	0	0	2	1	0	2	0	1	0	0	4	0	Proteobacteria	Alphaproteobacteria	Sphingomonadales	Erythrobacteraceae	Altererythrobacter	indicus
374	9	4	1	3	0	0	0	1	0	0	0	0	0	Proteobacteria	Alphaproteobacteria	Unassigned	Unassigned	Unassigned	Unassigned
403	10	0	0	0	0	0	0	0	0	0	0	3	7	Proteobacteria	Alphaproteobacteria	Rhizobiales	Aurantimonadaceae	Fulvimarina	Unassigned
489	10	0	0	0	2	0	0	1	0	0	0	3	4	Proteobacteria	Deltaproteobacteria	Desulfuromonadales	Desulfuromonadaceae	Unassigned	Unassigned
627	10	0	0	0	1	0	0	0	0	0	0	5	4	Actinobacteria	Actinobacteria	Actinomycetales	Cellulomonadaceae	Demequina	Unassigned
1382	10	0	0	1	0	0	0	0	0	0	1	5	3	Proteobacteria	Gammaproteobacteria	Thiotrichales	Piscirickettsiaceae	Unassigned	Unassigned
1822	7	1	0	0	2	0	3	0	0	0	0	0	1	Bacteroidetes	Flavobacteriia	Flavobacteriales	Flavobacteriaceae	Psychroserpens	Unassigned
142	9	0	0	0	0	0	0	0	0	0	0	0	9	Proteobacteria	Alphaproteobacteria	Unassigned	Unassigned	Unassigned	Unassigned
186	9	6	0	0	0	1	2	0	0	0	0	0	0	Proteobacteria	Alphaproteobacteria	Rhodobacterales	Rhodobacteraceae	Unassigned	Unassigned
194	9	0	0	0	0	0	1	0	1	0	0	0	7	Bacteroidetes	[Rhodothermi]	[Rhodothermales]	Rhodothermaceae	Rubricoccus	Unassigned
196	1	1	0	0	0	0	0	0	0	0	0	0	0	Proteobacteria	Gammaproteobacteria	Aeromonadales	Unassigned	Unassigned	Unassigned
203	7	1	0	0	0	2	3	0	0	0	0	1	0	Proteobacteria	Gammaproteobacteria	Oceanospirillales	Oceanospirillaceae	Marinomonas	Unassigned
208	9	0	0	7	2	0	0	0	0	0	0	0	0	Chloroflexi	Anaerolineae	SBR1031	SJA-101	Unassigned	Unassigned
240	8	2	0	1	0	0	4	0	0	1	0	0	0	Proteobacteria	Gammaproteobacteria	Thiotrichales	Piscirickettsiaceae	Unassigned	Unassigned
243	9	1	0	0	0	0	8	0	0	0	0	0	0	Cyanobacteria	Synechococcophycideae	Pseudanabaenales	Pseudanabaenaceae	Unassigned	Unassigned
259	5	0	0	0	0	1	4	0	0	0	0	0	0	Bacteroidetes	Flavobacteriia	Flavobacteriales	Flavobacteriaceae	Polaribacter	Unassigned
260	8	0	0	1	0	5	2	0	0	0	0	0	0	Proteobacteria	Gammaproteobacteria	Alteromonadales	OM60	Unassigned	Unassigned
265	9	0	2	0	0	0	0	0	0	1	6	0	0	Proteobacteria	Deltaproteobacteria	Desulfobacterales	Desulfobulbaceae	Desulfobulbus	Unassigned
285	9	0	0	0	0	0	0	0	0	0	0	4	5	Bacteroidetes	Cytophagia	Cytophagales	Flammeovirgaceae	Fulvivirga	Unassigned
313	9	0	0	0	0	0	4	0	0	0	0	1	4	Proteobacteria	Deltaproteobacteria	Myxococcales	Unassigned	Unassigned	Unassigned
327	9	0	0	0	0	0	0	4	5	0	0	0	0	Bacteroidetes	Cytophagia	Cytophagales	Flammeovirgaceae	Unassigned	Unassigned
341	9	0	0	0	0	4	5	0	0	0	0	0	0	Proteobacteria	Alphaproteobacteria	Rhodobacterales	Rhodobacteraceae	Phaeobacter	Unassigned
369	9	0	0	0	3	0	0	0	5	0	0	0	1	Bacteroidetes	Flavobacteriia	Flavobacteriales	Flavobacteriaceae	Unassigned	Unassigned
413	9	0	0	0	0	0	4	0	0	1	4	0	0	Proteobacteria	Gammaproteobacteria	Oceanospirillales	Hahellaceae	Hahella	ganghwensis
841	2	0	0	0	0	0	0	0	0	1	1	0	0	Proteobacteria	Alphaproteobacteria	BD7-3	Unassigned	Unassigned	Unassigned
1110	9	1	0	2	4	0	0	0	0	0	0	1	1	Proteobacteria	Alphaproteobacteria	Rhodobacterales	Rhodobacteraceae	Unassigned	Unassigned
163	8	0	0	0	0	0	0	0	0	0	0	0	8	Bacteroidetes	[Rhodothermi]	[Rhodothermales]	Rhodothermaceae	Rubricoccus	Unassigned
189	5	3	1	0	0	0	1	0	0	0	0	0	0	Verrucomicrobia	Verrucomicrobiae	Verrucomicrobiales	Verrucomicrobiaceae	Rubritalea	Unassigned
214	8	1	0	0	6	0	0	0	0	0	0	1	0	Cyanobacteria	Oscillatoriophycideae	Chroococcales	Xenococcaceae	Unassigned	Unassigned
217	7	5	1	0	0	1	0	0	0	0	0	0	0	Bacteroidetes	BME43	Unassigned	Unassigned	Unassigned	Unassigned
230	8	0	0	0	0	0	0	0	0	0	0	0	8	Bacteroidetes	Cytophagia	Cytophagales	Cytophagaceae	Persicitalea	jodogahamensis
252	7	0	0	0	0	0	0	0	0	0	7	0	0	Proteobacteria	Deltaproteobacteria	Desulfuromonadales	Pelobacteraceae	Pelobacter	Unassigned

262	4	2	0	2	0	0	0	0	0	0	0	0	0	Proteobacteria	Alphaproteobacteria	Rhizobiales	Unassigned	Unassigned	Unassigned
269	7	1	0	1	0	2	2	0	0	1	0	0	0	Actinobacteria	Acidimicrobiia	Acidimicrobiales	ntu14	Unassigned	Unassigned
311	8	0	0	5	3	0	0	0	0	0	0	0	0	Proteobacteria	Alphaproteobacteria	Rhodospirillales	Rhodospirillaceae	Unassigned	Unassigned
323	6	2	0	3	0	0	0	0	0	1	0	0	0	Proteobacteria	Alphaproteobacteria	BD7-3	Unassigned	Unassigned	Unassigned
346	8	1	0	0	0	1	0	4	0	0	0	1	1	Bacteroidetes	[Saprospirae]	[Saprospirales]	Chitinophagaceae	Unassigned	Unassigned
349	8	0	0	5	0	0	0	0	0	0	0	0	3	Proteobacteria	Gammaproteobacteria	HTCC2188	HTCC2089	Unassigned	Unassigned
354	1	0	0	0	0	1	0	0	0	0	0	0	0	Proteobacteria	Alphaproteobacteria	BD7-3	Unassigned	Unassigned	Unassigned
356	7	0	0	0	0	1	3	1	1	0	0	1	0	Proteobacteria	Alphaproteobacteria	Rhodobacterales	Hyphomonadaceae	Unassigned	Unassigned
360	7	4	0	0	0	0	3	0	0	0	0	0	0	Proteobacteria	Gammaproteobacteria	Pseudomonadales	Pseudomonadaceae	Pseudomonas	pseudoalcaligenes
396	8	2	1	0	1	0	2	1	1	0	0	0	0	Proteobacteria	Gammaproteobacteria	Oceanospirillales	Saccharospirillaceae	Saccharospirillum	Unassigned
410	8	0	0	2	0	0	0	0	0	0	0	4	2	Proteobacteria	Deltaproteobacteria	Myxococcales	Unassigned	Unassigned	Unassigned
447	8	0	0	1	3	0	0	2	2	0	0	0	0	Proteobacteria	Alphaproteobacteria	Rhodobacterales	Rhodobacteraceae	Rhodobacter	Unassigned
455	4	0	0	0	0	2	2	0	0	0	0	0	0	Proteobacteria	Alphaproteobacteria	BD7-3	Unassigned	Unassigned	Unassigned
457	2	0	0	0	0	1	1	0	0	0	0	0	0	Bacteroidetes	[Saprospirae]	[Saprospirales]	Saprospiraceae	Unassigned	Unassigned
738	8	4	0	4	0	0	0	0	0	0	0	0	0	Proteobacteria	Alphaproteobacteria	Rhodobacterales	Rhodobacteraceae	Amaricoccus	Unassigned
764	7	4	0	0	0	3	0	0	0	0	0	0	0	Cyanobacteria	ML635J-21	Unassigned	Unassigned	Unassigned	Unassigned
1154	6	1	0	1	0	0	0	0	0	0	0	3	1	Proteobacteria	Alphaproteobacteria	Rhodobacterales	Hyphomonadaceae	Hyphomonas	Unassigned
1371	5	0	0	0	3	0	1	0	0	0	0	1	0	Proteobacteria	Alphaproteobacteria	Rhodobacterales	Rhodobacteraceae	Amaricoccus	Unassigned
1709	6	2	0	0	0	1	0	3	0	0	0	0	0	Proteobacteria	Alphaproteobacteria	Rhodobacterales	Rhodobacteraceae	Phaeobacter	Unassigned
1843	4	1	2	0	0	1	0	0	0	0	0	0	0	Proteobacteria	Epsilonproteobacteria	Campylobacterales	Campylobacteraceae	Arcobacter	Unassigned
172	7	0	0	0	7	0	0	0	0	0	0	0	0	Proteobacteria	Gammaproteobacteria	Oceanospirillales	Halomonadaceae	Haererehalobacter	salaria
179	7	0	0	0	0	0	0	0	7	0	0	0	0	Proteobacteria	Gammaproteobacteria	Oceanospirillales	Saccharospirillaceae	Unassigned	Unassigned
184	7	0	0	1	6	0	0	0	0	0	0	0	0	Proteobacteria	Gammaproteobacteria	Alteromonadales	HTCC2188	Unassigned	Unassigned
199	1	0	0	0	0	0	0	0	0	0	1	0	0	Proteobacteria	Deltaproteobacteria	Bdellovibrionales	Bacteriovoracaceae	Bacteriovorax	Unassigned
200	7	2	0	0	0	4	0	0	0	1	0	0	0	Caldithrix	Caldithrixae	Caldithrixales	BA059	Unassigned	Unassigned
205	7	0	0	0	0	0	5	0	0	0	2	0	0	Proteobacteria	Gammaproteobacteria	Oceanospirillales	Oceanospirillaceae	Unassigned	Unassigned
215	7	1	0	0	0	0	0	0	6	0	0	0	0	Proteobacteria	Gammaproteobacteria	Alteromonadales	Alteromonadaceae	Cellvibrio	Unassigned
218	7	3	0	0	0	2	1	0	0	1	0	0	0	Proteobacteria	Deltaproteobacteria	Myxococcales	Unassigned	Unassigned	Unassigned
229	4	0	1	0	0	2	0	0	0	0	1	0	0	Proteobacteria	Deltaproteobacteria	PB19	Unassigned	Unassigned	Unassigned
235	6	0	0	0	2	1	3	0	0	0	0	0	0	Bacteroidetes	Flavobacteriia	Flavobacteriales	Flavobacteriaceae	Unassigned	Unassigned
237	7	0	0	3	3	0	0	0	0	1	0	0	0	Bacteroidetes	[Saprospirae]	[Saprospirales]	Saprospiraceae	Unassigned	Unassigned
241	7	3	1	0	1	2	0	0	0	0	0	0	0	Proteobacteria	Alphaproteobacteria	Rhizobiales	Unassigned	Unassigned	Unassigned
242	7	1	0	0	0	4	2	0	0	0	0	0	0	Proteobacteria	Alphaproteobacteria	Rhodospirillales	Unassigned	Unassigned	Unassigned
249	7	0	0	0	0	0	0	0	0	0	0	0	7	Bacteroidetes	Bacteroidia	Bacteroidales	Unassigned	Unassigned	Unassigned
254	5	5	0	0	0	0	0	0	0	0	0	0	0	Proteobacteria	Alphaproteobacteria	Rickettsiales	Unassigned	Unassigned	Unassigned
270	6	1	0	0	1	3	0	0	0	1	0	0	0	Cyanobacteria	ML635J-21	Unassigned	Unassigned	Unassigned	Unassigned
295	5	1	0	0	0	1	3	0	0	0	0	0	0	Proteobacteria	Gammaproteobacteria	HTCC2188	HTCC2089	Unassigned	Unassigned
297	6	2	1	0	0	1	2	0	0	0	0	0	0	Proteobacteria	Alphaproteobacteria	Unassigned	Unassigned	Unassigned	Unassigned
303	7	0	0	0	4	0	0	0	0	0	0	0	3	Bacteroidetes	Cytophagia	Cytophagales	Unassigned	Unassigned	Unassigned
306	7	0	0	6	1	0	0	0	0	0	0	0	0	Bacteroidetes	Cytophagia	Cytophagales	Cytophagaceae	Unassigned	Unassigned
312	4	4	0	0	0	0	0	0	0	0	0	0	0	Proteobacteria	Gammaproteobacteria	Legionellales	Unassigned	Unassigned	Unassigned
315	7	0	0	1	6	0	0	0	0	0	0	0	0	Proteobacteria	Gammaproteobacteria	Legionellales	Unassigned	Unassigned	Unassigned
318	7	0	0	0	0	0	0	0	6	0	0	1	0	Proteobacteria	Gammaproteobacteria	Alteromonadales	Unassigned	Unassigned	Unassigned
329	7	0	0	0	0	0	0	0	0	0	1	1	5	Proteobacteria	Deltaproteobacteria	Myxococcales	Unassigned	Unassigned	Unassigned
364	5	1	0	0	0	1	2	0	0	0	1	0	0	Proteobacteria	Gammaproteobacteria	HTCC2188	HTCC2089	Acinetobacter	radioresistens

372	0	0	0	0	0	0	0	0	0	0	0	0	0	Proteobacteria	Epsilonproteobacteria	Campylobacterales	Helicobacteraceae	Unassigned	Unassigned
373	7	0	0	2	0	0	0	1	0	0	0	1	3	Proteobacteria	Alphaproteobacteria	Rhodobacterales	Rhodobacteraceae	Unassigned	Unassigned
404	7	0	0	2	5	0	0	0	0	0	0	0	0	Proteobacteria	Deltaproteobacteria	Desulfobacterales	Desulfobulbaceae	Unassigned	Unassigned
405	7	0	0	4	0	0	0	2	1	0	0	0	0	Chloroflexi	Anaerolineae	SBR1031	A4b	Unassigned	Unassigned
406	7	1	0	1	1	0	1	0	1	0	2	0	0	Proteobacteria	Gammaproteobacteria	Alteromonadales	Alteromonadaceae	ND137	Unassigned
430	7	0	0	0	0	0	0	0	0	0	0	0	7	Proteobacteria	Deltaproteobacteria	Myxococcales	Haliangiaceae	Haliangium	Unassigned
431	3	0	0	1	0	0	1	0	0	0	0	1	0	Proteobacteria	Gammaproteobacteria	Thiohalorhabdales	Unassigned	Unassigned	Unassigned
1258	7	0	0	0	0	0	0	0	0	0	0	2	5	Proteobacteria	Alphaproteobacteria	Rhizobiales	Unassigned	Unassigned	Unassigned
1317	7	0	0	0	0	0	0	0	0	0	0	3	4	Proteobacteria	Alphaproteobacteria	Rhizobiales	Phyllobacteriaceae	Unassigned	Unassigned
1323	7	0	0	2	0	0	0	0	0	0	0	1	4	Cyanobacteria	Synechococcophycideae	Pseudanabaenales	Pseudanabaenaceae	Unassigned	Unassigned
192	6	0	0	0	0	0	0	0	0	0	0	0	6	Acidobacteria	[Chloracidobacteria]	RB41	Ellin6075	Unassigned	Unassigned
197	6	0	0	0	6	0	0	0	0	0	0	0	0	Firmicutes	Clostridia	Clostridiales	Peptostreptococcaceae	Unassigned	Unassigned
206	6	0	0	0	6	0	0	0	0	0	0	0	0	Proteobacteria	Alphaproteobacteria	Rhodobacterales	Rhodobacteraceae	Unassigned	Unassigned
216	6	0	0	0	0	0	6	0	0	0	0	0	0	Bacteroidetes	[Saprospirae]	[Saprospirales]	Saprospiraceae	Unassigned	Unassigned
220	6	2	0	0	0	0	4	0	0	0	0	0	0	Bacteroidetes	[Rhodothermi]	[Rhodothermales]	Rhodothermaceae	Unassigned	Unassigned
236	6	0	0	1	3	0	0	1	0	0	0	0	1	Proteobacteria	Alphaproteobacteria	Rhodospirillales	Rhodospirillaceae	Unassigned	Unassigned
239	6	0	0	3	0	0	0	0	0	0	0	1	2	Bacteroidetes	[Saprospirae]	[Saprospirales]	Saprospiraceae	Lewinella	Unassigned
251	6	0	0	1	4	0	0	1	0	0	0	0	0	Proteobacteria	Deltaproteobacteria	Myxococcales	Unassigned	Unassigned	Unassigned
257	6	1	0	0	0	4	0	0	0	1	0	0	0	Proteobacteria	Deltaproteobacteria	Unassigned	Unassigned	Unassigned	Unassigned
261	3	0	0	0	0	3	0	0	0	0	0	0	0	Bacteroidetes	Cytophagia	Cytophagales	Flammeovirgaceae	Unassigned	Unassigned
263	6	0	0	0	6	0	0	0	0	0	0	0	0	Acidobacteria	Sva0725	Sva0725	Unassigned	Unassigned	Unassigned
296	2	0	0	0	0	2	0	0	0	0	0	0	0	Bacteroidetes	[Saprospirae]	[Saprospirales]	Saprospiraceae	Unassigned	Unassigned
298	0	0	0	0	0	0	0	0	0	0	0	0	0	Proteobacteria	Gammaproteobacteria	Legionellales	Unassigned	Unassigned	Unassigned
305	0	0	0	0	0	0	0	0	0	0	0	0	0	Proteobacteria	Alphaproteobacteria	BD7-3	Unassigned	Unassigned	Unassigned
308	6	0	0	0	0	0	0	0	6	0	0	0	0	Proteobacteria	Alphaproteobacteria	Rhodospirillales	Rhodospirillaceae	Unassigned	Unassigned
316	6	3	0	0	2	0	0	0	1	0	0	0	0	Proteobacteria	Alphaproteobacteria	Rhodospirillales	Rhodospirillaceae	Unassigned	Unassigned
336	6	0	0	5	1	0	0	0	0	0	0	0	0	Proteobacteria	Gammaproteobacteria	HOC36	Unassigned	Unassigned	Unassigned
337	6	0	0	1	3	0	0	0	2	0	0	0	0	Bacteroidetes	Cytophagia	Cytophagales	Flammeovirgaceae	Unassigned	Unassigned
344	6	0	0	0	0	0	0	0	0	0	0	1	5	Proteobacteria	Gammaproteobacteria	Alteromonadales	Unassigned	Unassigned	Unassigned
345	5	1	0	0	0	3	0	1	0	0	0	0	0	Proteobacteria	Gammaproteobacteria	HTCC2188	HTCC2089	Unassigned	Unassigned
353	5	3	0	0	0	1	0	0	0	1	0	0	0	Proteobacteria	Gammaproteobacteria	Oceanospirillales	Oleiphilaceae	Unassigned	Unassigned
361	3	0	0	0	0	1	0	1	0	0	1	0	0	Proteobacteria	Gammaproteobacteria	Vibrionales	Vibrionaceae	Vibrio	Unassigned
377	6	2	0	0	0	4	0	0	0	0	0	0	0	Proteobacteria	Gammaproteobacteria	Chromatiales	Unassigned	Unassigned	Unassigned
391	3	0	0	0	0	1	1	0	0	1	0	0	0	Proteobacteria	Gammaproteobacteria	Chromatiales	Unassigned	Unassigned	Unassigned
416	5	0	0	2	2	0	0	0	0	0	1	0	0	Proteobacteria	Deltaproteobacteria	Desulfuromonadales	Pelobacteraceae	Pelobacter	Unassigned
420	6	0	0	0	0	0	0	5	1	0	0	0	0	Bacteroidetes	Bacteroidia	Bacteroidales	Unassigned	Unassigned	Unassigned
423	6	1	0	0	0	0	0	5	0	0	0	0	0	Chloroflexi	Anaerolineae	SBR1031	A4b	Unassigned	Unassigned
424	2	0	0	0	0	1	1	0	0	0	0	0	0	Proteobacteria	Alphaproteobacteria	Unassigned	Unassigned	Unassigned	Unassigned
434	0	0	0	0	0	0	0	0	0	0	0	0	0	Proteobacteria	Gammaproteobacteria	Oceanospirillales	Saccharospirillaceae	Reinekea	Unassigned
435	6	0	0	0	0	0	0	1	5	0	0	0	0	Proteobacteria	Gammaproteobacteria	Alteromonadales	Alteromonadaceae	Unassigned	Unassigned
438	3	0	0	0	0	2	1	0	0	0	0	0	0	Proteobacteria	Gammaproteobacteria	Legionellales	Unassigned	Unassigned	Unassigned
470	0	0	0	0	0	0	0	0	0	0	0	0	0	Proteobacteria	Gammaproteobacteria	Chromatiales	Chromatiaceae	Thiorhodovibrio	winogradskyi
502	2	0	0	0	0	0	2	0	0	0	0	0	0	Proteobacteria	Alphaproteobacteria	Rhodobacterales	Hyphomonadaceae	Unassigned	Unassigned
509	6	2	0	0	4	0	0	0	0	0	0	0	0	Proteobacteria	Alphaproteobacteria	Rhodobacterales	Rhodobacteraceae	Unassigned	Unassigned
512	6	0	0	2	2	0	0	0	0	0	0	0	2	Proteobacteria	Alphaproteobacteria	Unassigned	Unassigned	Unassigned	Unassigned

573	3	0	0	0	0	2	1	0	0	0	0	0	0	Proteobacteria	Alphaproteobacteria	Unassigned	Unassigned	Unassigned	Unassigned
580	5	1	3	0	0	1	0	0	0	0	0	0	0	Bacteroidetes	[Saprospirae]	[Saprospirales]	Saprospiraceae	Unassigned	Unassigned
587	6	0	0	2	0	0	0	0	0	0	0	3	1	Bacteroidetes	[Saprospirae]	[Saprospirales]	Saprospiraceae	Unassigned	Unassigned
622	6	0	0	5	0	0	0	0	0	0	0	1	0	Actinobacteria	Acidimicrobiia	Acidimicrobiales	koll13	Unassigned	Unassigned
649	1	0	0	0	0	1	0	0	0	0	0	0	0	Bacteroidetes	[Saprospirae]	[Saprospirales]	Saprospiraceae	Unassigned	Unassigned
669	5	1	0	0	0	4	0	0	0	0	0	0	0	Proteobacteria	Gammaproteobacteria	Unassigned	Unassigned	Unassigned	Unassigned
719	0	0	0	0	0	0	0	0	0	0	0	0	0	Proteobacteria	Epsilonproteobacteria	Campylobacterales	Helicobacteraceae	Unassigned	Unassigned
749	6	0	0	1	2	0	0	2	0	0	0	1	0	Proteobacteria	Alphaproteobacteria	Kiloniellales	Unassigned	Unassigned	Unassigned
928	4	0	0	1	0	2	1	0	0	0	0	0	0	Proteobacteria	Gammaproteobacteria	Alteromonadales	OM60	Unassigned	Unassigned
1070	0	0	0	0	0	0	0	0	0	0	0	0	0	Proteobacteria	Gammaproteobacteria	Vibrionales	Pseudoalteromonadacea	Pseudoalteromonas	Unassigned
1261	6	0	0	3	0	0	0	0	0	0	0	3	0	Proteobacteria	Gammaproteobacteria	Chromatiales	Unassigned	Unassigned	Unassigned
1329	6	0	0	2	0	0	0	2	2	0	0	0	0	Bacteroidetes	Cytophagia	Cytophagales	Flammeovirgaceae	Unassigned	Unassigned
1881	6	1	0	1	0	2	0	0	0	1	0	1	0	Proteobacteria	Alphaproteobacteria	Rhodobacterales	Rhodobacteraceae	Octadecabacter	Unassigned
221	5	0	0	0	0	0	5	0	0	0	0	0	0	Proteobacteria	Alphaproteobacteria	Rickettsiales	Rickettsiaceae	Unassigned	Unassigned
228	2	0	0	0	0	2	0	0	0	0	0	0	0	Bacteroidetes	[Saprospirae]	[Saprospirales]	Saprospiraceae	Unassigned	Unassigned
232	5	0	0	0	0	1	2	0	0	2	0	0	0	[Thermi]	Deinococci	Deinococcales	Trueperaceae	Unassigned	Unassigned
238	5	0	0	0	5	0	0	0	0	0	0	0	0	Proteobacteria	Gammaproteobacteria	Alteromonadales	Unassigned	Unassigned	Unassigned
244	5	0	0	0	0	0	0	0	0	0	0	0	5	Proteobacteria	Deltaproteobacteria	Myxococcales	Unassigned	Unassigned	Unassigned
266	5	0	0	4	0	0	0	0	0	0	0	1	0	Proteobacteria	Gammaproteobacteria	[Marinicellales]	[Marinicellaceae]	Unassigned	Unassigned
271	1	0	0	0	1	0	0	0	0	0	0	0	0	Bacteroidetes	Sphingobacteriia	Sphingobacteriales	NS11-12	Unassigned	Unassigned
274	0	0	0	0	0	0	0	0	0	0	0	0	0	Proteobacteria	Gammaproteobacteria	Vibrionales	Pseudoalteromonadacea	Pseudoalteromonas	Unassigned
276	5	5	0	0	0	0	0	0	0	0	0	0	0	Proteobacteria	Deltaproteobacteria	Myxococcales	Nannocystaceae	Plesiocystis	Unassigned
277	2	0	0	0	0	0	2	0	0	0	0	0	0	Chlorobi	OPB56	Unassigned	Unassigned	Unassigned	Unassigned
282	5	0	0	3	0	0	0	0	0	0	1	1	0	Proteobacteria	Deltaproteobacteria	Desulfobacterales	Desulfobulbaceae	Unassigned	Unassigned
290	5	4	0	1	0	0	0	0	0	0	0	0	0	Proteobacteria	Alphaproteobacteria	BD7-3	Unassigned	Unassigned	Unassigned
294	5	4	0	0	0	1	0	0	0	0	0	0	0	Proteobacteria	Alphaproteobacteria	Unassigned	Unassigned	Unassigned	Unassigned
301	5	0	0	0	0	0	0	0	0	0	0	4	1	Acidobacteria	Sva0725	Sva0725	Unassigned	Unassigned	Unassigned
307	5	1	0	0	0	0	0	0	0	0	0	1	3	Proteobacteria	Alphaproteobacteria	Rhodospirillales	Rhodospirillaceae	Unassigned	Unassigned
320	5	0	0	0	0	1	0	0	0	0	0	0	4	Bacteroidetes	Flavobacteriia	Flavobacteriales	Unassigned	Unassigned	Unassigned
321	5	0	0	0	0	0	0	0	0	0	0	2	3	Bacteroidetes	Cytophagia	Cytophagales	Flammeovirgaceae	Tunicatimonas	pelagia
322	0	0	0	0	0	0	0	0	0	0	0	0	0	Proteobacteria	Gammaproteobacteria	Legionellales	Francisellaceae	Francisella	Unassigned
324	5	0	0	1	4	0	0	0	0	0	0	0	0	Proteobacteria	Gammaproteobacteria	Alteromonadales	Alteromonadaceae	Unassigned	Unassigned
326	5	0	0	0	4	0	0	0	0	0	0	1	0	Proteobacteria	Deltaproteobacteria	Syntrophobacterales	Syntrophobacteraceae	Unassigned	Unassigned
331	0	0	0	0	0	0	0	0	0	0	0	0	0	Bacteroidetes	Flavobacteriia	Flavobacteriales	Flavobacteriaceae	Unassigned	Unassigned
333	1	0	0	0	0	1	0	0	0	0	0	0	0	Bacteroidetes	Cytophagia	Cytophagales	Cyclobacteriaceae	Unassigned	Unassigned
342	0	0	0	0	0	0	0	0	0	0	0	0	0	Bacteroidetes	[Saprospirae]	[Saprospirales]	Unassigned	Unassigned	Unassigned
347	5	0	0	0	0	0	0	4	1	0	0	0	0	Proteobacteria	Alphaproteobacteria	Rhodobacterales	Rhodobacteraceae	Rhodovulum	Unassigned
366	5	0	0	0	0	0	0	0	5	0	0	0	0	Proteobacteria	Gammaproteobacteria	Alteromonadales	Alteromonadaceae	Marinobacter	Unassigned
375	5	0	0	0	0	0	0	0	0	0	0	4	1	Proteobacteria	Gammaproteobacteria	Alteromonadales	Alteromonadaceae	Glaciecola	Unassigned
378	5	0	0	0	5	0	0	0	0	0	0	0	0	Acidobacteria	Sva0725	Sva0725	Unassigned	Unassigned	Unassigned
387	5	0	0	2	2	0	0	1	0	0	0	0	0	Gemmatimonadete	Gemm-2	Unassigned	Unassigned	Unassigned	Unassigned
407	5	0	0	3	2	0	0	0	0	0	0	0	0	Bacteroidetes	Cytophagia	Cytophagales	Flammeovirgaceae	Flexithrix	Unassigned
415	5	5	0	0	0	0	0	0	0	0	0	0	0	Verrucomicrobia	Verrucomicrobiae	Verrucomicrobiales	Verrucomicrobiaceae	Persicirhabdus	Unassigned
417	1	0	0	0	0	1	0	0	0	0	0	0	0	Bacteroidetes	Flavobacteriia	Flavobacteriales	Unassigned	Unassigned	Unassigned
421	1	1	0	0	0	0	0	0	0	0	0	0	0	Proteobacteria	Alphaproteobacteria	BD7-3	Unassigned	Unassigned	Unassigned

422	5	2	0	1	1	0	0	1	0	0	0	0	0	Proteobacteria	Alphaproteobacteria	Unassigned	Unassigned	Unassigned	Unassigned
441	0	0	0	0	0	0	0	0	0	0	0	0	0	Proteobacteria	Alphaproteobacteria	Rickettsiales	Unassigned	Unassigned	Unassigned
442	5	0	0	5	0	0	0	0	0	0	0	0	0	Bacteroidetes	[Saprospirae]	[Saprospirales]	Saprospiraceae	Unassigned	Unassigned
445	5	0	0	0	0	0	0	0	0	0	0	0	5	Bacteroidetes	Cytophagia	Cytophagales	Flammeovirgaceae	Unassigned	Unassigned
452	2	0	0	0	0	1	1	0	0	0	0	0	0	Proteobacteria	Gammaproteobacteria	Alteromonadales	Ferrimonadaceae	Ferrimonas	Unassigned
467	0	0	0	0	0	0	0	0	0	0	0	0	0	Bacteroidetes	[Saprospirae]	[Saprospirales]	Saprospiraceae	Unassigned	Unassigned
490	5	0	0	0	0	2	2	0	0	1	0	0	0	Gemmatimonadetes	Gemmatimonadetes	KD8-87	Unassigned	Unassigned	Unassigned
531	5	0	0	1	2	1	1	0	0	0	0	0	0	Proteobacteria	Alphaproteobacteria	Rhodospirillales	Rhodospirillaceae	Inquilinus	Unassigned
534	5	0	0	2	2	0	0	0	0	0	0	1	0	Proteobacteria	Alphaproteobacteria	Rhizobiales	Hyphomicrobiaceae	Rhodoplanes	Unassigned
545	5	0	0	2	3	0	0	0	0	0	0	0	0	Proteobacteria	Alphaproteobacteria	Rhodospirillales	Rhodospirillaceae	Unassigned	Unassigned
563	1	0	0	0	0	1	0	0	0	0	0	0	0	Proteobacteria	Gammaproteobacteria	Unassigned	Unassigned	Unassigned	Unassigned
568	5	0	0	0	0	0	0	0	0	0	0	2	3	Bacteroidetes	[Saprospirae]	[Saprospirales]	Saprospiraceae	Lewinella	Unassigned
572	5	0	0	0	2	0	1	1	0	0	0	0	1	Proteobacteria	Alphaproteobacteria	Rhodospirillales	Rhodospirillaceae	Unassigned	Unassigned
577	5	1	0	2	0	0	0	0	0	0	2	0	0	Chloroflexi	Anaerolineae	SBR1031	A4b	Unassigned	Unassigned
579	5	0	0	0	5	0	0	0	0	0	0	0	0	Proteobacteria	Alphaproteobacteria	Rhodospirillales	Rhodospirillaceae	Unassigned	Unassigned
582	5	0	0	0	2	0	0	0	3	0	0	0	0	Bacteroidetes	Cytophagia	Cytophagales	Flammeovirgaceae	Marinoscillum	furvescens
590	4	0	0	0	0	0	0	0	0	0	4	0	0	Proteobacteria	Deltaproteobacteria	Desulfobacterales	Desulfobulbaceae	Desulfotalea	Unassigned
637	4	1	3	0	0	0	0	0	0	0	0	0	0	Proteobacteria	Epsilonproteobacteria	Campylobacterales	Campylobacteraceae	Arcobacter	Unassigned
639	5	0	0	0	0	0	0	3	2	0	0	0	0	Bacteroidetes	Cytophagia	Cytophagales	Flammeovirgaceae	Unassigned	Unassigned
642	3	1	0	1	0	0	0	1	0	0	0	0	0	GN02	BB34	Unassigned	Unassigned	Unassigned	Unassigned
675	3	0	0	0	0	3	0	0	0	0	0	0	0	Proteobacteria	Alphaproteobacteria	BD7-3	Unassigned	Unassigned	Unassigned
721	5	0	0	1	2	1	1	0	0	0	0	0	0	Proteobacteria	Alphaproteobacteria	BD7-3	Unassigned	Unassigned	Unassigned
740	5	0	0	2	1	1	0	0	0	0	0	1	0	Caldithrix	Caldithrixae	Caldithrixales	Caldithrixaceae	Unassigned	Unassigned
888	5	0	0	0	0	0	0	0	0	0	0	0	5	Proteobacteria	Alphaproteobacteria	Sphingomonadales	Erythrobacteraceae	Unassigned	Unassigned
1065	1	0	0	0	0	0	0	0	0	1	0	0	0	Proteobacteria	Gammaproteobacteria	Vibrionales	Vibrionaceae	Unassigned	Unassigned
1092	0	0	0	0	0	0	0	0	0	0	0	0	0	Bacteroidetes	Flavobacteriia	Flavobacteriales	Cryomorphaceae	Cryomorpha	Unassigned
1096	5	0	0	0	0	0	0	0	0	0	0	2	3	Bacteroidetes	[Saprospirae]	[Saprospirales]	Saprospiraceae	Lewinella	Unassigned
1547	5	0	0	0	0	2	0	0	0	0	2	1	0	Chlorobi	Ignavibacteria	Ignavibacteriales	lheB3-7	Unassigned	Unassigned
1599	4	0	0	0	0	0	0	0	0	0	4	0	0	Proteobacteria	Gammaproteobacteria	Alteromonadales	Colwelliaceae	Unassigned	Unassigned
1610	4	0	0	0	0	0	0	0	4	0	0	0	0	Proteobacteria	Gammaproteobacteria	Alteromonadales	Unassigned	Unassigned	Unassigned
1729	3	0	0	0	0	1	1	0	0	0	0	0	1	Proteobacteria	Alphaproteobacteria	Rhizobiales	Phyllobacteriaceae	Unassigned	Unassigned
278	4	0	0	0	0	0	0	0	0	0	0	0	4	Proteobacteria	Deltaproteobacteria	Myxococcales	Unassigned	Unassigned	Unassigned
280	4	0	0	0	0	0	0	0	0	0	0	0	4	Proteobacteria	Alphaproteobacteria	Rhodospirillales	Rhodospirillaceae	magnetite-containing	magneticvibrio
284	0	0	0	0	0	0	0	0	0	0	0	0	0	Proteobacteria	Gammaproteobacteria	Legionellales	Unassigned	Unassigned	Unassigned
287	4	0	0	0	0	3	1	0	0	0	0	0	0	Proteobacteria	Gammaproteobacteria	Chromatiales	Unassigned	Unassigned	Unassigned
291	4	0	0	0	0	0	0	0	4	0	0	0	0	Proteobacteria	Gammaproteobacteria	Legionellales	Coxiellaceae	Coxiella	Unassigned
292	0	0	0	0	0	0	0	0	0	0	0	0	0	Proteobacteria	Alphaproteobacteria	Rickettsiales	Unassigned	Unassigned	Unassigned
302	0	0	0	0	0	0	0	0	0	0	0	0	0	Proteobacteria	Alphaproteobacteria	Unassigned	Unassigned	Unassigned	Unassigned
304	4	0	0	0	4	0	0	0	0	0	0	0	0	Acidobacteria	Sva0725	Sva0725	Unassigned	Unassigned	Unassigned
310	4	0	0	0	4	0	0	0	0	0	0	0	0	Proteobacteria	Betaproteobacteria	Unassigned	Unassigned	Unassigned	Unassigned
314	4	0	0	0	0	0	0	0	0	0	0	2	2	Proteobacteria	Gammaproteobacteria	Thiotrichales	Piscirickettsiaceae	Methylophaga	Unassigned
317	4	0	0	1	3	0	0	0	0	0	0	0	0	Proteobacteria	Alphaproteobacteria	Caulobacterales	Caulobacteraceae	Unassigned	Unassigned
319	3	0	0	0	0	0	0	0	0	2	1	0	0	Spirochaetes	MVP-15	PL-11B10	Unassigned	Unassigned	Unassigned
325	4	0	0	0	0	0	0	3	1	0	0	0	0	Proteobacteria	Deltaproteobacteria	Myxococcales	Unassigned	Unassigned	Unassigned
328	4	0	0	0	0	0	0	0	0	0	0	2	2	Proteobacteria	Gammaproteobacteria	Alteromonadales	Alteromonadaceae	Unassigned	Unassigned

334	4	0	0	0	1	0	0	0	0	0	0	0	3	Cyanobacteria	Oscillatoriophycideae	Chroococcales	Xenococcaceae	Unassigned	Unassigned
339	0	0	0	0	0	0	0	0	0	0	0	0	0	Bacteroidetes	Flavobacteriia	Flavobacteriales	Unassigned	Unassigned	Unassigned
352	4	0	0	0	0	1	3	0	0	0	0	0	0	Proteobacteria	Alphaproteobacteria	Rhodospirillales	Rhodospirillaceae	Unassigned	Unassigned
355	4	0	0	1	3	0	0	0	0	0	0	0	0	Proteobacteria	Alphaproteobacteria	Rhizobiales	Rhizobiaceae	Agrobacterium	Unassigned
365	4	0	0	0	0	1	3	0	0	0	0	0	0	Bacteroidetes	Flavobacteriia	Flavobacteriales	Flavobacteriaceae	Coccinimonas	marina
367	4	0	0	0	0	0	0	1	3	0	0	0	0	Bacteroidetes	Cytophagia	Cytophagales	Flammeovirgaceae	Marivirga	tractuosa
370	0	0	0	0	0	0	0	0	0	0	0	0	0	GN02	GN07	Unassigned	Unassigned	Unassigned	Unassigned
376	4	0	0	0	0	0	0	0	0	0	0	3	1	Actinobacteria	Acidimicrobiia	Acidimicrobiales	koll13	Unassigned	Unassigned
380	4	4	0	0	0	0	0	0	0	0	0	0	0	Bacteroidetes	Flavobacteriia	Flavobacteriales	Cryomorphaceae	Unassigned	Unassigned
381	2	0	1	0	0	1	0	0	0	0	0	0	0	TM6	SJA-4	Unassigned	Unassigned	Unassigned	Unassigned
382	3	0	0	0	0	2	0	0	0	0	0	1	0	Proteobacteria	Alphaproteobacteria	BD7-3	Unassigned	Unassigned	Unassigned
388	2	0	0	0	0	2	0	0	0	0	0	0	0	Proteobacteria	Deltaproteobacteria	Myxococcales	Unassigned	Unassigned	Unassigned
395	4	0	0	4	0	0	0	0	0	0	0	0	0	GN02	3BR-5F	Unassigned	Unassigned	Unassigned	Unassigned
401	4	0	0	3	1	0	0	0	0	0	0	0	0	Acidobacteria	[Chloracidobacteria]	RB41	Ellin6075	Unassigned	Unassigned
408	3	0	0	0	0	2	0	0	0	0	1	0	0	Proteobacteria	Alphaproteobacteria	Rhizobiales	Phyllobacteriaceae	Unassigned	Unassigned
409	4	0	1	1	1	0	0	0	1	0	0	0	0	Proteobacteria	Betaproteobacteria	Rhodocyclales	Rhodocyclaceae	Azoarcus	Unassigned
412	4	0	0	0	1	0	0	0	0	0	0	1	2	Bacteroidetes	Flavobacteriia	Flavobacteriales	Flavobacteriaceae	Pseudozobellia	thermophila
414	4	2	0	0	0	2	0	0	0	0	0	0	0	Bacteroidetes	[Saprospirae]	[Saprospirales]	Saprospiraceae	Unassigned	Unassigned
425	2	2	0	0	0	0	0	0	0	0	0	0	0	Proteobacteria	Alphaproteobacteria	Unassigned	Unassigned	Unassigned	Unassigned
428	4	0	0	0	1	0	0	0	0	0	0	0	3	Bacteroidetes	Bacteroidia	Bacteroidales	Unassigned	Unassigned	Unassigned
429	4	0	0	0	3	0	0	0	0	0	0	0	1	Bacteroidetes	Flavobacteriia	Flavobacteriales	Flavobacteriaceae	Unassigned	Unassigned
436	0	0	0	0	0	0	0	0	0	0	0	0	0	Proteobacteria	Gammaproteobacteria	Thiotrichales	Thiotrichaceae	Unassigned	Unassigned
440	0	0	0	0	0	0	0	0	0	0	0	0	0	Proteobacteria	Gammaproteobacteria	Oceanospirillales	Oceanospirillaceae	Unassigned	Unassigned
443	4	0	0	0	0	0	0	0	0	0	0	2	2	Proteobacteria	Gammaproteobacteria	Alteromonadales	Unassigned	Unassigned	Unassigned
444	2	0	0	1	1	0	0	0	0	0	0	0	0	Proteobacteria	Gammaproteobacteria	Thiohalorhabdales	Unassigned	Unassigned	Unassigned
459	4	3	0	0	0	0	0	0	0	0	0	1	0	Proteobacteria	Alphaproteobacteria	Rhizobiales	Unassigned	Unassigned	Unassigned
461	4	0	0	2	0	0	0	1	1	0	0	0	0	Chloroflexi	Anaerolineae	SBR1031	A4b	Unassigned	Unassigned
462	4	0	0	0	0	1	0	0	0	0	1	2	0	Bacteroidetes	Bacteroidia	Bacteroidales	SB-1	Unassigned	Unassigned
465	4	0	0	2	0	0	0	2	0	0	0	0	0	Actinobacteria	Acidimicrobiia	Acidimicrobiales	koll13	Unassigned	Unassigned
469	4	0	0	0	0	0	0	0	0	0	0	2	2	Actinobacteria	Actinobacteria	Actinomycetales	Nocardioidaceae	Unassigned	Unassigned
472	4	0	0	0	0	0	4	0	0	0	0	0	0	Proteobacteria	Deltaproteobacteria	Myxococcales	Cystobacterineae	Unassigned	Unassigned
480	3	0	0	0	0	0	2	0	0	0	0	1	0	Proteobacteria	Alphaproteobacteria	Rickettsiales	Rickettsiaceae	Unassigned	Unassigned
483	4	1	0	0	1	1	0	0	0	1	0	0	0	Actinobacteria	Acidimicrobiia	Acidimicrobiales	Microthrixaceae	Unassigned	Unassigned
491	3	0	0	0	0	2	1	0	0	0	0	0	0	Bacteroidetes	[Saprospirae]	[Saprospirales]	Saprospiraceae	Lewinella	Unassigned
494	4	0	0	2	2	0	0	0	0	0	0	0	0	Proteobacteria	Deltaproteobacteria	NB1-j	Unassigned	Unassigned	Unassigned
503	0	0	0	0	0	0	0	0	0	0	0	0	0	Acidobacteria	Holophagae	Holophagales	Unassigned	Unassigned	Unassigned
515	4	0	0	0	0	3	1	0	0	0	0	0	0	Proteobacteria	Alphaproteobacteria	Unassigned	Unassigned	Unassigned	Unassigned
516	4	0	0	4	0	0	0	0	0	0	0	0	0	Actinobacteria	Nitriliruptoria	Euzebyales	Euzebyaceae	Euzebya	Unassigned
517	0	0	0	0	0	0	0	0	0	0	0	0	0	Bacteroidetes	[Saprospirae]	[Saprospirales]	Saprospiraceae	Lewinella	Unassigned
524	4	1	0	1	1	0	0	0	1	0	0	0	0	Bacteroidetes	Cytophagia	Cytophagales	Cytophagaceae	Unassigned	Unassigned
532	2	0	0	0	0	0	0	0	0	1	1	0	0	Proteobacteria	Deltaproteobacteria	Bdellovibrionales	Bacteriovoracaceae	Unassigned	Unassigned
536	4	0	0	0	0	1	0	3	0	0	0	0	0	Proteobacteria	Alphaproteobacteria	Sphingomonadales	Sphingomonadaceae	Kaistobacter	Unassigned
543	4	0	0	0	0	0	4	0	0	0	0	0	0	Proteobacteria	Gammaproteobacteria	Legionellales	Coxiellaceae	Coxiella	Unassigned
560	4	0	0	1	2	1	0	0	0	0	0	0	0	SBR1093	VHS-B5-50	Unassigned	Unassigned	Unassigned	Unassigned
574	4	2	0	0	0	0	0	1	1	0	0	0	0	Proteobacteria	Alphaproteobacteria	Kordiimonadales	Kordiimonadaceae	Unassigned	Unassigned

575	4	0	0	3	0	0	0	0	1	0	0	0	0	Bacteroidetes	Cytophagia	Cytophagales	Cytophagaceae	Unassigned	Unassigned
581	2	0	0	0	0	2	0	0	0	0	0	0	0	Proteobacteria	TA18	CV90	Unassigned	Unassigned	Unassigned
586	4	1	0	0	0	1	0	1	1	0	0	0	0	Proteobacteria	Alphaproteobacteria	BD7-3	Unassigned	Unassigned	Unassigned
603	4	0	0	3	0	0	0	0	0	0	0	1	0	Proteobacteria	Gammaproteobacteria	Alteromonadales	OM60	Unassigned	Unassigned
612	4	0	0	2	0	1	1	0	0	0	0	0	0	Bacteroidetes	[Saprospirae]	[Saprospirales]	Saprospiraceae	Unassigned	Unassigned
617	2	0	0	0	0	0	2	0	0	0	0	0	0	Bacteroidetes	[Saprospirae]	[Saprospirales]	Saprospiraceae	Unassigned	Unassigned
619	3	0	0	0	0	0	3	0	0	0	0	0	0	Bacteroidetes	Cytophagia	Cytophagales	Flammeovirgaceae	Roseivirga	Unassigned
629	0	0	0	0	0	0	0	0	0	0	0	0	0	Proteobacteria	Gammaproteobacteria	Thiotrichales	Piscirickettsiaceae	Unassigned	Unassigned
643	0	0	0	0	0	0	0	0	0	0	0	0	0	Bacteroidetes	Flavobacteriia	Flavobacteriales	Cryomorphaceae	Unassigned	Unassigned
651	4	0	0	0	0	0	0	3	1	0	0	0	0	Chlorobi	Ignavibacteria	Ignavibacteriales	lheB3-7	Unassigned	Unassigned
653	4	0	0	1	0	0	0	2	0	0	0	1	0	Bacteroidetes	[Rhodothermi]	[Rhodothermales]	Rhodothermaceae	Rubricoccus	Unassigned
661	4	0	0	0	0	0	0	0	0	1	3	0	0	Proteobacteria	Deltaproteobacteria	Desulfuromonadales	Pelobacteraceae	Pelobacter	Unassigned
701	2	0	0	0	0	2	0	0	0	0	0	0	0	Proteobacteria	Gammaproteobacteria	[Marinicellales]	[Marinicellaceae]	Marinicella	Unassigned
752	4	0	0	2	2	0	0	0	0	0	0	0	0	Proteobacteria	Alphaproteobacteria	Rhodospirillales	Rhodospirillaceae	Inquilinus	Unassigned
756	4	0	0	0	0	0	0	2	2	0	0	0	0	Proteobacteria	Alphaproteobacteria	BD7-3	Unassigned	Unassigned	Unassigned
761	4	0	0	0	0	0	0	0	3	0	0	0	1	Bacteroidetes	Flavobacteriia	Flavobacteriales	Flavobacteriaceae	Ulvibacter	Unassigned
775	4	0	0	1	1	0	0	0	0	0	0	2	0	Bacteroidetes	[Saprospirae]	[Saprospirales]	Saprospiraceae	Unassigned	Unassigned
783	4	4	0	0	0	0	0	0	0	0	0	0	0	Bacteroidetes	Bacteroidia	Bacteroidales	Unassigned	Unassigned	Unassigned
808	4	0	0	1	0	0	0	2	0	0	0	1	0	Actinobacteria	Actinobacteria	Actinomycetales	Cellulomonadaceae	Demequina	Unassigned
839	1	0	0	0	0	0	0	0	0	1	0	0	0	Proteobacteria	Gammaproteobacteria	Alteromonadales	Alteromonadaceae	nsmpVI18	Unassigned
859	4	0	0	0	1	0	0	3	0	0	0	0	0	Bacteroidetes	Flavobacteriia	Flavobacteriales	Flavobacteriaceae	Unassigned	Unassigned
860	4	0	0	0	0	0	0	4	0	0	0	0	0	Gemmatimonadetes	Gemm-2	Unassigned	Unassigned	Unassigned	Unassigned
881	4	0	0	2	0	0	0	0	1	0	0	0	1	Proteobacteria	Gammaproteobacteria	Chromatiales	Unassigned	Unassigned	Unassigned
955	0	0	0	0	0	0	0	0	0	0	0	0	0	Bacteroidetes	Flavobacteriia	Flavobacteriales	Flavobacteriaceae	Unassigned	Unassigned
1023	4	0	0	0	0	0	0	3	1	0	0	0	0	Proteobacteria	Alphaproteobacteria	Unassigned	Unassigned	Unassigned	Unassigned
1045	3	2	0	0	0	0	0	0	0	0	1	0	0	Proteobacteria	Alphaproteobacteria	Rickettsiales	Unassigned	Unassigned	Unassigned
1056	4	0	0	4	0	0	0	0	0	0	0	0	0	Actinobacteria	Acidimicrobiia	Acidimicrobiales	Unassigned	Unassigned	Unassigned
1149	3	0	0	0	0	0	0	1	2	0	0	0	0	Proteobacteria	Gammaproteobacteria	Alteromonadales	Alteromonadaceae	Marinobacter	Unassigned
1160	4	0	0	0	0	2	0	0	0	1	1	0	0	Proteobacteria	Gammaproteobacteria	Unassigned	Unassigned	Unassigned	Unassigned
1166	4	2	1	0	1	0	0	0	0	0	0	0	0	Bacteroidetes	[Saprospirae]	[Saprospirales]	Chitinophagaceae	Unassigned	Unassigned
1237	4	0	0	0	0	0	0	3	1	0	0	0	0	Bacteroidetes	[Rhodothermi]	[Rhodothermales]	[Balneolaceae]	KSA1	Unassigned
1239	4	0	0	1	1	0	0	0	0	0	1	0	1	Proteobacteria	Gammaproteobacteria	Alteromonadales	Unassigned	Unassigned	Unassigned
1326	4	1	0	0	0	1	2	0	0	0	0	0	0	Proteobacteria	Alphaproteobacteria	Rhodobacterales	Rhodobacteraceae	Paracoccus	Unassigned
1356	2	1	0	0	0	0	0	0	0	0	1	0	0	Proteobacteria	Alphaproteobacteria	BD7-3	Unassigned	Unassigned	Unassigned
1390	4	0	0	3	0	0	0	1	0	0	0	0	0	Gemmatimonadetes	Gemm-2	Unassigned	Unassigned	Unassigned	Unassigned
1403	4	0	0	3	0	0	0	0	0	0	0	0	1	Proteobacteria	Alphaproteobacteria	Rhodospirillales	Unassigned	Unassigned	Unassigned
1404	4	0	0	4	0	0	0	0	0	0	0	0	0	Proteobacteria	Deltaproteobacteria	Desulfobacterales	Desulfobulbaceae	Unassigned	Unassigned
1429	4	0	0	2	1	0	0	0	0	0	0	0	1	Proteobacteria	Alphaproteobacteria	Caulobacterales	Caulobacteraceae	Unassigned	Unassigned
1631	1	0	0	0	0	1	0	0	0	0	0	0	0	Proteobacteria	Alphaproteobacteria	BD7-3	Unassigned	Unassigned	Unassigned
1742	4	0	0	1	1	0	0	0	0	0	0	1	1	Proteobacteria	Gammaproteobacteria	Thiotrichales	Piscirickettsiaceae	Unassigned	Unassigned
1897	4	0	0	2	2	0	0	0	0	0	0	0	0	Proteobacteria	Alphaproteobacteria	Rhizobiales	Hyphomicrobiaceae	Devosia	Unassigned
338	3	0	0	0	0	0	0	3	0	0	0	0	0	Actinobacteria	Acidimicrobiia	Acidimicrobiales	Unassigned	Unassigned	Unassigned
340	3	0	0	0	0	0	0	0	0	0	0	0	3	Proteobacteria	Alphaproteobacteria	Rhodospirillales	Rhodospirillaceae	magnetite-containing	magneticvibrio
343	0	0	0	0	0	0	0	0	0	0	0	0	0	Bacteroidetes	[Saprospirae]	[Saprospirales]	Saprospiraceae	Unassigned	Unassigned
350	3	0	0	0	1	0	0	0	0	0	0	0	2	Proteobacteria	Gammaproteobacteria	Alteromonadales	Alteromonadaceae	Unassigned	Unassigned

357	3	0	0	0	3	0	0	0	0	0	0	0	0	Proteobacteria	Deltaproteobacteria	Spirobacillales	Unassigned	Unassigned	Unassigned
359	3	0	0	1	0	0	0	0	0	0	0	0	2	Actinobacteria	Acidimicrobiia	Acidimicrobiales	Unassigned	Unassigned	Unassigned
368	3	0	0	0	3	0	0	0	0	0	0	0	0	Bacteroidetes	[Saprospirae]	[Saprospirales]	Saprospiraceae	Portibacter	lacus
371	3	0	0	0	0	0	0	0	0	0	0	1	2	Bacteroidetes	[Saprospirae]	[Saprospirales]	Saprospiraceae	Unassigned	Unassigned
379	3	2	1	0	0	0	0	0	0	0	0	0	0	Proteobacteria	Gammaproteobacteria	Legionellales	Coxiellaceae	Coxiella	Unassigned
384	3	0	0	0	0	0	3	0	0	0	0	0	0	Proteobacteria	Alphaproteobacteria	Rhodospirillales	Rhodospirillaceae	Unassigned	Unassigned
386	3	0	0	0	0	0	0	0	0	0	0	0	3	Proteobacteria	Deltaproteobacteria	Desulfobacterales	Desulfobulbaceae	Unassigned	Unassigned
389	3	0	0	0	3	0	0	0	0	0	0	0	0	Chlorobi	Unassigned	Unassigned	Unassigned	Unassigned	Unassigned
390	3	0	0	0	0	0	0	3	0	0	0	0	0	Proteobacteria	Alphaproteobacteria	Kiloniellales	Unassigned	Unassigned	Unassigned
392	3	0	0	0	0	0	0	0	3	0	0	0	0	Proteobacteria	Gammaproteobacteria	Oceanospirillales	Alcanivoracaceae	Alcanivorax	Unassigned
393	3	0	0	1	2	0	0	0	0	0	0	0	0	Proteobacteria	Gammaproteobacteria	Alteromonadales	211ds20	Unassigned	Unassigned
394	0	0	0	0	0	0	0	0	0	0	0	0	0	GN02	BD1-5	Unassigned	Unassigned	Unassigned	Unassigned
397	3	0	1	0	0	0	2	0	0	0	0	0	0	Proteobacteria	Gammaproteobacteria	Chromatiales	Unassigned	Unassigned	Unassigned
398	3	0	0	3	0	0	0	0	0	0	0	0	0	Actinobacteria	Acidimicrobiia	Acidimicrobiales	JdFBGBact	Unassigned	Unassigned
400	3	0	0	0	0	0	0	0	0	0	0	0	3	Proteobacteria	Alphaproteobacteria	Rhodospirillales	Unassigned	Unassigned	Unassigned
402	1	0	0	0	0	1	0	0	0	0	0	0	0	Proteobacteria	Gammaproteobacteria	[Marinicellales]	[Marinicellaceae]	Unassigned	Unassigned
419	2	1	0	0	0	0	0	1	0	0	0	0	0	Bacteroidetes	Cytophagia	Cytophagales	Cyclobacteriaceae	Unassigned	Unassigned
427	3	0	0	1	0	0	0	0	0	0	0	0	2	Proteobacteria	Deltaproteobacteria	Myxococcales	Unassigned	Unassigned	Unassigned
432	0	0	0	0	0	0	0	0	0	0	0	0	0	Bacteroidetes	[Saprospirae]	[Saprospirales]	Saprospiraceae	Unassigned	Unassigned
433	3	0	0	0	0	1	2	0	0	0	0	0	0	Gemmatimonadete:	Gemm-2	Unassigned	Unassigned	Unassigned	Unassigned
437	3	0	0	0	3	0	0	0	0	0	0	0	0	Proteobacteria	Gammaproteobacteria	Oceanospirillales	Oceanospirillaceae	Marinomonas	Unassigned
446	3	0	0	0	0	0	0	0	0	0	0	1	2	Proteobacteria	Deltaproteobacteria	Desulfobacterales	Desulfobulbaceae	Unassigned	Unassigned
448	0	0	0	0	0	0	0	0	0	0	0	0	0	Proteobacteria	Alphaproteobacteria	Unassigned	Unassigned	Unassigned	Unassigned
450	3	0	0	3	0	0	0	0	0	0	0	0	0	Acidobacteria	Sva0725	Sva0725	Unassigned	Unassigned	Unassigned
456	0	0	0	0	0	0	0	0	0	0	0	0	0	Proteobacteria	Gammaproteobacteria	Thiotrichales	Thiotrichaceae	Unassigned	Unassigned
466	3	0	0	0	0	0	0	3	0	0	0	0	0	Acidobacteria	Solibacteres	Solibacterales	Solibacteraceae	Unassigned	Unassigned
471	3	0	0	0	0	0	3	0	0	0	0	0	0	Bacteroidetes	Flavobacteriia	Flavobacteriales	Flavobacteriaceae	Unassigned	Unassigned
473	1	0	0	1	0	0	0	0	0	0	0	0	0	Proteobacteria	Alphaproteobacteria	BD7-3	Unassigned	Unassigned	Unassigned
477	3	0	0	0	0	2	1	0	0	0	0	0	0	Proteobacteria	Gammaproteobacteria	Unassigned	Unassigned	Unassigned	Unassigned
479	3	0	0	0	0	2	1	0	0	0	0	0	0	Proteobacteria	Gammaproteobacteria	Chromatiales	Unassigned	Unassigned	Unassigned
481	3	0	0	0	0	0	0	1	2	0	0	0	0	Proteobacteria	Deltaproteobacteria	Myxococcales	Unassigned	Unassigned	Unassigned
484	3	0	0	0	0	0	0	0	0	0	0	2	1	Bacteroidetes	Cytophagia	Cytophagales	Flammeovirgaceae	Tunicatimonas	pelagia
486	3	0	0	0	0	0	0	0	0	0	0	2	1	Proteobacteria	Deltaproteobacteria	Desulfuromonadales	Desulfuromonadaceae	Unassigned	Unassigned
487	3	0	0	1	1	0	0	0	0	0	0	0	1	Acidobacteria	Acidobacteria-6	CCU21	Unassigned	Unassigned	Unassigned
492	3	0	0	0	0	0	0	0	0	0	0	0	3	Proteobacteria	Gammaproteobacteria	Alteromonadales	Alteromonadaceae	Unassigned	Unassigned
493	3	0	0	0	0	0	0	0	1	0	0	0	2	Proteobacteria	Gammaproteobacteria	Unassigned	Unassigned	Unassigned	Unassigned
495	3	3	0	0	0	0	0	0	0	0	0	0	0	Proteobacteria	Deltaproteobacteria	NB1-j	JTB38	Unassigned	Unassigned
499	1	0	0	0	0	0	1	0	0	0	0	0	0	Bacteroidetes	Flavobacteriia	Flavobacteriales	Cryomorphaceae	Owenweeksia	Unassigned
505	3	0	0	1	2	0	0	0	0	0	0	0	0	Proteobacteria	Gammaproteobacteria	Thiohalorhabdales	Unassigned	Unassigned	Unassigned
506	3	2	0	0	1	0	0	0	0	0	0	0	0	Proteobacteria	Deltaproteobacteria	Myxococcales	Unassigned	Unassigned	Unassigned
508	3	0	0	0	3	0	0	0	0	0	0	0	0	Proteobacteria	Deltaproteobacteria	Desulfobacterales	Desulfobulbaceae	Unassigned	Unassigned
510	3	1	0	0	0	2	0	0	0	0	0	0	0	Proteobacteria	Deltaproteobacteria	Unassigned	Unassigned	Unassigned	Unassigned
511	1	0	0	0	0	1	0	0	0	0	0	0	0	Proteobacteria	Deltaproteobacteria	PB19	Unassigned	Unassigned	Unassigned
521	3	0	1	0	1	1	0	0	0	0	0	0	0	Proteobacteria	Gammaproteobacteria	[Marinicellales]	[Marinicellaceae]	Marinicella	Unassigned
529	1	1	0	0	0	0	0	0	0	0	0	0	0	Proteobacteria	Epsilonproteobacteria	Campylobacterales	Helicobacteraceae	Unassigned	Unassigned

538	3	0	0	0	0	0	0	3	0	0	0	0	0	WS3	PRR-12	GN03	Unassigned	Unassigned	Unassigned
549	3	0	0	1	2	0	0	0	0	0	0	0	0	Chloroflexi	TK17	Unassigned	Unassigned	Unassigned	Unassigned
555	0	0	0	0	0	0	0	0	0	0	0	0	0	Proteobacteria	Deltaproteobacteria	Unassigned	Unassigned	Unassigned	Unassigned
557	1	0	0	0	0	1	0	0	0	0	0	0	0	Bacteroidetes	BME43	Unassigned	Unassigned	Unassigned	Unassigned
558	3	0	0	0	0	1	2	0	0	0	0	0	0	Gemmatimonadete	Gemm-2	Unassigned	Unassigned	Unassigned	Unassigned
559	3	0	0	0	0	1	2	0	0	0	0	0	0	Verrucomicrobia	Verrucomicrobiae	Verrucomicrobiales	Verrucomicrobiaceae	Persicirhabdus	Unassigned
569	3	0	0	0	0	2	1	0	0	0	0	0	0	Proteobacteria	Deltaproteobacteria	NB1-j	JTB38	Unassigned	Unassigned
576	0	0	0	0	0	0	0	0	0	0	0	0	0	Proteobacteria	Gammaproteobacteria	Alteromonadales	Psychromonadaceae	Psychromonas	Unassigned
585	3	0	0	0	0	0	0	0	0	0	0	2	1	WS2	SHA-109	Unassigned	Unassigned	Unassigned	Unassigned
592	3	0	0	0	0	0	0	0	0	0	0	0	3	Proteobacteria	Gammaproteobacteria	Thiotrichales	Piscirickettsiaceae	Unassigned	Unassigned
595	3	0	0	0	1	0	0	0	0	0	0	0	2	Bacteroidetes	Flavobacteriia	Flavobacteriales	Flavobacteriaceae	Unassigned	Unassigned
596	3	0	0	0	0	0	0	0	0	0	0	0	3	Spirochaetes	Spirochaetes	Spirochaetales	Spirochaetaceae	Unassigned	Unassigned
598	3	0	0	0	0	0	0	0	0	0	0	3	0	Actinobacteria	Actinobacteria	Actinomycetales	Pseudonocardiaceae	Unassigned	Unassigned
606	0	0	0	0	0	0	0	0	0	0	0	0	0	Firmicutes	Clostridia	Clostridiales	Clostridiaceae	Clostridium	Unassigned
608	0	0	0	0	0	0	0	0	0	0	0	0	0	Proteobacteria	Alphaproteobacteria	BD7-3	Unassigned	Unassigned	Unassigned
609	1	0	0	0	0	0	1	0	0	0	0	0	0	Proteobacteria	Gammaproteobacteria	Oceanospirillales	Unassigned	Unassigned	Unassigned
611	3	0	1	0	0	0	0	0	0	0	2	0	0	Verrucomicrobia	Opitutae	[Pelagicoccales]	[Pelagicoccaceae]	Pelagicoccus	Unassigned
613	0	0	0	0	0	0	0	0	0	0	0	0	0	Proteobacteria	Gammaproteobacteria	Thiotrichales	Thiotrichaceae	Unassigned	Unassigned
614	3	0	0	0	0	0	0	0	0	0	0	1	2	Bacteroidetes	[Rhodothermi]	[Rhodothermales]	Rhodothermaceae	Rubricoccus	Unassigned
615	3	0	0	0	0	0	0	3	0	0	0	0	0	Cyanobacteria	Nostocophycideae	Nostocales	Nostocaceae	Nostoc	Unassigned
618	2	0	0	0	0	0	2	0	0	0	0	0	0	Proteobacteria	Gammaproteobacteria	Alteromonadales	HTCC2188	Unassigned	Unassigned
624	3	3	0	0	0	0	0	0	0	0	0	0	0	Proteobacteria	Gammaproteobacteria	Oceanospirillales	Oceanospirillaceae	Unassigned	Unassigned
625	2	0	0	0	0	1	1	0	0	0	0	0	0	Proteobacteria	Deltaproteobacteria	Bdellovibrionales	Bdellovibrionaceae	Bdellovibrio	Unassigned
628	3	0	0	0	0	0	0	0	0	0	3	0	0	Proteobacteria	Gammaproteobacteria	Unassigned	Unassigned	Unassigned	Unassigned
630	3	2	0	0	0	1	0	0	0	0	0	0	0	Proteobacteria	Gammaproteobacteria	Thiotrichales	Piscirickettsiaceae	Unassigned	Unassigned
631	0	0	0	0	0	0	0	0	0	0	0	0	0	Firmicutes	Clostridia	Clostridiales	JTB215	Unassigned	Unassigned
633	3	0	0	3	0	0	0	0	0	0	0	0	0	Gemmatimonadete	Gemm-2	Unassigned	Unassigned	Unassigned	Unassigned
635	3	0	0	0	0	0	0	0	2	0	0	0	1	Proteobacteria	Deltaproteobacteria	Myxococcales	Unassigned	Unassigned	Unassigned
636	3	0	0	0	0	0	0	0	0	0	0	1	2	Bacteroidetes	[Rhodothermi]	[Rhodothermales]	Rhodothermaceae	Rubricoccus	Unassigned
645	2	1	0	0	0	1	0	0	0	0	0	0	0	Proteobacteria	Alphaproteobacteria	Unassigned	Unassigned	Unassigned	Unassigned
646	3	0	0	0	0	0	0	0	0	2	1	0	0	Acidobacteria	Holophagae	Holophagales	Unassigned	Unassigned	Unassigned
647	3	0	0	0	0	0	0	3	0	0	0	0	0	Bacteroidetes	[Rhodothermi]	[Rhodothermales]	[Balneolaceae]	Balneola	Unassigned
648	1	1	0	0	0	0	0	0	0	0	0	0	0	Proteobacteria	Deltaproteobacteria	Bdellovibrionales	Bdellovibrionaceae	Bdellovibrio	Unassigned
662	3	0	0	0	0	0	3	0	0	0	0	0	0	Proteobacteria	Gammaproteobacteria	Oceanospirillales	Oceanospirillaceae	Marinomonas	Unassigned
663	3	0	0	2	0	0	0	1	0	0	0	0	0	Firmicutes	Bacilli	Bacillales	[Exiguobacteraceae]	Exiguobacterium	Unassigned
665	3	2	1	0	0	0	0	0	0	0	0	0	0	Proteobacteria	Gammaproteobacteria	Legionellales	Coxiellaceae	Unassigned	Unassigned
668	3	0	0	0	0	0	0	3	0	0	0	0	0	Proteobacteria	Deltaproteobacteria	Desulfobacterales	Desulfobulbaceae	Unassigned	Unassigned
672	3	0	0	0	0	0	0	0	0	0	0	1	2	Proteobacteria	Alphaproteobacteria	Rhodospirillales	Rhodospirillaceae	Unassigned	Unassigned
676	3	1	0	2	0	0	0	0	0	0	0	0	0	Chloroflexi	Anaerolineae	SBR1031	A4b	Unassigned	Unassigned
677	3	0	0	2	0	1	0	0	0	0	0	0	0	Chloroflexi	Anaerolineae	SBR1031	A4b	Unassigned	Unassigned
684	3	0	0	1	2	0	0	0	0	0	0	0	0	Actinobacteria	Acidimicrobiia	Acidimicrobiales	SC3-41	Unassigned	Unassigned
685	2	0	0	0	1	1	0	0	0	0	0	0	0	Proteobacteria	Gammaproteobacteria	Chromatiales	Unassigned	Unassigned	Unassigned
694	1	0	0	0	0	0	0	0	0	1	0	0	0	Bacteroidetes	Flavobacteriia	Flavobacteriales	Cryomorphaceae	Unassigned	Unassigned
699	3	0	0	1	2	0	0	0	0	0	0	0	0	Bacteroidetes	Cytophagia	Cytophagales	Cytophagaceae	Unassigned	Unassigned
703	1	0	0	0	0	0	0	0	0	0	1	0	0	Firmicutes	Clostridia	Clostridiales	[Acidaminobacteraceae]	WH1-8	Unassigned

713	3	0	0	2	1	0	0	0	0	0	0	0	0	Actinobacteria	Acidimicrobiia	Acidimicrobiales	Unassigned	Unassigned	Unassigned
730	3	0	0	0	2	0	1	0	0	0	0	0	0	Acidobacteria	Sva0725	Sva0725	Unassigned	Unassigned	Unassigned
739	3	0	0	1	1	0	0	0	0	0	0	0	1	Actinobacteria	Actinobacteria	Actinomycetales	Nocardioidaceae	Unassigned	Unassigned
750	2	0	0	1	1	0	0	0	0	0	0	0	0	Proteobacteria	Alphaproteobacteria	Unassigned	Unassigned	Unassigned	Unassigned
758	3	0	0	2	1	0	0	0	0	0	0	0	0	Proteobacteria	Gammaproteobacteria	Chromatiales	Unassigned	Unassigned	Unassigned
763	3	0	0	3	0	0	0	0	0	0	0	0	0	Proteobacteria	Gammaproteobacteria	Chromatiales	Unassigned	Unassigned	Unassigned
772	1	0	0	1	0	0	0	0	0	0	0	0	0	Proteobacteria	Epsilonproteobacteria	Campylobacterales	Campylobacteraceae	Arcobacter	Unassigned
780	3	2	1	0	0	0	0	0	0	0	0	0	0	Proteobacteria	Gammaproteobacteria	Alteromonadales	OM60	Unassigned	Unassigned
784	3	0	0	0	0	0	0	3	0	0	0	0	0	Chlorobi	Ignavibacteria	Ignavibacteriales	lheB3-7	Unassigned	Unassigned
801	3	1	0	1	1	0	0	0	0	0	0	0	0	Proteobacteria	Deltaproteobacteria	NB1-j	JTB38	Unassigned	Unassigned
817	3	0	0	0	0	1	0	0	0	1	1	0	0	Proteobacteria	Deltaproteobacteria	Desulfobacterales	Desulfobulbaceae	Unassigned	Unassigned
819	3	3	0	0	0	0	0	0	0	0	0	0	0	Proteobacteria	Gammaproteobacteria	Unassigned	Unassigned	Unassigned	Unassigned
827	2	1	1	0	0	0	0	0	0	0	0	0	0	Acidobacteria	Holophagae	Holophagales	Unassigned	Unassigned	Unassigned
837	3	0	0	0	0	0	0	1	0	0	2	0	0	Proteobacteria	Deltaproteobacteria	Desulfobacterales	Desulfobulbaceae	Unassigned	Unassigned
843	2	1	0	0	0	0	1	0	0	0	0	0	0	Proteobacteria	Gammaproteobacteria	Thiotrichales	Piscirickettsiaceae	Unassigned	Unassigned
844	3	2	0	0	0	0	0	0	0	0	0	1	0	Proteobacteria	Alphaproteobacteria	BD7-3	Unassigned	Unassigned	Unassigned
857	3	0	0	0	0	0	0	2	0	0	0	1	0	Proteobacteria	Gammaproteobacteria	[Marinicellales]	[Marinicellaceae]	Unassigned	Unassigned
873	3	0	0	0	0	0	0	3	0	0	0	0	0	Proteobacteria	Gammaproteobacteria	Thiotrichales	Piscirickettsiaceae	Unassigned	Unassigned
880	3	0	0	0	0	0	0	1	0	0	0	0	2	Proteobacteria	Deltaproteobacteria	Myxococcales	Unassigned	Unassigned	Unassigned
897	3	0	0	0	0	0	0	2	1	0	0	0	0	Acidobacteria	Solibacteres	Solibacterales	PAUC26f	Unassigned	Unassigned
909	2	0	0	0	0	1	0	0	0	1	0	0	0	Proteobacteria	Alphaproteobacteria	Rhodobacterales	Hyphomonadaceae	Unassigned	Unassigned
921	3	0	0	1	0	0	2	0	0	0	0	0	0	Proteobacteria	Gammaproteobacteria	Alteromonadales	Alteromonadaceae	Unassigned	Unassigned
924	3	0	0	3	0	0	0	0	0	0	0	0	0	Proteobacteria	Alphaproteobacteria	Rhodospirillales	Unassigned	Unassigned	Unassigned
933	3	0	0	3	0	0	0	0	0	0	0	0	0	Bacteroidetes	[Saprospirae]	[Saprospirales]	Chitinophagaceae	Unassigned	Unassigned
957	3	0	0	0	0	0	3	0	0	0	0	0	0	Proteobacteria	Gammaproteobacteria	Legionellales	Unassigned	Unassigned	Unassigned
974	3	0	0	0	0	0	0	0	0	0	0	3	0	Bacteroidetes	[Saprospirae]	[Saprospirales]	Saprospiraceae	Lewinella	Unassigned
977	2	0	2	0	0	0	0	0	0	0	0	0	0	Proteobacteria	Alphaproteobacteria	Rhodospirillales	Rhodospirillaceae	Nisaea	Unassigned
994	3	0	0	1	1	0	0	0	0	0	0	1	0	Acidobacteria	[Chloracidobacteria]	RB41	Ellin6075	Unassigned	Unassigned
1040	3	1	1	0	0	0	1	0	0	0	0	0	0	Proteobacteria	Alphaproteobacteria	Rickettsiales	Unassigned	Unassigned	Unassigned
1042	1	1	0	0	0	0	0	0	0	0	0	0	0	Cyanobacteria	Synechococcophycideae	Pseudanabaenales	Pseudanabaenaceae	Halomicronema	Unassigned
1043	1	1	0	0	0	0	0	0	0	0	0	0	0	Proteobacteria	Gammaproteobacteria	Legionellales	Unassigned	Unassigned	Unassigned
1044	3	2	0	0	0	1	0	0	0	0	0	0	0	Actinobacteria	Acidimicrobiia	Acidimicrobiales	wb1_P06	Unassigned	Unassigned
1049	2	0	0	0	0	2	0	0	0	0	0	0	0	Proteobacteria	Gammaproteobacteria	Chromatiales	Unassigned	Unassigned	Unassigned
1053	0	0	0	0	0	0	0	0	0	0	0	0	0	GN02	Unassigned	Unassigned	Unassigned	Unassigned	Unassigned
1058	3	0	0	2	0	0	0	0	0	0	0	0	1	Proteobacteria	Gammaproteobacteria	Alteromonadales	Alteromonadaceae	Unassigned	Unassigned
1066	3	0	0	2	0	0	0	0	0	0	0	1	0	Chlorobi	OPB56	Unassigned	Unassigned	Unassigned	Unassigned
1093	1	0	0	0	0	0	1	0	0	0	0	0	0	Bacteroidetes	Cytophagia	Cytophagales	Flammeovirgaceae	Flexithrix	Unassigned
1107	3	0	0	1	0	0	0	0	0	0	0	2	0	Bacteroidetes	[Saprospirae]	[Saprospirales]	Saprospiraceae	Unassigned	Unassigned
1135	3	0	0	2	1	0	0	0	0	0	0	0	0	Proteobacteria	Alphaproteobacteria	Unassigned	Unassigned	Unassigned	Unassigned
1140	2	1	0	0	0	0	0	0	0	0	1	0	0	Proteobacteria	Gammaproteobacteria	Alteromonadales	Colwelliaceae	Unassigned	Unassigned
1215	3	0	0	2	1	0	0	0	0	0	0	0	0	Proteobacteria	Gammaproteobacteria	Thiotrichales	Piscirickettsiaceae	Unassigned	Unassigned
1223	3	0	0	1	1	0	0	1	0	0	0	0	0	Proteobacteria	Deltaproteobacteria	Myxococcales	Unassigned	Unassigned	Unassigned
1245	3	0	0	2	1	0	0	0	0	0	0	0	0	Proteobacteria	Alphaproteobacteria	BD7-3	Unassigned	Unassigned	Unassigned
1274	3	0	0	1	0	0	0	0	0	0	0	1	1	Proteobacteria	Gammaproteobacteria	Alteromonadales	Unassigned	Unassigned	Unassigned
1320	3	0	0	0	0	0	0	0	0	0	0	2	1	Cyanobacteria	Oscillatoriophycideae	Oscillatoriales	Phormidiaceae	Microcoleus	Unassigned

1360	3	0	1	0	0	0	0	1	0	0	0	0	1	Proteobacteria	Gammaproteobacteria	Oceanospirillales	Oceanospirillaceae	Amphritea	Unassigned
1378	3	0	0	2	1	0	0	0	0	0	0	0	0	Actinobacteria	Nitriliruptoria	Nitriliruptorales	Nitriliruptoraceae	Unassigned	Unassigned
1379	3	0	0	1	0	0	0	0	1	0	0	1	0	Bacteroidetes	Flavobacteriia	Flavobacteriales	Flavobacteriaceae	Psychroserpens	Unassigned
1389	3	0	0	1	0	0	0	0	0	0	0	1	1	Bacteroidetes	[Rhodothermi]	[Rhodothermales]	Rhodothermaceae	Rubricoccus	Unassigned
1408	3	0	0	2	1	0	0	0	0	0	0	0	0	Bacteroidetes	Cytophagia	Cytophagales	Flammeovirgaceae	Tunicatimonas	pelagia
1425	3	0	0	0	3	0	0	0	0	0	0	0	0	Proteobacteria	Gammaproteobacteria	Alteromonadales	Alteromonadaceae	Unassigned	Unassigned
1447	0	0	0	0	0	0	0	0	0	0	0	0	0	Proteobacteria	Gammaproteobacteria	Alteromonadales	OM60	Unassigned	Unassigned
1450	1	0	0	0	0	0	0	0	0	0	0	0	1	Bacteroidetes	Flavobacteriia	Flavobacteriales	Flavobacteriaceae	Kordia	Unassigned
1473	3	0	0	0	0	0	0	0	0	0	0	2	1	Bacteroidetes	[Saprospirae]	[Saprospirales]	Saprospiraceae	Lewinella	cohaerens
1498	3	0	1	0	0	2	0	0	0	0	0	0	0	Proteobacteria	Alphaproteobacteria	Rhodobacterales	Rhodobacteraceae	Unassigned	Unassigned
1501	0	0	0	0	0	0	0	0	0	0	0	0	0	Proteobacteria	Gammaproteobacteria	Vibrionales	Vibrionaceae	Vibrio	Unassigned
1516	1	0	0	0	1	0	0	0	0	0	0	0	0	Proteobacteria	Alphaproteobacteria	BD7-3	Unassigned	Unassigned	Unassigned
1585	3	0	0	0	2	0	0	0	0	0	0	1	0	Proteobacteria	Alphaproteobacteria	Rhodospirillales	Rhodospirillaceae	Unassigned	Unassigned
1627	0	0	0	0	0	0	0	0	0	0	0	0	0	Unassigned	Unassigned	Unassigned	Unassigned	Unassigned	Unassigned
1703	3	0	0	0	1	2	0	0	0	0	0	0	0	Proteobacteria	Deltaproteobacteria	NB1-j	JTB38	Unassigned	Unassigned
1728	3	1	0	0	0	1	1	0	0	0	0	0	0	Proteobacteria	Alphaproteobacteria	Rhodobacterales	Rhodobacteraceae	Anaerospora	Unassigned
1759	3	0	0	1	0	0	0	0	0	0	0	1	1	Proteobacteria	Alphaproteobacteria	Rhodobacterales	Rhodobacteraceae	Unassigned	Unassigned
1767	2	0	0	0	0	0	0	0	0	0	2	0	0	Proteobacteria	Gammaproteobacteria	Alteromonadales	Alteromonadaceae	Glaciecola	Unassigned
1768	1	0	0	0	0	0	0	0	0	1	0	0	0	Proteobacteria	Alphaproteobacteria	Kiloniellales	Kiloniellaceae	Thalassospira	Unassigned
1803	3	0	0	0	0	1	0	2	0	0	0	0	0	Proteobacteria	Epsilonproteobacteria	Campylobacterales	Helicobacteraceae	Unassigned	Unassigned
1834	1	0	0	0	0	1	0	0	0	0	0	0	0	Proteobacteria	Alphaproteobacteria	BD7-3	Unassigned	Unassigned	Unassigned
1872	3	0	1	0	1	0	0	0	0	0	0	1	0	Proteobacteria	Gammaproteobacteria	Alteromonadales	OM60	Unassigned	Unassigned
449	0	0	0	0	0	0	0	0	0	0	0	0	0	Proteobacteria	Deltaproteobacteria	Myxococcales	Unassigned	Unassigned	Unassigned
453	2	0	0	2	0	0	0	0	0	0	0	0	0	Proteobacteria	Deltaproteobacteria	Bdellovibrionales	Bacteriovoracaceae	Unassigned	Unassigned
454	2	0	0	0	0	0	0	0	0	0	2	0	0	Proteobacteria	Deltaproteobacteria	Desulfobacterales	Desulfobulbaceae	Unassigned	Unassigned
458	2	0	0	0	2	0	0	0	0	0	0	0	0	Proteobacteria	Gammaproteobacteria	Oceanospirillales	Oceanospirillaceae	Neptunomonas	Unassigned
460	2	0	0	2	0	0	0	0	0	0	0	0	0	Proteobacteria	Deltaproteobacteria	Myxococcales	Unassigned	Unassigned	Unassigned
463	2	0	0	0	0	2	0	0	0	0	0	0	0	GN02	GN07	Unassigned	Unassigned	Unassigned	Unassigned
464	2	0	0	0	0	1	0	0	0	0	1	0	0	Spirochaetes	Spirochaetes	Spirochaetales	Spirochaetaceae	Spirochaeta	Unassigned
468	2	0	0	0	0	0	0	0	2	0	0	0	0	Proteobacteria	Gammaproteobacteria	Alteromonadales	HTCC2188	HTCC	Unassigned
474	2	0	0	1	1	0	0	0	0	0	0	0	0	Proteobacteria	Gammaproteobacteria	HTCC2188	HTCC2089	Unassigned	Unassigned
475	2	0	0	1	1	0	0	0	0	0	0	0	0	Actinobacteria	Actinobacteria	Actinomycetales	Mycobacteriaceae	Mycobacterium	Unassigned
476	2	0	0	0	0	0	0	0	2	0	0	0	0	Chloroflexi	Anaerolineae	Anaerolineales	Anaerolinaceae	Unassigned	Unassigned
478	1	0	0	0	0	1	0	0	0	0	0	0	0	Proteobacteria	Gammaproteobacteria	Oceanospirillales	Unassigned	Unassigned	Unassigned
482	1	0	0	0	0	1	0	0	0	0	0	0	0	Bacteroidetes	Bacteroidia	Bacteroidales	SB-1	Unassigned	Unassigned
488	2	0	0	0	2	0	0	0	0	0	0	0	0	Proteobacteria	Gammaproteobacteria	Unassigned	Unassigned	Unassigned	Unassigned
496	2	0	0	0	0	0	0	0	0	0	0	2	0	Proteobacteria	Alphaproteobacteria	Rhizobiales	Unassigned	Unassigned	Unassigned
497	2	0	0	0	0	0	0	0	0	0	0	0	2	GN02	3BR-5F	Unassigned	Unassigned	Unassigned	Unassigned
498	0	0	0	0	0	0	0	0	0	0	0	0	0	Proteobacteria	TA18	CV90	Unassigned	Unassigned	Unassigned
500	2	0	0	0	0	0	0	0	0	0	0	2	0	Bacteroidetes	Cytophagia	Cytophagales	Flammeovirgaceae	Unassigned	Unassigned
501	0	0	0	0	0	0	0	0	0	0	0	0	0	Proteobacteria	Deltaproteobacteria	Bdellovibrionales	Bdellovibrionaceae	Bdellovibrio	Unassigned
504	2	1	1	0	0	0	0	0	0	0	0	0	0	Proteobacteria	Alphaproteobacteria	Rhizobiales	Hyphomicrobiaceae	Unassigned	Unassigned
507	2	0	0	0	0	0	0	0	0	0	0	0	2	Actinobacteria	Actinobacteria	Actinomycetales	Intrasporangiaceae	Unassigned	Unassigned
513	2	0	0	0	2	0	0	0	0	0	0	0	0	Firmicutes	Bacilli	Bacillales	[Exiguobacteraceae]	Unassigned	Unassigned
514	1	0	0	0	0	1	0	0	0	0	0	0	0	Proteobacteria	Alphaproteobacteria	BD7-3	Unassigned	Unassigned	Unassigned

518	2	0	0	0	1	0	0	0	0	0	0	0	1	Proteobacteria	Deltaproteobacteria	GMD14H09	Unassigned	Unassigned	Unassigned
519	2	0	0	0	2	0	0	0	0	0	0	0	0	Cyanobacteria	Synechococcophycideae	Pseudanabaenales	Pseudanabaenaceae	Unassigned	Unassigned
522	2	0	0	1	1	0	0	0	0	0	0	0	0	Acidobacteria	Sva0725	Sva0725	Unassigned	Unassigned	Unassigned
523	2	0	0	0	0	0	0	0	0	0	0	0	2	Proteobacteria	Deltaproteobacteria	Myxococcales	Unassigned	Unassigned	Unassigned
525	2	0	0	1	0	0	0	0	0	0	0	1	0	Proteobacteria	Gammaproteobacteria	[Marinicellales]	[Marinicellaceae]	Unassigned	Unassigned
526	2	0	0	0	1	1	0	0	0	0	0	0	0	Proteobacteria	Gammaproteobacteria	[Marinicellales]	[Marinicellaceae]	Unassigned	Unassigned
527	2	0	0	0	2	0	0	0	0	0	0	0	0	Proteobacteria	Alphaproteobacteria	Rhodospirillales	Unassigned	Unassigned	Unassigned
528	2	0	0	0	0	0	0	0	2	0	0	0	0	Proteobacteria	Gammaproteobacteria	Unassigned	Unassigned	Unassigned	Unassigned
530	0	0	0	0	0	0	0	0	0	0	0	0	0	Bacteroidetes	Flavobacteriia	Flavobacteriales	Flavobacteriaceae	Unassigned	Unassigned
535	2	0	0	0	2	0	0	0	0	0	0	0	0	OP11	WCHB1-64	d153	Unassigned	Unassigned	Unassigned
537	0	0	0	0	0	0	0	0	0	0	0	0	0	Cyanobacteria	Unassigned	Unassigned	Unassigned	Unassigned	Unassigned
539	2	0	0	2	0	0	0	0	0	0	0	0	0	Proteobacteria	Deltaproteobacteria	Syntrophobacterales	Syntrophobacteraceae	Unassigned	Unassigned
540	2	0	0	0	2	0	0	0	0	0	0	0	0	Chlorobi	SJA-28	Unassigned	Unassigned	Unassigned	Unassigned
542	0	0	0	0	0	0	0	0	0	0	0	0	0	Bacteroidetes	Flavobacteriia	Flavobacteriales	Flavobacteriaceae	Unassigned	Unassigned
544	2	0	0	0	0	0	0	2	0	0	0	0	0	Bacteroidetes	[Rhodothermi]	[Rhodothermales]	[Balneolaceae]	Balneola	Unassigned
546	2	0	0	0	0	0	0	0	0	0	0	1	1	Chlorobi	Ignavibacteria	Ignavibacteriales	Ignavibacteriaceae	Unassigned	Unassigned
547	2	0	0	0	2	0	0	0	0	0	0	0	0	Proteobacteria	Gammaproteobacteria	Alteromonadales	Unassigned	Unassigned	Unassigned
548	2	0	0	0	2	0	0	0	0	0	0	0	0	Spirochaetes	[Leptospirae]	[Leptospirales]	Leptospiraceae	Turneriella	Unassigned
550	2	0	0	0	0	1	1	0	0	0	0	0	0	Proteobacteria	Gammaproteobacteria	Unassigned	Unassigned	Unassigned	Unassigned
551	2	0	0	0	2	0	0	0	0	0	0	0	0	Acidobacteria	Sva0725	Sva0725	Unassigned	Unassigned	Unassigned
552	0	0	0	0	0	0	0	0	0	0	0	0	0	Proteobacteria	Betaproteobacteria	Burkholderiales	Alcaligenaceae	Unassigned	Unassigned
554	0	0	0	0	0	0	0	0	0	0	0	0	0	Bacteroidetes	Flavobacteriia	Flavobacteriales	Cryomorphaceae	Owenweeksia	Unassigned
556	2	0	0	0	2	0	0	0	0	0	0	0	0	Proteobacteria	Deltaproteobacteria	Desulfobacterales	Desulfobulbaceae	Unassigned	Unassigned
561	2	1	0	0	0	0	1	0	0	0	0	0	0	Proteobacteria	Gammaproteobacteria	Alteromonadales	HTCC2188	HTCC	Unassigned
562	2	0	0	0	0	1	1	0	0	0	0	0	0	Proteobacteria	Betaproteobacteria	Methylophilales	Methylophilaceae	Methylotenera	mobilis
564	2	0	0	0	0	0	0	0	0	0	0	0	2	Actinobacteria	Thermoleophilia	Solirubrobacterales	Unassigned	Unassigned	Unassigned
565	2	0	0	0	0	0	0	0	0	0	0	0	2	Spirochaetes	Spirochaetes	Spirochaetales	Spirochaetaceae	Unassigned	Unassigned
566	2	0	0	0	0	0	2	0	0	0	0	0	0	Cyanobacteria	Synechococcophycideae	Pseudanabaenales	Pseudanabaenaceae	Unassigned	Unassigned
567	0	0	0	0	0	0	0	0	0	0	0	0	0	Bacteroidetes	[Saprospirae]	[Saprospirales]	Saprospiraceae	Saprospira	Unassigned
570	2	0	0	0	0	0	0	0	0	0	0	0	2	Proteobacteria	Deltaproteobacteria	Bdellovibrionales	Bacteriovoracaceae	Unassigned	Unassigned
571	2	0	0	1	1	0	0	0	0	0	0	0	0	Proteobacteria	Deltaproteobacteria	Myxococcales	Unassigned	Unassigned	Unassigned
578	2	0	0	2	0	0	0	0	0	0	0	0	0	Spirochaetes	[Leptospirae]	[Leptospirales]	Leptospiraceae	Leptonema	Unassigned
583	2	0	0	0	0	0	0	1	0	0	0	1	0	Proteobacteria	Gammaproteobacteria	Thiotrichales	Piscirickettsiaceae	Methylophaga	Unassigned
584	2	0	0	0	0	0	0	0	0	0	0	0	2	Proteobacteria	Gammaproteobacteria	Alteromonadales	Alteromonadaceae	CandidatusEndobugula	Unassigned
589	2	0	0	2	0	0	0	0	0	0	0	0	0	Proteobacteria	Alphaproteobacteria	Rhodospirillales	Unassigned	Unassigned	Unassigned
591	2	0	0	1	0	0	0	1	0	0	0	0	0	Proteobacteria	Betaproteobacteria	Burkholderiales	Comamonadaceae	Rhodoferax	Unassigned
593	2	1	0	0	0	0	0	0	0	1	0	0	0	Proteobacteria	Gammaproteobacteria	Alteromonadales	OM60	Unassigned	Unassigned
594	2	0	0	0	0	0	0	0	0	0	0	0	2	Gemmatimonadetes	Gemm-4	Unassigned	Unassigned	Unassigned	Unassigned
597	2	0	0	0	0	0	2	0	0	0	0	0	0	Verrucomicrobia	Verrucomicrobiae	Verrucomicrobiales	Verrucomicrobiaceae	Verrucomicrobium	Unassigned
600	2	0	0	2	0	0	0	0	0	0	0	0	0	Proteobacteria	Alphaproteobacteria	Unassigned	Unassigned	Unassigned	Unassigned
601	0	0	0	0	0	0	0	0	0	0	0	0	0	Proteobacteria	Alphaproteobacteria	Rickettsiales	Unassigned	Unassigned	Unassigned
602	2	2	0	0	0	0	0	0	0	0	0	0	0	Proteobacteria	Deltaproteobacteria	Myxococcales	Unassigned	Unassigned	Unassigned
604	2	0	0	1	0	0	0	0	0	0	0	1	0	Chloroflexi	Anaerolineae	SBR1031	SJA-101	Unassigned	Unassigned
605	1	0	1	0	0	0	0	0	0	0	0	0	0	Proteobacteria	Deltaproteobacteria	GMD14H09	Unassigned	Unassigned	Unassigned
607	0	0	0	0	0	0	0	0	0	0	0	0	0	Proteobacteria	Gammaproteobacteria	Thiotrichales	Thiotrichaceae	Unassigned	Unassigned

610	2	0	0	0	0	0	0	0	0	0	0	0	2	Proteobacteria	Betaproteobacteria	Unassigned	Unassigned	Unassigned	Unassigned
616	2	0	0	0	2	0	0	0	0	0	0	0	0	Bacteroidetes	Flavobacteriia	Flavobacteriales	Cryomorphaceae	Lishizhenia	caseinilytica
621	0	0	0	0	0	0	0	0	0	0	0	0	0	WS6	SC72	Unassigned	Unassigned	Unassigned	Unassigned
623	2	0	0	0	0	0	1	0	1	0	0	0	0	Proteobacteria	Gammaproteobacteria	Pseudomonadales	Pseudomonadaceae	Pseudomonas	pseudoalcaligenes
626	2	0	0	0	0	0	0	0	0	0	0	2	0	Proteobacteria	Deltaproteobacteria	PB19	Unassigned	Unassigned	Unassigned
632	2	0	0	2	0	0	0	0	0	0	0	0	0	Cyanobacteria	Oscillatoriophycideae	Chroococcales	Xenococcaceae	Unassigned	Unassigned
634	2	0	0	0	0	0	2	0	0	0	0	0	0	Proteobacteria	Gammaproteobacteria	Alteromonadales	211ds20	Unassigned	Unassigned
638	2	0	0	0	0	0	0	2	0	0	0	0	0	Proteobacteria	Deltaproteobacteria	Myxococcales	Haliangiaceae	Unassigned	Unassigned
641	2	1	1	0	0	0	0	0	0	0	0	0	0	Chloroflexi	Anaerolineae	SBR1031	SJA-101	Unassigned	Unassigned
644	2	0	0	0	0	0	0	0	2	0	0	0	0	Bacteroidetes	Cytophagia	Cytophagales	Cytophagaceae	Unassigned	Unassigned
650	1	1	0	0	0	0	0	0	0	0	0	0	0	Proteobacteria	Alphaproteobacteria	Rhodospirillales	Rhodospirillaceae	Nisaea	Unassigned
652	2	0	0	0	0	1	0	1	0	0	0	0	0	Proteobacteria	Deltaproteobacteria	Syntrophobacterales	Syntrophobacteraceae	Unassigned	Unassigned
654	2	0	0	0	0	0	0	0	0	0	0	2	0	Actinobacteria	Nitriliruptoria	Nitriliruptorales	Nitriliruptoraceae	Unassigned	Unassigned
655	2	0	0	0	0	0	0	0	0	0	2	0	0	Proteobacteria	Deltaproteobacteria	Bdellovibrionales	Bacteriovoracaceae	Unassigned	Unassigned
656	2	0	0	0	0	0	0	0	0	0	0	0	2	Proteobacteria	Gammaproteobacteria	HOC36	Unassigned	Unassigned	Unassigned
657	2	0	0	0	0	0	0	0	0	0	0	0	2	Proteobacteria	Gammaproteobacteria	Oceanospirillales	Oceanospirillaceae	Unassigned	Unassigned
658	2	1	0	0	0	1	0	0	0	0	0	0	0	Proteobacteria	Gammaproteobacteria	Chromatiales	Unassigned	Unassigned	Unassigned
659	2	0	0	0	0	1	0	0	0	0	1	0	0	Proteobacteria	Deltaproteobacteria	NB1-j	JTB38	Unassigned	Unassigned
660	2	0	0	1	0	0	0	0	1	0	0	0	0	Proteobacteria	Gammaproteobacteria	Legionellales	Coxiellaceae	Unassigned	Unassigned
664	2	0	0	0	0	0	2	0	0	0	0	0	0	Bacteroidetes	Cytophagia	Cytophagales	Flammeovirgaceae	Unassigned	Unassigned
666	2	0	0	2	0	0	0	0	0	0	0	0	0	Bacteroidetes	[Saprospirae]	[Saprospirales]	Saprospiraceae	Unassigned	Unassigned
667	0	0	0	0	0	0	0	0	0	0	0	0	0	Bacteroidetes	Flavobacteriia	Flavobacteriales	Flavobacteriaceae	Unassigned	Unassigned
670	2	0	0	0	0	0	0	0	0	0	0	0	2	Bacteroidetes	Flavobacteriia	Flavobacteriales	Flavobacteriaceae	Aquimarina	intermedia
671	1	0	0	0	0	0	0	0	0	0	1	0	0	Proteobacteria	Deltaproteobacteria	Desulfovibrionales	Desulfomicrobiaceae	Unassigned	Unassigned
673	2	0	0	2	0	0	0	0	0	0	0	0	0	Proteobacteria	Deltaproteobacteria	Myxococcales	Unassigned	Unassigned	Unassigned
674	2	0	0	1	0	0	0	0	0	0	0	1	0	Chloroflexi	Anaerolineae	SBR1031	A4b	Unassigned	Unassigned
678	2	2	0	0	0	0	0	0	0	0	0	0	0	Proteobacteria	Gammaproteobacteria	Legionellales	Unassigned	Unassigned	Unassigned
680	2	0	0	2	0	0	0	0	0	0	0	0	0	Proteobacteria	Alphaproteobacteria	Rhodospirillales	Rhodospirillaceae	Unassigned	Unassigned
687	1	0	0	0	0	1	0	0	0	0	0	0	0	Proteobacteria	Alphaproteobacteria	BD7-3	Unassigned	Unassigned	Unassigned
691	0	0	0	0	0	0	0	0	0	0	0	0	0	Proteobacteria	Epsilonproteobacteria	Campylobacterales	Helicobacteraceae	Sulfurimonas	Unassigned
700	1	0	1	0	0	0	0	0	0	0	0	0	0	Proteobacteria	Alphaproteobacteria	Rhizobiales	Phyllobacteriaceae	Unassigned	Unassigned
702	0	0	0	0	0	0	0	0	0	0	0	0	0	GN02	Unassigned	Unassigned	Unassigned	Unassigned	Unassigned
709	2	0	0	0	1	0	0	1	0	0	0	0	0	Bacteroidetes	Flavobacteriia	Flavobacteriales	Unassigned	Unassigned	Unassigned
717	2	0	0	0	1	0	0	1	0	0	0	0	0	TM6	SJA-4	Unassigned	Unassigned	Unassigned	Unassigned
718	2	0	0	0	2	0	0	0	0	0	0	0	0	Firmicutes	Bacilli	Bacillales	Unassigned	Unassigned	Unassigned
722	2	0	0	0	1	0	0	0	0	0	0	1	0	Bacteroidetes	Bacteroidia	Bacteroidales	Unassigned	Unassigned	Unassigned
723	2	0	0	0	1	0	0	0	0	0	0	1	0	Fibrobacteres	Fibrobacteria	Ucp1540	Unassigned	Unassigned	Unassigned
726	2	0	0	2	0	0	0	0	0	0	0	0	0	Proteobacteria	Deltaproteobacteria	Myxococcales	Unassigned	Unassigned	Unassigned
731	2	0	0	1	1	0	0	0	0	0	0	0	0	Proteobacteria	Alphaproteobacteria	BD7-3	Unassigned	Unassigned	Unassigned
742	2	0	0	1	0	1	0	0	0	0	0	0	0	Cyanobacteria	4C0d-2	MLE1-12	Unassigned	Unassigned	Unassigned
744	1	0	0	0	0	0	1	0	0	0	0	0	0	Proteobacteria	Deltaproteobacteria	Spirobacillales	Unassigned	Unassigned	Unassigned
746	2	1	0	0	1	0	0	0	0	0	0	0	0	Verrucomicrobia	Verrucomicrobiae	Verrucomicrobiales	Verrucomicrobiaceae	Haloferula	Unassigned
751	2	0	0	1	1	0	0	0	0	0	0	0	0	Chloroflexi	Anaerolineae	SBR1031	SJA-101	Unassigned	Unassigned
754	2	0	0	1	1	0	0	0	0	0	0	0	0	Acidobacteria	Solibacteres	Solibacterales	PAUC26f	Unassigned	Unassigned
757	2	0	0	0	0	0	0	0	2	0	0	0	0	Proteobacteria	Gammaproteobacteria	Alteromonadales	Alteromonadaceae	Unassigned	Unassigned

762	2	0	0	0	0	0	0	0	1	0	0	0	1 Bacteroidetes	Flavobacteriia	Flavobacteriales	Flavobacteriaceae	Unassigned	Unassigned
769	0	0	0	0	0	0	0	0	0	0	0	0	0 Proteobacteria	Gammaproteobacteria	Alteromonadales	Alteromonadaceae	Glaciecola	Unassigned
773	2	0	0	2	0	0	0	0	0	0	0	0	0 Proteobacteria	Deltaproteobacteria	Desulfuromonadales	Desulfuromonadaceae	Pelobacter	acidigallici
777	2	0	0	0	0	0	2	0	0	0	0	0	0 Proteobacteria	Gammaproteobacteria	Alteromonadales	OM60	Unassigned	Unassigned
785	2	0	0	0	0	0	0	1	1	0	0	0	0 Bacteroidetes	[Rhodothermi]	[Rhodothermales]	[Balneolaceae]	Balneola	Unassigned
789	2	2	0	0	0	0	0	0	0	0	0	0	0 Proteobacteria	Betaproteobacteria	Nitrosomonadales	Nitrosomonadaceae	Unassigned	Unassigned
791	2	0	0	0	0	0	0	0	0	0	2	0	0 Proteobacteria	Deltaproteobacteria	Desulfobacterales	Desulfobulbaceae	Desulfobulbus	Unassigned
797	2	2	0	0	0	0	0	0	0	0	0	0	0 Proteobacteria	Deltaproteobacteria	Myxococcales	Polyangiaceae	Unassigned	Unassigned
800	1	1	0	0	0	0	0	0	0	0	0	0	0 Bacteroidetes	Flavobacteriia	Flavobacteriales	Cryomorphaceae	Crocinitomix	Unassigned
803	2	1	0	0	0	0	0	0	0	0	0	0	1 Proteobacteria	Deltaproteobacteria	NB1-j	JTB38	Unassigned	Unassigned
804	2	0	0	0	0	1	1	0	0	0	0	0	0 Proteobacteria	Deltaproteobacteria	Myxococcales	Unassigned	Unassigned	Unassigned
813	2	0	0	1	0	1	0	0	0	0	0	0	0 Proteobacteria	Deltaproteobacteria	Myxococcales	Unassigned	Unassigned	Unassigned
814	2	0	0	1	0	1	0	0	0	0	0	0	0 Gemmatimonadete:	Gemm-2	Unassigned	Unassigned	Unassigned	Unassigned
815	2	0	0	0	0	1	0	0	0	0	0	0	1 Proteobacteria	Alphaproteobacteria	Rhizobiales	Rhizobiaceae	Unassigned	Unassigned
828	0	0	0	0	0	0	0	0	0	0	0	0	0 TM6	SJA-4	Unassigned	Unassigned	Unassigned	Unassigned
829	0	0	0	0	0	0	0	0	0	0	0	0	0 Proteobacteria	Deltaproteobacteria	Unassigned	Unassigned	Unassigned	Unassigned
832	2	0	0	0	0	0	0	0	0	0	2	0	0 Proteobacteria	Deltaproteobacteria	Desulfobacterales	Desulfobulbaceae	Unassigned	Unassigned
833	0	0	0	0	0	0	0	0	0	0	0	0	0 Proteobacteria	Deltaproteobacteria	GMD14H09	Unassigned	Unassigned	Unassigned
836	2	0	0	0	0	0	0	0	0	1	1	0	0 Proteobacteria	Gammaproteobacteria	Thiotrichales	Piscirickettsiaceae	Unassigned	Unassigned
851	2	0	0	0	0	0	0	2	0	0	0	0	0 Actinobacteria	Nitriliruptoria	Euzebyales	Euzebyaceae	Euzebya	Unassigned
853	2	0	0	0	0	0	0	2	0	0	0	0	0 Bacteroidetes	Cytophagia	Cytophagales	Flammeovirgaceae	Unassigned	Unassigned
855	2	1	0	0	0	0	0	0	0	1	0	0	0 Chloroflexi	Anaerolineae	SBR1031	SJA-101	Unassigned	Unassigned
866	2	0	0	0	0	1	0	0	0	0	0	1	0 Proteobacteria	Deltaproteobacteria	Unassigned	Unassigned	Unassigned	Unassigned
874	2	0	0	0	0	0	0	2	0	0	0	0	0 Acidobacteria	Sva0725	Sva0725	Unassigned	Unassigned	Unassigned
875	2	0	0	0	1	0	0	0	0	0	0	1	0 Proteobacteria	Alphaproteobacteria	Rhizobiales	Hyphomicrobiaceae	Unassigned	Unassigned
891	2	0	0	0	0	0	2	0	0	0	0	0	0 Proteobacteria	Alphaproteobacteria	Rhizobiales	Hyphomicrobiaceae	Unassigned	Unassigned
894	2	0	0	0	1	0	1	0	0	0	0	0	0 Verrucomicrobia	Opitutae	[Pelagicoccales]	[Pelagicoccaceae]	Pelagicoccus	Unassigned
906	2	1	0	0	0	0	1	0	0	0	0	0	0 Proteobacteria	Gammaproteobacteria	HTCC2188	HTCC2089	Unassigned	Unassigned
911	2	1	0	0	0	1	0	0	0	0	0	0	0 Nitrospirae	Nitrospira	Nitrospirales	Nitrospiraceae	Unassigned	Unassigned
913	2	0	0	0	0	0	0	2	0	0	0	0	0 Proteobacteria	Gammaproteobacteria	Alteromonadales	Alteromonadaceae	Porticoccus	Unassigned
919	2	0	0	1	0	0	0	0	0	0	0	1	0 Bacteroidetes	[Saprospirae]	[Saprospirales]	Saprospiraceae	Unassigned	Unassigned
935	2	0	0	0	2	0	0	0	0	0	0	0	0 Acidobacteria	[Chloracidobacteria]	RB41	Ellin6075	Unassigned	Unassigned
944	2	0	0	2	0	0	0	0	0	0	0	0	0 Acidobacteria	[Chloracidobacteria]	RB41	Ellin6075	Unassigned	Unassigned
945	2	0	0	2	0	0	0	0	0	0	0	0	0 Bacteroidetes	[Saprospirae]	[Saprospirales]	Saprospiraceae	Lewinella	nigricans
949	2	0	0	1	0	0	0	0	1	0	0	0	0 Chloroflexi	Anaerolineae	SBR1031	A4b	Unassigned	Unassigned
952	0	0	0	0	0	0	0	0	0	0	0	0	0 Fusobacteria	Fusobacteriia	Fusobacteriales	Fusobacteriaceae	Fusobacterium	Unassigned
959	0	0	0	0	0	0	0	0	0	0	0	0	0 Bacteroidetes	Bacteroidia	Bacteroidales	Unassigned	Unassigned	Unassigned
962	1	0	0	0	0	0	0	0	0	0	0	1	0 Proteobacteria	Deltaproteobacteria	GMD14H09	Unassigned	Unassigned	Unassigned
966	1	0	0	0	0	0	1	0	0	0	0	0	0 TM6	SJA-4	Unassigned	Unassigned	Unassigned	Unassigned
969	2	0	0	0	0	0	0	0	0	0	0	1	1 Bacteroidetes	[Saprospirae]	[Saprospirales]	Saprospiraceae	Lewinella	Unassigned
970	0	0	0	0	0	0	0	0	0	0	0	0	0 GN02	Unassigned	Unassigned	Unassigned	Unassigned	Unassigned
975	2	0	0	0	0	0	0	0	0	0	0	1	1 Bacteroidetes	[Rhodothermi]	[Rhodothermales]	Rhodothermaceae	Unassigned	Unassigned
984	2	0	0	2	0	0	0	0	0	0	0	0	0 Proteobacteria	Alphaproteobacteria	Unassigned	Unassigned	Unassigned	Unassigned
985	2	0	0	1	0	0	0	1	0	0	0	0	0 Actinobacteria	Actinobacteria	Actinomycetales	Intrasporangiaceae	Unassigned	Unassigned
986	2	0	0	2	0	0	0	0	0	0	0	0	0 Verrucomicrobia	Verrucomicrobiae	Verrucomicrobiales	Verrucomicrobiaceae	Luteolibacter	Unassigned

988	2	0	0	1	0	1	0	0	0	0	0	0	0	Gemmatimonadete	Gemm-2	Unassigned	Unassigned	Unassigned	Unassigned
997	0	0	0	0	0	0	0	0	0	0	0	0	0	Bacteroidetes	Flavobacteriia	Flavobacteriales	Flavobacteriaceae	Unassigned	Unassigned
1000	2	0	0	0	0	0	0	0	0	0	0	1	1	Bacteroidetes	[Saprospirae]	[Saprospirales]	Saprospiraceae	Unassigned	Unassigned
1001	2	0	0	0	0	0	0	0	0	0	0	1	1	Actinobacteria	Actinobacteria	Actinomycetales	Unassigned	Unassigned	Unassigned
1006	2	0	0	0	0	0	0	0	0	0	0	0	2	Bacteroidetes	Cytophagia	Cytophagales	Flammeovirgaceae	Flexithrix	Unassigned
1008	1	0	1	0	0	0	0	0	0	0	0	0	0	Proteobacteria	Deltaproteobacteria	Desulfovibrionales	Desulfomicrobiaceae	Desulfomicrobium	Unassigned
1009	2	0	1	0	0	0	1	0	0	0	0	0	0	Bacteroidetes	Cytophagia	Cytophagales	Flammeovirgaceae	Unassigned	Unassigned
1014	2	0	0	0	0	0	0	1	0	0	0	1	0	Bacteroidetes	Flavobacteriia	Flavobacteriales	Cryomorphaceae	Owenweeksia	Unassigned
1019	0	0	0	0	0	0	0	0	0	0	0	0	0	Proteobacteria	Gammaproteobacteria	Legionellales	Francisellaceae	Unassigned	Unassigned
1028	2	0	0	0	0	2	0	0	0	0	0	0	0	Proteobacteria	Gammaproteobacteria	Legionellales	Unassigned	Unassigned	Unassigned
1039	1	0	0	0	0	1	0	0	0	0	0	0	0	Proteobacteria	Alphaproteobacteria	BD7-3	Unassigned	Unassigned	Unassigned
1050	2	0	0	0	0	1	1	0	0	0	0	0	0	Proteobacteria	Gammaproteobacteria	Alteromonadales	OM60	Unassigned	Unassigned
1052	0	0	0	0	0	0	0	0	0	0	0	0	0	GN02	3BR-5F	Unassigned	Unassigned	Unassigned	Unassigned
1060	2	0	0	1	1	0	0	0	0	0	0	0	0	Proteobacteria	Deltaproteobacteria	Unassigned	Unassigned	Unassigned	Unassigned
1062	2	0	0	1	0	0	0	0	0	0	0	1	0	Acidobacteria	Sva0725	Sva0725	Unassigned	Unassigned	Unassigned
1064	0	0	0	0	0	0	0	0	0	0	0	0	0	Bacteroidetes	Flavobacteriia	Flavobacteriales	Cryomorphaceae	Unassigned	Unassigned
1072	2	0	1	1	0	0	0	0	0	0	0	0	0	Unassigned	Unassigned	Unassigned	Unassigned	Unassigned	Unassigned
1074	2	0	0	0	0	1	0	0	0	0	0	1	0	WPS-2	Unassigned	Unassigned	Unassigned	Unassigned	Unassigned
1078	2	0	0	2	0	0	0	0	0	0	0	0	0	Proteobacteria	Gammaproteobacteria	HOC36	Unassigned	Unassigned	Unassigned
1079	2	0	0	1	0	0	0	0	1	0	0	0	0	Bacteroidetes	Flavobacteriia	Flavobacteriales	Flavobacteriaceae	Flavobacterium	Unassigned
1080	2	0	0	0	0	0	0	0	2	0	0	0	0	Proteobacteria	Gammaproteobacteria	Alteromonadales	Alteromonadaceae	Unassigned	Unassigned
1085	2	0	0	2	0	0	0	0	0	0	0	0	0	Proteobacteria	Deltaproteobacteria	NB1-j	JTB38	Unassigned	Unassigned
1088	1	0	0	0	0	0	1	0	0	0	0	0	0	Firmicutes	Clostridia	Clostridiales	Lachnospiraceae	Unassigned	Unassigned
1098	2	0	0	2	0	0	0	0	0	0	0	0	0	Proteobacteria	Alphaproteobacteria	Rhodospirillales	Unassigned	Unassigned	Unassigned
1102	0	0	0	0	0	0	0	0	0	0	0	0	0	Bacteroidetes	Flavobacteriia	Flavobacteriales	Flavobacteriaceae	Winogradskyella	thalassocola
1103	1	0	1	0	0	0	0	0	0	0	0	0	0	Proteobacteria	Gammaproteobacteria	Legionellales	Coxiellaceae	Unassigned	Unassigned
1112	2	0	0	0	0	0	0	0	0	0	0	2	0	Acidobacteria	OS-K	Unassigned	Unassigned	Unassigned	Unassigned
1113	1	0	0	0	0	0	0	0	0	0	0	1	0	Bacteroidetes	Flavobacteriia	Flavobacteriales	Cryomorphaceae	Unassigned	Unassigned
1118	2	0	0	1	0	1	0	0	0	0	0	0	0	Proteobacteria	Deltaproteobacteria	NB1-j	JTB38	Unassigned	Unassigned
1119	2	0	0	2	0	0	0	0	0	0	0	0	0	[Thermi]	Deinococci	Deinococcales	Trueperaceae	Unassigned	Unassigned
1126	2	0	0	1	0	0	0	0	0	0	0	1	0	Chlorobi	Ignavibacteria	Ignavibacteriales	Ignavibacteriaceae	Unassigned	Unassigned
1128	2	0	0	2	0	0	0	0	0	0	0	0	0	Proteobacteria	Deltaproteobacteria	NB1-j	JTB38	Unassigned	Unassigned
1131	2	0	0	0	0	0	0	0	2	0	0	0	0	Bacteroidetes	Cytophagia	Cytophagales	Flammeovirgaceae	Unassigned	Unassigned
1141	2	0	0	1	1	0	0	0	0	0	0	0	0	Proteobacteria	Betaproteobacteria	Rhodocyclales	Rhodocyclaceae	Azoarcus	Unassigned
1147	2	0	0	0	0	0	0	2	0	0	0	0	0	Bacteroidetes	[Rhodothermi]	[Rhodothermales]	[Balneolaceae]	KSA1	Unassigned
1150	2	0	0	0	0	0	0	2	0	0	0	0	0	Proteobacteria	Gammaproteobacteria	Alteromonadales	Alteromonadaceae	BD2-13	Unassigned
1158	2	1	0	0	0	0	1	0	0	0	0	0	0	Proteobacteria	Gammaproteobacteria	Thiotrichales	Piscirickettsiaceae	Methylophaga	Unassigned
1162	2	1	0	0	0	0	1	0	0	0	0	0	0	Proteobacteria	Deltaproteobacteria	NB1-j	JTB38	Unassigned	Unassigned
1172	0	0	0	0	0	0	0	0	0	0	0	0	0	Proteobacteria	Gammaproteobacteria	Legionellales	Francisellaceae	Unassigned	Unassigned
1174	1	0	0	0	0	0	0	0	0	0	0	1	0	Proteobacteria	Deltaproteobacteria	NB1-j	JTB38	Unassigned	Unassigned
1177	0	0	0	0	0	0	0	0	0	0	0	0	0	Proteobacteria	Gammaproteobacteria	Chromatiales	Chromatiaceae	Unassigned	Unassigned
1178	1	0	0	0	0	0	1	0	0	0	0	0	0	Bacteroidetes	Flavobacteriia	Flavobacteriales	Cryomorphaceae	Cryomorpha	Unassigned
1179	1	0	0	0	0	0	1	0	0	0	0	0	0	Proteobacteria	Deltaproteobacteria	Myxococcales	Unassigned	Unassigned	Unassigned
1181	2	0	0	1	0	0	0	0	0	0	0	1	0	Proteobacteria	Gammaproteobacteria	Thiotrichales	Piscirickettsiaceae	Unassigned	Unassigned
1183	2	0	0	0	1	0	0	0	1	0	0	0	0	Proteobacteria	Gammaproteobacteria	[Marinicellales]	[Marinicellaceae]	Unassigned	Unassigned

1185	2	0	0	2	0	0	0	0	0	0	0	0	0	Proteobacteria	Deltaproteobacteria	Spirobacillales	Unassigned	Unassigned	Unassigned
1198	2	0	0	0	1	0	0	0	0	0	1	0	0	Bacteroidetes	Bacteroidia	Bacteroidales	Unassigned	Unassigned	Unassigned
1200	2	0	0	0	2	0	0	0	0	0	0	0	0	Proteobacteria	Gammaproteobacteria	Oceanospirillales	Unassigned	Unassigned	Unassigned
1203	2	0	0	0	0	0	2	0	0	0	0	0	0	Spirochaetes	[Leptospirae]	[Leptospirales]	Leptospiraceae	Leptonema	Unassigned
1216	2	0	0	1	0	0	0	1	0	0	0	0	0	Actinobacteria	Acidimicrobiia	Acidimicrobiales	Unassigned	Unassigned	Unassigned
1221	2	0	0	0	0	2	0	0	0	0	0	0	0	Bacteroidetes	Cytophagia	Cytophagales	[Amoebophilaceae]	SC3-56	Unassigned
1228	2	0	0	0	0	0	0	0	2	0	0	0	0	Bacteroidetes	Cytophagia	Cytophagales	Flammeovirgaceae	Unassigned	Unassigned
1229	2	0	0	1	0	0	0	0	1	0	0	0	0	Chloroflexi	Anaerolineae	SBR1031	A4b	Unassigned	Unassigned
1233	2	0	0	0	0	0	0	0	2	0	0	0	0	Bacteroidetes	Flavobacteriia	Flavobacteriales	Cryomorphaceae	Unassigned	Unassigned
1235	2	0	0	0	0	0	0	0	2	0	0	0	0	Proteobacteria	Gammaproteobacteria	Unassigned	Unassigned	Unassigned	Unassigned
1240	2	0	0	2	0	0	0	0	0	0	0	0	0	Proteobacteria	Alphaproteobacteria	Unassigned	Unassigned	Unassigned	Unassigned
1246	2	0	0	1	0	0	0	0	0	0	0	1	0	Bacteroidetes	Bacteroidia	Bacteroidales	SB-1	Unassigned	Unassigned
1247	2	0	0	0	0	0	0	0	0	0	0	1	1	Acidobacteria	Sva0725	Sva0725	Unassigned	Unassigned	Unassigned
1253	2	0	0	0	0	0	0	0	0	0	0	2	0	Acidobacteria	OS-K	Unassigned	Unassigned	Unassigned	Unassigned
1262	2	0	0	2	0	0	0	0	0	0	0	0	0	Proteobacteria	Deltaproteobacteria	Desulfuromonadales	Unassigned	Unassigned	Unassigned
1263	2	0	0	2	0	0	0	0	0	0	0	0	0	Proteobacteria	Deltaproteobacteria	NB1-j	JTB38	Unassigned	Unassigned
1279	2	0	0	0	0	0	0	1	0	0	0	1	0	Proteobacteria	Gammaproteobacteria	Thiotrichales	Piscirickettsiaceae	Methylophaga	Unassigned
1282	1	0	0	0	0	1	0	0	0	0	0	0	0	Proteobacteria	Deltaproteobacteria	GMD14H09	Unassigned	Unassigned	Unassigned
1288	2	0	0	0	0	1	1	0	0	0	0	0	0	Proteobacteria	Gammaproteobacteria	Chromatiales	Unassigned	Unassigned	Unassigned
1295	2	0	0	0	1	0	0	1	0	0	0	0	0	Bacteroidetes	Flavobacteriia	Flavobacteriales	Flavobacteriaceae	Flavobacterium	gelidilacus
1303	1	0	0	0	0	0	0	0	1	0	0	0	0	Bacteroidetes	Sphingobacteriia	Sphingobacteriales	Unassigned	Unassigned	Unassigned
1304	0	0	0	0	0	0	0	0	0	0	0	0	0	Bacteroidetes	[Rhodothermi]	[Rhodothermales]	[Balneolaceae]	Unassigned	Unassigned
1306	0	0	0	0	0	0	0	0	0	0	0	0	0	Bacteroidetes	Cytophagia	Cytophagales	Cytophagaceae	Leadbetterella	Unassigned
1313	2	0	0	0	0	0	0	0	0	0	0	1	1	GN04	GN15	Unassigned	Unassigned	Unassigned	Unassigned
1331	2	0	0	0	0	0	0	0	0	0	0	2	0	Proteobacteria	Deltaproteobacteria	Myxococcales	Unassigned	Unassigned	Unassigned
1347	0	0	0	0	0	0	0	0	0	0	0	0	0	Proteobacteria	Alphaproteobacteria	Rhizobiales	Unassigned	Unassigned	Unassigned
1349	1	0	0	0	0	0	1	0	0	0	0	0	0	Proteobacteria	Gammaproteobacteria	Legionellales	Francisellaceae	Unassigned	Unassigned
1353	2	0	0	0	0	0	0	0	0	0	0	0	2	Bacteroidetes	[Rhodothermi]	[Rhodothermales]	Rhodothermaceae	Rubricoccus	Unassigned
1354	0	0	0	0	0	0	0	0	0	0	0	0	0	Proteobacteria	Alphaproteobacteria	Rickettsiales	Rickettsiaceae	Unassigned	Unassigned
1363	0	0	0	0	0	0	0	0	0	0	0	0	0	Firmicutes	Clostridia	Clostridiales	Veillonellaceae	Acidaminococcus	Unassigned
1368	0	0	0	0	0	0	0	0	0	0	0	0	0	Proteobacteria	Epsilonproteobacteria	Campylobacterales	Helicobacteraceae	Unassigned	Unassigned
1373	2	0	0	1	1	0	0	0	0	0	0	0	0	Proteobacteria	Deltaproteobacteria	Bdellovibrionales	Bacteriovoracaceae	Unassigned	Unassigned
1385	2	0	1	1	0	0	0	0	0	0	0	0	0	Proteobacteria	Gammaproteobacteria	Alteromonadales	Alteromonadaceae	Gilvimarinus	chinensis
1387	2	0	0	0	0	0	0	0	0	0	0	0	2	Proteobacteria	Alphaproteobacteria	Rhodobacterales	Rhodobacteraceae	Unassigned	Unassigned
1388	2	0	0	0	0	0	0	0	0	0	0	1	1	Proteobacteria	Alphaproteobacteria	Rhodospirillales	Rhodospirillaceae	Unassigned	Unassigned
1392	2	0	0	1	0	0	0	0	0	0	0	0	1	Proteobacteria	Gammaproteobacteria	Unassigned	Unassigned	Unassigned	Unassigned
1399	2	0	0	0	0	0	2	0	0	0	0	0	0	Proteobacteria	Alphaproteobacteria	Rhodospirillales	Rhodospirillaceae	Unassigned	Unassigned
1416	2	0	0	0	1	0	0	0	1	0	0	0	0	Proteobacteria	Alphaproteobacteria	Rhodospirillales	Rhodospirillaceae	Unassigned	Unassigned
1426	2	0	0	0	2	0	0	0	0	0	0	0	0	Proteobacteria	Gammaproteobacteria	Alteromonadales	Alteromonadaceae	Unassigned	Unassigned
1427	2	0	0	0	2	0	0	0	0	0	0	0	0	Chloroflexi	S085	Unassigned	Unassigned	Unassigned	Unassigned
1430	2	0	0	1	1	0	0	0	0	0	0	0	0	Proteobacteria	Gammaproteobacteria	HOC36	Unassigned	Unassigned	Unassigned
1433	2	0	0	1	0	0	0	0	0	0	0	1	0	Proteobacteria	Gammaproteobacteria	Thiotrichales	Thiotrichaceae	Unassigned	Unassigned
1434	2	0	1	0	0	0	0	0	0	0	1	0	0	Proteobacteria	Betaproteobacteria	Methylophilales	Methylophilaceae	Methylotenera	mobilis
1441	2	0	0	2	0	0	0	0	0	0	0	0	0	Bacteroidetes	[Saprospirae]	[Saprospirales]	Saprospiraceae	Unassigned	Unassigned
1446	2	0	0	0	0	0	0	0	2	0	0	0	0	Proteobacteria	Deltaproteobacteria	Myxococcales	Unassigned	Unassigned	Unassigned

1460	2	0	0	1	0	0	0	0	0	0	0	0	1	Proteobacteria	Gammaproteobacteria	Chromatiales	Unassigned	Unassigned	Unassigned
1461	2	0	0	0	0	0	0	1	0	0	0	0	1	Bacteroidetes	Flavobacteriia	Flavobacteriales	Cryomorphaceae	Crocinitomix	Unassigned
1462	2	0	0	0	0	0	0	0	0	0	0	1	1	Actinobacteria	Nitriliruptoria	Nitriliruptorales	Nitriliruptoraceae	Unassigned	Unassigned
1465	2	0	0	1	0	0	0	0	0	0	0	0	1	Proteobacteria	Gammaproteobacteria	[Marinicellales]	[Marinicellaceae]	Marinicella	Unassigned
1472	2	0	0	0	0	0	0	0	0	0	0	0	2	Proteobacteria	Alphaproteobacteria	Sphingomonadales	Sphingomonadaceae	Unassigned	Unassigned
1478	2	0	0	0	0	0	0	2	0	0	0	0	0	Acidobacteria	Sva0725	Sva0725	Unassigned	Unassigned	Unassigned
1488	2	0	0	0	0	0	0	2	0	0	0	0	0	Proteobacteria	Gammaproteobacteria	Oceanospirillales	Saccharospirillaceae	Unassigned	Unassigned
1490	2	0	0	0	0	1	0	0	0	0	0	1	0	Proteobacteria	Deltaproteobacteria	Spirobacillales	Unassigned	Unassigned	Unassigned
1499	1	0	1	0	0	0	0	0	0	0	0	0	0	Bacteroidetes	[Saprospirae]	[Saprospirales]	Saprospiraceae	Unassigned	Unassigned
1500	2	0	2	0	0	0	0	0	0	0	0	0	0	Proteobacteria	Betaproteobacteria	Rhodocyclales	Rhodocyclaceae	Azoarcus	Unassigned
1503	2	0	0	0	0	0	0	0	0	0	0	2	0	Proteobacteria	Deltaproteobacteria	Desulfobacterales	Desulfobulbaceae	Unassigned	Unassigned
1514	1	0	1	0	0	0	0	0	0	0	0	0	0	Proteobacteria	Gammaproteobacteria	Chromatiales	Chromatiaceae	Thiorhodococcus	kakinadensis
1517	0	0	0	0	0	0	0	0	0	0	0	0	0	GN02	IIB17	Unassigned	Unassigned	Unassigned	Unassigned
1522	2	0	0	0	2	0	0	0	0	0	0	0	0	Proteobacteria	Gammaproteobacteria	Alteromonadales	Alteromonadaceae	Marinobacter	Unassigned
1525	0	0	0	0	0	0	0	0	0	0	0	0	0	Verrucomicrobia	Verrucomicrobiae	Verrucomicrobiales	Verrucomicrobiaceae	Luteolibacter	Unassigned
1528	0	0	0	0	0	0	0	0	0	0	0	0	0	Proteobacteria	Alphaproteobacteria	Rickettsiales	Rickettsiaceae	Unassigned	Unassigned
1536	2	0	0	0	0	0	0	1	0	0	0	0	1	Proteobacteria	Deltaproteobacteria	Desulfuromonadales	Desulfuromonadaceae	Unassigned	Unassigned
1540	2	0	0	0	0	0	0	0	0	0	0	2	0	Bacteroidetes	Cytophagia	Cytophagales	Flammeovirgaceae	Tunicatimonas	pelagia
1544	2	0	0	0	0	0	0	1	0	0	1	0	0	Proteobacteria	Alphaproteobacteria	Rhizobiales	Hyphomicrobiaceae	Devosia	Unassigned
1552	1	0	0	0	0	1	0	0	0	0	0	0	0	Proteobacteria	Gammaproteobacteria	Alteromonadales	Colwelliaceae	Unassigned	Unassigned
1560	2	2	0	0	0	0	0	0	0	0	0	0	0	Proteobacteria	Deltaproteobacteria	Spirobacillales	Unassigned	Unassigned	Unassigned
1571	0	0	0	0	0	0	0	0	0	0	0	0	0	Proteobacteria	Deltaproteobacteria	Bdellovibrionales	Bacteriovoracaceae	Unassigned	Unassigned
1572	0	0	0	0	0	0	0	0	0	0	0	0	0	Proteobacteria	Gammaproteobacteria	Pseudomonadales	Moraxellaceae	Perlucidibaca	Unassigned
1587	2	0	0	2	0	0	0	0	0	0	0	0	0	Proteobacteria	Gammaproteobacteria	Chromatiales	Ectothiorhodospiraceae	Unassigned	Unassigned
1596	2	0	0	1	1	0	0	0	0	0	0	0	0	TM7	TM7-1	Unassigned	Unassigned	Unassigned	Unassigned
1606	2	0	1	0	0	0	1	0	0	0	0	0	0	Proteobacteria	Gammaproteobacteria	Alteromonadales	HTCC2188	HTCC	Unassigned
1607	2	1	1	0	0	0	0	0	0	0	0	0	0	Proteobacteria	Deltaproteobacteria	Spirobacillales	Unassigned	Unassigned	Unassigned
1609	2	0	0	2	0	0	0	0	0	0	0	0	0	Bacteroidetes	[Saprospirae]	[Saprospirales]	Saprospiraceae	Unassigned	Unassigned
1614	2	0	0	1	0	0	0	0	0	0	0	1	0	Acidobacteria	Sva0725	Sva0725	Unassigned	Unassigned	Unassigned
1617	2	0	0	1	0	0	0	0	0	0	0	1	0	Proteobacteria	Deltaproteobacteria	Desulfobacterales	Desulfobulbaceae	Unassigned	Unassigned
1620	2	0	0	0	0	0	0	0	2	0	0	0	0	Bacteroidetes	Cytophagia	Cytophagales	Flammeovirgaceae	Unassigned	Unassigned
1632	2	0	0	0	0	0	1	1	0	0	0	0	0	Bacteroidetes	[Saprospirae]	[Saprospirales]	Saprospiraceae	Unassigned	Unassigned
1670	1	0	0	0	0	1	0	0	0	0	0	0	0	Proteobacteria	Deltaproteobacteria	Bdellovibrionales	Bacteriovoracaceae	Unassigned	Unassigned
1682	2	0	0	1	0	0	0	0	0	0	0	1	0	Actinobacteria	Acidimicrobiia	Acidimicrobiales	koll13	Unassigned	Unassigned
1688	2	0	0	0	0	0	0	0	0	0	2	0	0	Proteobacteria	Deltaproteobacteria	Desulfobacterales	Desulfobulbaceae	Unassigned	Unassigned
1696	2	2	0	0	0	0	0	0	0	0	0	0	0	Unassigned	Unassigned	Unassigned	Unassigned	Unassigned	Unassigned
1702	2	0	0	0	0	2	0	0	0	0	0	0	0	Proteobacteria	Gammaproteobacteria	HTCC2188	HTCC2089	Acinetobacter	radioresistens
1716	2	0	0	1	0	0	0	0	0	0	0	1	0	Proteobacteria	Gammaproteobacteria	Pseudomonadales	Pseudomonadaceae	Pseudomonas	Unassigned
1726	2	0	0	1	0	0	0	0	0	0	0	0	1	Bacteroidetes	[Saprospirae]	[Saprospirales]	Saprospiraceae	Lewinella	Unassigned
1749	2	1	1	0	0	0	0	0	0	0	0	0	0	TM7	Unassigned	Unassigned	Unassigned	Unassigned	Unassigned
1758	2	0	0	0	0	0	0	0	1	0	0	1	0	Bacteroidetes	Cytophagia	Cytophagales	Flammeovirgaceae	Flexithrix	dorotheae
1772	0	0	0	0	0	0	0	0	0	0	0	0	0	Proteobacteria	Alphaproteobacteria	BD7-3	Unassigned	Unassigned	Unassigned
1793	2	1	0	0	1	0	0	0	0	0	0	0	0	Proteobacteria	Gammaproteobacteria	Thiotrichales	Piscirickettsiaceae	Unassigned	Unassigned
1797	0	0	0	0	0	0	0	0	0	0	0	0	0	Proteobacteria	Gammaproteobacteria	Alteromonadales	OM60	Congregibacter	Unassigned
1845	2	1	0	0	0	0	1	0	0	0	0	0	0	Proteobacteria	Alphaproteobacteria	Rhodobacterales	Rhodobacteraceae	Unassigned	Unassigned

1847	2	0	0	0	2	0	0	0	0	0	0	0	0	Proteobacteria	Gammaproteobacteria	Oceanospirillales	Oceanospirillaceae	Marinomonas	Unassigned
1864	2	0	0	1	0	0	0	0	0	0	0	0	1	Bacteroidetes	[Rhodothermi]	[Rhodothermales]	Rhodothermaceae	Rubricoccus	Unassigned
1893	2	0	0	1	1	0	0	0	0	0	0	0	0	Proteobacteria	Deltaproteobacteria	NB1-j	JTB38	Unassigned	Unassigned
1895	2	0	0	1	0	0	0	0	0	0	0	0	1	Chloroflexi	Anaerolineae	SBR1031	SJA-101	Unassigned	Unassigned
683	1	0	0	0	1	0	0	0	0	0	0	0	0	Bacteroidetes	Flavobacteriia	Flavobacteriales	Flavobacteriaceae	Coralibacter	albidoflavus
688	1	0	0	0	1	0	0	0	0	0	0	0	0	Unassigned	Unassigned	Unassigned	Unassigned	Unassigned	Unassigned
689	0	0	0	0	0	0	0	0	0	0	0	0	0	Proteobacteria	Gammaproteobacteria	Chromatiales	Chromatiaceae	Rhabdochromatium	marinum
690	0	0	0	0	0	0	0	0	0	0	0	0	0	Proteobacteria	Alphaproteobacteria	Rickettsiales	Unassigned	Unassigned	Unassigned
692	0	0	0	0	0	0	0	0	0	0	0	0	0	Verrucomicrobia	Verrucomicrobiae	Verrucomicrobiales	Verrucomicrobiaceae	Unassigned	Unassigned
693	0	0	0	0	0	0	0	0	0	0	0	0	0	Proteobacteria	Alphaproteobacteria	Rhodobacterales	Rhodobacteraceae	Unassigned	Unassigned
695	1	0	0	0	0	1	0	0	0	0	0	0	0	Bacteroidetes	Cytophagia	Cytophagales	Cytophagaceae	Unassigned	Unassigned
696	1	0	0	0	0	1	0	0	0	0	0	0	0	Proteobacteria	Alphaproteobacteria	Unassigned	Unassigned	Unassigned	Unassigned
697	1	0	0	0	0	1	0	0	0	0	0	0	0	Proteobacteria	Gammaproteobacteria	Legionellales	Unassigned	Unassigned	Unassigned
698	0	0	0	0	0	0	0	0	0	0	0	0	0	Proteobacteria	Alphaproteobacteria	Rickettsiales	Unassigned	Unassigned	Unassigned
704	1	0	0	0	1	0	0	0	0	0	0	0	0	Proteobacteria	Deltaproteobacteria	Desulfovibrionales	Desulfovibrionaceae	Desulfovibrio	Unassigned
705	1	0	0	0	1	0	0	0	0	0	0	0	0	Bacteroidetes	Flavobacteriia	Flavobacteriales	Cryomorphaceae	Unassigned	Unassigned
706	1	0	0	0	1	0	0	0	0	0	0	0	0	Proteobacteria	Gammaproteobacteria	Xanthomonadales	Xanthomonadaceae	Unassigned	Unassigned
708	1	0	0	0	1	0	0	0	0	0	0	0	0	Proteobacteria	Deltaproteobacteria	Myxococcales	Unassigned	Unassigned	Unassigned
710	1	0	0	0	1	0	0	0	0	0	0	0	0	Unassigned	Unassigned	Unassigned	Unassigned	Unassigned	Unassigned
711	1	0	0	0	1	0	0	0	0	0	0	0	0	Proteobacteria	Alphaproteobacteria	Rhodospirillales	Rhodospirillaceae	Rhodospirillum	Unassigned
712	1	0	0	0	1	0	0	0	0	0	0	0	0	Proteobacteria	Alphaproteobacteria	BD7-3	Unassigned	Unassigned	Unassigned
714	1	0	0	0	1	0	0	0	0	0	0	0	0	Proteobacteria	Alphaproteobacteria	Unassigned	Unassigned	Unassigned	Unassigned
715	1	0	0	0	1	0	0	0	0	0	0	0	0	Proteobacteria	Gammaproteobacteria	Alteromonadales	Alteromonadaceae	Marinobacter	Unassigned
716	1	0	0	0	1	0	0	0	0	0	0	0	0	Actinobacteria	Acidimicrobiia	Acidimicrobiales	Unassigned	Unassigned	Unassigned
724	1	0	0	0	1	0	0	0	0	0	0	0	0	Planctomycetes	Phycisphaerae	Unassigned	Unassigned	Unassigned	Unassigned
725	1	0	0	1	0	0	0	0	0	0	0	0	0	Bacteroidetes	Flavobacteriia	Flavobacteriales	Flavobacteriaceae	Muricauda	Unassigned
727	1	0	0	1	0	0	0	0	0	0	0	0	0	Bacteroidetes	[Saprospirae]	[Saprospirales]	Chitinophagaceae	Unassigned	Unassigned
728	1	0	0	1	0	0	0	0	0	0	0	0	0	Actinobacteria	Acidimicrobiia	Acidimicrobiales	JdFBGBact	Unassigned	Unassigned
729	1	0	0	1	0	0	0	0	0	0	0	0	0	Proteobacteria	Deltaproteobacteria	Myxococcales	Nannocystaceae	Plesiocystis	Unassigned
732	1	0	0	1	0	0	0	0	0	0	0	0	0	Proteobacteria	Deltaproteobacteria	Spirobacillales	Unassigned	Unassigned	Unassigned
733	1	0	0	0	1	0	0	0	0	0	0	0	0	Proteobacteria	Alphaproteobacteria	Kiloniellales	Unassigned	Unassigned	Unassigned
734	1	0	0	1	0	0	0	0	0	0	0	0	0	Bacteroidetes	[Saprospirae]	[Saprospirales]	Saprospiraceae	Unassigned	Unassigned
735	1	0	0	1	0	0	0	0	0	0	0	0	0	Bacteroidetes	Sphingobacteriia	Sphingobacteriales	Unassigned	Unassigned	Unassigned
736	1	0	0	1	0	0	0	0	0	0	0	0	0	Proteobacteria	Deltaproteobacteria	Myxococcales	Unassigned	Unassigned	Unassigned
737	1	0	0	1	0	0	0	0	0	0	0	0	0	Bacteroidetes	[Saprospirae]	[Saprospirales]	Saprospiraceae	Unassigned	Unassigned
741	1	0	0	1	0	0	0	0	0	0	0	0	0	Proteobacteria	Deltaproteobacteria	FAC87	Unassigned	Unassigned	Unassigned
743	1	0	0	0	1	0	0	0	0	0	0	0	0	Proteobacteria	Betaproteobacteria	Burkholderiales	Comamonadaceae	Unassigned	Unassigned
745	1	0	0	0	1	0	0	0	0	0	0	0	0	Bacteroidetes	Flavobacteriia	Flavobacteriales	Flavobacteriaceae	Ulvibacter	Unassigned
747	1	0	0	0	1	0	0	0	0	0	0	0	0	Proteobacteria	Deltaproteobacteria	Myxococcales	Unassigned	Unassigned	Unassigned
748	1	0	0	0	1	0	0	0	0	0	0	0	0	Bacteroidetes	BME43	Unassigned	Unassigned	Unassigned	Unassigned
753	1	0	0	0	1	0	0	0	0	0	0	0	0	Bacteroidetes	Flavobacteriia	Flavobacteriales	Flavobacteriaceae	Unassigned	Unassigned
755	1	0	0	0	0	0	0	0	1	0	0	0	0	Proteobacteria	Deltaproteobacteria	Unassigned	Unassigned	Unassigned	Unassigned
759	1	0	0	1	0	0	0	0	0	0	0	0	0	Proteobacteria	Gammaproteobacteria	Chromatiales	Unassigned	Unassigned	Unassigned
760	1	0	0	0	0	0	0	0	1	0	0	0	0	Bacteroidetes	Flavobacteriia	Flavobacteriales	Flavobacteriaceae	Leeuwenhoekiella	marinoflava
765	0	0	0	0	0	0	0	0	0	0	0	0	0	Proteobacteria	Gammaproteobacteria	Chromatiales	Chromatiaceae	Allochromatium	vinosum

766	0	0	0	0	0	0	0	0	0	0	0	0	0 Proteobacteria	Gammaproteobacteria	Legionellales	Unassigned	Unassigned	Unassigned
767	0	0	0	0	0	0	0	0	0	0	0	0	0 Proteobacteria	Alphaproteobacteria	BD7-3	Unassigned	Unassigned	Unassigned
768	0	0	0	0	0	0	0	0	0	0	0	0	0 Bacteroidetes	Flavobacteriia	Flavobacteriales	Cryomorphaceae	Cryomorpha	Unassigned
770	0	0	0	0	0	0	0	0	0	0	0	0	0 Firmicutes	Clostridia	Clostridiales	[Tissierellaceae]	Sedimentibacter	Unassigned
771	1	0	0	1	0	0	0	0	0	0	0	0	0 Proteobacteria	Alphaproteobacteria	Rhizobiales	Cohaesibacteraceae	Unassigned	Unassigned
774	1	0	0	1	0	0	0	0	0	0	0	0	0 Proteobacteria	Alphaproteobacteria	Unassigned	Unassigned	Unassigned	Unassigned
776	1	0	0	0	0	0	1	0	0	0	0	0	0 Proteobacteria	Alphaproteobacteria	Rhodospirillales	Rhodospirillaceae	Unassigned	Unassigned
778	1	0	0	0	0	0	0	0	1	0	0	0	0 Proteobacteria	Gammaproteobacteria	Legionellales	Coxiellaceae	Unassigned	Unassigned
779	1	0	0	0	0	0	1	0	0	0	0	0	0 Bacteroidetes	Cytophagia	Cytophagales	Flammeovirgaceae	Unassigned	Unassigned
781	1	1	0	0	0	0	0	0	0	0	0	0	0 Proteobacteria	Gammaproteobacteria	Legionellales	Coxiellaceae	Unassigned	Unassigned
786	1	0	0	0	0	0	0	1	0	0	0	0	0 Proteobacteria	Gammaproteobacteria	Alteromonadales	Alteromonadaceae	Porticoccus	Unassigned
787	1	1	0	0	0	0	0	0	0	0	0	0	0 Proteobacteria	Alphaproteobacteria	Rhizobiales	Unassigned	Unassigned	Unassigned
788	1	1	0	0	0	0	0	0	0	0	0	0	0 Proteobacteria	Alphaproteobacteria	Rickettsiales	Unassigned	Unassigned	Unassigned
790	1	0	0	0	0	0	0	0	0	0	1	0	0 Proteobacteria	Deltaproteobacteria	Myxococcales	Unassigned	Unassigned	Unassigned
793	1	0	0	0	0	0	0	0	0	1	0	0	0 Bacteroidetes	BME43	Unassigned	Unassigned	Unassigned	Unassigned
794	1	0	0	0	0	0	0	0	0	1	0	0	0 Proteobacteria	Gammaproteobacteria	Legionellales	Coxiellaceae	Coxiella	Unassigned
795	1	0	0	0	0	0	0	1	0	0	0	0	0 Nitrospirae	Nitrospira	Nitrospirales	Nitrospiraceae	Unassigned	Unassigned
796	1	0	0	0	0	0	0	1	0	0	0	0	0 Proteobacteria	Deltaproteobacteria	Desulfuromonadales	Pelobacteraceae	Unassigned	Unassigned
798	1	1	0	0	0	0	0	0	0	0	0	0	0 Verrucomicrobia	Opitutae	[Cerasicoccales]	[Cerasicoccaceae]	Unassigned	Unassigned
799	1	1	0	0	0	0	0	0	0	0	0	0	0 Verrucomicrobia	Verrucomicrobiae	Verrucomicrobiales	Verrucomicrobiaceae	MSBL3	Unassigned
802	1	1	0	0	0	0	0	0	0	0	0	0	0 Proteobacteria	Deltaproteobacteria	Bdellovibrionales	Bacteriovoracaceae	Bacteriovorax	Unassigned
806	1	0	0	0	0	0	0	1	0	0	0	0	0 Chloroflexi	Anaerolineae	SBR1031	A4b	Unassigned	Unassigned
807	1	0	0	0	0	0	0	1	0	0	0	0	0 Chloroflexi	Anaerolineae	Anaerolineales	Anaerolinaceae	T78	Unassigned
809	1	0	0	0	0	0	0	1	0	0	0	0	0 Firmicutes	Clostridia	Clostridiales	Peptostreptococcaceae	Clostridium	sticklandii
810	1	0	0	0	0	1	0	0	0	0	0	0	0 Proteobacteria	Gammaproteobacteria	Unassigned	Unassigned	Unassigned	Unassigned
811	1	0	0	0	0	0	0	1	0	0	0	0	0 WS3	PRR-12	GN03	Unassigned	Unassigned	Unassigned
812	1	0	0	0	0	1	0	0	0	0	0	0	0 Tenericutes	Mollicutes	Unassigned	Unassigned	Unassigned	Unassigned
816	1	1	0	0	0	0	0	0	0	0	0	0	0 Proteobacteria	Alphaproteobacteria	Unassigned	Unassigned	Unassigned	Unassigned
818	1	1	0	0	0	0	0	0	0	0	0	0	0 Proteobacteria	Alphaproteobacteria	BD7-3	Unassigned	Unassigned	Unassigned
820	1	1	0	0	0	0	0	0	0	0	0	0	0 Proteobacteria	Gammaproteobacteria	Legionellales	Coxiellaceae	Unassigned	Unassigned
821	1	1	0	0	0	0	0	0	0	0	0	0	0 Proteobacteria	Alphaproteobacteria	Unassigned	Unassigned	Unassigned	Unassigned
822	1	0	0	0	0	1	0	0	0	0	0	0	0 Firmicutes	Clostridia	Clostridiales	Unassigned	Unassigned	Unassigned
823	1	0	0	0	0	1	0	0	0	0	0	0	0 Proteobacteria	Gammaproteobacteria	Unassigned	Unassigned	Unassigned	Unassigned
824	1	0	0	0	0	1	0	0	0	0	0	0	0 Verrucomicrobia	Verrucomicrobiae	Verrucomicrobiales	Verrucomicrobiaceae	Verrucomicrobium	Unassigned
826	0	0	0	0	0	0	0	0	0	0	0	0	0 Proteobacteria	Gammaproteobacteria	Legionellales	Unassigned	Unassigned	Unassigned
830	0	0	0	0	0	0	0	0	0	0	0	0	0 OD1	ZB2	Unassigned	Unassigned	Unassigned	Unassigned
831	0	0	0	0	0	0	0	0	0	0	0	0	0 Proteobacteria	Alphaproteobacteria	Unassigned	Unassigned	Unassigned	Unassigned
834	0	0	0	0	0	0	0	0	0	0	0	0	0 TM6	Unassigned	Unassigned	Unassigned	Unassigned	Unassigned
835	0	0	0	0	0	0	0	0	0	0	0	0	0 Proteobacteria	Deltaproteobacteria	GMD14H09	Unassigned	Unassigned	Unassigned
838	1	0	0	0	0	0	0	0	0	1	0	0	0 Proteobacteria	Alphaproteobacteria	Unassigned	Unassigned	Unassigned	Unassigned
840	1	0	0	0	0	0	0	0	0	1	0	0	0 Verrucomicrobia	Verrucomicrobiae	Verrucomicrobiales	Verrucomicrobiaceae	Unassigned	Unassigned
842	1	0	0	0	0	0	0	0	0	1	0	0	0 Proteobacteria	Alphaproteobacteria	Rickettsiales	Rickettsiaceae	Unassigned	Unassigned
846	1	1	0	0	0	0	0	0	0	0	0	0	0 Proteobacteria	Gammaproteobacteria	Oceanospirillales	Halomonadaceae	CandidatusPortiera	Unassigned
847	1	1	0	0	0	0	0	0	0	0	0	0	0 Proteobacteria	Gammaproteobacteria	Unassigned	Unassigned	Unassigned	Unassigned
848	1	1	0	0	0	0	0	0	0	0	0	0	0 Proteobacteria	Alphaproteobacteria	Kiloniellales	Unassigned	Unassigned	Unassigned

849	1	1	0	0	0	0	0	0	0	0	0	0	0	Proteobacteria	Gammaproteobacteria	Legionellales	Unassigned	Unassigned	Unassigned
850	1	0	0	0	0	0	0	1	0	0	0	0	0	Bacteroidetes	Cytophagia	Cytophagales	Flammeovirgaceae	Unassigned	Unassigned
852	1	0	0	0	0	0	0	1	0	0	0	0	0	Proteobacteria	Gammaproteobacteria	Thiotrichales	Thiotrichaceae	Unassigned	Unassigned
854	1	0	0	0	0	0	0	1	0	0	0	0	0	Bacteroidetes	[Rhodothermi]	[Rhodothermales]	Rhodothermaceae	Unassigned	Unassigned
856	1	1	0	0	0	0	0	0	0	0	0	0	0	Proteobacteria	Deltaproteobacteria	GMD14H09	Unassigned	Unassigned	Unassigned
858	1	0	0	0	0	0	0	1	0	0	0	0	0	Proteobacteria	Betaproteobacteria	Burkholderiales	Comamonadaceae	Leptothrix	Unassigned
861	1	0	0	0	0	0	0	1	0	0	0	0	0	Proteobacteria	Betaproteobacteria	Hydrogenophilales	Hydrogenophilaceae	Thiobacillus	Unassigned
862	1	0	0	0	0	0	0	1	0	0	0	0	0	Bacteroidetes	Bacteroidia	Bacteroidales	SB-1	Unassigned	Unassigned
863	1	0	0	0	0	0	0	1	0	0	0	0	0	Proteobacteria	Deltaproteobacteria	Syntrophobacterales	Syntrophobacteraceae	Unassigned	Unassigned
864	1	0	0	0	0	0	0	1	0	0	0	0	0	Actinobacteria	Acidimicrobiia	Acidimicrobiales	Microthrixaceae	Unassigned	Unassigned
865	1	0	0	0	0	1	0	0	0	0	0	0	0	Bacteroidetes	Bacteroidia	Bacteroidales	Unassigned	Unassigned	Unassigned
867	1	0	0	0	0	0	0	1	0	0	0	0	0	Cyanobacteria	4C0d-2	Unassigned	Unassigned	Unassigned	Unassigned
868	1	0	0	0	0	0	0	1	0	0	0	0	0	Gemmatimonadete:	Gemm-2	Unassigned	Unassigned	Unassigned	Unassigned
869	1	0	0	0	0	0	0	1	0	0	0	0	0	Proteobacteria	Gammaproteobacteria	HTCC2188	HTCC2089	Unassigned	Unassigned
871	1	0	0	0	0	0	0	1	0	0	0	0	0	Bacteroidetes	Flavobacteriia	Flavobacteriales	Cryomorphaceae	Unassigned	Unassigned
872	1	0	0	0	0	1	0	0	0	0	0	0	0	Actinobacteria	Acidimicrobiia	Acidimicrobiales	JdFBGBact	Unassigned	Unassigned
876	1	0	0	0	1	0	0	0	0	0	0	0	0	Proteobacteria	Deltaproteobacteria	Unassigned	Unassigned	Unassigned	Unassigned
877	1	0	0	0	0	0	0	0	0	0	0	0	1	Bacteroidetes	Flavobacteriia	Flavobacteriales	Flavobacteriaceae	Polaribacter	Unassigned
878	1	0	0	0	0	0	0	0	0	0	0	0	1	Proteobacteria	Deltaproteobacteria	Myxococcales	Unassigned	Unassigned	Unassigned
879	1	0	0	0	0	0	0	0	0	0	0	0	1	Proteobacteria	Deltaproteobacteria	Bdellovibrionales	Bacteriovoracaceae	Unassigned	Unassigned
882	1	0	0	0	0	0	0	0	0	0	0	0	1	Gemmatimonadete:	Gemm-2	Unassigned	Unassigned	Unassigned	Unassigned
883	1	0	0	0	0	0	0	0	0	0	0	0	1	Proteobacteria	Alphaproteobacteria	Rhodobacterales	Hyphomonadaceae	Robiginitomaculum	antarcticum
884	1	0	0	0	0	0	0	0	0	0	0	0	1	Proteobacteria	Gammaproteobacteria	Oceanospirillales	Oceanospirillaceae	Unassigned	Unassigned
886	1	0	0	0	0	0	0	0	0	0	0	0	1	Proteobacteria	Deltaproteobacteria	GMD14H09	Unassigned	Unassigned	Unassigned
887	1	0	0	0	0	0	0	0	0	0	0	0	1	Chloroflexi	Anaerolineae	CFB-26	Unassigned	Unassigned	Unassigned
890	1	0	0	0	0	0	1	0	0	0	0	0	0	Proteobacteria	Deltaproteobacteria	Spirobacillales	Unassigned	Unassigned	Unassigned
893	1	0	0	0	0	0	1	0	0	0	0	0	0	Bacteroidetes	[Saprospirae]	[Saprospirales]	Chitinophagaceae	Unassigned	Unassigned
895	1	0	0	0	0	0	1	0	0	0	0	0	0	Proteobacteria	Deltaproteobacteria	Bdellovibrionales	Bacteriovoracaceae	Bacteriovorax	Unassigned
896	1	0	0	0	0	0	0	0	0	0	0	0	1	Proteobacteria	Deltaproteobacteria	Sva0853	JTB36	Unassigned	Unassigned
899	1	1	0	0	0	0	0	0	0	0	0	0	0	Proteobacteria	Deltaproteobacteria	Myxococcales	Nannocystaceae	Plesiocystis	Unassigned
900	1	0	0	0	0	0	0	1	0	0	0	0	0	Bacteroidetes	Bacteroidia	Bacteroidales	SB-1	Unassigned	Unassigned
901	1	1	0	0	0	0	0	0	0	0	0	0	0	Proteobacteria	Alphaproteobacteria	Unassigned	Unassigned	Unassigned	Unassigned
902	1	1	0	0	0	0	0	0	0	0	0	0	0	Proteobacteria	Gammaproteobacteria	Vibrionales	Vibrionaceae	Photobacterium	Unassigned
903	1	1	0	0	0	0	0	0	0	0	0	0	0	Proteobacteria	Unassigned	Unassigned	Unassigned	Unassigned	Unassigned
904	1	1	0	0	0	0	0	0	0	0	0	0	0	Proteobacteria	Gammaproteobacteria	Chromatiales	Unassigned	Unassigned	Unassigned
905	1	1	0	0	0	0	0	0	0	0	0	0	0	Proteobacteria	Alphaproteobacteria	Rhodobacterales	Rhodobacteraceae	Unassigned	Unassigned
907	1	1	0	0	0	0	0	0	0	0	0	0	0	Bacteroidetes	Flavobacteriia	Flavobacteriales	Flavobacteriaceae	Unassigned	Unassigned
908	1	1	0	0	0	0	0	0	0	0	0	0	0	Proteobacteria	Gammaproteobacteria	Legionellales	Coxiellaceae	Aquicella	Unassigned
910	1	1	0	0	0	0	0	0	0	0	0	0	0	Proteobacteria	Gammaproteobacteria	Pseudomonadales	Moraxellaceae	Acinetobacter	venetianus
912	1	0	0	0	0	0	0	0	0	0	0	0	1	Proteobacteria	Deltaproteobacteria	Myxococcales	Unassigned	Unassigned	Unassigned
915	1	0	0	0	0	0	0	1	0	0	0	0	0	Proteobacteria	Deltaproteobacteria	PB19	Unassigned	Unassigned	Unassigned
916	1	0	0	0	0	0	0	1	0	0	0	0	0	Proteobacteria	Alphaproteobacteria	Rhizobiales	Rhodobiaceae	Afifella	Unassigned
917	1	0	0	0	0	0	0	1	0	0	0	0	0	Proteobacteria	Gammaproteobacteria	Chromatiales	Unassigned	Unassigned	Unassigned
918	1	0	0	0	0	0	0	1	0	0	0	0	0	Proteobacteria	Gammaproteobacteria	Alteromonadales	Unassigned	Unassigned	Unassigned
920	1	0	0	1	0	0	0	0	0	0	0	0	0	Proteobacteria	Deltaproteobacteria	NB1-j	Unassigned	Unassigned	Unassigned

922	1	0	0	1	0	0	0	0	0	0	0	0	0	Chloroflexi	TK17	mle1-48	Unassigned	Unassigned	Unassigned
923	1	0	0	1	0	0	0	0	0	0	0	0	0	Proteobacteria	Gammaproteobacteria	Salinisphaerales	Salinisphaeraceae	Unassigned	Unassigned
925	1	0	0	1	0	0	0	0	0	0	0	0	0	Chloroflexi	Anaerolineae	SBR1031	A4b	Unassigned	Unassigned
926	1	0	0	1	0	0	0	0	0	0	0	0	0	Proteobacteria	Gammaproteobacteria	Chromatiales	Unassigned	Unassigned	Unassigned
927	1	0	0	1	0	0	0	0	0	0	0	0	0	Gemmatimonadete	Gemm-2	Unassigned	Unassigned	Unassigned	Unassigned
929	1	0	0	1	0	0	0	0	0	0	0	0	0	Proteobacteria	Gammaproteobacteria	Pseudomonadales	Moraxellaceae	Psychrobacter	Unassigned
930	1	0	0	1	0	0	0	0	0	0	0	0	0	Gemmatimonadete	Gemm-2	Unassigned	Unassigned	Unassigned	Unassigned
931	1	0	0	0	1	0	0	0	0	0	0	0	0	Proteobacteria	Gammaproteobacteria	Legionellales	Coxiellaceae	Unassigned	Unassigned
932	1	0	0	1	0	0	0	0	0	0	0	0	0	Bacteroidetes	[Saprospirae]	[Saprospirales]	Saprospiraceae	Unassigned	Unassigned
934	1	0	0	1	0	0	0	0	0	0	0	0	0	Proteobacteria	Gammaproteobacteria	Alteromonadales	Alteromonadaceae	Unassigned	Unassigned
937	0	0	0	0	0	0	0	0	0	0	0	0	0	Proteobacteria	Gammaproteobacteria	Pseudomonadales	Pseudomonadaceae	Pseudomonas	veronii
938	0	0	0	0	0	0	0	0	0	0	0	0	0	Proteobacteria	Gammaproteobacteria	Oceanospirillales	Oceanospirillaceae	Oleispira	Unassigned
939	0	0	0	0	0	0	0	0	0	0	0	0	0	Proteobacteria	Alphaproteobacteria	Rickettsiales	Unassigned	Unassigned	Unassigned
940	0	0	0	0	0	0	0	0	0	0	0	0	0	Fusobacteria	Fusobacteriia	Fusobacteriales	Unassigned	Unassigned	Unassigned
941	1	0	0	1	0	0	0	0	0	0	0	0	0	Proteobacteria	Deltaproteobacteria	Myxococcales	Haliangiaceae	Unassigned	Unassigned
942	1	0	0	1	0	0	0	0	0	0	0	0	0	Bacteroidetes	[Saprospirae]	[Saprospirales]	Saprospiraceae	Unassigned	Unassigned
943	1	0	0	1	0	0	0	0	0	0	0	0	0	Proteobacteria	Gammaproteobacteria	Alteromonadales	Alteromonadaceae	Glaciecola	Unassigned
946	1	0	0	1	0	0	0	0	0	0	0	0	0	Chlorobi	Ignavibacteria	Ignavibacteriales	Ignavibacteriaceae	Unassigned	Unassigned
947	1	0	0	1	0	0	0	0	0	0	0	0	0	Unassigned	Unassigned	Unassigned	Unassigned	Unassigned	Unassigned
948	1	0	0	1	0	0	0	0	0	0	0	0	0	Proteobacteria	Gammaproteobacteria	Chromatiales	Unassigned	Unassigned	Unassigned
950	1	0	0	1	0	0	0	0	0	0	0	0	0	Proteobacteria	Betaproteobacteria	Unassigned	Unassigned	Unassigned	Unassigned
951	0	0	0	0	0	0	0	0	0	0	0	0	0	Proteobacteria	Gammaproteobacteria	Legionellales	Coxiellaceae	Aquicella	Unassigned
953	0	0	0	0	0	0	0	0	0	0	0	0	0	SR1	Unassigned	Unassigned	Unassigned	Unassigned	Unassigned
954	0	0	0	0	0	0	0	0	0	0	0	0	0	Proteobacteria	Gammaproteobacteria	Legionellales	Unassigned	Unassigned	Unassigned
956	0	0	0	0	0	0	0	0	0	0	0	0	0	Firmicutes	Clostridia	Clostridiales	Lachnospiraceae	Clostridium	fimetarium
958	1	0	0	0	0	0	1	0	0	0	0	0	0	Proteobacteria	Deltaproteobacteria	Unassigned	Unassigned	Unassigned	Unassigned
960	0	0	0	0	0	0	0	0	0	0	0	0	0	Proteobacteria	Deltaproteobacteria	Desulfobacterales	Unassigned	Unassigned	Unassigned
961	0	0	0	0	0	0	0	0	0	0	0	0	0	Proteobacteria	Alphaproteobacteria	Rhodobacterales	Hyphomonadaceae	Unassigned	Unassigned
963	1	0	0	0	1	0	0	0	0	0	0	0	0	Proteobacteria	Gammaproteobacteria	Legionellales	Coxiellaceae	Unassigned	Unassigned
964	1	0	0	0	1	0	0	0	0	0	0	0	0	Proteobacteria	Gammaproteobacteria	Alteromonadales	HTCC2188	HTCC	Unassigned
965	1	0	0	0	1	0	0	0	0	0	0	0	0	Proteobacteria	Gammaproteobacteria	Alteromonadales	Alteromonadaceae	Unassigned	Unassigned
968	1	0	0	0	0	0	0	0	0	0	0	1	0	WS3	PRR-12	GN03	Unassigned	Unassigned	Unassigned
972	1	0	0	0	0	0	0	0	0	0	0	1	0	Proteobacteria	Gammaproteobacteria	Legionellales	Unassigned	Unassigned	Unassigned
973	1	0	0	0	0	0	0	0	0	0	0	1	0	Unassigned	Unassigned	Unassigned	Unassigned	Unassigned	Unassigned
976	1	0	1	0	0	0	0	0	0	0	0	0	0	Proteobacteria	Alphaproteobacteria	Kiloniellales	Unassigned	Unassigned	Unassigned
978	1	0	0	0	0	0	0	0	0	0	0	1	0	Bacteroidetes	Cytophagia	Cytophagales	Flammeovirgaceae	Unassigned	Unassigned
979	1	0	0	0	0	0	0	0	0	0	0	1	0	Proteobacteria	Gammaproteobacteria	Chromatiales	Ectothiorhodospiraceae	Unassigned	Unassigned
980	1	0	0	0	0	0	0	0	0	0	0	1	0	Bacteroidetes	[Saprospirae]	[Saprospirales]	Saprospiraceae	Unassigned	Unassigned
982	0	0	0	0	0	0	0	0	0	0	0	0	0	Proteobacteria	Gammaproteobacteria	Legionellales	Unassigned	Unassigned	Unassigned
983	0	0	0	0	0	0	0	0	0	0	0	0	0	Proteobacteria	Deltaproteobacteria	Bdellovibrionales	Bacteriovoracaceae	Unassigned	Unassigned
989	1	0	0	1	0	0	0	0	0	0	0	0	0	Proteobacteria	Gammaproteobacteria	Chromatiales	Chromatiaceae	Halochromatium	Unassigned
990	1	0	0	0	0	0	0	0	0	0	0	1	0	Proteobacteria	Alphaproteobacteria	Rhodobacterales	Rhodobacteraceae	Maribius	salinus
991	1	0	0	0	0	0	0	0	0	0	0	1	0	Proteobacteria	Gammaproteobacteria	Legionellales	Legionellaceae	Unassigned	Unassigned
992	1	0	0	0	0	0	0	0	0	0	0	1	0	Proteobacteria	Deltaproteobacteria	Myxococcales	Polyangiaceae	Unassigned	Unassigned
993	1	0	0	1	0	0	0	0	0	0	0	0	0	Acidobacteria	Sva0725	Sva0725	Unassigned	Unassigned	Unassigned

995	0	0	0	0	0	0	0	0	0	0	0	0	Proteobacteria	Deltaproteobacteria	Bdellovibrionales	Bacteriovoracaceae	Bacteriovorax	Unassigned
996	0	0	0	0	0	0	0	0	0	0	0	0	Proteobacteria	Gammaproteobacteria	HTCC2188	Unassigned	Unassigned	Unassigned
998	1	0	0	0	0	0	0	0	0	0	0	1	Proteobacteria	Alphaproteobacteria	Rhodospirillales	Rhodospirillaceae	Unassigned	Unassigned
999	1	0	0	0	0	0	0	0	0	0	0	1	Proteobacteria	Gammaproteobacteria	Vibrionales	Vibrionaceae	Vibrio	Unassigned
1002	1	0	0	0	0	0	0	0	0	0	0	1	Proteobacteria	Zetaproteobacteria	Mariprofundales	Mariprofundaceae	Mariprofundus	Unassigned
1003	1	0	0	0	0	0	0	0	0	0	0	1	Proteobacteria	Gammaproteobacteria	Alteromonadales	Alteromonadaceae	CandidatusEndobugula	Unassigned
1004	1	0	0	0	0	0	0	0	0	0	0	1	Proteobacteria	Deltaproteobacteria	Myxococcales	Cystobacterineae	Unassigned	Unassigned
1007	1	0	0	0	0	0	1	0	0	0	0	0	Proteobacteria	Gammaproteobacteria	Oceanospirillales	Halomonadaceae	CandidatusPortiera	Unassigned
1010	1	0	1	0	0	0	0	0	0	0	0	0	Proteobacteria	Deltaproteobacteria	Myxococcales	Haliangiaceae	Unassigned	Unassigned
1012	1	0	0	1	0	0	0	0	0	0	0	0	Actinobacteria	Acidimicrobiia	Acidimicrobiales	Unassigned	Unassigned	Unassigned
1015	1	0	0	0	0	0	0	0	0	0	1	0	Proteobacteria	Deltaproteobacteria	Desulfobacterales	Desulfobulbaceae	Unassigned	Unassigned
1016	1	0	1	0	0	0	0	0	0	0	0	0	Bacteroidetes	[Saprospirae]	[Saprospirales]	Saprospiraceae	Unassigned	Unassigned
1017	1	0	0	0	0	0	0	0	0	0	1	0	Proteobacteria	Gammaproteobacteria	Alteromonadales	Alteromonadaceae	Glaciecola	lipolytica
1018	1	0	0	0	0	0	0	0	0	0	1	0	Acidobacteria	Unassigned	Unassigned	Unassigned	Unassigned	Unassigned
1020	0	0	0	0	0	0	0	0	0	0	0	0	Bacteroidetes	BME43	Unassigned	Unassigned	Unassigned	Unassigned
1022	1	0	0	0	0	0	0	1	0	0	0	0	Chloroflexi	Anaerolineae	GCA004	Unassigned	Unassigned	Unassigned
1024	0	0	0	0	0	0	0	0	0	0	0	0	Proteobacteria	Alphaproteobacteria	BD7-3	Unassigned	Unassigned	Unassigned
1025	0	0	0	0	0	0	0	0	0	0	0	0	Proteobacteria	Gammaproteobacteria	Oceanospirillales	Oceanospirillaceae	Marinobacterium	Unassigned
1027	1	0	0	0	0	1	0	0	0	0	0	0	Bacteroidetes	Cytophagia	Cytophagales	Flammeovirgaceae	Unassigned	Unassigned
1029	1	0	0	0	0	0	0	1	0	0	0	0	[Thermi]	Deinococci	Deinococcales	Trueperaceae	B-42	Unassigned
1030	1	0	0	0	0	0	0	1	0	0	0	0	Bacteroidetes	[Saprospirae]	[Saprospirales]	Saprospiraceae	Unassigned	Unassigned
1031	1	0	0	0	0	0	0	1	0	0	0	0	TM6	SJA-4	Unassigned	Unassigned	Unassigned	Unassigned
1032	1	0	0	0	0	1	0	0	0	0	0	0	Proteobacteria	Gammaproteobacteria	Legionellales	Coxiellaceae	Unassigned	Unassigned
1033	1	0	0	0	0	0	0	1	0	0	0	0	Proteobacteria	Gammaproteobacteria	Legionellales	Coxiellaceae	Unassigned	Unassigned
1034	1	0	0	0	0	0	0	1	0	0	0	0	WS3	PRR-12	GN03	Unassigned	Unassigned	Unassigned
1035	1	0	0	0	0	0	0	1	0	0	0	0	Proteobacteria	Deltaproteobacteria	Myxococcales	Haliangiaceae	Haliangium	Unassigned
1036	1	0	0	0	0	0	0	1	0	0	0	0	Bacteroidetes	Cytophagia	Cytophagales	Flammeovirgaceae	Unassigned	Unassigned
1037	1	0	0	0	0	1	0	0	0	0	0	0	Bacteroidetes	Cytophagia	Cytophagales	Flammeovirgaceae	Unassigned	Unassigned
1038	1	0	0	0	0	0	0	1	0	0	0	0	Acidobacteria	Solibacteres	Solibacterales	PAUC26f	Unassigned	Unassigned
1041	1	0	0	0	0	1	0	0	0	0	0	0	Proteobacteria	Alphaproteobacteria	Rickettsiales	Unassigned	Unassigned	Unassigned
1046	0	0	0	0	0	0	0	0	0	0	0	0	Proteobacteria	Deltaproteobacteria	Unassigned	Unassigned	Unassigned	Unassigned
1047	0	0	0	0	0	0	0	0	0	0	0	0	Proteobacteria	Gammaproteobacteria	Legionellales	Francisellaceae	Francisella	Unassigned
1048	0	0	0	0	0	0	0	0	0	0	0	0	GN02	BD1-5	Unassigned	Unassigned	Unassigned	Unassigned
1051	1	0	0	0	0	1	0	0	0	0	0	0	Proteobacteria	Deltaproteobacteria	Bdellovibrionales	Bdellovibrionaceae	Bdellovibrio	Unassigned
1054	0	0	0	0	0	0	0	0	0	0	0	0	Bacteroidetes	Flavobacteriia	Flavobacteriales	Cryomorphaceae	Unassigned	Unassigned
1055	1	0	0	1	0	0	0	0	0	0	0	0	Proteobacteria	Gammaproteobacteria	Unassigned	Unassigned	Unassigned	Unassigned
1057	1	0	0	1	0	0	0	0	0	0	0	0	Verrucomicrobia	[Pedosphaerae]	[Pedosphaerales]	Unassigned	Unassigned	Unassigned
1059	1	0	0	1	0	0	0	0	0	0	0	0	Gemmatimonadete:	Gemm-2	Unassigned	Unassigned	Unassigned	Unassigned
1061	1	0	0	1	0	0	0	0	0	0	0	0	Cyanobacteria	Oscillatoriophycideae	Oscillatoriales	Phormidiaceae	Phormidium	Unassigned
1063	0	0	0	0	0	0	0	0	0	0	0	0	Proteobacteria	Gammaproteobacteria	Alteromonadales	Alteromonadaceae	Unassigned	Unassigned
1067	1	0	0	1	0	0	0	0	0	0	0	0	Proteobacteria	Alphaproteobacteria	BD7-3	Unassigned	Unassigned	Unassigned
1068	1	0	0	0	0	1	0	0	0	0	0	0	Proteobacteria	Deltaproteobacteria	Unassigned	Unassigned	Unassigned	Unassigned
1069	1	0	0	0	0	1	0	0	0	0	0	0	Proteobacteria	Alphaproteobacteria	Unassigned	Unassigned	Unassigned	Unassigned
1071	1	0	0	0	0	0	0	0	0	0	0	1	0	Proteobacteria	Deltaproteobacteria	Myxococcales	Cystobacterineae	Unassigned	Unassigned
1073	1	0	0	0	0	0	0	0	0	0	0	1	0	Proteobacteria	Deltaproteobacteria	Myxococcales	Nannocystaceae	Plesiocystis	Unassigned

1075	1	0	0	1	0	0	0	0	0	0	0	0	Proteobacteria	Betaproteobacteria	Burkholderiales	Oxalobacteraceae	Oxalobacter	Unassigned
1076	1	0	0	1	0	0	0	0	0	0	0	0	Proteobacteria	Gammaproteobacteria	Alteromonadales	Alteromonadaceae	Unassigned	Unassigned
1077	1	0	0	1	0	0	0	0	0	0	0	0	GN02	3BR-5F	Unassigned	Unassigned	Unassigned	Unassigned
1081	1	0	0	0	0	0	0	0	1	0	0	0	Planctomycetes	Phycisphaerae	Phycisphaerales	Unassigned	Unassigned	Unassigned
1082	1	0	0	1	0	0	0	0	0	0	0	0	Cyanobacteria	Oscillatoriophycideae	Chroococcales	Unassigned	Unassigned	Unassigned
1083	1	0	0	1	0	0	0	0	0	0	0	0	Bacteroidetes	Cytophagia	Cytophagales	[Amoebophilaceae]	Ucs1325	Unassigned
1084	1	0	0	1	0	0	0	0	0	0	0	0	Tenericutes	Mollicutes	Anaeroplasmatales	Anaeroplasmataceae	Asteroleplasma	Unassigned
1086	1	0	0	1	0	0	0	0	0	0	0	0	Bacteroidetes	Flavobacteriia	Flavobacteriales	Flavobacteriaceae	Pseudozobellia	thermophila
1087	1	0	0	1	0	0	0	0	0	0	0	0	Gemmatimonadete:	Gemm-1	Unassigned	Unassigned	Unassigned	Unassigned
1089	1	0	0	0	0	0	1	0	0	0	0	0	Cyanobacteria	Synechococcophycideae	Pseudanabaenales	Pseudanabaenaceae	Unassigned	Unassigned
1091	1	0	0	0	0	0	0	0	0	0	0	1	Proteobacteria	Deltaproteobacteria	Myxococcales	OM27	Unassigned	Unassigned
1094	1	0	0	0	0	0	1	0	0	0	0	0	Bacteroidetes	[Saprospirae]	[Saprospirales]	Saprospiraceae	Saprospira	Unassigned
1095	1	0	0	0	0	0	0	0	0	0	0	1	Spirochaetes	Spirochaetes	Spirochaetales	Spirochaetaceae	Spirochaeta	Unassigned
1097	1	0	0	0	0	0	1	0	0	0	0	0	Bacteroidetes	[Saprospirae]	[Saprospirales]	Saprospiraceae	Unassigned	Unassigned
1099	0	0	0	0	0	0	0	0	0	0	0	0	Cyanobacteria	4C0d-2	MLE1-12	Unassigned	Unassigned	Unassigned
1100	0	0	0	0	0	0	0	0	0	0	0	0	Proteobacteria	TA18	PHOS-HD29	Unassigned	Unassigned	Unassigned
1101	0	0	0	0	0	0	0	0	0	0	0	0	Proteobacteria	Gammaproteobacteria	Oceanospirillales	Oceanospirillaceae	Oceanospirillum	Unassigned
1104	0	0	0	0	0	0	0	0	0	0	0	0	Proteobacteria	Gammaproteobacteria	Xanthomonadales	Xanthomonadaceae	Wohlfahrtiimonas	Unassigned
1105	1	0	0	0	0	0	0	0	0	0	1	0	Proteobacteria	Gammaproteobacteria	Oceanospirillales	Oceanospirillaceae	Unassigned	Unassigned
1106	1	0	0	0	0	0	0	0	0	0	0	1	Chloroflexi	Anaerolineae	SHA-20	Unassigned	Unassigned	Unassigned
1108	1	0	0	0	0	0	0	0	0	0	0	1	WS3	PRR-12	GN03	Unassigned	Unassigned	Unassigned
1109	1	0	0	0	0	0	0	0	0	0	0	1	Proteobacteria	Deltaproteobacteria	Spirobacillales	Unassigned	Unassigned	Unassigned
1111	1	0	0	0	0	0	0	0	0	0	0	1	Proteobacteria	Deltaproteobacteria	Myxococcales	Nannocystaceae	Plesiocystis	Unassigned
1114	1	1	0	0	0	0	0	0	0	0	0	0	Proteobacteria	Deltaproteobacteria	Spirobacillales	Unassigned	Unassigned	Unassigned
1115	1	1	0	0	0	0	0	0	0	0	0	0	Proteobacteria	Alphaproteobacteria	Unassigned	Unassigned	Unassigned	Unassigned
1116	1	1	0	0	0	0	0	0	0	0	0	0	Proteobacteria	Gammaproteobacteria	HTCC2188	HTCC2089	Unassigned	Unassigned
1117	1	0	0	1	0	0	0	0	0	0	0	0	Bacteroidetes	Flavobacteriia	Flavobacteriales	Flavobacteriaceae	Aquimarina	brevivitae
1120	1	0	0	1	0	0	0	0	0	0	0	0	Proteobacteria	Gammaproteobacteria	[Marinicellales]	[Marinicellaceae]	Unassigned	Unassigned
1121	1	0	0	1	0	0	0	0	0	0	0	0	TM6	SJA-4	Unassigned	Unassigned	Unassigned	Unassigned
1123	1	0	0	1	0	0	0	0	0	0	0	0	Acidobacteria	Acidobacteria-6	CCU21	Unassigned	Unassigned	Unassigned
1124	1	0	0	1	0	0	0	0	0	0	0	0	Proteobacteria	Deltaproteobacteria	NB1-j	NB1-i	Unassigned	Unassigned
1125	1	0	0	1	0	0	0	0	0	0	0	0	Proteobacteria	Deltaproteobacteria	Myxococcales	Polyangiaceae	Unassigned	Unassigned
1127	1	0	0	1	0	0	0	0	0	0	0	0	Proteobacteria	Deltaproteobacteria	Myxococcales	Cystobacterineae	Unassigned	Unassigned
1129	1	0	0	1	0	0	0	0	0	0	0	0	Proteobacteria	Alphaproteobacteria	Rhizobiales	Hyphomicrobiaceae	Unassigned	Unassigned
1130	1	0	0	1	0	0	0	0	0	0	0	0	Proteobacteria	Gammaproteobacteria	Alteromonadales	HTCC2188	HTCC	Unassigned
1132	1	0	0	1	0	0	0	0	0	0	0	0	Proteobacteria	Deltaproteobacteria	Myxococcales	Unassigned	Unassigned	Unassigned
1133	1	0	0	1	0	0	0	0	0	0	0	0	WS6	B142	Unassigned	Unassigned	Unassigned	Unassigned
1134	1	0	0	1	0	0	0	0	0	0	0	0	Proteobacteria	Gammaproteobacteria	Alteromonadales	Ferrimonadaceae	Ferrimonas	Unassigned
1136	1	0	0	0	0	0	0	0	0	1	0	0	Unassigned	Unassigned	Unassigned	Unassigned	Unassigned	Unassigned
1138	1	0	0	0	0	0	0	0	0	0	1	0	Firmicutes	Clostridia	Clostridiales	[Acidaminobacteraceae]	Fusibacter	Unassigned
1139	1	0	0	0	0	0	0	0	0	0	1	0	Acidobacteria	Holophagae	Holophagales	Unassigned	Unassigned	Unassigned
1142	1	0	0	1	0	0	0	0	0	0	0	0	GN02	BD1-5	Unassigned	Unassigned	Unassigned	Unassigned
1143	1	0	0	1	0	0	0	0	0	0	0	0	Proteobacteria	Betaproteobacteria	Unassigned	Unassigned	Unassigned	Unassigned
1144	1	0	0	1	0	0	0	0	0	0	0	0	Bacteroidetes	[Saprospirae]	[Saprospirales]	Saprospiraceae	Unassigned	Unassigned
1145	1	0	0	1	0	0	0	0	0	0	0	0	Proteobacteria	Alphaproteobacteria	Caulobacterales	Caulobacteraceae	Unassigned	Unassigned

1146	1	0	0	1	0	0	0	0	0	0	0	0	0	GN02	BD1-5	Unassigned	Unassigned	Unassigned	Unassigned
1148	1	0	0	0	0	0	0	1	0	0	0	0	0	Firmicutes	Clostridia	Clostridiales	Clostridiaceae	Clostridium	Unassigned
1151	1	0	0	0	0	0	0	1	0	0	0	0	0	Proteobacteria	Gammaproteobacteria	Legionellales	Coxiellaceae	Aquicella	Unassigned
1153	0	0	0	0	0	0	0	0	0	0	0	0	0	Proteobacteria	Alphaproteobacteria	Rhodobacterales	Rhodobacteraceae	Thalassobacter	stenotrophicus
1155	1	0	0	0	0	0	0	1	0	0	0	0	0	Proteobacteria	Gammaproteobacteria	Oceanospirillales	Halomonadaceae	Halomonas	Unassigned
1156	1	0	0	0	0	0	0	1	0	0	0	0	0	Acidobacteria	Solibacteres	Solibacterales	PAUC26f	Unassigned	Unassigned
1157	1	0	0	0	0	0	0	1	0	0	0	0	0	Proteobacteria	Gammaproteobacteria	Oceanospirillales	Alcanivoracaceae	Alcanivorax	Unassigned
1161	1	0	0	0	0	1	0	0	0	0	0	0	0	Proteobacteria	Gammaproteobacteria	Thiotrichales	Thiotrichaceae	Thiothrix	Unassigned
1163	1	0	0	0	0	1	0	0	0	0	0	0	0	Proteobacteria	Deltaproteobacteria	Bdellovibrionales	Bdellovibrionaceae	Bdellovibrio	Unassigned
1164	1	0	0	0	0	1	0	0	0	0	0	0	0	Proteobacteria	Deltaproteobacteria	Unassigned	Unassigned	Unassigned	Unassigned
1165	1	0	0	0	0	1	0	0	0	0	0	0	0	Proteobacteria	Gammaproteobacteria	Legionellales	Unassigned	Unassigned	Unassigned
1167	1	0	0	0	0	0	0	1	0	0	0	0	0	Proteobacteria	Alphaproteobacteria	BD7-3	Unassigned	Unassigned	Unassigned
1168	1	0	0	0	0	1	0	0	0	0	0	0	0	Proteobacteria	Deltaproteobacteria	Myxococcales	Unassigned	Unassigned	Unassigned
1170	1	0	0	0	0	1	0	0	0	0	0	0	0	Bacteroidetes	Cytophagia	Cytophagales	Flammeovirgaceae	Fulvivirga	Unassigned
1171	0	0	0	0	0	0	0	0	0	0	0	0	0	Bacteroidetes	Bacteroidia	Bacteroidales	Bacteroidaceae	Bacteroides	Unassigned
1173	0	0	0	0	0	0	0	0	0	0	0	0	0	Bacteroidetes	BME43	Unassigned	Unassigned	Unassigned	Unassigned
1175	0	0	0	0	0	0	0	0	0	0	0	0	0	Proteobacteria	Gammaproteobacteria	Alteromonadales	Alteromonadaceae	Glaciecola	Unassigned
1176	0	0	0	0	0	0	0	0	0	0	0	0	0	Proteobacteria	Gammaproteobacteria	Chromatiales	Unassigned	Unassigned	Unassigned
1180	0	0	0	0	0	0	0	0	0	0	0	0	0	Proteobacteria	Gammaproteobacteria	Pasteurellales	Unassigned	Unassigned	Unassigned
1182	1	0	0	0	1	0	0	0	0	0	0	0	0	Bacteroidetes	Bacteroidia	Bacteroidales	Unassigned	Unassigned	Unassigned
1184	1	0	0	1	0	0	0	0	0	0	0	0	0	Proteobacteria	Deltaproteobacteria	Bdellovibrionales	Bacteriovoracaceae	Unassigned	Unassigned
1186	1	0	0	1	0	0	0	0	0	0	0	0	0	Proteobacteria	Deltaproteobacteria	Myxococcales	OM27	Unassigned	Unassigned
1187	1	0	0	0	1	0	0	0	0	0	0	0	0	Proteobacteria	Deltaproteobacteria	Unassigned	Unassigned	Unassigned	Unassigned
1188	1	0	0	1	0	0	0	0	0	0	0	0	0	Actinobacteria	Thermoleophilia	Solirubrobacterales	Unassigned	Unassigned	Unassigned
1189	1	0	0	1	0	0	0	0	0	0	0	0	0	Proteobacteria	Deltaproteobacteria	Syntrophobacterales	Syntrophaceae	Desulfobacca	Unassigned
1190	1	0	0	1	0	0	0	0	0	0	0	0	0	Proteobacteria	Alphaproteobacteria	Rhodobacterales	Rhodobacteraceae	Unassigned	Unassigned
1191	1	0	0	1	0	0	0	0	0	0	0	0	0	Proteobacteria	Gammaproteobacteria	Legionellales	Coxiellaceae	Unassigned	Unassigned
1192	1	0	0	1	0	0	0	0	0	0	0	0	0	Bacteroidetes	[Saprospirae]	[Saprospirales]	Saprospiraceae	Unassigned	Unassigned
1193	1	0	0	1	0	0	0	0	0	0	0	0	0	Bacteroidetes	[Saprospirae]	[Saprospirales]	Unassigned	Unassigned	Unassigned
1194	1	0	0	1	0	0	0	0	0	0	0	0	0	Proteobacteria	Gammaproteobacteria	Chromatiales	Ectothiorhodospiraceae	Unassigned	Unassigned
1195	1	0	0	1	0	0	0	0	0	0	0	0	0	Chloroflexi	Anaerolineae	SBR1031	A4b	Unassigned	Unassigned
1196	1	0	0	1	0	0	0	0	0	0	0	0	0	Acidobacteria	Sva0725	Sva0725	Unassigned	Unassigned	Unassigned
1197	1	0	0	0	1	0	0	0	0	0	0	0	0	Cyanobacteria	4C0d-2	SM2F09	Unassigned	Unassigned	Unassigned
1201	1	0	0	0	1	0	0	0	0	0	0	0	0	Proteobacteria	Gammaproteobacteria	Thiotrichales	Unassigned	Unassigned	Unassigned
1202	1	0	0	0	1	0	0	0	0	0	0	0	0	Bacteroidetes	Cytophagia	Cytophagales	[Amoebophilaceae]	Unassigned	Unassigned
1204	1	0	0	0	0	0	0	0	0	0	0	0	1	Bacteroidetes	BME43	Unassigned	Unassigned	Unassigned	Unassigned
1205	1	0	0	0	1	0	0	0	0	0	0	0	0	Proteobacteria	Deltaproteobacteria	Myxococcales	Nannocystaceae	Plesiocystis	Unassigned
1206	1	0	0	0	0	0	0	0	0	0	0	0	1	Verrucomicrobia	Verrucomicrobiae	Verrucomicrobiales	Verrucomicrobiaceae	Rubritalea	Unassigned
1207	1	0	0	0	0	0	0	0	0	0	0	0	1	Proteobacteria	Alphaproteobacteria	Unassigned	Unassigned	Unassigned	Unassigned
1208	1	0	0	0	1	0	0	0	0	0	0	0	0	Bacteroidetes	Cytophagia	Cytophagales	Flammeovirgaceae	Unassigned	Unassigned
1209	1	0	0	0	1	0	0	0	0	0	0	0	0	Proteobacteria	Deltaproteobacteria	Bdellovibrionales	Bacteriovoracaceae	Unassigned	Unassigned
1211	1	0	0	1	0	0	0	0	0	0	0	0	0	Bacteroidetes	[Saprospirae]	[Saprospirales]	Saprospiraceae	Unassigned	Unassigned
1212	1	0	0	1	0	0	0	0	0	0	0	0	0	Bacteroidetes	Bacteroidia	Bacteroidales	SB-1	Unassigned	Unassigned
1213	1	0	0	1	0	0	0	0	0	0	0	0	0	Proteobacteria	Gammaproteobacteria	Unassigned	Unassigned	Unassigned	Unassigned
1214	1	0	0	1	0	0	0	0	0	0	0	0	0	Bacteroidetes	[Saprospirae]	[Saprospirales]	Saprospiraceae	Lewinella	Unassigned

1217	1	0	0	0	0	0	0	1	0	0	0	0	0	Bacteroidetes	Bacteroidia	Bacteroidales	SB-1	Unassigned	Unassigned
1218	1	0	0	0	0	0	0	1	0	0	0	0	0	Proteobacteria	Gammaproteobacteria	Oceanospirillales	Halomonadaceae	Halomonas	Unassigned
1219	1	0	0	0	0	0	0	1	0	0	0	0	0	OP11	WCHB1-64	Unassigned	Unassigned	Unassigned	Unassigned
1220	1	0	0	0	0	0	0	1	0	0	0	0	0	Proteobacteria	Gammaproteobacteria	Alteromonadales	Alteromonadaceae	Unassigned	Unassigned
1222	1	0	0	0	0	0	0	1	0	0	0	0	0	Verrucomicrobia	[Pedosphaerae]	Unassigned	Unassigned	Unassigned	Unassigned
1224	1	0	0	1	0	0	0	0	0	0	0	0	0	Proteobacteria	Gammaproteobacteria	Vibrionales	Vibrionaceae	Vibrio	Unassigned
1225	1	0	0	1	0	0	0	0	0	0	0	0	0	Bacteroidetes	Cytophagia	Cytophagales	Flammeovirgaceae	Marinoscillum	furvescens
1226	1	0	0	0	0	0	0	0	1	0	0	0	0	Proteobacteria	Gammaproteobacteria	Alteromonadales	Idiomarinaceae	Idiomarina	Unassigned
1227	1	0	0	0	0	0	0	0	1	0	0	0	0	Bacteroidetes	Cytophagia	Cytophagales	Flammeovirgaceae	Unassigned	Unassigned
1230	1	0	0	0	0	0	0	0	1	0	0	0	0	Verrucomicrobia	Opitutae	[Pelagicoccales]	[Pelagicoccaceae]	Pelagicoccus	Unassigned
1231	1	0	0	0	0	0	0	0	1	0	0	0	0	Proteobacteria	Gammaproteobacteria	34P16	Unassigned	Unassigned	Unassigned
1232	1	0	0	0	0	0	0	0	1	0	0	0	0	Proteobacteria	Gammaproteobacteria	Chromatiales	Unassigned	Unassigned	Unassigned
1234	1	0	0	0	0	0	0	0	1	0	0	0	0	Proteobacteria	Deltaproteobacteria	Spirobacillales	Unassigned	Unassigned	Unassigned
1236	1	0	0	0	0	0	0	0	1	0	0	0	0	Proteobacteria	Deltaproteobacteria	GMD14H09	Unassigned	Unassigned	Unassigned
1238	1	0	0	0	0	0	0	0	1	0	0	0	0	Acidobacteria	Holophagae	Holophagales	Unassigned	Unassigned	Unassigned
1241	1	0	0	0	0	0	0	0	0	0	0	1	0	WS3	PRR-12	GN03	Unassigned	Unassigned	Unassigned
1242	1	0	0	0	0	0	0	0	0	0	0	1	0	TM7	TM7-1	Unassigned	Unassigned	Unassigned	Unassigned
1243	1	0	0	1	0	0	0	0	0	0	0	0	0	Proteobacteria	Gammaproteobacteria	Legionellales	Unassigned	Unassigned	Unassigned
1244	1	0	0	1	0	0	0	0	0	0	0	0	0	OP11	WCHB1-64	d153	Unassigned	Unassigned	Unassigned
1248	1	0	0	0	0	0	0	0	0	0	0	1	0	Proteobacteria	Deltaproteobacteria	Myxococcales	Unassigned	Unassigned	Unassigned
1249	1	0	0	0	0	0	0	0	0	0	0	1	0	Bacteroidetes	Bacteroidia	Bacteroidales	VC21_Bac22	Unassigned	Unassigned
1250	1	0	0	0	0	0	0	0	0	0	0	1	0	Proteobacteria	Deltaproteobacteria	Myxococcales	Unassigned	Unassigned	Unassigned
1251	1	0	0	0	0	0	0	0	0	0	0	1	0	Bacteroidetes	Bacteroidia	Bacteroidales	VC21_Bac22	Unassigned	Unassigned
1252	1	0	0	0	0	0	0	0	0	0	1	0	0	Proteobacteria	Deltaproteobacteria	PB19	Unassigned	Unassigned	Unassigned
1254	1	0	0	0	0	0	0	0	0	0	0	1	0	Bacteroidetes	[Saprospirae]	[Saprospirales]	Saprospiraceae	Lewinella	Unassigned
1255	0	0	0	0	0	0	0	0	0	0	0	0	0	Proteobacteria	Gammaproteobacteria	Legionellales	Coxiellaceae	Unassigned	Unassigned
1256	0	0	0	0	0	0	0	0	0	0	0	0	0	Proteobacteria	Epsilonproteobacteria	Campylobacterales	Campylobacteraceae	Arcobacter	Unassigned
1257	0	0	0	0	0	0	0	0	0	0	0	0	0	Proteobacteria	Deltaproteobacteria	Bdellovibrionales	Bdellovibrionaceae	Bdellovibrio	Unassigned
1259	0	0	0	0	0	0	0	0	0	0	0	0	0	Bacteroidetes	Flavobacteriia	Flavobacteriales	Flavobacteriaceae	Polaribacter	Unassigned
1260	1	0	0	1	0	0	0	0	0	0	0	0	0	Bacteroidetes	Cytophagia	Cytophagales	Flammeovirgaceae	Unassigned	Unassigned
1264	1	0	0	1	0	0	0	0	0	0	0	0	0	Bacteroidetes	Bacteroidia	Bacteroidales	Unassigned	Unassigned	Unassigned
1265	1	0	0	1	0	0	0	0	0	0	0	0	0	Proteobacteria	Gammaproteobacteria	Legionellales	Unassigned	Unassigned	Unassigned
1266	1	0	0	1	0	0	0	0	0	0	0	0	0	Proteobacteria	Gammaproteobacteria	Oceanospirillales	Hahellaceae	Hahella	ganghwensis
1267	1	0	0	1	0	0	0	0	0	0	0	0	0	Chloroflexi	Anaerolineae	SBR1031	A4b	Unassigned	Unassigned
1268	0	0	0	0	0	0	0	0	0	0	0	0	0	TM7	TM7-3	Blgi18	Unassigned	Unassigned	Unassigned
1269	1	0	0	1	0	0	0	0	0	0	0	0	0	TM7	TM7-1	Unassigned	Unassigned	Unassigned	Unassigned
1270	1	0	0	1	0	0	0	0	0	0	0	0	0	Cyanobacteria	Oscillatoriophycideae	Oscillatoriales	Phormidiaceae	Microcoleus	Unassigned
1271	1	0	0	1	0	0	0	0	0	0	0	0	0	Proteobacteria	Gammaproteobacteria	HOC36	Unassigned	Unassigned	Unassigned
1272	1	0	0	1	0	0	0	0	0	0	0	0	0	Proteobacteria	Unassigned	Unassigned	Unassigned	Unassigned	Unassigned
1273	1	0	0	1	0	0	0	0	0	0	0	0	0	Unassigned	Unassigned	Unassigned	Unassigned	Unassigned	Unassigned
1275	1	0	0	1	0	0	0	0	0	0	0	0	0	Proteobacteria	Gammaproteobacteria	Pseudomonadales	Moraxellaceae	Psychrobacter	pacificensis
1276	1	0	0	1	0	0	0	0	0	0	0	0	0	Actinobacteria	Acidimicrobiia	Acidimicrobiales	Unassigned	Unassigned	Unassigned
1277	1	0	0	1	0	0	0	0	0	0	0	0	0	Actinobacteria	Acidimicrobiia	Acidimicrobiales	Unassigned	Unassigned	Unassigned
1278	1	0	0	1	0	0	0	0	0	0	0	0	0	Proteobacteria	Alphaproteobacteria	Unassigned	Unassigned	Unassigned	Unassigned
1280	1	0	0	0	0	0	0	0	0	0	0	1	0	Proteobacteria	Deltaproteobacteria	Desulfobacterales	Desulfobulbaceae	Unassigned	Unassigned

1281	1	0	0	0	0	0	0	0	0	0	0	1	0	Chloroflexi	Anaerolineae	CFB-26	Unassigned	Unassigned	Unassigned
1283	1	0	0	0	0	0	0	0	0	0	0	1	0	Proteobacteria	Deltaproteobacteria	Myxococcales	Unassigned	Unassigned	Unassigned
1284	1	0	0	0	0	0	0	0	0	0	0	1	0	Proteobacteria	Gammaproteobacteria	Alteromonadales	OM60	Unassigned	Unassigned
1286	1	0	0	0	0	0	0	1	0	0	0	0	0	Proteobacteria	Alphaproteobacteria	Rickettsiales	Rickettsiaceae	Unassigned	Unassigned
1287	1	0	0	0	0	0	0	1	0	0	0	0	0	Actinobacteria	Actinobacteria	Actinomycetales	Unassigned	Unassigned	Unassigned
1289	1	0	0	0	0	0	0	1	0	0	0	0	0	Chlorobi	Ignavibacteria	Ignavibacteriales	lheB3-7	Unassigned	Unassigned
1290	1	0	0	0	0	0	0	1	0	0	0	0	0	Spirochaetes	Spirochaetes	Spirochaetales	Spirochaetaceae	Unassigned	Unassigned
1291	1	0	0	0	0	0	0	1	0	0	0	0	0	Chlorobi	Ignavibacteria	Ignavibacteriales	Ignavibacteriaceae	Unassigned	Unassigned
1292	1	0	0	0	0	0	0	1	0	0	0	0	0	Bacteroidetes	Flavobacteriia	Flavobacteriales	Cryomorphaceae	Unassigned	Unassigned
1293	1	0	0	0	0	0	0	1	0	0	0	0	0	Acidobacteria	AT-s2-57	Unassigned	Unassigned	Unassigned	Unassigned
1294	1	0	0	0	0	0	0	1	0	0	0	0	0	Proteobacteria	Gammaproteobacteria	Chromatiales	Unassigned	Unassigned	Unassigned
1296	0	0	0	0	0	0	0	0	0	0	0	0	0	Proteobacteria	Gammaproteobacteria	Legionellales	Coxiellaceae	Unassigned	Unassigned
1298	0	0	0	0	0	0	0	0	0	0	0	0	0	Proteobacteria	Gammaproteobacteria	Alteromonadales	Colwelliaceae	Thalassomonas	Unassigned
1299	0	0	0	0	0	0	0	0	0	0	0	0	0	Bacteroidetes	Flavobacteriia	Flavobacteriales	Cryomorphaceae	Unassigned	Unassigned
1300	1	0	0	0	0	0	0	0	0	0	1	0	0	Spirochaetes	Spirochaetes	Spirochaetales	Spirochaetaceae	Spirochaeta	Unassigned
1301	1	0	0	0	0	0	0	0	0	0	1	0	0	Unassigned	Unassigned	Unassigned	Unassigned	Unassigned	Unassigned
1305	0	0	0	0	0	0	0	0	0	0	0	0	0	Proteobacteria	Deltaproteobacteria	Unassigned	Unassigned	Unassigned	Unassigned
1307	0	0	0	0	0	0	0	0	0	0	0	0	0	GN02	BB34	Unassigned	Unassigned	Unassigned	Unassigned
1308	0	0	0	0	0	0	0	0	0	0	0	0	0	Bacteroidetes	[Saprospirae]	[Saprospirales]	Saprospiraceae	Unassigned	Unassigned
1309	0	0	0	0	0	0	0	0	0	0	0	0	0	Proteobacteria	Gammaproteobacteria	Legionellales	Coxiellaceae	Unassigned	Unassigned
1310	0	0	0	0	0	0	0	0	0	0	0	0	0	Proteobacteria	Alphaproteobacteria	Rickettsiales	Rickettsiaceae	Unassigned	Unassigned
1311	0	0	0	0	0	0	0	0	0	0	0	0	0	Proteobacteria	Gammaproteobacteria	Alteromonadales	Alteromonadaceae	HTCC2207	Unassigned
1312	1	0	0	0	0	0	0	0	0	0	0	0	1	Proteobacteria	Gammaproteobacteria	[Marinicellales]	[Marinicellaceae]	Marinicella	Unassigned
1314	1	0	0	0	0	0	0	0	0	0	0	0	1	Proteobacteria	Gammaproteobacteria	HTCC2188	HTCC2089	Unassigned	Unassigned
1315	1	0	0	0	0	0	0	0	0	0	0	0	1	Proteobacteria	Gammaproteobacteria	Alteromonadales	Unassigned	Unassigned	Unassigned
1316	1	0	0	0	0	0	0	0	0	0	0	0	1	Proteobacteria	Gammaproteobacteria	Unassigned	Unassigned	Unassigned	Unassigned
1318	1	0	0	0	0	0	0	0	0	0	0	0	1	Proteobacteria	Deltaproteobacteria	Desulfarculales	Desulfarculaceae	Unassigned	Unassigned
1319	1	0	0	0	0	0	1	0	0	0	0	0	0	Bacteroidetes	[Saprospirae]	[Saprospirales]	Chitinophagaceae	Unassigned	Unassigned
1321	1	0	0	0	0	0	0	0	0	0	0	0	1	Actinobacteria	Nitriliruptoria	Nitriliruptorales	Nitriliruptoraceae	Unassigned	Unassigned
1322	1	0	0	0	0	0	0	0	0	0	0	0	1	Bacteroidetes	Bacteroidia	Bacteroidales	Unassigned	Unassigned	Unassigned
1327	1	0	0	0	0	0	0	0	1	0	0	0	0	Proteobacteria	Alphaproteobacteria	BD7-3	Unassigned	Unassigned	Unassigned
1328	1	0	0	0	0	0	0	0	1	0	0	0	0	Proteobacteria	Gammaproteobacteria	Alteromonadales	Idiomarinaceae	Idiomarina	Unassigned
1330	1	0	0	0	0	0	0	0	1	0	0	0	0	Proteobacteria	Deltaproteobacteria	Myxococcales	Unassigned	Unassigned	Unassigned
1332	1	0	0	0	0	0	0	0	0	0	0	1	0	Proteobacteria	Gammaproteobacteria	Unassigned	Unassigned	Unassigned	Unassigned
1333	0	0	0	0	0	0	0	0	0	0	0	0	0	Bacteroidetes	Flavobacteriia	Flavobacteriales	Unassigned	Unassigned	Unassigned
1334	1	0	0	0	0	0	0	0	0	0	0	1	0	Proteobacteria	Alphaproteobacteria	Rhizobiales	Hyphomicrobiaceae	Pedomicrobium	Unassigned
1335	0	0	0	0	0	0	0	0	0	0	0	0	0	Unassigned	Unassigned	Unassigned	Unassigned	Unassigned	Unassigned
1336	1	0	0	0	0	0	0	0	0	0	0	1	0	Bacteroidetes	Bacteroidia	Bacteroidales	Unassigned	Unassigned	Unassigned
1337	1	0	0	0	0	0	0	0	0	0	0	1	0	Actinobacteria	Thermoleophilia	Solirubrobacterales	Unassigned	Unassigned	Unassigned
1338	1	0	0	0	0	0	0	0	0	0	0	1	0	Chloroflexi	Anaerolineae	OPB11	Unassigned	Unassigned	Unassigned
1339	0	0	0	0	0	0	0	0	0	0	0	0	0	Proteobacteria	Gammaproteobacteria	Alteromonadales	Alteromonadaceae	Glaciecola	Unassigned
1340	1	0	0	0	0	0	0	0	0	0	0	1	0	Proteobacteria	Deltaproteobacteria	Myxococcales	Unassigned	Unassigned	Unassigned
1342	1	0	0	0	0	0	0	0	0	0	0	1	0	Proteobacteria	Deltaproteobacteria	Desulfuromonadales	Desulfuromonadaceae	Unassigned	Unassigned
1343	1	0	0	0	0	0	0	0	0	0	0	1	0	Bacteroidetes	[Saprospirae]	[Saprospirales]	Saprospiraceae	Lewinella	Unassigned
1345	0	0	0	0	0	0	0	0	0	0	0	0	0	Bacteroidetes	Sphingobacteriia	Sphingobacteriales	Unassigned	Unassigned	Unassigned

1346	0	0	0	0	0	0	0	0	0	0	0	0	Unassigned	Unassigned	Unassigned	Unassigned	Unassigned	Unassigned
1348	0	0	0	0	0	0	0	0	0	0	0	0	Proteobacteria	Gammaproteobacteria	Legionellales	Unassigned	Unassigned	Unassigned
1350	1	0	0	0	0	0	0	0	0	0	0	1	Proteobacteria	Zetaproteobacteria	Mariprofundales	Mariprofundaceae	Mariprofundus	Unassigned
1351	1	0	0	0	0	0	0	0	0	0	0	1	Bacteroidetes	Bacteroidia	Bacteroidales	Unassigned	Unassigned	Unassigned
1352	0	0	0	0	0	0	0	0	0	0	0	0	Proteobacteria	Deltaproteobacteria	Bdellovibrionales	Bacteriovoracaceae	Unassigned	Unassigned
1355	0	0	0	0	0	0	0	0	0	0	0	0	Acidobacteria	Holophagae	Holophagales	Unassigned	Unassigned	Unassigned
1357	0	0	0	0	0	0	0	0	0	0	0	0	Proteobacteria	Gammaproteobacteria	Legionellales	Francisellaceae	Unassigned	Unassigned
1358	0	0	0	0	0	0	0	0	0	0	0	0	Proteobacteria	Gammaproteobacteria	Oceanospirillales	Hahellaceae	Hahella	Unassigned
1359	1	0	1	0	0	0	0	0	0	0	0	0	Gemmatimonadete:	Gemm-2	Unassigned	Unassigned	Unassigned	Unassigned
1361	1	0	1	0	0	0	0	0	0	0	0	0	Proteobacteria	TA18	CV90	Unassigned	Unassigned	Unassigned
1362	0	0	0	0	0	0	0	0	0	0	0	0	Bacteroidetes	Flavobacteriia	Flavobacteriales	Unassigned	Unassigned	Unassigned
1364	0	0	0	0	0	0	0	0	0	0	0	0	Proteobacteria	Gammaproteobacteria	Chromatiales	Chromatiaceae	Marichromatium	Unassigned
1365	0	0	0	0	0	0	0	0	0	0	0	0	Bacteroidetes	Cytophagia	Cytophagales	Flammeovirgaceae	Unassigned	Unassigned
1366	0	0	0	0	0	0	0	0	0	0	0	0	Proteobacteria	Epsilonproteobacteria	Campylobacterales	Helicobacteraceae	Sulfurimonas	Unassigned
1367	0	0	0	0	0	0	0	0	0	0	0	0	Proteobacteria	Alphaproteobacteria	Rhizobiales	Phyllobacteriaceae	Unassigned	Unassigned
1369	0	0	0	0	0	0	0	0	0	0	0	0	Proteobacteria	Gammaproteobacteria	Enterobacteriales	Enterobacteriaceae	Providencia	Unassigned
1370	1	0	0	0	1	0	0	0	0	0	0	0	Proteobacteria	Deltaproteobacteria	Desulfuromonadales	Pelobacteraceae	Pelobacter	Unassigned
1372	0	0	0	0	0	0	0	0	0	0	0	0	Proteobacteria	Gammaproteobacteria	Legionellales	Coxiellaceae	Unassigned	Unassigned
1374	1	0	0	0	1	0	0	0	0	0	0	0	TM6	SJA-4	Unassigned	Unassigned	Unassigned	Unassigned
1375	0	0	0	0	0	0	0	0	0	0	0	0	Bacteroidetes	Bacteroidia	Bacteroidales	Porphyromonadaceae	Dysgonomonas	Unassigned
1376	1	0	0	0	1	0	0	0	0	0	0	0	Proteobacteria	Deltaproteobacteria	Myxococcales	Unassigned	Unassigned	Unassigned
1377	1	0	0	0	1	0	0	0	0	0	0	0	Actinobacteria	Acidimicrobiia	Acidimicrobiales	JdFBGBact	Unassigned	Unassigned
1380	1	0	0	1	0	0	0	0	0	0	0	0	Proteobacteria	Gammaproteobacteria	Legionellales	Coxiellaceae	Unassigned	Unassigned
1381	1	0	0	1	0	0	0	0	0	0	0	0	Proteobacteria	Deltaproteobacteria	NB1-j	JTB38	Unassigned	Unassigned
1383	1	0	0	1	0	0	0	0	0	0	0	0	Bacteroidetes	Bacteroidia	Bacteroidales	Unassigned	Unassigned	Unassigned
1384	1	0	0	1	0	0	0	0	0	0	0	0	Proteobacteria	Gammaproteobacteria	Chromatiales	Unassigned	Unassigned	Unassigned
1386	1	0	0	0	0	0	0	0	0	0	0	1	Proteobacteria	Deltaproteobacteria	Myxococcales	Polyangiaceae	Unassigned	Unassigned
1391	1	0	0	1	0	0	0	0	0	0	0	0	Proteobacteria	Deltaproteobacteria	Bdellovibrionales	Bacteriovoracaceae	Unassigned	Unassigned
1393	1	0	0	0	0	0	0	0	0	0	0	1	Spirochaetes	Spirochaetes	Spirochaetales	Spirochaetaceae	Unassigned	Unassigned
1394	1	0	0	0	0	0	0	0	0	0	0	1	Proteobacteria	Gammaproteobacteria	Alteromonadales	Alteromonadaceae	Unassigned	Unassigned
1395	1	0	0	0	0	0	0	0	0	0	0	1	Proteobacteria	Gammaproteobacteria	Thiotrichales	Piscirickettsiaceae	Unassigned	Unassigned
1396	1	0	0	0	0	0	0	0	0	0	0	1	Unassigned	Unassigned	Unassigned	Unassigned	Unassigned	Unassigned
1397	1	0	0	0	0	0	0	0	0	0	0	1	Gemmatimonadete:	Gemm-2	Unassigned	Unassigned	Unassigned	Unassigned
1398	1	0	0	0	1	0	0	0	0	0	0	0	Proteobacteria	Deltaproteobacteria	Unassigned	Unassigned	Unassigned	Unassigned
1400	1	0	0	0	1	0	0	0	0	0	0	0	Chlorobi	SJA-28	Unassigned	Unassigned	Unassigned	Unassigned
1402	1	0	0	1	0	0	0	0	0	0	0	0	Proteobacteria	Gammaproteobacteria	Chromatiales	Unassigned	Unassigned	Unassigned
1405	0	0	0	0	0	0	0	0	0	0	0	0	Proteobacteria	Gammaproteobacteria	Alteromonadales	Unassigned	Unassigned	Unassigned
1406	1	0	0	1	0	0	0	0	0	0	0	0	Gemmatimonadete:	Gemm-2	Unassigned	Unassigned	Unassigned	Unassigned
1407	1	0	0	1	0	0	0	0	0	0	0	0	Proteobacteria	Gammaproteobacteria	Legionellales	Coxiellaceae	Unassigned	Unassigned
1409	1	0	0	1	0	0	0	0	0	0	0	0	Chloroflexi	Anaerolineae	CFB-26	Unassigned	Unassigned	Unassigned
1410	1	0	0	0	0	0	0	0	0	0	1	0	Proteobacteria	Deltaproteobacteria	Bdellovibrionales	Bacteriovoracaceae	Unassigned	Unassigned
1411	1	0	0	1	0	0	0	0	0	0	0	0	Chloroflexi	Anaerolineae	SBR1031	A4b	Unassigned	Unassigned
1412	1	0	0	1	0	0	0	0	0	0	0	0	Chloroflexi	Anaerolineae	SBR1031	A4b	Unassigned	Unassigned
1413	1	0	0	1	0	0	0	0	0	0	0	0	Fibrobacteres	Fibrobacteria	258ds10	Unassigned	Unassigned	Unassigned
1414	1	0	0	1	0	0	0	0	0	0	0	0	Bacteroidetes	[Saprospirae]	[Saprospirales]	Saprospiraceae	Unassigned	Unassigned

1415	1	0	0	1	0	0	0	0	0	0	0	0	Bacteroidetes	[Rhodothermi]	[Rhodothermales]	Rhodothermaceae	Unassigned	Unassigned
1417	1	0	0	0	0	0	0	0	1	0	0	0	Proteobacteria	Deltaproteobacteria	Spirobacillales	Unassigned	Unassigned	Unassigned
1418	1	0	0	0	0	0	0	0	1	0	0	0	Proteobacteria	Alphaproteobacteria	Unassigned	Unassigned	Unassigned	Unassigned
1419	1	0	0	0	1	0	0	0	0	0	0	0	Proteobacteria	Gammaproteobacteria	Legionellales	Coxiellaceae	Unassigned	Unassigned
1420	1	0	0	0	1	0	0	0	0	0	0	0	Bacteroidetes	Flavobacteriia	Flavobacteriales	Flavobacteriaceae	Unassigned	Unassigned
1421	1	0	0	0	1	0	0	0	0	0	0	0	Proteobacteria	Deltaproteobacteria	Spirobacillales	Unassigned	Unassigned	Unassigned
1422	0	0	0	0	0	0	0	0	0	0	0	0	Proteobacteria	Gammaproteobacteria	Unassigned	Unassigned	Unassigned	Unassigned
1423	1	0	0	0	1	0	0	0	0	0	0	0	Acidobacteria	Holophagae	Holophagales	Unassigned	Unassigned	Unassigned
1424	0	0	0	0	0	0	0	0	0	0	0	0	Bacteroidetes	Cytophagia	Cytophagales	Unassigned	Unassigned	Unassigned
1428	1	0	0	0	1	0	0	0	0	0	0	0	Proteobacteria	Alphaproteobacteria	Rickettsiales	Rickettsiaceae	Unassigned	Unassigned
1431	1	0	0	0	1	0	0	0	0	0	0	0	Gemmatimonadetes	Gemm-2	Unassigned	Unassigned	Unassigned	Unassigned
1432	1	0	0	0	1	0	0	0	0	0	0	0	OP11	WCHB1-64	d153	Unassigned	Unassigned	Unassigned
1435	0	0	0	0	0	0	0	0	0	0	0	0	Bacteroidetes	Flavobacteriia	Flavobacteriales	Flavobacteriaceae	Croceitalea	dokdonensis
1436	0	0	0	0	0	0	0	0	0	0	0	0	Verrucomicrobia	Verrucomicrobiae	Verrucomicrobiales	Verrucomicrobiaceae	Haloferula	Unassigned
1437	0	0	0	0	0	0	0	0	0	0	0	0	Planctomycetes	Phycisphaerae	Phycisphaerales	Unassigned	Unassigned	Unassigned
1438	1	0	0	1	0	0	0	0	0	0	0	0	Proteobacteria	Deltaproteobacteria	Myxococcales	Haliangiaceae	Unassigned	Unassigned
1439	1	0	0	1	0	0	0	0	0	0	0	0	Proteobacteria	Gammaproteobacteria	Legionellales	Unassigned	Unassigned	Unassigned
1440	0	0	0	0	0	0	0	0	0	0	0	0	Proteobacteria	Alphaproteobacteria	BD7-3	Unassigned	Unassigned	Unassigned
1442	1	0	0	0	0	0	1	0	0	0	0	0	Proteobacteria	Alphaproteobacteria	Rhodospirillales	Rhodospirillaceae	Unassigned	Unassigned
1443	1	0	0	0	0	0	1	0	0	0	0	0	Proteobacteria	Gammaproteobacteria	Unassigned	Unassigned	Unassigned	Unassigned
1444	1	0	0	0	0	0	0	0	1	0	0	0	Proteobacteria	Gammaproteobacteria	Alteromonadales	Idiomarinaceae	Idiomarina	Unassigned
1445	1	0	0	0	0	0	1	0	0	0	0	0	Bacteroidetes	[Saprospirae]	[Saprospirales]	Chitinophagaceae	Unassigned	Unassigned
1448	0	0	0	0	0	0	0	0	0	0	0	0	Proteobacteria	Unassigned	Unassigned	Unassigned	Unassigned	Unassigned
1449	1	0	0	0	1	0	0	0	0	0	0	0	Proteobacteria	Alphaproteobacteria	Unassigned	Unassigned	Unassigned	Unassigned
1451	0	0	0	0	0	0	0	0	0	0	0	0	Elusimicrobia	Elusimicrobia	Elusimicrobiales	Elusimicrobiaceae	Elusimicrobium	minutum
1452	1	0	0	0	1	0	0	0	0	0	0	0	Proteobacteria	Gammaproteobacteria	Legionellales	Coxiellaceae	Unassigned	Unassigned
1453	1	0	0	0	1	0	0	0	0	0	0	0	Proteobacteria	Gammaproteobacteria	Alteromonadales	HTCC2188	HTCC	Unassigned
1455	1	0	0	0	1	0	0	0	0	0	0	0	Proteobacteria	Gammaproteobacteria	HTCC2188	HTCC2089	Unassigned	Unassigned
1456	1	0	0	0	1	0	0	0	0	0	0	0	WS3	PRR-12	Sediment-1	Unassigned	Unassigned	Unassigned
1457	1	0	0	0	1	0	0	0	0	0	0	0	Proteobacteria	Gammaproteobacteria	Thiotrichales	Thiotrichaceae	Unassigned	Unassigned
1459	1	0	0	0	1	0	0	0	0	0	0	0	Proteobacteria	Deltaproteobacteria	NB1-j	JTB38	Unassigned	Unassigned
1463	1	0	0	0	0	0	0	0	0	0	0	1	Proteobacteria	Deltaproteobacteria	Myxococcales	Unassigned	Unassigned	Unassigned
1464	1	0	0	0	0	0	0	0	0	0	0	1	Planctomycetes	Phycisphaerae	Phycisphaerales	Unassigned	Unassigned	Unassigned
1466	1	0	0	0	0	0	0	0	0	0	0	1	Proteobacteria	Deltaproteobacteria	Desulfobacterales	Desulfobulbaceae	Unassigned	Unassigned
1468	1	0	0	0	0	0	0	0	0	0	0	1	Firmicutes	Clostridia	Clostridiales	Clostridiaceae	Clostridium	Unassigned
1469	1	0	0	0	0	0	1	0	0	0	0	0	Proteobacteria	Alphaproteobacteria	Rhodobacterales	Rhodobacteraceae	Unassigned	Unassigned
1470	1	0	0	0	0	0	1	0	0	0	0	0	Proteobacteria	Alphaproteobacteria	Rickettsiales	Rickettsiaceae	Unassigned	Unassigned
1471	1	0	0	0	0	0	1	0	0	0	0	0	Bacteroidetes	Flavobacteriia	Flavobacteriales	Unassigned	Unassigned	Unassigned
1474	1	0	0	0	0	0	0	0	0	0	0	1	Proteobacteria	Deltaproteobacteria	Desulfuromonadales	Pelobacteraceae	Unassigned	Unassigned
1475	1	0	0	0	0	0	1	0	0	0	0	0	Proteobacteria	Alphaproteobacteria	BD7-3	Unassigned	Unassigned	Unassigned
1476	1	0	0	0	0	0	0	1	0	0	0	0	Armatimonadetes	[Fimbriimonadia]	[Fimbriimonadales]	Unassigned	Unassigned	Unassigned
1477	1	0	0	0	0	0	0	1	0	0	0	0	Acidobacteria	[Chloracidobacteria]	RB41	Ellin6075	Unassigned	Unassigned
1479	1	0	0	0	0	0	0	1	0	0	0	0	Proteobacteria	Gammaproteobacteria	Unassigned	Unassigned	Unassigned	Unassigned
1480	1	0	0	0	0	0	0	1	0	0	0	0	Proteobacteria	Gammaproteobacteria	Thiotrichales	Piscirickettsiaceae	Methylophaga	Unassigned
1481	1	0	0	0	0	0	0	1	0	0	0	0	Bacteroidetes	[Rhodothermi]	[Rhodothermales]	[Balneolaceae]	KSA1	Unassigned

1482	1	0	0	0	0	0	0	1	0	0	0	0	0	Acidobacteria	[Chloracidobacteria]	RB41	Ellin6075	Unassigned	Unassigned
1483	1	0	0	0	0	0	0	1	0	0	0	0	0	Proteobacteria	Gammaproteobacteria	Legionellales	Coxiellaceae	Unassigned	Unassigned
1484	1	0	0	0	0	0	0	1	0	0	0	0	0	Caldithrix	Caldithrixae	Caldithrixales	BA059	Unassigned	Unassigned
1485	1	0	0	0	0	0	0	1	0	0	0	0	0	Gemmatimonadete	Gemm-5	Unassigned	Unassigned	Unassigned	Unassigned
1486	1	0	0	0	0	0	0	1	0	0	0	0	0	Proteobacteria	Gammaproteobacteria	Oceanospirillales	Halomonadaceae	Halomonas	Unassigned
1487	1	0	0	0	0	0	0	1	0	0	0	0	0	Acidobacteria	Solibacteres	Solibacterales	PAUC26f	Unassigned	Unassigned
1489	1	0	0	0	0	1	0	0	0	0	0	0	0	Proteobacteria	Gammaproteobacteria	Thiotrichales	Thiotrichaceae	Unassigned	Unassigned
1491	1	0	0	0	0	1	0	0	0	0	0	0	0	Proteobacteria	Gammaproteobacteria	HTCC2188	Unassigned	Unassigned	Unassigned
1492	1	0	0	0	0	1	0	0	0	0	0	0	0	Proteobacteria	Gammaproteobacteria	Thiotrichales	Thiotrichaceae	Leucothrix	Unassigned
1493	1	0	0	0	0	1	0	0	0	0	0	0	0	Bacteroidetes	Flavobacteriia	Flavobacteriales	Unassigned	Unassigned	Unassigned
1494	1	0	0	0	0	1	0	0	0	0	0	0	0	Proteobacteria	Alphaproteobacteria	BD7-3	Unassigned	Unassigned	Unassigned
1495	1	0	0	0	0	1	0	0	0	0	0	0	0	Proteobacteria	Gammaproteobacteria	Alteromonadales	Alteromonadaceae	nsmpVI18	Unassigned
1496	1	0	0	0	0	1	0	0	0	0	0	0	0	Proteobacteria	Deltaproteobacteria	Myxococcales	Polyangiaceae	Unassigned	Unassigned
1497	1	0	0	0	0	1	0	0	0	0	0	0	0	Bacteroidetes	Flavobacteriia	Flavobacteriales	Unassigned	Unassigned	Unassigned
1502	0	0	0	0	0	0	0	0	0	0	0	0	0	Proteobacteria	Gammaproteobacteria	Legionellales	Unassigned	Unassigned	Unassigned
1504	1	0	0	0	0	0	0	0	0	0	0	1	0	WS3	PRR-12	GN03	Unassigned	Unassigned	Unassigned
1505	1	0	0	0	0	0	0	0	0	0	0	1	0	Gemmatimonadete	Gemm-2	Unassigned	Unassigned	Unassigned	Unassigned
1506	1	0	0	0	0	0	0	0	0	0	0	1	0	Bacteroidetes	[Saprospirae]	[Saprospirales]	Saprospiraceae	Unassigned	Unassigned
1508	0	0	0	0	0	0	0	0	0	0	0	0	0	Proteobacteria	Alphaproteobacteria	Rickettsiales	Rickettsiaceae	Unassigned	Unassigned
1509	0	0	0	0	0	0	0	0	0	0	0	0	0	Proteobacteria	Unassigned	Unassigned	Unassigned	Unassigned	Unassigned
1510	0	0	0	0	0	0	0	0	0	0	0	0	0	Proteobacteria	Deltaproteobacteria	Bdellovibrionales	Bacteriovoracaceae	Unassigned	Unassigned
1511	0	0	0	0	0	0	0	0	0	0	0	0	0	Proteobacteria	Alphaproteobacteria	Rickettsiales	Rickettsiaceae	Unassigned	Unassigned
1512	0	0	0	0	0	0	0	0	0	0	0	0	0	Verrucomicrobia	Opitutae	Puniceicoccales	Puniceicoccaceae	Unassigned	Unassigned
1513	0	0	0	0	0	0	0	0	0	0	0	0	0	Proteobacteria	TA18	PHOS-HD29	Unassigned	Unassigned	Unassigned
1515	1	0	0	0	0	1	0	0	0	0	0	0	0	Planctomycetes	Phycisphaerae	Phycisphaerales	Phycisphaeraceae	Unassigned	Unassigned
1518	0	0	0	0	0	0	0	0	0	0	0	0	0	GN02	BB34	Unassigned	Unassigned	Unassigned	Unassigned
1519	0	0	0	0	0	0	0	0	0	0	0	0	0	Proteobacteria	Gammaproteobacteria	Chromatiales	Chromatiaceae	Allochromatium	vinosum
1520	1	0	0	0	1	0	0	0	0	0	0	0	0	Actinobacteria	Acidimicrobiia	Acidimicrobiales	SC3-41	Unassigned	Unassigned
1521	0	0	0	0	0	0	0	0	0	0	0	0	0	Proteobacteria	Deltaproteobacteria	Spirobacillales	Unassigned	Unassigned	Unassigned
1523	0	0	0	0	0	0	0	0	0	0	0	0	0	SR1	Unassigned	Unassigned	Unassigned	Unassigned	Unassigned
1524	0	0	0	0	0	0	0	0	0	0	0	0	0	TM6	SBRH58	Unassigned	Unassigned	Unassigned	Unassigned
1526	0	0	0	0	0	0	0	0	0	0	0	0	0	TM6	SJA-4	Unassigned	Unassigned	Unassigned	Unassigned
1527	0	0	0	0	0	0	0	0	0	0	0	0	0	Proteobacteria	Deltaproteobacteria	Myxococcales	Polyangiaceae	Chondromyces	Unassigned
1529	1	1	0	0	0	0	0	0	0	0	0	0	0	Bacteroidetes	[Saprospirae]	[Saprospirales]	Unassigned	Unassigned	Unassigned
1530	1	1	0	0	0	0	0	0	0	0	0	0	0	Verrucomicrobia	Verrucomicrobiae	Verrucomicrobiales	Verrucomicrobiaceae	Unassigned	Unassigned
1531	1	0	0	0	0	0	0	0	0	0	0	1	0	Bacteroidetes	Flavobacteriia	Flavobacteriales	Cryomorphaceae	Crocinitomix	Unassigned
1532	1	1	0	0	0	0	0	0	0	0	0	0	0	Proteobacteria	Deltaproteobacteria	Myxococcales	Nannocystaceae	Plesiocystis	Unassigned
1533	1	1	0	0	0	0	0	0	0	0	0	0	0	Proteobacteria	Alphaproteobacteria	Unassigned	Unassigned	Unassigned	Unassigned
1534	1	0	0	0	0	0	0	1	0	0	0	0	0	Proteobacteria	Betaproteobacteria	Burkholderiales	Comamonadaceae	Limnohabitans	Unassigned
1535	1	1	0	0	0	0	0	0	0	0	0	0	0	Proteobacteria	Gammaproteobacteria	Alteromonadales	OM60	Unassigned	Unassigned
1537	1	1	0	0	0	0	0	0	0	0	0	0	0	Proteobacteria	Gammaproteobacteria	Legionellales	Unassigned	Unassigned	Unassigned
1538	1	0	0	0	0	0	0	1	0	0	0	0	0	Proteobacteria	Deltaproteobacteria	Spirobacillales	Unassigned	Unassigned	Unassigned
1539	1	0	0	0	0	0	0	1	0	0	0	0	0	Proteobacteria	Deltaproteobacteria	Myxococcales	Haliangiaceae	Unassigned	Unassigned
1541	1	0	0	0	0	0	0	0	0	0	1	0	0	Tenericutes	Mollicutes	RF39	Unassigned	Unassigned	Unassigned
1542	1	0	0	0	0	0	0	0	0	0	0	1	0	Chloroflexi	Anaerolineae	Anaerolineales	Anaerolinaceae	C1_B004	Unassigned

1545	1	0	0	0	0	0	0	0	0	0	0	1	0	Bacteroidetes	[Rhodothermi]	[Rhodothermales]	Rhodothermaceae	Unassigned	Unassigned
1546	1	0	0	0	0	0	0	0	0	0	0	1	0	Actinobacteria	Actinobacteria	Actinomycetales	Nocardioidaceae	Unassigned	Unassigned
1548	1	0	0	0	0	1	0	0	0	0	0	0	0	Proteobacteria	Alphaproteobacteria	Rickettsiales	Unassigned	Unassigned	Unassigned
1550	1	0	0	0	0	1	0	0	0	0	0	0	0	Proteobacteria	Deltaproteobacteria	Myxococcales	Unassigned	Unassigned	Unassigned
1551	0	0	0	0	0	0	0	0	0	0	0	0	0	Firmicutes	Bacilli	Lactobacillales	Enterococcaceae	Vagococcus	Unassigned
1553	0	0	0	0	0	0	0	0	0	0	0	0	0	Bacteroidetes	Bacteroidia	Bacteroidales	Porphyromonadaceae	Dysgonomonas	gadei
1554	1	0	0	0	0	0	0	0	0	1	0	0	0	Proteobacteria	Deltaproteobacteria	Myxococcales	Unassigned	Unassigned	Unassigned
1555	1	0	0	0	0	0	0	1	0	0	0	0	0	OD1	ZB2	Unassigned	Unassigned	Unassigned	Unassigned
1556	1	0	0	0	0	0	0	1	0	0	0	0	0	Proteobacteria	Alphaproteobacteria	Rhizobiales	Unassigned	Unassigned	Unassigned
1557	1	0	0	0	0	0	0	1	0	0	0	0	0	Proteobacteria	Gammaproteobacteria	Legionellales	Legionellaceae	Unassigned	Unassigned
1558	1	1	0	0	0	0	0	0	0	0	0	0	0	Proteobacteria	Deltaproteobacteria	Bdellovibrionales	Bacteriovoracaceae	Bacteriovorax	Unassigned
1559	1	1	0	0	0	0	0	0	0	0	0	0	0	Proteobacteria	Gammaproteobacteria	Legionellales	Unassigned	Unassigned	Unassigned
1561	1	1	0	0	0	0	0	0	0	0	0	0	0	Proteobacteria	Gammaproteobacteria	Legionellales	Unassigned	Unassigned	Unassigned
1562	0	0	0	0	0	0	0	0	0	0	0	0	0	Proteobacteria	Alphaproteobacteria	Rickettsiales	Rickettsiaceae	Unassigned	Unassigned
1563	1	0	0	0	0	0	0	0	0	0	0	1	0	Proteobacteria	Alphaproteobacteria	Unassigned	Unassigned	Unassigned	Unassigned
1564	1	1	0	0	0	0	0	0	0	0	0	0	0	Proteobacteria	Deltaproteobacteria	Myxococcales	Nannocystaceae	Plesiocystis	Unassigned
1565	1	1	0	0	0	0	0	0	0	0	0	0	0	Bacteroidetes	Cytophagia	Cytophagales	Cyclobacteriaceae	Unassigned	Unassigned
1566	1	0	0	0	0	0	0	0	0	0	0	1	0	Proteobacteria	Gammaproteobacteria	Thiotrichales	Thiotrichaceae	Unassigned	Unassigned
1567	1	1	0	0	0	0	0	0	0	0	0	0	0	Proteobacteria	Gammaproteobacteria	Legionellales	Coxiellaceae	Unassigned	Unassigned
1568	1	0	0	0	0	0	0	0	0	0	0	1	0	Chloroflexi	Anaerolineae	SBR1031	SHA-31	Unassigned	Unassigned
1569	1	0	0	0	0	0	0	0	0	0	0	1	0	Bacteroidetes	[Saprospirae]	[Saprospirales]	Saprospiraceae	Unassigned	Unassigned
1570	1	0	0	0	0	0	0	0	0	0	0	1	0	Bacteroidetes	Bacteroidia	Bacteroidales	Unassigned	Unassigned	Unassigned
1573	1	0	0	0	0	0	0	0	0	0	1	0	0	Planctomycetes	OM190	agg27	Unassigned	Unassigned	Unassigned
1574	1	0	1	0	0	0	0	0	0	0	0	0	0	Firmicutes	Clostridia	Clostridiales	Ruminococcaceae	Unassigned	Unassigned
1575	1	0	0	0	0	0	0	0	0	1	0	0	0	Proteobacteria	Deltaproteobacteria	Bdellovibrionales	Bacteriovoracaceae	Bacteriovorax	Unassigned
1577	0	0	0	0	0	0	0	0	0	0	0	0	0	Proteobacteria	Gammaproteobacteria	Legionellales	Unassigned	Unassigned	Unassigned
1578	1	0	0	0	0	0	0	0	0	0	0	1	0	Proteobacteria	Gammaproteobacteria	Oceanospirillales	Unassigned	Unassigned	Unassigned
1579	0	0	0	0	0	0	0	0	0	0	0	0	0	Proteobacteria	Unassigned	Unassigned	Unassigned	Unassigned	Unassigned
1580	0	0	0	0	0	0	0	0	0	0	0	0	0	SR1	Unassigned	Unassigned	Unassigned	Unassigned	Unassigned
1581	0	0	0	0	0	0	0	0	0	0	0	0	0	Proteobacteria	Gammaproteobacteria	Alteromonadales	OM60	Congregibacter	Unassigned
1582	0	0	0	0	0	0	0	0	0	0	0	0	0	Bacteroidetes	Flavobacteriia	Flavobacteriales	Cryomorphaceae	Owenweeksia	Unassigned
1583	1	0	0	0	0	0	0	0	0	0	0	1	0	Acidobacteria	Sva0725	Sva0725	Unassigned	Unassigned	Unassigned
1584	1	0	0	0	0	0	0	0	0	0	0	1	0	Proteobacteria	Gammaproteobacteria	Chromatiales	Unassigned	Unassigned	Unassigned
1586	1	0	0	1	0	0	0	0	0	0	0	0	0	Proteobacteria	Gammaproteobacteria	Legionellales	Unassigned	Unassigned	Unassigned
1588	1	0	0	0	0	0	0	0	0	0	1	0	0	Proteobacteria	Deltaproteobacteria	Unassigned	Unassigned	Unassigned	Unassigned
1589	1	0	0	1	0	0	0	0	0	0	0	0	0	Proteobacteria	Deltaproteobacteria	Unassigned	Unassigned	Unassigned	Unassigned
1590	1	0	0	1	0	0	0	0	0	0	0	0	0	Bacteroidetes	[Saprospirae]	[Saprospirales]	Saprospiraceae	Unassigned	Unassigned
1591	1	0	0	1	0	0	0	0	0	0	0	0	0	Chloroflexi	Anaerolineae	GCA004	Unassigned	Unassigned	Unassigned
1592	1	0	0	1	0	0	0	0	0	0	0	0	0	Actinobacteria	Acidimicrobiia	Acidimicrobiales	TK06	Unassigned	Unassigned
1593	1	0	0	1	0	0	0	0	0	0	0	0	0	Acidobacteria	Sva0725	Sva0725	Unassigned	Unassigned	Unassigned
1594	1	0	0	1	0	0	0	0	0	0	0	0	0	Actinobacteria	Acidimicrobiia	Acidimicrobiales	Unassigned	Unassigned	Unassigned
1595	1	0	0	1	0	0	0	0	0	0	0	0	0	Acidobacteria	Sva0725	Sva0725	Unassigned	Unassigned	Unassigned
1597	1	0	0	1	0	0	0	0	0	0	0	0	0	GN02	GKS2-174	Unassigned	Unassigned	Unassigned	Unassigned
1598	0	0	0	0	0	0	0	0	0	0	0	0	0	Proteobacteria	Alphaproteobacteria	Rhizobiales	Unassigned	Unassigned	Unassigned
1600	0	0	0	0	0	0	0	0	0	0	0	0	0	Bacteroidetes	Flavobacteriia	Flavobacteriales	Cryomorphaceae	Crocinitomix	Unassigned

1601	0	0	0	0	0	0	0	0	0	0	0	0	Bacteroidetes	Bacteroidia	Bacteroidales	SB-1	Unassigned	Unassigned
1602	0	0	0	0	0	0	0	0	0	0	0	0	Proteobacteria	Deltaproteobacteria	Bdellovibrionales	Bacteriovoracaceae	Unassigned	Unassigned
1603	1	0	0	1	0	0	0	0	0	0	0	0	Actinobacteria	Acidimicrobiia	Acidimicrobiales	EB1017	Unassigned	Unassigned
1604	1	0	0	1	0	0	0	0	0	0	0	0	Proteobacteria	Deltaproteobacteria	Myxococcales	Cystobacterineae	Unassigned	Unassigned
1605	1	0	0	1	0	0	0	0	0	0	0	0	Proteobacteria	Gammaproteobacteria	Alteromonadales	Alteromonadaceae	Teredinibacter	turnerae
1608	1	0	0	1	0	0	0	0	0	0	0	0	Proteobacteria	Deltaproteobacteria	Myxococcales	Unassigned	Unassigned	Unassigned
1611	1	0	0	1	0	0	0	0	0	0	0	0	Proteobacteria	Alphaproteobacteria	Rhodospirillales	Unassigned	Unassigned	Unassigned
1612	1	0	0	1	0	0	0	0	0	0	0	0	Chloroflexi	Thermomicrobia	AKYG1722	Unassigned	Unassigned	Unassigned
1613	1	0	0	1	0	0	0	0	0	0	0	0	Proteobacteria	Gammaproteobacteria	Chromatiales	Unassigned	Unassigned	Unassigned
1615	1	0	0	1	0	0	0	0	0	0	0	0	Bacteroidetes	Flavobacteriia	Flavobacteriales	Unassigned	Unassigned	Unassigned
1616	1	0	0	1	0	0	0	0	0	0	0	0	Proteobacteria	Gammaproteobacteria	Alteromonadales	Alteromonadaceae	Glaciecola	Unassigned
1618	1	0	0	1	0	0	0	0	0	0	0	0	Proteobacteria	Deltaproteobacteria	Bdellovibrionales	Bacteriovoracaceae	Unassigned	Unassigned
1619	1	0	0	1	0	0	0	0	0	0	0	0	Proteobacteria	Gammaproteobacteria	Unassigned	Unassigned	Unassigned	Unassigned
1621	1	0	0	1	0	0	0	0	0	0	0	0	TM7	TM7-1	Unassigned	Unassigned	Unassigned	Unassigned
1622	1	0	0	1	0	0	0	0	0	0	0	0	Proteobacteria	Gammaproteobacteria	Thiohalorhabdales	Unassigned	Unassigned	Unassigned
1623	1	0	0	0	0	1	0	0	0	0	0	0	TM6	SJA-4	S1198	Unassigned	Unassigned	Unassigned
1624	1	0	0	0	0	1	0	0	0	0	0	0	Proteobacteria	Alphaproteobacteria	Rhodobacterales	Rhodobacteraceae	Unassigned	Unassigned
1625	0	0	0	0	0	0	0	0	0	0	0	0	Bacteroidetes	Flavobacteriia	Flavobacteriales	Cryomorphaceae	Unassigned	Unassigned
1626	0	0	0	0	0	0	0	0	0	0	0	0	Proteobacteria	Gammaproteobacteria	HOC36	Unassigned	Unassigned	Unassigned
1628	1	0	0	0	0	1	0	0	0	0	0	0	Proteobacteria	Deltaproteobacteria	Bdellovibrionales	Bacteriovoracaceae	Bacteriovorax	Unassigned
1629	1	0	0	0	0	0	0	1	0	0	0	0	Chloroflexi	Anaerolineae	Anaerolineales	Anaerolinaceae	SHD-231	Unassigned
1630	1	0	0	0	0	0	0	1	0	0	0	0	Gemmatimonadetes	Gemm-2	Unassigned	Unassigned	Unassigned	Unassigned
1633	1	0	0	0	0	1	0	0	0	0	0	0	Proteobacteria	Gammaproteobacteria	Unassigned	Unassigned	Unassigned	Unassigned
1634	1	0	0	0	0	1	0	0	0	0	0	0	Proteobacteria	Alphaproteobacteria	Rickettsiales	Unassigned	Unassigned	Unassigned
1635	1	0	0	0	0	1	0	0	0	0	0	0	Proteobacteria	Deltaproteobacteria	Myxococcales	Nannocystaceae	Plesiocystis	Unassigned
1636	1	0	0	0	0	1	0	0	0	0	0	0	Proteobacteria	Epsilonproteobacteria	Campylobacterales	Unassigned	Unassigned	Unassigned
1637	1	0	0	0	0	1	0	0	0	0	0	0	Proteobacteria	Gammaproteobacteria	Legionellales	Unassigned	Unassigned	Unassigned
1638	1	0	0	0	0	0	0	1	0	0	0	0	Proteobacteria	Gammaproteobacteria	Salinisphaerales	Unassigned	Unassigned	Unassigned
1639	1	0	0	0	0	0	0	1	0	0	0	0	Proteobacteria	Deltaproteobacteria	Myxococcales	Polyangiaceae	Unassigned	Unassigned
1640	1	0	0	0	0	0	0	1	0	0	0	0	Proteobacteria	Deltaproteobacteria	GMD14H09	Unassigned	Unassigned	Unassigned
1641	1	0	0	0	0	1	0	0	0	0	0	0	Bacteroidetes	[Rhodothermi]	[Rhodothermales]	[Balneolaceae]	Balneola	Unassigned
1642	1	0	0	0	0	0	0	0	0	0	1	0	Firmicutes	Clostridia	Clostridiales	Unassigned	Unassigned	Unassigned
1643	1	0	0	0	0	0	0	0	0	1	0	0	Firmicutes	Clostridia	Clostridiales	Unassigned	Unassigned	Unassigned
1644	1	0	0	0	0	0	0	0	0	0	0	1	Proteobacteria	Alphaproteobacteria	Rickettsiales	Unassigned	Unassigned	Unassigned
1645	1	0	0	0	0	0	0	0	0	0	0	1	Proteobacteria	Gammaproteobacteria	Alteromonadales	OM60	Unassigned	Unassigned
1646	1	0	0	0	0	0	0	0	0	0	0	1	Actinobacteria	Actinobacteria	Actinomycetales	Unassigned	Unassigned	Unassigned
1647	1	0	0	0	0	0	0	0	0	0	0	1	Proteobacteria	Alphaproteobacteria	BD7-3	Unassigned	Unassigned	Unassigned
1648	1	0	0	0	0	0	0	0	0	0	0	1	Proteobacteria	Deltaproteobacteria	GMD14H09	Unassigned	Unassigned	Unassigned
1649	1	0	0	0	0	0	0	0	0	0	0	1	Proteobacteria	Deltaproteobacteria	Myxococcales	Haliangiaceae	Haliangium	Unassigned
1650	1	0	0	0	0	0	0	0	0	0	0	1	Chlorobi	Unassigned	Unassigned	Unassigned	Unassigned	Unassigned
1651	1	0	0	0	0	0	0	0	0	0	0	1	Proteobacteria	Alphaproteobacteria	Rhizobiales	Hyphomicrobiaceae	Unassigned	Unassigned
1652	1	0	0	0	0	0	0	0	0	0	0	1	Proteobacteria	Deltaproteobacteria	Myxococcales	Unassigned	Unassigned	Unassigned
1654	1	0	0	0	0	0	0	0	0	0	0	1	Bacteroidetes	[Rhodothermi]	[Rhodothermales]	[Balneolaceae]	KSA1	Unassigned
1655	1	0	1	0	0	0	0	0	0	0	0	0	Proteobacteria	Gammaproteobacteria	Oceanospirillales	Unassigned	Unassigned	Unassigned
1656	0	0	0	0	0	0	0	0	0	0	0	0	Proteobacteria	Gammaproteobacteria	Vibrionales	Vibrionaceae	Unassigned	Unassigned

1658	1	0	0	1	0	0	0	0	0	0	0	0	0	Verrucomicrobia	Verrucomicrobiae	Verrucomicrobiales	Verrucomicrobiaceae	MSBL3	Unassigned
1659	0	0	0	0	0	0	0	0	0	0	0	0	0	Firmicutes	Clostridia	Clostridiales	Unassigned	Unassigned	Unassigned
1660	0	0	0	0	0	0	0	0	0	0	0	0	0	Proteobacteria	Alphaproteobacteria	Rickettsiales	Unassigned	Unassigned	Unassigned
1661	1	0	0	1	0	0	0	0	0	0	0	0	0	Proteobacteria	Gammaproteobacteria	Xanthomonadales	Xanthomonadaceae	Thermomonas	Unassigned
1662	1	0	0	0	0	0	0	1	0	0	0	0	0	Proteobacteria	Deltaproteobacteria	Unassigned	Unassigned	Unassigned	Unassigned
1663	0	0	0	0	0	0	0	0	0	0	0	0	0	Proteobacteria	Unassigned	Unassigned	Unassigned	Unassigned	Unassigned
1664	0	0	0	0	0	0	0	0	0	0	0	0	0	Bacteroidetes	Flavobacteriia	Flavobacteriales	Unassigned	Unassigned	Unassigned
1665	0	0	0	0	0	0	0	0	0	0	0	0	0	Proteobacteria	Gammaproteobacteria	Salinisphaerales	Unassigned	Unassigned	Unassigned
1666	1	0	0	0	0	0	0	0	0	0	0	1	0	Proteobacteria	Gammaproteobacteria	Legionellales	Unassigned	Unassigned	Unassigned
1669	1	0	0	0	0	0	0	0	0	0	0	1	0	Proteobacteria	Alphaproteobacteria	Rickettsiales	Rickettsiaceae	Unassigned	Unassigned
1671	0	0	0	0	0	0	0	0	0	0	0	0	0	OD1	Mb-NB09	Unassigned	Unassigned	Unassigned	Unassigned
1672	1	0	0	0	0	0	0	0	0	0	0	1	0	Proteobacteria	Deltaproteobacteria	Sva0853	JTB36	Unassigned	Unassigned
1673	1	0	0	0	0	0	0	0	0	0	0	1	0	Proteobacteria	Alphaproteobacteria	Sphingomonadales	Erythrobacteraceae	Unassigned	Unassigned
1674	0	0	0	0	0	0	0	0	0	0	0	0	0	Proteobacteria	Alphaproteobacteria	BD7-3	Unassigned	Unassigned	Unassigned
1675	0	0	0	0	0	0	0	0	0	0	0	0	0	Proteobacteria	Gammaproteobacteria	Alteromonadales	Unassigned	Unassigned	Unassigned
1676	0	0	0	0	0	0	0	0	0	0	0	0	0	Proteobacteria	Alphaproteobacteria	BD7-3	Unassigned	Unassigned	Unassigned
1677	0	0	0	0	0	0	0	0	0	0	0	0	0	Verrucomicrobia	Verrucomicrobiae	Verrucomicrobiales	Verrucomicrobiaceae	Luteolibacter	Unassigned
1678	0	0	0	0	0	0	0	0	0	0	0	0	0	Proteobacteria	Gammaproteobacteria	Legionellales	Coxiellaceae	Aquicella	Unassigned
1680	1	0	0	0	0	0	0	0	0	0	0	1	0	Bacteroidetes	Flavobacteriia	Flavobacteriales	Flavobacteriaceae	Gramella	Unassigned
1681	1	0	0	0	0	0	0	0	0	0	0	1	0	Proteobacteria	Deltaproteobacteria	Desulfobacterales	Desulfobulbaceae	Unassigned	Unassigned
1683	1	0	0	0	0	0	0	0	0	0	0	1	0	Proteobacteria	Gammaproteobacteria	[Marinicellales]	[Marinicellaceae]	Unassigned	Unassigned
1684	0	0	0	0	0	0	0	0	0	0	0	0	0	Proteobacteria	Gammaproteobacteria	Legionellales	Francisellaceae	Unassigned	Unassigned
1685	0	0	0	0	0	0	0	0	0	0	0	0	0	Proteobacteria	Alphaproteobacteria	Rickettsiales	Unassigned	Unassigned	Unassigned
1686	1	0	0	0	0	0	0	0	0	0	1	0	0	Proteobacteria	Gammaproteobacteria	Alteromonadales	Alteromonadaceae	BD2-13	Unassigned
1687	1	0	0	0	0	0	0	0	0	0	1	0	0	WS3	PRR-12	GN03	KSB4	Unassigned	Unassigned
1689	1	0	0	0	0	0	0	0	0	0	1	0	0	Proteobacteria	Deltaproteobacteria	Bdellovibrionales	Bdellovibrionaceae	Bdellovibrio	Unassigned
1690	1	1	0	0	0	0	0	0	0	0	0	0	0	Proteobacteria	Gammaproteobacteria	Alteromonadales	[Chromatiaceae]	Unassigned	Unassigned
1691	1	1	0	0	0	0	0	0	0	0	0	0	0	Proteobacteria	Gammaproteobacteria	Oceanospirillales	Oceanospirillaceae	Unassigned	Unassigned
1692	1	1	0	0	0	0	0	0	0	0	0	0	0	Proteobacteria	Betaproteobacteria	Burkholderiales	Comamonadaceae	Variovorax	paradoxus
1693	1	0	0	0	0	0	0	0	0	1	0	0	0	Proteobacteria	Deltaproteobacteria	Desulfovibrionales	Desulfovibrionaceae	Desulfovibrio	Unassigned
1694	1	1	0	0	0	0	0	0	0	0	0	0	0	Actinobacteria	Acidimicrobiia	Acidimicrobiales	koll13	Unassigned	Unassigned
1695	1	1	0	0	0	0	0	0	0	0	0	0	0	Proteobacteria	Deltaproteobacteria	Bdellovibrionales	Bacteriovoracaceae	Unassigned	Unassigned
1697	1	1	0	0	0	0	0	0	0	0	0	0	0	Proteobacteria	Alphaproteobacteria	Rhodobacterales	Hyphomonadaceae	Unassigned	Unassigned
1699	1	0	0	0	0	0	0	1	0	0	0	0	0	Proteobacteria	Gammaproteobacteria	Oceanospirillales	Oceanospirillaceae	Unassigned	Unassigned
1700	1	0	0	0	0	0	0	1	0	0	0	0	0	Proteobacteria	Deltaproteobacteria	Desulfobacterales	Desulfobulbaceae	Desulfobulbus	Unassigned
1701	1	1	0	0	0	0	0	0	0	0	0	0	0	Planctomycetes	Phycisphaerae	Phycisphaerales	Unassigned	Unassigned	Unassigned
1704	1	1	0	0	0	0	0	0	0	0	0	0	0	Proteobacteria	Gammaproteobacteria	Unassigned	Unassigned	Unassigned	Unassigned
1705	1	0	0	0	0	0	0	1	0	0	0	0	0	Bacteroidetes	Bacteroidia	Bacteroidales	SB-1	Unassigned	Unassigned
1706	1	0	0	0	0	0	0	1	0	0	0	0	0	TM6	SJA-4	Unassigned	Unassigned	Unassigned	Unassigned
1707	1	0	0	0	0	0	0	1	0	0	0	0	0	WS6	SC72	MAT-CR-H2-G03	Unassigned	Unassigned	Unassigned
1708	1	0	0	0	0	0	0	1	0	0	0	0	0	Bacteroidetes	[Rhodothermi]	[Rhodothermales]	[Balneolaceae]	KSA1	Unassigned
1710	1	0	0	0	0	0	0	1	0	0	0	0	0	Bacteroidetes	Cytophagia	Cytophagales	Cytophagaceae	Pontibacter	Unassigned
1711	1	0	0	0	0	1	0	0	0	0	0	0	0	Acidobacteria	Holophagae	Holophagales	Unassigned	Unassigned	Unassigned
1712	1	0	0	0	0	1	0	0	0	0	0	0	0	TM7	TM7-1	Unassigned	Unassigned	Unassigned	Unassigned
1713	1	0	0	0	0	0	0	0	1	0	0	0	0	Proteobacteria	Gammaproteobacteria	Alteromonadales	Alteromonadaceae	Marinobacter	Unassigned

1714	1	0	0	0	0	0	0	0	1	0	0	0	0	Chloroflexi	Anaerolineae	SBR1031	A4b	Unassigned	Unassigned
1715	1	0	0	1	0	0	0	0	0	0	0	0	0	Proteobacteria	Deltaproteobacteria	Myxococcales	Haliangiaceae	Haliangium	Unassigned
1717	1	0	0	1	0	0	0	0	0	0	0	0	0	Acidobacteria	[Chloracidobacteria]	RB41	Ellin6075	Unassigned	Unassigned
1718	1	0	0	1	0	0	0	0	0	0	0	0	0	Actinobacteria	Acidimicrobiia	Acidimicrobiales	koll13	Unassigned	Unassigned
1719	1	0	0	1	0	0	0	0	0	0	0	0	0	Proteobacteria	Alphaproteobacteria	Rhodospirillales	Unassigned	Unassigned	Unassigned
1720	0	0	0	0	0	0	0	0	0	0	0	0	0	Proteobacteria	Gammaproteobacteria	Vibrionales	Vibrionaceae	Vibrio	Unassigned
1721	1	0	0	0	0	0	0	0	0	0	0	1	0	Gemmatimonadetes	Gemm-2	Unassigned	Unassigned	Unassigned	Unassigned
1722	1	0	0	0	0	0	0	0	0	0	0	1	0	Acidobacteria	Holophagae	Holophagales	Unassigned	Unassigned	Unassigned
1723	0	0	0	0	0	0	0	0	0	0	0	0	0	Proteobacteria	Deltaproteobacteria	Spirobacillales	Unassigned	Unassigned	Unassigned
1724	0	0	0	0	0	0	0	0	0	0	0	0	0	Proteobacteria	Gammaproteobacteria	Legionellales	Unassigned	Unassigned	Unassigned
1725	1	0	0	0	0	0	1	0	0	0	0	0	0	Proteobacteria	Deltaproteobacteria	Spirobacillales	Unassigned	Unassigned	Unassigned
1727	1	0	0	1	0	0	0	0	0	0	0	0	0	Bacteroidetes	Flavobacteriia	Flavobacteriales	Unassigned	Unassigned	Unassigned
1730	1	0	0	1	0	0	0	0	0	0	0	0	0	Proteobacteria	Gammaproteobacteria	[Marinicellales]	[Marinicellaceae]	Unassigned	Unassigned
1731	1	0	0	1	0	0	0	0	0	0	0	0	0	Acidobacteria	RB25	Unassigned	Unassigned	Unassigned	Unassigned
1732	1	0	0	1	0	0	0	0	0	0	0	0	0	Proteobacteria	Deltaproteobacteria	NB1-j	Unassigned	Unassigned	Unassigned
1733	1	0	0	1	0	0	0	0	0	0	0	0	0	Proteobacteria	Gammaproteobacteria	Unassigned	Unassigned	Unassigned	Unassigned
1734	0	0	0	0	0	0	0	0	0	0	0	0	0	Proteobacteria	Alphaproteobacteria	Rickettsiales	Rickettsiaceae	Unassigned	Unassigned
1735	0	0	0	0	0	0	0	0	0	0	0	0	0	Bacteroidetes	Flavobacteriia	Flavobacteriales	Cryomorphaceae	Owenweeksia	Unassigned
1736	1	0	0	1	0	0	0	0	0	0	0	0	0	Bacteroidetes	Flavobacteriia	Flavobacteriales	Cryomorphaceae	Unassigned	Unassigned
1737	1	0	0	0	0	0	0	0	0	0	0	0	1	Proteobacteria	Alphaproteobacteria	Unassigned	Unassigned	Unassigned	Unassigned
1738	1	0	0	1	0	0	0	0	0	0	0	0	0	Bacteroidetes	Cytophagia	Cytophagales	Flammeovirgaceae	Flexithrix	Unassigned
1739	1	0	0	1	0	0	0	0	0	0	0	0	0	Acidobacteria	Sva0725	Sva0725	Unassigned	Unassigned	Unassigned
1741	1	0	0	0	0	0	0	0	0	0	0	0	1	Proteobacteria	Deltaproteobacteria	Myxococcales	Unassigned	Unassigned	Unassigned
1743	1	0	0	0	0	0	0	0	0	0	0	0	1	Bacteroidetes	[Saprospirae]	[Saprospirales]	Chitinophagaceae	Unassigned	Unassigned
1744	1	0	0	0	0	0	0	0	0	0	0	1	0	Chloroflexi	Anaerolineae	envOPS12	Unassigned	Unassigned	Unassigned
1745	1	0	0	0	0	0	0	0	0	0	0	1	0	Actinobacteria	Acidimicrobiia	Acidimicrobiales	Unassigned	Unassigned	Unassigned
1746	1	0	0	0	0	0	0	0	0	0	0	1	0	Bacteroidetes	[Rhodothermi]	[Rhodothermales]	Rhodothermaceae	Rubricoccus	Unassigned
1747	1	0	0	0	0	0	0	0	0	0	0	1	0	Proteobacteria	Alphaproteobacteria	Kordiimonadales	Unassigned	Unassigned	Unassigned
1748	1	0	1	0	0	0	0	0	0	0	0	0	0	Proteobacteria	Alphaproteobacteria	Unassigned	Unassigned	Unassigned	Unassigned
1750	1	0	0	0	0	0	0	0	0	0	0	1	0	Actinobacteria	Actinobacteria	Unassigned	Unassigned	Unassigned	Unassigned
1751	1	0	0	0	0	0	0	0	0	0	0	1	0	Bacteroidetes	Bacteroidia	Bacteroidales	SB-1	Unassigned	Unassigned
1752	0	0	0	0	0	0	0	0	0	0	0	0	0	GN02	3BR-5F	Unassigned	Unassigned	Unassigned	Unassigned
1753	1	0	0	0	0	0	0	0	0	0	0	1	0	Chloroflexi	Anaerolineae	SBR1031	A4b	Unassigned	Unassigned
1754	1	0	0	0	0	0	0	0	0	0	0	1	0	Bacteroidetes	Cytophagia	Cytophagales	Unassigned	Unassigned	Unassigned
1755	1	0	0	0	0	0	0	0	0	0	0	1	0	Cyanobacteria	Oscillatoriophycideae	Chroococcales	Unassigned	Unassigned	Unassigned
1756	1	0	0	0	0	0	0	0	0	0	0	1	0	Proteobacteria	Gammaproteobacteria	Alteromonadales	OM60	Unassigned	Unassigned
1757	1	0	0	0	0	0	0	0	0	0	0	1	0	Acidobacteria	[Chloracidobacteria]	RB41	Ellin6075	Unassigned	Unassigned
1760	1	0	0	0	0	0	0	0	0	0	0	1	0	Proteobacteria	Deltaproteobacteria	PB19	Unassigned	Unassigned	Unassigned
1761	1	0	0	0	0	0	0	0	0	0	0	1	0	Proteobacteria	Deltaproteobacteria	Desulfobacterales	Desulfobulbaceae	Unassigned	Unassigned
1762	1	0	0	0	0	0	0	0	0	0	0	1	0	Proteobacteria	Deltaproteobacteria	Desulfobacterales	Desulfobulbaceae	Unassigned	Unassigned
1763	1	0	0	0	0	0	0	0	0	0	0	1	0	Bacteroidetes	[Saprospirae]	[Saprospirales]	Saprospiraceae	Unassigned	Unassigned
1764	0	0	0	0	0	0	0	0	0	0	0	0	0	Proteobacteria	Alphaproteobacteria	Rhodospirillales	Rhodospirillaceae	Novispirillum	Unassigned
1765	0	0	0	0	0	0	0	0	0	0	0	0	0	Chlorobi	OPB56	Unassigned	Unassigned	Unassigned	Unassigned
1766	0	0	0	0	0	0	0	0	0	0	0	0	0	Proteobacteria	Deltaproteobacteria	Bdellovibrionales	Bacteriovoracaceae	Bacteriovorax	Unassigned
1769	1	0	0	0	0	0	0	0	0	1	0	0	0	Proteobacteria	Unassigned	Unassigned	Unassigned	Unassigned	Unassigned

1771	0	0	0	0	0	0	0	0	0	0	0	0	Proteobacteria	Alphaproteobacteria	Rickettsiales	Rickettsiaceae	Unassigned	Unassigned
1773	0	0	0	0	0	0	0	0	0	0	0	0	Proteobacteria	Deltaproteobacteria	Myxococcales	OM27	Unassigned	Unassigned
1774	1	0	1	0	0	0	0	0	0	0	0	0	Proteobacteria	Alphaproteobacteria	Kiloniellales	Unassigned	Unassigned	Unassigned
1775	1	1	0	0	0	0	0	0	0	0	0	0	Proteobacteria	Deltaproteobacteria	Myxococcales	Unassigned	Unassigned	Unassigned
1776	1	0	0	0	0	0	0	0	0	1	0	0	Proteobacteria	Deltaproteobacteria	Desulfobacterales	Desulfobacteraceae	Desulfobacter	Unassigned
1777	1	1	0	0	0	0	0	0	0	0	0	0	Proteobacteria	Gammaproteobacteria	Thiotrichales	Piscirickettsiaceae	Unassigned	Unassigned
1778	1	1	0	0	0	0	0	0	0	0	0	0	Chloroflexi	Anaerolineae	Caldilineales	Caldilineaceae	Unassigned	Unassigned
1779	0	0	0	0	0	0	0	0	0	0	0	0	TM6	SJA-4	Unassigned	Unassigned	Unassigned	Unassigned
1780	0	0	0	0	0	0	0	0	0	0	0	0	Proteobacteria	Alphaproteobacteria	Rickettsiales	Unassigned	Unassigned	Unassigned
1781	1	0	0	0	0	0	0	0	0	0	1	0	Proteobacteria	Gammaproteobacteria	Alteromonadales	Unassigned	Unassigned	Unassigned
1782	1	0	0	0	0	0	0	0	0	0	1	0	Bacteroidetes	Flavobacteriia	Flavobacteriales	Unassigned	Unassigned	Unassigned
1783	1	1	0	0	0	0	0	0	0	0	0	0	TM7	Unassigned	Unassigned	Unassigned	Unassigned	Unassigned
1784	1	1	0	0	0	0	0	0	0	0	0	0	Bacteroidetes	[Saprospirae]	[Saprospirales]	Saprospiraceae	Unassigned	Unassigned
1785	1	1	0	0	0	0	0	0	0	0	0	0	Verrucomicrobia	Verrucomicrobiae	Verrucomicrobiales	Verrucomicrobiaceae	Unassigned	Unassigned
1787	1	0	0	0	0	0	0	0	0	0	1	0	Proteobacteria	Alphaproteobacteria	Rickettsiales	Rickettsiaceae	Unassigned	Unassigned
1788	0	0	0	0	0	0	0	0	0	0	0	0	Proteobacteria	Unassigned	Unassigned	Unassigned	Unassigned	Unassigned
1789	1	0	0	0	0	1	0	0	0	0	0	0	Bacteroidetes	Bacteroidia	Bacteroidales	Porphyromonadaceae	Dysgonomonas	Unassigned
1790	1	1	0	0	0	0	0	0	0	0	0	0	Bacteroidetes	Flavobacteriia	Flavobacteriales	Unassigned	Unassigned	Unassigned
1791	1	0	0	0	0	0	0	0	1	0	0	0	Bacteroidetes	Cytophagia	Cytophagales	Flammeovirgaceae	Unassigned	Unassigned
1792	1	0	0	0	0	0	0	0	0	1	0	0	WS3	PRR-12	GN03	KSB4	Unassigned	Unassigned
1794	1	1	0	0	0	0	0	0	0	0	0	0	Proteobacteria	Alphaproteobacteria	Rhizobiales	Unassigned	Unassigned	Unassigned
1795	0	0	0	0	0	0	0	0	0	0	0	0	Proteobacteria	Gammaproteobacteria	Oceanospirillales	Oceanospirillaceae	Marinobacterium	Unassigned
1796	0	0	0	0	0	0	0	0	0	0	0	0	Proteobacteria	Alphaproteobacteria	Unassigned	Unassigned	Unassigned	Unassigned
1798	0	0	0	0	0	0	0	0	0	0	0	0	Proteobacteria	Gammaproteobacteria	Alteromonadales	Unassigned	Unassigned	Unassigned
1799	0	0	0	0	0	0	0	0	0	0	0	0	Proteobacteria	Gammaproteobacteria	Thiotrichales	Piscirickettsiaceae	Thiomicrospira	frisia
1800	1	0	0	0	1	0	0	0	0	0	0	0	Proteobacteria	Deltaproteobacteria	Myxococcales	Haliangiaceae	Unassigned	Unassigned
1801	0	0	0	0	0	0	0	0	0	0	0	0	Proteobacteria	Gammaproteobacteria	Unassigned	Unassigned	Unassigned	Unassigned
1802	0	0	0	0	0	0	0	0	0	0	0	0	Chloroflexi	Anaerolineae	SBR1031	SJA-101	Unassigned	Unassigned
1804	1	0	0	0	0	0	1	0	0	0	0	0	Verrucomicrobia	Opitutae	[Pelagicoccales]	[Pelagicoccaceae]	Pelagicoccus	Unassigned
1805	1	0	0	0	0	0	1	0	0	0	0	0	WS3	PRR-12	GN03	KSB4	Unassigned	Unassigned
1806	1	0	0	0	0	0	1	0	0	0	0	0	[Thermi]	Deinococci	Deinococcales	Trueperaceae	B-42	Unassigned
1807	0	0	0	0	0	0	0	0	0	0	0	0	Proteobacteria	Gammaproteobacteria	Vibrionales	Vibrionaceae	Vibrio	Unassigned
1808	0	0	0	0	0	0	0	0	0	0	0	0	Proteobacteria	Alphaproteobacteria	RF32	Unassigned	Unassigned	Unassigned
1809	0	0	0	0	0	0	0	0	0	0	0	0	Proteobacteria	Alphaproteobacteria	Unassigned	Unassigned	Unassigned	Unassigned
1810	0	0	0	0	0	0	0	0	0	0	0	0	Proteobacteria	Gammaproteobacteria	Alteromonadales	Alteromonadaceae	Glaciecola	Unassigned
1811	0	0	0	0	0	0	0	0	0	0	0	0	OP11	WCHB1-64	Unassigned	Unassigned	Unassigned	Unassigned
1812	1	0	0	0	0	0	0	0	0	1	0	0	Proteobacteria	Deltaproteobacteria	Bdellovibrionales	Bacteriovoracaceae	Unassigned	Unassigned
1813	1	0	0	0	0	0	0	0	0	0	1	0	Acidobacteria	Acidobacteria-6	CCU21	Unassigned	Unassigned	Unassigned
1814	1	0	0	0	0	0	0	0	0	0	1	0	Proteobacteria	Gammaproteobacteria	Enterobacteriales	Enterobacteriaceae	CandidatusPhlomobact	Unassigned
1815	1	0	0	0	0	0	0	0	0	0	1	0	Proteobacteria	Deltaproteobacteria	Spirobacillales	Unassigned	Unassigned	Unassigned
1816	1	0	0	0	0	0	0	0	0	0	1	0	Gemmatimonadete	Gemm-4	Unassigned	Unassigned	Unassigned	Unassigned
1817	0	0	0	0	0	0	0	0	0	0	0	0	OP8	OP8_2	Unassigned	Unassigned	Unassigned	Unassigned
1818	0	0	0	0	0	0	0	0	0	0	0	0	Proteobacteria	Gammaproteobacteria	Alteromonadales	Alteromonadaceae	CandidatusEndobugula	Unassigned
1819	0	0	0	0	0	0	0	0	0	0	0	0	Bacteroidetes	Flavobacteriia	Flavobacteriales	Cryomorphaceae	Unassigned	Unassigned
1820	0	0	0	0	0	0	0	0	0	0	0	0	Proteobacteria	Deltaproteobacteria	Bdellovibrionales	Bacteriovoracaceae	Unassigned	Unassigned

1821	0	0	0	0	0	0	0	0	0	0	0	0	0	Verrucomicrobia	Verrucomicrobiae	Verrucomicrobiales	Verrucomicrobiaceae	Luteolibacter	Unassigned
1823	1	0	0	0	0	0	0	1	0	0	0	0	0	Proteobacteria	Alphaproteobacteria	Rhodobacterales	Rhodobacteraceae	Unassigned	Unassigned
1824	1	0	0	0	0	0	0	1	0	0	0	0	0	Proteobacteria	Gammaproteobacteria	Unassigned	Unassigned	Unassigned	Unassigned
1825	1	0	0	0	0	0	0	1	0	0	0	0	0	Chloroflexi	Anaerolineae	SBR1031	Unassigned	Unassigned	Unassigned
1826	1	0	0	0	0	0	0	1	0	0	0	0	0	Proteobacteria	Gammaproteobacteria	Thiotrichales	Piscirickettsiaceae	Methylophaga	Unassigned
1827	1	0	0	0	0	0	0	1	0	0	0	0	0	WS3	PRR-12	GN03	KSB4	Unassigned	Unassigned
1828	1	0	0	0	0	0	0	1	0	0	0	0	0	Chlorobi	OPB56	Unassigned	Unassigned	Unassigned	Unassigned
1829	1	0	0	0	0	0	0	1	0	0	0	0	0	Firmicutes	Bacilli	Bacillales	Alicyclobacillaceae	Alicyclobacillus	Unassigned
1830	0	0	0	0	0	0	0	0	0	0	0	0	0	Proteobacteria	Alphaproteobacteria	Unassigned	Unassigned	Unassigned	Unassigned
1832	0	0	0	0	0	0	0	0	0	0	0	0	0	Proteobacteria	Deltaproteobacteria	Unassigned	Unassigned	Unassigned	Unassigned
1833	1	0	0	0	0	1	0	0	0	0	0	0	0	Proteobacteria	Deltaproteobacteria	Unassigned	Unassigned	Unassigned	Unassigned
1836	1	0	0	0	0	0	0	1	0	0	0	0	0	Proteobacteria	Deltaproteobacteria	Spirobacillales	Unassigned	Unassigned	Unassigned
1837	1	0	0	0	0	0	0	1	0	0	0	0	0	Proteobacteria	Gammaproteobacteria	Legionellales	Coxiellaceae	Unassigned	Unassigned
1838	1	0	0	0	0	0	0	1	0	0	0	0	0	Bacteroidetes	Cytophagia	Cytophagales	Cytophagaceae	Unassigned	Unassigned
1839	0	0	0	0	0	0	0	0	0	0	0	0	0	OP8	OP8_2	Unassigned	Unassigned	Unassigned	Unassigned
1840	0	0	0	0	0	0	0	0	0	0	0	0	0	Proteobacteria	Alphaproteobacteria	RF32	Unassigned	Unassigned	Unassigned
1841	0	0	0	0	0	0	0	0	0	0	0	0	0	Bacteroidetes	Bacteroidia	Bacteroidales	Rikenellaceae	Unassigned	Unassigned
1842	0	0	0	0	0	0	0	0	0	0	0	0	0	Proteobacteria	Betaproteobacteria	Burkholderiales	Comamonadaceae	RS62	Unassigned
1844	1	1	0	0	0	0	0	0	0	0	0	0	0	Proteobacteria	Gammaproteobacteria	Legionellales	Coxiellaceae	Unassigned	Unassigned
1846	1	1	0	0	0	0	0	0	0	0	0	0	0	Proteobacteria	Gammaproteobacteria	Oceanospirillales	Halomonadaceae	Kushneria	Unassigned
1848	1	0	0	0	1	0	0	0	0	0	0	0	0	OP11	WCHB1-64	Unassigned	Unassigned	Unassigned	Unassigned
1849	1	0	0	0	1	0	0	0	0	0	0	0	0	Acidobacteria	Holophagae	Holophagales	Unassigned	Unassigned	Unassigned
1850	0	0	0	0	0	0	0	0	0	0	0	0	0	Bacteroidetes	Cytophagia	Cytophagales	Flammeovirgaceae	Flexibacter	Unassigned
1851	1	0	0	0	1	0	0	0	0	0	0	0	0	Bacteroidetes	[Saprospirae]	[Saprospirales]	Saprospiraceae	Lewinella	Unassigned
1852	0	0	0	0	0	0	0	0	0	0	0	0	0	Firmicutes	Clostridia	Clostridiales	[Acidaminobacteraceae]	Fusibacter	Unassigned
1853	1	0	0	0	1	0	0	0	0	0	0	0	0	Proteobacteria	Gammaproteobacteria	Legionellales	Unassigned	Unassigned	Unassigned
1854	1	0	0	0	1	0	0	0	0	0	0	0	0	Proteobacteria	Deltaproteobacteria	Bdellovibrionales	Bdellovibrionaceae	Unassigned	Unassigned
1855	1	0	0	0	0	1	0	0	0	0	0	0	0	Proteobacteria	Alphaproteobacteria	Rickettsiales	Rickettsiaceae	Unassigned	Unassigned
1856	0	0	0	0	0	0	0	0	0	0	0	0	0	Proteobacteria	Alphaproteobacteria	BD7-3	Unassigned	Unassigned	Unassigned
1857	1	0	0	1	0	0	0	0	0	0	0	0	0	Bacteroidetes	[Saprospirae]	[Saprospirales]	Saprospiraceae	Unassigned	Unassigned
1858	1	0	0	1	0	0	0	0	0	0	0	0	0	Acidobacteria	AT-s54	Unassigned	Unassigned	Unassigned	Unassigned
1859	1	0	0	1	0	0	0	0	0	0	0	0	0	Proteobacteria	Deltaproteobacteria	Myxococcales	Unassigned	Unassigned	Unassigned
1860	1	0	0	1	0	0	0	0	0	0	0	0	0	Proteobacteria	Gammaproteobacteria	Oceanospirillales	Halomonadaceae	CandidatusPortiera	Unassigned
1861	1	0	0	0	0	0	0	0	0	0	0	0	1	Proteobacteria	Alphaproteobacteria	BD7-3	Unassigned	Unassigned	Unassigned
1862	1	0	0	0	0	0	0	0	0	0	0	0	1	Actinobacteria	Thermoleophilia	Solirubrobacterales	Unassigned	Unassigned	Unassigned
1863	1	0	0	0	0	0	0	0	0	0	0	0	1	Acidobacteria	RB25	Unassigned	Unassigned	Unassigned	Unassigned
1865	1	0	0	1	0	0	0	0	0	0	0	0	0	Proteobacteria	Gammaproteobacteria	Alteromonadales	Alteromonadaceae	Simiduia	agarivorans
1866	1	0	0	1	0	0	0	0	0	0	0	0	0	Proteobacteria	Gammaproteobacteria	Alteromonadales	HTCC2188	HTCC	Unassigned
1867	1	0	0	1	0	0	0	0	0	0	0	0	0	OP11	WCHB1-64	Unassigned	Unassigned	Unassigned	Unassigned
1868	1	0	0	1	0	0	0	0	0	0	0	0	0	Proteobacteria	Deltaproteobacteria	Myxococcales	Haliangiaceae	Unassigned	Unassigned
1869	1	0	0	1	0	0	0	0	0	0	0	0	0	Proteobacteria	Deltaproteobacteria	Desulfobacterales	Desulfobulbaceae	Unassigned	Unassigned
1870	1	0	0	0	1	0	0	0	0	0	0	0	0	Bacteroidetes	[Rhodothermi]	[Rhodothermales]	Rhodothermaceae	Unassigned	Unassigned
1871	1	0	0	0	1	0	0	0	0	0	0	0	0	OP11	WCHB1-64	d153	Unassigned	Unassigned	Unassigned
1873	1	0	0	0	1	0	0	0	0	0	0	0	0	Proteobacteria	Gammaproteobacteria	Legionellales	Legionellaceae	Unassigned	Unassigned
1874	1	0	0	0	0	0	0	0	0	0	0	0	1	Proteobacteria	Alphaproteobacteria	Rickettsiales	Unassigned	Unassigned	Unassigned

1875	1	0	0	0	0	0	0	0	0	0	0	0	1	Chloroflexi	Thermomicrobia	JG30-KF-CM45	Unassigned	Unassigned	Unassigned
1876	1	0	0	0	1	0	0	0	0	0	0	0	0	Proteobacteria	Alphaproteobacteria	Rhodospirillales	Rhodospirillaceae	Unassigned	Unassigned
1877	1	0	0	0	1	0	0	0	0	0	0	0	0	Proteobacteria	Gammaproteobacteria	Alteromonadales	HTCC2188	Unassigned	Unassigned
1878	1	0	0	0	1	0	0	0	0	0	0	0	0	Proteobacteria	Alphaproteobacteria	Rhodospirillales	Rhodospirillaceae	Unassigned	Unassigned
1879	1	0	0	0	0	0	0	0	0	0	0	0	1	Proteobacteria	Gammaproteobacteria	Legionellales	Legionellaceae	Unassigned	Unassigned
1880	1	0	0	0	0	0	0	0	0	0	0	0	1	Bacteroidetes	Bacteroidia	Bacteroidales	Unassigned	Unassigned	Unassigned
1882	1	0	0	1	0	0	0	0	0	0	0	0	0	Proteobacteria	Deltaproteobacteria	Desulfobacterales	Desulfobulbaceae	Desulfotalea	Unassigned
1883	1	0	0	1	0	0	0	0	0	0	0	0	0	Bacteroidetes	[Rhodothermi]	[Rhodothermales]	Rhodothermaceae	Unassigned	Unassigned
1884	1	0	0	1	0	0	0	0	0	0	0	0	0	Acidobacteria	Sva0725	Sva0725	Unassigned	Unassigned	Unassigned
1885	1	0	0	0	0	0	0	0	1	0	0	0	0	Proteobacteria	Deltaproteobacteria	Myxococcales	Unassigned	Unassigned	Unassigned
1886	1	0	0	0	0	1	0	0	0	0	0	0	0	Proteobacteria	Gammaproteobacteria	[Marinicellales]	[Marinicellaceae]	Unassigned	Unassigned
1887	1	0	0	0	0	0	0	1	0	0	0	0	0	Actinobacteria	Acidimicrobiia	Acidimicrobiales	Iamiaceae	Iamia	Unassigned
1888	1	0	0	0	0	1	0	0	0	0	0	0	0	Proteobacteria	Gammaproteobacteria	Chromatiales	Chromatiaceae	Thiorhodococcus	Unassigned
1889	1	0	0	0	0	0	0	1	0	0	0	0	0	Proteobacteria	Deltaproteobacteria	NB1-j	NB1-i	Unassigned	Unassigned
1890	1	0	0	0	0	1	0	0	0	0	0	0	0	Proteobacteria	Gammaproteobacteria	Legionellales	Coxiellaceae	Unassigned	Unassigned
1891	1	0	0	0	0	0	0	0	1	0	0	0	0	Bacteroidetes	Bacteroidia	Bacteroidales	Unassigned	Unassigned	Unassigned
1892	1	0	0	1	0	0	0	0	0	0	0	0	0	Bacteroidetes	[Saprospirae]	[Saprospirales]	Saprospiraceae	Unassigned	Unassigned
1894	1	0	0	0	0	0	0	0	1	0	0	0	0	Proteobacteria	Gammaproteobacteria	Pseudomonadales	Pseudomonadaceae	Pseudomonas	Unassigned
1896	1	0	0	0	0	0	0	0	1	0	0	0	0	Proteobacteria	Deltaproteobacteria	Myxococcales	Haliangiaceae	Unassigned	Unassigned
1898	1	0	0	0	0	0	0	0	1	0	0	0	0	Bacteroidetes	Flavobacteriia	Flavobacteriales	Unassigned	Unassigned	Unassigned
	12924																		

Table S4. Common OTUs between grow-out environments (sediment vs. aquaponics) of *H. portulacoides* rhizosphere.

OTU	Aquaponic	Sediment	Domain	Phylum	Class	Order	Family	Genus	Species
3	29	2	Bacteria	Proteobacteria	Epsilonproteobacteria	Campylobacterales	Campylobacteraceae	Arcobacter	Unassigned
14	92	7	Bacteria	Proteobacteria	Alphaproteobacteria	Rhodobacterales	Rhodobacteraceae	Loktanella	Unassigned
18	23	17	Bacteria	Proteobacteria	Alphaproteobacteria	Rhizobiales	Phyllobacteriaceae	Unassigned	Unassigned
1169	5	2	Bacteria	Proteobacteria	Alphaproteobacteria	Rhodobacterales	Rhodobacteraceae	Unassigned	Unassigned
17	16	4	Bacteria	Proteobacteria	Alphaproteobacteria	Rhodobacterales	Rhodobacteraceae	Marivita	Unassigned
1507	2	19	Bacteria	Proteobacteria	Alphaproteobacteria	Sphingomonadales	Erythrobacteraceae	Unassigned	Unassigned
26	12	1	Bacteria	Proteobacteria	Epsilonproteobacteria	Campylobacterales	Campylobacteraceae	Arcobacter	Unassigned
92	31	8	Bacteria	Proteobacteria	Alphaproteobacteria	Rhodobacterales	Rhodobacteraceae	Phaeobacter	Unassigned
29	31	18	Bacteria	Proteobacteria	Alphaproteobacteria	Rhizobiales	Phyllobacteriaceae	Unassigned	Unassigned
80	3	38	Bacteria	Proteobacteria	Alphaproteobacteria	Sphingomonadales	Unassigned	Unassigned	Unassigned
25	6	28	Bacteria	Proteobacteria	Deltaproteobacteria	Myxococcales	Unassigned	Unassigned	Unassigned
151	1	7	Bacteria	Proteobacteria	Alphaproteobacteria	Rhodobacterales	Rhodobacteraceae	Unassigned	Unassigned
61	9	22	Bacteria	Proteobacteria	Alphaproteobacteria	Rhizobiales	Hyphomicrobiaceae	Devosia	Unassigned
73	5	4	Bacteria	Proteobacteria	Alphaproteobacteria	Rhodobacterales	Rhodobacteraceae	Jannaschia	Unassigned
93	1	5	Bacteria	Proteobacteria	Gammaproteobacteria	Chromatiales	Unassigned	Unassigned	Unassigned
33	8	2	Bacteria	Proteobacteria	Alphaproteobacteria	Rhizobiales	Hyphomicrobiaceae	Unassigned	Unassigned
31	5	39	Bacteria	Proteobacteria	Alphaproteobacteria	Sphingomonadales	Erythrobacteraceae	Unassigned	Unassigned
202	8	6	Bacteria	Proteobacteria	Alphaproteobacteria	Rhodobacterales	Rhodobacteraceae	Unassigned	Unassigned
30	17	12	Bacteria	Proteobacteria	Alphaproteobacteria	Rhizobiales	Hyphomicrobiaceae	Devosia	Unassigned
418	27	4	Bacteria	Proteobacteria	Alphaproteobacteria	Rhizobiales	Phyllobacteriaceae	Unassigned	Unassigned
28	3	6	Bacteria	Proteobacteria	Gammaproteobacteria	Alteromonadales	Unassigned	Unassigned	Unassigned
34	11	1	Bacteria	Proteobacteria	Gammaproteobacteria	Chromatiales	Chromatiaceae	Halochromatium	Unassigned
275	8	2	Bacteria	Proteobacteria	Alphaproteobacteria	Rhodobacterales	Rhodobacteraceae	Unassigned	Unassigned
52	11	3	Bacteria	Proteobacteria	Betaproteobacteria	Methylophilales	Methylophilaceae	Methylotenera	mobilis
125	15	8	Bacteria	Proteobacteria	Betaproteobacteria	Burkholderiales	Comamonadaceae	Hydrogenophaga	Unassigned
53	5	5	Bacteria	Proteobacteria	Alphaproteobacteria	Rhizobiales	Hyphomicrobiaceae	Devosia	Unassigned
248	3	12	Bacteria	Proteobacteria	Alphaproteobacteria	Rhizobiales	Phyllobacteriaceae	Unassigned	Unassigned
160	7	9	Bacteria	Proteobacteria	Alphaproteobacteria	Kiloniellales	Kiloniellaceae	Unassigned	Unassigned
72	15	1	Bacteria	Proteobacteria	Alphaproteobacteria	Rhizobiales	Unassigned	Unassigned	Unassigned
183	2	1	Bacteria	Proteobacteria	Betaproteobacteria	Methylophilales	Methylophilaceae	Methylotenera	mobilis
825	12	5	Bacteria	Proteobacteria	Alphaproteobacteria	Rhodobacterales	Rhodobacteraceae	Phaeobacter	Unassigned

68	14	2	Bacteria	Proteobacteria	Alphaproteobacteria	Rhodobacterales	Rhodobacteraceae	Amaricoccus	Unassigned
898	1	5	Bacteria	Proteobacteria	Alphaproteobacteria	Sphingomonadales	Erythrobacteraceae	Lutibacterium	Unassigned
56	4	2	Bacteria	Proteobacteria	Deltaproteobacteria	Myxococcales	Unassigned	Unassigned	Unassigned
75	1	28	Bacteria	Firmicutes	Bacilli	Bacillales	Planococcaceae	Planococcus	Unassigned
541	11	3	Bacteria	Proteobacteria	Alphaproteobacteria	Rhodobacterales	Rhodobacteraceae	Phaeobacter	Unassigned
155	4	9	Bacteria	Actinobacteria	Acidimicrobiia	Acidimicrobiales	C111	Unassigned	Unassigned
300	4	12	Bacteria	Proteobacteria	Gammaproteobacteria	Alteromonadales	OM60	Congregibacter	Unassigned
1401	8	7	Bacteria	Proteobacteria	Alphaproteobacteria	Rhodobacterales	Rhodobacteraceae	Anaerospora	Unassigned
103	2	8	Bacteria	Proteobacteria	Alphaproteobacteria	Rhizobiales	Hyphomicrobiaceae	Unassigned	Unassigned
348	2	1	Bacteria	Proteobacteria	Alphaproteobacteria	Rhodobacterales	Rhodobacteraceae	Unassigned	Unassigned
85	6	2	Bacteria	Gemmatimonade	Gemm-2	Unassigned	Unassigned	Unassigned	Unassigned
154	1	13	Bacteria	Actinobacteria	Acidimicrobiia	Acidimicrobiales	Unassigned	Unassigned	Unassigned
1210	2	9	Bacteria	Proteobacteria	Gammaproteobacteria	Alteromonadales	OM60	Unassigned	Unassigned
1285	1	1	Bacteria	Proteobacteria	Alphaproteobacteria	Sphingomonadales	Erythrobacteraceae	Unassigned	Unassigned
176	5	1	Bacteria	Proteobacteria	Gammaproteobacteria	Chromatiales	Unassigned	Unassigned	Unassigned
212	1	10	Bacteria	Proteobacteria	Alphaproteobacteria	Rhodobacterales	Unassigned	Unassigned	Unassigned
222	2	1	Bacteria	Proteobacteria	Alphaproteobacteria	Sphingomonadales	Erythrobacteraceae	Erythrobacter	Unassigned
78	2	1	Bacteria	Proteobacteria	Alphaproteobacteria	Rhizobiales	Rhizobiaceae	Unassigned	Unassigned
258	2	2	Bacteria	Proteobacteria	Alphaproteobacteria	Sphingomonadales	Erythrobacteraceae	Unassigned	Unassigned
111	2	7	Bacteria	Proteobacteria	Alphaproteobacteria	Rhodobacterales	Rhodobacteraceae	Paracoccus	Unassigned
175	3	5	Bacteria	Actinobacteria	Acidimicrobiia	Acidimicrobiales	C111	Unassigned	Unassigned
681	3	5	Bacteria	Proteobacteria	Alphaproteobacteria	Rhizobiales	Hyphomicrobiaceae	Devosia	Unassigned
76	2	5	Bacteria	Proteobacteria	Gammaproteobacteria	Alteromonadales	HTCC2188	Unassigned	Unassigned
971	8	1	Bacteria	Proteobacteria	Alphaproteobacteria	Rhodobacterales	Rhodobacteraceae	Dinoroseobacter	Unassigned
70	6	1	Bacteria	Acidobacteria	Solibacteres	Solibacterales	PAUC26f	Unassigned	Unassigned
288	4	5	Bacteria	Proteobacteria	Alphaproteobacteria	Rhodobacterales	Rhodobacteraceae	Unassigned	Unassigned
129	10	1	Bacteria	Proteobacteria	Alphaproteobacteria	Rhodospirillales	Unassigned	Unassigned	Unassigned
102	8	2	Bacteria	Proteobacteria	Alphaproteobacteria	Rhizobiales	Unassigned	Unassigned	Unassigned
1122	1	7	Bacteria	Proteobacteria	Alphaproteobacteria	Rhodospirillales	Unassigned	Unassigned	Unassigned
268	1	7	Bacteria	Proteobacteria	Gammaproteobacteria	Thiotrichales	Piscirickettsiaceae	Unassigned	Unassigned
168	3	2	Bacteria	Bacteroidetes	[Saprospirae]	[Saprospirales]	Saprospiraceae	Lewinella	nigricans
187	6	1	Bacteria	WS6	B142	Unassigned	Unassigned	Unassigned	Unassigned
451	1	6	Bacteria	Actinobacteria	Acidimicrobiia	Acidimicrobiales	koll13	Unassigned	Unassigned

720	1	3	Bacteria	Proteobacteria	Alphaproteobacteria	Rhizobiales	Hyphomicrobiaceae	Devosia	Unassigned
1657	2	2	Bacteria	Proteobacteria	Alphaproteobacteria	Rhodobacterales	Rhodobacteraceae	Unassigned	Unassigned
211	2	1	Bacteria	Proteobacteria	Gammaproteobacteria	Oceanospirillales	Oceanospirillaceae	Unassigned	Unassigned
233	8	2	Bacteria	Chloroflexi	Anaerolineae	SBR1031	A4b	Unassigned	Unassigned
309	3	4	Bacteria	Proteobacteria	Alphaproteobacteria	Rhizobiales	Unassigned	Unassigned	Unassigned
399	2	2	Bacteria	Proteobacteria	Alphaproteobacteria	Rhizobiales	Hyphomicrobiaceae	Unassigned	Unassigned
335	2	3	Bacteria	Nitrospirae	Nitrospira	Nitrospirales	Nitrospiraceae	Unassigned	Unassigned
374	4	3	Bacteria	Proteobacteria	Alphaproteobacteria	Unassigned	Unassigned	Unassigned	Unassigned
240	2	1	Bacteria	Proteobacteria	Gammaproteobacteria	Thiotrichales	Piscirickettsiaceae	Unassigned	Unassigned
1110	1	2	Bacteria	Proteobacteria	Alphaproteobacteria	Rhodobacterales	Rhodobacteraceae	Unassigned	Unassigned
262	2	2	Bacteria	Proteobacteria	Alphaproteobacteria	Rhizobiales	Unassigned	Unassigned	Unassigned
269	1	1	Bacteria	Actinobacteria	Acidimicrobiia	Acidimicrobiales	ntu14	Unassigned	Unassigned
323	2	3	Bacteria	Proteobacteria	Alphaproteobacteria	BD7-3	Unassigned	Unassigned	Unassigned
738	4	4	Bacteria	Proteobacteria	Alphaproteobacteria	Rhodobacterales	Rhodobacteraceae	Amaricoccus	Unassigned
1154	1	1	Bacteria	Proteobacteria	Alphaproteobacteria	Rhodobacterales	Hyphomonadaceae	Hyphomonas	Unassigned
406	1	1	Bacteria	Proteobacteria	Gammaproteobacteria	Alteromonadales	Alteromonadaceae	ND137	Unassigned
1881	1	1	Bacteria	Proteobacteria	Alphaproteobacteria	Rhodobacterales	Rhodobacteraceae	Octadecabacter	Unassigned
290	4	1	Bacteria	Proteobacteria	Alphaproteobacteria	BD7-3	Unassigned	Unassigned	Unassigned
422	2	1	Bacteria	Proteobacteria	Alphaproteobacteria	Unassigned	Unassigned	Unassigned	Unassigned
577	1	2	Bacteria	Chloroflexi	Anaerolineae	SBR1031	A4b	Unassigned	Unassigned
642	1	1	Bacteria	GN02	BB34	Unassigned	Unassigned	Unassigned	Unassigned
524	1	1	Bacteria	Bacteroidetes	Cytophagia	Cytophagales	Cytophagaceae	Unassigned	Unassigned
676	1	2	Bacteria	Chloroflexi	Anaerolineae	SBR1031	A4b	Unassigned	Unassigned
801	1	1	Bacteria	Proteobacteria	Deltaproteobacteria	NB1-j	JTB38	Unassigned	Unassigned

Table S5. Common OTUs between grow-out environments (sediment vs. aquaponics) of *H. portulacoides* endosphere

OTU	Endosphere	Endosphere	Domain	Phylum	Class	Order	Family	Genus	Species
3	27	3	Bacteria	Proteobacteria	Epsilonproteobacteria	Campylobacterales	Campylobacteraceae	Arcobacter	Unassigned
14	111	4	Bacteria	Proteobacteria	Alphaproteobacteria	Rhodobacterales	Rhodobacteraceae	Loktanella	Unassigned
6	16	9	Bacteria	Proteobacteria	Betaproteobacteria	Rhodocyclales	Rhodocyclaceae	Unassigned	Unassigned
18	14	28	Bacteria	Proteobacteria	Alphaproteobacteria	Rhizobiales	Phyllobacteriaceae	Unassigned	Unassigned
1169	3	4	Bacteria	Proteobacteria	Alphaproteobacteria	Rhodobacterales	Rhodobacteraceae	Unassigned	Unassigned
32	5	1	Bacteria	Proteobacteria	Alphaproteobacteria	Rhodobacterales	Rhodobacteraceae	Octadecabacter	Unassigned
17	7	4	Bacteria	Proteobacteria	Alphaproteobacteria	Rhodobacterales	Rhodobacteraceae	Marivita	Unassigned
92	1	11	Bacteria	Proteobacteria	Alphaproteobacteria	Rhodobacterales	Rhodobacteraceae	Phaeobacter	Unassigned
29	17	13	Bacteria	Proteobacteria	Alphaproteobacteria	Rhizobiales	Phyllobacteriaceae	Unassigned	Unassigned
25	3	59	Bacteria	Proteobacteria	Deltaproteobacteria	Myxococcales	Unassigned	Unassigned	Unassigned
61	11	27	Bacteria	Proteobacteria	Alphaproteobacteria	Rhizobiales	Hyphomicrobiaceae	Devosia	Unassigned
73	1	7	Bacteria	Proteobacteria	Alphaproteobacteria	Rhodobacterales	Rhodobacteraceae	Jannaschia	Unassigned
33	3	3	Bacteria	Proteobacteria	Alphaproteobacteria	Rhizobiales	Hyphomicrobiaceae	Unassigned	Unassigned
31	2	26	Bacteria	Proteobacteria	Alphaproteobacteria	Sphingomonadales	Erythrobacteraceae	Unassigned	Unassigned
202	6	5	Bacteria	Proteobacteria	Alphaproteobacteria	Rhodobacterales	Rhodobacteraceae	Unassigned	Unassigned
30	22	11	Bacteria	Proteobacteria	Alphaproteobacteria	Rhizobiales	Hyphomicrobiaceae	Devosia	Unassigned
418	11	7	Bacteria	Proteobacteria	Alphaproteobacteria	Rhizobiales	Phyllobacteriaceae	Unassigned	Unassigned
28	8	11	Bacteria	Proteobacteria	Gammaproteobacteria	Alteromonadales	Unassigned	Unassigned	Unassigned
54	6	3	Bacteria	Proteobacteria	Alphaproteobacteria	Rhodobacterales	Rhodobacteraceae	Phaeobacter	Unassigned
52	3	13	Bacteria	Proteobacteria	Betaproteobacteria	Methylophilales	Methylophilaceae	Methylotenera	mobilis
125	8	18	Bacteria	Proteobacteria	Betaproteobacteria	Burkholderiales	Comamonadaceae	Hydrogenophaga	Unassigned
46	1	7	Bacteria	Proteobacteria	Alphaproteobacteria	Kiloniellales	Kiloniellaceae	Thalassospira	Unassigned
53	1	11	Bacteria	Proteobacteria	Alphaproteobacteria	Rhizobiales	Hyphomicrobiaceae	Devosia	Unassigned
248	4	7	Bacteria	Proteobacteria	Alphaproteobacteria	Rhizobiales	Phyllobacteriaceae	Unassigned	Unassigned
72	3	1	Bacteria	Proteobacteria	Alphaproteobacteria	Rhizobiales	Unassigned	Unassigned	Unassigned
183	3	1	Bacteria	Proteobacteria	Betaproteobacteria	Methylophilales	Methylophilaceae	Methylotenera	mobilis
825	1	2	Bacteria	Proteobacteria	Alphaproteobacteria	Rhodobacterales	Rhodobacteraceae	Phaeobacter	Unassigned
49	1	1	Bacteria	Proteobacteria	Gammaproteobacteria	[Marinicellales]	[Marinicellaceae]	Marinicella	Unassigned
56	4	1	Bacteria	Proteobacteria	Deltaproteobacteria	Myxococcales	Unassigned	Unassigned	Unassigned
1401	2	3	Bacteria	Proteobacteria	Alphaproteobacteria	Rhodobacterales	Rhodobacteraceae	Anaerospora	Unassigned
103	1	3	Bacteria	Proteobacteria	Alphaproteobacteria	Rhizobiales	Hyphomicrobiaceae	Unassigned	Unassigned

136	1	4	Bacteria	Proteobacteria	Gammaproteobacteria	Chromatiales	Unassigned	Unassigned	Unassigned
176	6	1	Bacteria	Proteobacteria	Gammaproteobacteria	Chromatiales	Unassigned	Unassigned	Unassigned
76	2	17	Bacteria	Proteobacteria	Gammaproteobacteria	Alteromonadales	HTCC2188	Unassigned	Unassigned
100	1	1	Bacteria	Proteobacteria	Deltaproteobacteria	Desulfobacterales	Desulfobulbaceae	Unassigned	Unassigned
1657	1	1	Bacteria	Proteobacteria	Alphaproteobacteria	Rhodobacterales	Rhodobacteraceae	Unassigned	Unassigned
211	3	5	Bacteria	Proteobacteria	Gammaproteobacteria	Oceanospirillales	Oceanospirillaceae	Unassigned	Unassigned
245	1	2	Bacteria	Bacteroidetes	Flavobacteriia	Flavobacteriales	Flavobacteriaceae	Maribacter	Unassigned
283	1	1	Bacteria	Proteobacteria	Alphaproteobacteria	Rhizobiales	Hyphomicrobiaceae	Unassigned	Unassigned
396	1	1	Bacteria	Proteobacteria	Gammaproteobacteria	Oceanospirillales	Saccharospirillaceae	Saccharospirillum	Unassigned
241	1	1	Bacteria	Proteobacteria	Alphaproteobacteria	Rhizobiales	Unassigned	Unassigned	Unassigned
409	1	1	Bacteria	Proteobacteria	Betaproteobacteria	Rhodocyclales	Rhodocyclaceae	Azoarcus	Unassigned
1166	1	1	Bacteria	Bacteroidetes	[Saprospirae]	[Saprospirales]	Chitinophagaceae	Unassigned	Unassigned
521	1	1	Bacteria	Proteobacteria	Gammaproteobacteria	[Marinicellales]	[Marinicellaceae]	Marinicella	Unassigned
1872	1	1	Bacteria	Proteobacteria	Gammaproteobacteria	Alteromonadales	OM60	Unassigned	Unassigned

Table S6. Common OTUs between grow-out environments (sediment vs. aquaponics) of *Sal.ramossisima* rhizosphere.

OTU	Aquaponic	Sediment	Domain	Phylum	Class	Order	Family	Genus	Species
14	22	3	Bacteria	Proteobacteria	Alphaproteobacteria	Rhodobacterales	Rhodobacteraceae	Loktanella	Unassigned
6	82	2	Bacteria	Proteobacteria	Betaproteobacteria	Rhodocyclales	Rhodocyclaceae	Unassigned	Unassigned
18	31	7	Bacteria	Proteobacteria	Alphaproteobacteria	Rhizobiales	Phyllobacteriaceae	Unassigned	Unassigned
17	34	3	Bacteria	Proteobacteria	Alphaproteobacteria	Rhodobacterales	Rhodobacteraceae	Marivita	Unassigned
1507	8	4	Bacteria	Proteobacteria	Alphaproteobacteria	Sphingomonadales	Erythrobacteraceae	Unassigned	Unassigned
92	38	3	Bacteria	Proteobacteria	Alphaproteobacteria	Rhodobacterales	Rhodobacteraceae	Phaeobacter	Unassigned
80	6	2	Bacteria	Proteobacteria	Alphaproteobacteria	Sphingomonadales	Unassigned	Unassigned	Unassigned
151	12	6	Bacteria	Proteobacteria	Alphaproteobacteria	Rhodobacterales	Rhodobacteraceae	Unassigned	Unassigned
33	2	10	Bacteria	Proteobacteria	Alphaproteobacteria	Rhizobiales	Hyphomicrobiaceae	Unassigned	Unassigned
202	5	1	Bacteria	Proteobacteria	Alphaproteobacteria	Rhodobacterales	Rhodobacteraceae	Unassigned	Unassigned
34	4	1	Bacteria	Proteobacteria	Gammaproteobacteria	Chromatiales	Chromatiaceae	Halochromatium	Unassigned
275	19	1	Bacteria	Proteobacteria	Alphaproteobacteria	Rhodobacterales	Rhodobacteraceae	Unassigned	Unassigned
160	15	1	Bacteria	Proteobacteria	Alphaproteobacteria	Kiloniellales	Kiloniellaceae	Unassigned	Unassigned
183	6	9	Bacteria	Proteobacteria	Betaproteobacteria	Methylophilales	Methylophilaceae	Methylotenera	mobilis
49	7	3	Bacteria	Proteobacteria	Gammaproteobacteria	[Marinicellales]	[Marinicellaceae]	Marinicella	Unassigned
68	13	4	Bacteria	Proteobacteria	Alphaproteobacteria	Rhodobacterales	Rhodobacteraceae	Amaricoccus	Unassigned
898	1	1	Bacteria	Proteobacteria	Alphaproteobacteria	Sphingomonadales	Erythrobacteraceae	Lutibacterium	Unassigned
44	4	6	Bacteria	Proteobacteria	Gammaproteobacteria	Alteromonadales	Unassigned	Unassigned	Unassigned
1401	2	1	Bacteria	Proteobacteria	Alphaproteobacteria	Rhodobacterales	Rhodobacteraceae	Anaerospora	Unassigned
103	2	2	Bacteria	Proteobacteria	Alphaproteobacteria	Rhizobiales	Hyphomicrobiaceae	Unassigned	Unassigned
348	5	6	Bacteria	Proteobacteria	Alphaproteobacteria	Rhodobacterales	Rhodobacteraceae	Unassigned	Unassigned
1210	1	1	Bacteria	Proteobacteria	Gammaproteobacteria	Alteromonadales	OM60	Unassigned	Unassigned
222	12	2	Bacteria	Proteobacteria	Alphaproteobacteria	Sphingomonadales	Erythrobacteraceae	Erythrobacter	Unassigned
78	8	1	Bacteria	Proteobacteria	Alphaproteobacteria	Rhizobiales	Rhizobiaceae	Unassigned	Unassigned
258	1	1	Bacteria	Proteobacteria	Alphaproteobacteria	Sphingomonadales	Erythrobacteraceae	Unassigned	Unassigned
111	8	1	Bacteria	Proteobacteria	Alphaproteobacteria	Rhodobacterales	Rhodobacteraceae	Paracoccus	Unassigned
115	6	1	Bacteria	Proteobacteria	Alphaproteobacteria	Rhizobiales	Hyphomicrobiaceae	Hyphomicrobium	Unassigned
681	8	1	Bacteria	Proteobacteria	Alphaproteobacteria	Rhizobiales	Hyphomicrobiaceae	Devosia	Unassigned
971	1	1	Bacteria	Proteobacteria	Alphaproteobacteria	Rhodobacterales	Rhodobacteraceae	Dinoroseobacter	Unassigned
97	10	2	Bacteria	Gemmatimonadetes	Gemmatimonadetes	Gemmatimonadales	Gemmatimonadaceae	Gemmatimonas	Unassigned
169	2	7	Bacteria	Proteobacteria	Gammaproteobacteria	Oceanospirillales	Oceanospirillaceae	Marinomonas	Unassigned

1657	1	1	Bacteria	Proteobacteria	Alphaproteobacteria	Rhodobacterales	Rhodobacteraceae	Unassigned	Unassigned
116	4	1	Bacteria	Proteobacteria	Gammaproteobacteria	HOC36	Unassigned	Unassigned	Unassigned
177	1	3	Bacteria	Cyanobacteria	Synechococcophycideae	Synechococcales	Synechococcaceae	Synechococcus	Unassigned
987	1	1	Bacteria	Actinobacteria	Acidimicrobiia	Acidimicrobiales	C111	Unassigned	Unassigned
346	1	4	Bacteria	Bacteroidetes	[Saprospirae]	[Saprospirales]	Chitinophagaceae	Unassigned	Unassigned
356	1	1	Bacteria	Proteobacteria	Alphaproteobacteria	Rhodobacterales	Hyphomonadaceae	Unassigned	Unassigned
1709	1	3	Bacteria	Proteobacteria	Alphaproteobacteria	Rhodobacterales	Rhodobacteraceae	Phaeobacter	Unassigned
345	3	1	Bacteria	Proteobacteria	Gammaproteobacteria	HTCC2188	HTCC2089	Unassigned	Unassigned
361	1	1	Bacteria	Proteobacteria	Gammaproteobacteria	Vibrionales	Vibrionaceae	Vibrio	Unassigned
536	1	3	Bacteria	Proteobacteria	Alphaproteobacteria	Sphingomonadales	Sphingomonadaceae	Kaistobacter	Unassigned
586	1	1	Bacteria	Proteobacteria	Alphaproteobacteria	BD7-3	Unassigned	Unassigned	Unassigned
1803	1	2	Bacteria	Proteobacteria	Epsilonproteobacteria	Campylobacterales	Helicobacteraceae	Unassigned	Unassigned
652	1	1	Bacteria	Proteobacteria	Deltaproteobacteria	Syntrophobacterales	Syntrophobacteraceae	Unassigned	Unassigned

Table S7. Common OTUs between grow-out environments (sediment vs. aquaponics) of *Sal.ramossisima* endosphere.

OTU	Aquaponic	Sediment	Domain	Phylum	Class	Order	Family	Genus	Species
6	18	3	Bacteria	Proteobacteria	Betaproteobacteria	Rhodocyclales	Rhodocyclaceae	Unassigned	Unassigned
18	32	24	Bacteria	Proteobacteria	Alphaproteobacteria	Rhizobiales	Phyllobacteriaceae	Unassigned	Unassigned
17	27	1	Bacteria	Proteobacteria	Alphaproteobacteria	Rhodobacterales	Rhodobacteraceae	Marivita	Unassigned
1507	3	10	Bacteria	Proteobacteria	Alphaproteobacteria	Sphingomonadales	Erythrobacteraceae	Unassigned	Unassigned
92	25	1	Bacteria	Proteobacteria	Alphaproteobacteria	Rhodobacterales	Rhodobacteraceae	Phaeobacter	Unassigned
15	111	1	Bacteria	Proteobacteria	Alphaproteobacteria	Rhizobiales	Hyphomicrobiaceae	Hyphomicrobium	Unassigned
80	5	1	Bacteria	Proteobacteria	Alphaproteobacteria	Sphingomonadales	Unassigned	Unassigned	Unassigned
151	2	2	Bacteria	Proteobacteria	Alphaproteobacteria	Rhodobacterales	Rhodobacteraceae	Unassigned	Unassigned
74	1	3	Bacteria	Proteobacteria	Alphaproteobacteria	Rhizobiales	Phyllobacteriaceae	Mesorhizobium	Unassigned
33	7	16	Bacteria	Proteobacteria	Alphaproteobacteria	Rhizobiales	Hyphomicrobiaceae	Unassigned	Unassigned
202	3	1	Bacteria	Proteobacteria	Alphaproteobacteria	Rhodobacterales	Rhodobacteraceae	Unassigned	Unassigned
34	2	1	Bacteria	Proteobacteria	Gammaproteobacteria	Chromatiales	Chromatiaceae	Halochromatium	Unassigned
125	3	1	Bacteria	Proteobacteria	Betaproteobacteria	Burkholderiales	Comamonadaceae	Hydrogenophaga	Unassigned
46	2	13	Bacteria	Proteobacteria	Alphaproteobacteria	Kiloniellales	Kiloniellaceae	Thalassospira	Unassigned
248	2	6	Bacteria	Proteobacteria	Alphaproteobacteria	Rhizobiales	Phyllobacteriaceae	Unassigned	Unassigned
183	9	10	Bacteria	Proteobacteria	Betaproteobacteria	Methylophilales	Methylophilaceae	Methylotenera	mobilis
49	19	7	Bacteria	Proteobacteria	Gammaproteobacteria	[Marinicellales]	[Marinicellaceae]	Marinicella	Unassigned
898	1	3	Bacteria	Proteobacteria	Alphaproteobacteria	Sphingomonadales	Erythrobacteraceae	Lutibacterium	Unassigned
44	1	26	Bacteria	Proteobacteria	Gammaproteobacteria	Alteromonadales	Unassigned	Unassigned	Unassigned
103	4	3	Bacteria	Proteobacteria	Alphaproteobacteria	Rhizobiales	Hyphomicrobiaceae	Unassigned	Unassigned
348	3	3	Bacteria	Proteobacteria	Alphaproteobacteria	Rhodobacterales	Rhodobacteraceae	Unassigned	Unassigned
115	5	3	Bacteria	Proteobacteria	Alphaproteobacteria	Rhizobiales	Hyphomicrobiaceae	Hyphomicrobium	Unassigned
681	6	1	Bacteria	Proteobacteria	Alphaproteobacteria	Rhizobiales	Hyphomicrobiaceae	Devosia	Unassigned
169	2	2	Bacteria	Proteobacteria	Gammaproteobacteria	Oceanospirillales	Oceanospirillaceae	Marinomonas	Unassigned
987	2	1	Bacteria	Actinobacteria	Acidimicrobiia	Acidimicrobiales	C111	Unassigned	Unassigned
351	2	1	Bacteria	Proteobacteria	Alphaproteobacteria	Sphingomonadales	Erythrobacteraceae	Altererythrobacter	indicus
194	1	1	Bacteria	Bacteroidetes	[Rhodothermi]	[Rhodothermales]	Rhodothermaceae	Rubricoccus	Unassigned
356	3	1	Bacteria	Proteobacteria	Alphaproteobacteria	Rhodobacterales	Hyphomonadaceae	Unassigned	Unassigned
396	2	1	Bacteria	Proteobacteria	Gammaproteobacteria	Oceanospirillales	Saccharospirillaceae	Saccharospirillum	Unassigned
406	1	1	Bacteria	Proteobacteria	Gammaproteobacteria	Alteromonadales	Alteromonadaceae	ND137	Unassigned
623	1	1	Bacteria	Proteobacteria	Gammaproteobacteria	Pseudomonadales	Pseudomonadaceae	Pseudomonas	pseudoalcaligenes

Table S8. Common OTUs between grow-out environments (sediment vs. aquaponics) of *Sar.perennis* rhizosphere.

OTU	Aquaponic	Sediment	Domain	Phylum	Class	Order	Family	Genus	Species
14	1	4	Bacteria	Proteobacteria	Alphaproteobacteria	Rhodobacterales	Rhodobacteraceae	Loktanella	Unassigned
6	38	1	Bacteria	Proteobacteria	Betaproteobacteria	Rhodocyclales	Rhodocyclaceae	Unassigned	Unassigned
18	4	10	Bacteria	Proteobacteria	Alphaproteobacteria	Rhizobiales	Phyllobacteriaceae	Unassigned	Unassigned
32	9	3	Bacteria	Proteobacteria	Alphaproteobacteria	Rhodobacterales	Rhodobacteraceae	Octadecabacter	Unassigned
17	4	1	Bacteria	Proteobacteria	Alphaproteobacteria	Rhodobacterales	Rhodobacteraceae	Marivita	Unassigned
1507	1	47	Bacteria	Proteobacteria	Alphaproteobacteria	Sphingomonadales	Erythrobacteraceae	Unassigned	Unassigned
26	2	1	Bacteria	Proteobacteria	Epsilonproteobacteria	Campylobacterales	Campylobacteraceae	Arcobacter	Unassigned
92	4	3	Bacteria	Proteobacteria	Alphaproteobacteria	Rhodobacterales	Rhodobacteraceae	Phaeobacter	Unassigned
61	3	4	Bacteria	Proteobacteria	Alphaproteobacteria	Rhizobiales	Hyphomicrobiaceae	Devosia	Unassigned
73	5	16	Bacteria	Proteobacteria	Alphaproteobacteria	Rhodobacterales	Rhodobacteraceae	Jannaschia	Unassigned
93	3	2	Bacteria	Proteobacteria	Gammaproteobacteria	Chromatiales	Unassigned	Unassigned	Unassigned
202	1	18	Bacteria	Proteobacteria	Alphaproteobacteria	Rhodobacterales	Rhodobacteraceae	Unassigned	Unassigned
418	1	3	Bacteria	Proteobacteria	Alphaproteobacteria	Rhizobiales	Phyllobacteriaceae	Unassigned	Unassigned
54	2	1	Bacteria	Proteobacteria	Alphaproteobacteria	Rhodobacterales	Rhodobacteraceae	Phaeobacter	Unassigned
275	1	10	Bacteria	Proteobacteria	Alphaproteobacteria	Rhodobacterales	Rhodobacteraceae	Unassigned	Unassigned
52	1	1	Bacteria	Proteobacteria	Betaproteobacteria	Methylophilales	Methylophilaceae	Methylotenera	mobilis
56	3	1	Bacteria	Proteobacteria	Deltaproteobacteria	Myxococcales	Unassigned	Unassigned	Unassigned
1401	2	2	Bacteria	Proteobacteria	Alphaproteobacteria	Rhodobacterales	Rhodobacteraceae	Anaerospora	Unassigned
348	1	1	Bacteria	Proteobacteria	Alphaproteobacteria	Rhodobacterales	Rhodobacteraceae	Unassigned	Unassigned
176	1	1	Bacteria	Proteobacteria	Gammaproteobacteria	Chromatiales	Unassigned	Unassigned	Unassigned
100	2	2	Bacteria	Proteobacteria	Deltaproteobacteria	Desulfobacterales	Desulfobulbaceae	Unassigned	Unassigned
164	1	1	Bacteria	Proteobacteria	Alphaproteobacteria	Kiloniellales	Unassigned	Unassigned	Unassigned
1881	1	1	Bacteria	Proteobacteria	Alphaproteobacteria	Rhodobacterales	Rhodobacteraceae	Octadecabacter	Unassigned

Table S9. Common OTUs between grow-out environments (sediment vs. aquaponics) of *Sar.perennis* endosphere.

OTU	Aquaponic	Sediment	Domain	Phylum	Class	Order	Family	Genus	Species
18	1	28	Bacteria	Proteobacteria	Alphaproteobacteria	Rhizobiales	Phyllobacteriaceae	Unassigned	Unassigned
29	6	1	Bacteria	Proteobacteria	Alphaproteobacteria	Rhizobiales	Phyllobacteriaceae	Unassigned	Unassigned
151	1	6	Bacteria	Proteobacteria	Alphaproteobacteria	Rhodobacterales	Rhodobacteraceae	Unassigned	Unassigned
74	1	44	Bacteria	Proteobacteria	Alphaproteobacteria	Rhizobiales	Phyllobacteriaceae	Mesorhizobium	Unassigned
61	7	8	Bacteria	Proteobacteria	Alphaproteobacteria	Rhizobiales	Hyphomicrobiaceae	Devosia	Unassigned
73	8	11	Bacteria	Proteobacteria	Alphaproteobacteria	Rhodobacterales	Rhodobacteraceae	Jannaschia	Unassigned
30	2	7	Bacteria	Proteobacteria	Alphaproteobacteria	Rhizobiales	Hyphomicrobiaceae	Devosia	Unassigned
34	1	1	Bacteria	Proteobacteria	Gammaproteobacteria	Chromatiales	Chromatiaceae	Halochromatium	Unassigned
52	1	2	Bacteria	Proteobacteria	Betaproteobacteria	Methylophilales	Methylophilaceae	Methylotenera	mobilis
46	1	21	Bacteria	Proteobacteria	Alphaproteobacteria	Kiloniellales	Kiloniellaceae	Thalassospira	Unassigned
248	1	4	Bacteria	Proteobacteria	Alphaproteobacteria	Rhizobiales	Phyllobacteriaceae	Unassigned	Unassigned
541	2	7	Bacteria	Proteobacteria	Alphaproteobacteria	Rhodobacterales	Rhodobacteraceae	Phaeobacter	Unassigned
176	3	2	Bacteria	Proteobacteria	Gammaproteobacteria	Chromatiales	Unassigned	Unassigned	Unassigned
971	2	3	Bacteria	Proteobacteria	Alphaproteobacteria	Rhodobacterales	Rhodobacteraceae	Dinoroseobacter	Unassigned
130	2	1	Bacteria	Proteobacteria	Alphaproteobacteria	Rhizobiales	Hyphomicrobiaceae	Unassigned	Unassigned
90	6	1	Bacteria	Proteobacteria	Deltaproteobacteria	Myxococcales	Unassigned	Unassigned	Unassigned
144	1	2	Bacteria	Proteobacteria	Deltaproteobacteria	Unassigned	Unassigned	Unassigned	Unassigned
885	2	4	Bacteria	Proteobacteria	Gammaproteobacteria	Unassigned	Unassigned	Unassigned	Unassigned
1382	1	3	Bacteria	Proteobacteria	Gammaproteobacteria	Thiotrichales	Piscirickettsiaceae	Unassigned	Unassigned
329	1	5	Bacteria	Proteobacteria	Deltaproteobacteria	Myxococcales	Unassigned	Unassigned	Unassigned
1239	1	1	Bacteria	Proteobacteria	Gammaproteobacteria	Alteromonadales	Unassigned	Unassigned	Unassigned

Table S10. Taxonomic affiliation of the most abundant OTUs (≥ 50 sequences) including OTU-numbers (OTU); number of sequence reads (Sum); taxonomic assignment, GenBank identifiers (GI) of closely related organisms identified using BLAST; sequence identity (Sq I) of these organisms with our representative OTU sequences and their source.

OTU	Sum	Phylum	Class	Order	Family	Genus	GI	Sq I	Source
23	68	Bacteroidetes	Saprospirae	Saprospirales	Unassigned	Unassigned	295147124	95	marine biofouling
42	87	Bacteroidetes	Saprospirae	Saprospirales	Saprospiraceae	Lewinella	530549991	98	seawater
79	59	Bacteroidetes	Saprospirae	Saprospirales	Saprospiraceae	Lewinella	530549991	93	seawater
38	50	Cyanobacteria	Synechococcophycideae	Synechococcales	Acaryochloridaceae	Acaryochloris	45476310	100	red algae epiphytic cyanobacterium
3	179	Proteobacteria	Epsilonproteobacteria	Campylobacterales	Campylobacteraceae	Arcobacter	557818018	99	intertidal sediment
4	227	Proteobacteria	Gammaproteobacteria	Oceanospirillales	Oceanospirillaceae	Unassigned	379334633	98	Figueiras beach sediments
6	283	Proteobacteria	Betaproteobacteria	Rhodocyclales	Rhodocyclaceae	Unassigned	115334100	100	marine aquaculture
7	195	Proteobacteria	Epsilonproteobacteria	Campylobacterales	Campylobacteraceae	Arcobacter	557818018	97	intertidal sediment
10	207	Proteobacteria	Epsilonproteobacteria	Campylobacterales	Campylobacteraceae	Arcobacter	397172659	97	saltern
11	272	Proteobacteria	Epsilonproteobacteria	Campylobacterales	Campylobacteraceae	Arcobacter	7671475	99	Black Sea shelf sediments
12	174	Proteobacteria	Gammaproteobacteria	Alteromonadales	Colwelliaceae	Unassigned	323126147	100	carbon source enrichment experiment
13	161	Proteobacteria	Alphaproteobacteria	Rhodobacterales	Rhodobacteraceae	Unassigned	756558269	97	aquacultured sea cucumber intestines
14	271	Proteobacteria	Alphaproteobacteria	Rhodobacterales	Rhodobacteraceae	Loktanella	698314707	100	surface seawater
15	143	Proteobacteria	Alphaproteobacteria	Rhizobiales	Hyphomicrobiaceae	Hyphomicrobium	41058911	98	soil and sediment sources
17	103	Proteobacteria	Alphaproteobacteria	Rhodobacterales	Rhodobacteraceae	Marivita	704001720	100	*Coccolithus braarudii* clonal culture
18	219	Proteobacteria	Alphaproteobacteria	Rhizobiales	Phyllobacteriaceae	Unassigned	704001721	100	*Coccolithus braarudii* clonal culture

19	136	Proteobacteria	Gammaproteobacteria	Chromatiales	Unassigned	Unassigned	683427046	99	Sippewissett salt marsh
22	122	Proteobacteria	Gammaproteobacteria	Xanthomonadales	Xanthomonadaceae	Dokdonella	566084877	99	triphenylmethane dye treatment bioreactor
24	90	Proteobacteria	Gammaproteobacteria	Chromatiales	Unassigned	Unassigned	219693545	96	water
25	107	Proteobacteria	Deltaproteobacteria	Myxococcales	Unassigned	Unassigned	699005313	92	soil
26	53	Proteobacteria	Epsilonproteobacteria	Campylobacterales	Campylobacteraceae	Arcobacter	507148066	98	*Spartina alterniflora* roots
27	93	Proteobacteria	Deltaproteobacteria	Myxococcales	Unassigned	Unassigned	339646456	92	wetland soil
28	69	Proteobacteria	Gammaproteobacteria	Alteromonadales	Unassigned	Unassigned	37528791	95	marine
29	115	Proteobacteria	Alphaproteobacteria	Rhizobiales	Phyllobacteriaceae	Unassigned	300791752	100	marine inoculum
30	74	Proteobacteria	Alphaproteobacteria	Rhizobiales	Hyphomicrobiaceae	Devosia	594541071	96	rhizoshere
31	75	Proteobacteria	Alphaproteobacteria	Sphingomonadales	Erythrobacteraceae	Unassigned	641803625	98	rhizoshere
32	142	Proteobacteria	Alphaproteobacteria	Rhodobacterales	Rhodobacteraceae	Octadecabacter	636560661	98	seawater
33	72	Proteobacteria	Alphaproteobacteria	Rhizobiales	Hyphomicrobiaceae	Unassigned	817049210	100	algea
36	105	Proteobacteria	Deltaproteobacteria	Desulfobacterales	Desulfobulbaceae	Unassigned	34761448	99	salt marsh sediment
41	66	Proteobacteria	Gammaproteobacteria	Chromatiales	Unassigned	Unassigned	683427046	97	Sippewissett salt marsh
46	52	Proteobacteria	Alphaproteobacteria	Kiloniellales	Kiloniellaceae	Thalassospira	730230191	99	surface sediment
52	55	Proteobacteria	Betaproteobacteria	Methylophilales	Methylophilaceae	Methylotenera	380850770	99	surface of antifouling paint
53	52	Proteobacteria	Alphaproteobacteria	Rhizobiales	Hyphomicrobiaceae	Devosia	373939505	100	estuarine water
58	71	Proteobacteria	Gammaproteobacteria	Unassigned	Unassigned	Unassigned	326699156	96	oil-polluted subtidal sediments
61	92	Proteobacteria	Alphaproteobacteria	Rhizobiales	Hyphomicrobiaceae	Devosia	72509674	97	*Galatella meyendorffii* root
64	99	Proteobacteria	Alphaproteobacteria	Rhodobacterales	Rhodobacteraceae	Unassigned	216409142	99	seaweed
73	78	Proteobacteria	Alphaproteobacteria	Rhodobacterales	Rhodobacteraceae	Jannaschia	470467218	99	coastal seawater
74	94	Proteobacteria	Alphaproteobacteria	Rhizobiales	Phyllobacteriaceae	Mesorhizobium	829496234	99	root nodule
80	114	Proteobacteria	Alphaproteobacteria	Sphingomonadales	Unassigned	Unassigned	429844878	100	red algea
92	127	Proteobacteria	Alphaproteobacteria	Rhodobacterales	Rhodobacteraceae	Phaeobacter	306518462	99	marine seawater

93	81	Proteobacteria	Gammaproteobacteria	Chromatiales	Unassigned	Unassigned	209869668	99	sediment from oil polluted water
125	55	Proteobacteria	Betaproteobacteria	Burkholderiales	Comamonadaceae	Hydrogenophaga	485090967	99	*Leontopodium alpinum rhizoplane*
151	86	Proteobacteria	Alphaproteobacteria	Rhodobacterales	Rhodobacteraceae	Unassigned	169788231	99	Lagoon sediment
202	60	Proteobacteria	Alphaproteobacteria	Rhodobacterales	Rhodobacteraceae	Unassigned	683427046	100	*Fucus spirales* macroalgae
248	51	Proteobacteria	Alphaproteobacteria	Rhizobiales	Phyllobacteriaceae	Unassigned	452108580	100	crude oil contaminated seawater
358	100	Proteobacteria	Alphaproteobacteria	Rhodobacterales	Rhodobacteraceae	Unassigned	698314710	100	surface seawater
418	69	Proteobacteria	Alphaproteobacteria	Rhizobiales	Phyllobacteriaceae	Unassigned	831210888	99	*Spartina alterniflora*
782	112	Proteobacteria	Epsilonproteobacteria	Campylobacterales	Campylobacteraceae	Arcobacter	767765908	99	marine sediment
1169	53	Proteobacteria	Alphaproteobacteria	Rhodobacterales	Rhodobacteraceae	Unassigned	544582403	100	seawater
1507	164	Proteobacteria	Alphaproteobacteria	Sphingomonadales	Erythrobacteraceae	Unassigned	346720604	100	petroleum contaminated soils

Table S11. Abundant OTUs (≥ 50 sequences) exclusive to aquaponics (Rhiz: Rhizosphere; End: Endosphere).

	H. portulacoides				***Sal.ramossisima***				***Sar. perennis***								
	**Aquaponic**		**Sediment**		**Aquaponic**		**Sediment**		**Aquaponic**		**Sediment**						
**OTU**	**Rhiz**	**End**	**Rhiz**	**End**	**Rhiz**	**End**	**Rhiz**	**End**	**Rhiz**	**End**	**Rhiz**	**End**	**Phylum**	**Class**	**Order**	**Family**	**Genus**
4	35	51	0	0	1	0	0	0	25	115	0	0	Proteobacteria	Gammaproteobacteria	Oceanospirillales	Oceanospirillaceae	Unassigned
7	44	37	0	0	15	1	0	0	33	65	0	0	Proteobacteria	Epsilonproteobacteria	Campylobacterales	Campylobacteraceae	Arcobacter
10	9	31	0	0	6	6	0	0	41	114	0	0	Proteobacteria	Epsilonproteobacteria	Campylobacterales	Campylobacteraceae	Arcobacter
11	155	98	0	0	7	1	0	0	9	2	0	0	Proteobacteria	Epsilonproteobacteria	Campylobacterales	Campylobacteraceae	Arcobacter
12	11	21	0	0	0	0	0	0	20	122	0	0	Proteobacteria	Gammaproteobacteria	Alteromonadales	Colwelliaceae	Unassigned
13	38	9	0	0	53	18	0	0	23	20	0	0	Proteobacteria	Alphaproteobacteria	Rhodobacterales	Rhodobacteraceae	Unassigned
19	10	37	0	0	10	9	0	0	13	57	0	0	Proteobacteria	Gammaproteobacteria	Chromatiales	Unassigned	Unassigned
23	14	0	0	0	45	4	0	0	4	1	0	0	Bacteroidetes	[Saprospirae]	[Saprospirales]	Unassigned	Unassigned
36	0	3	0	0	5	0	0	0	19	78	0	0	Proteobacteria	Deltaproteobacteria	Desulfobacterales	Desulfobulbaceae	Unassigned
38	1	0	0	0	15	34	0	0	0	0	0	0	Cyanobacteria	Synechococcophycideae	Synechococcales	Acaryochloridaceae	Acaryochloris
41	0	15	0	0	17	1	0	0	7	26	0	0	Proteobacteria	Gammaproteobacteria	Chromatiales	Unassigned	Unassigned
64	39	3	0	0	30	22	0	0	4	1	0	0	Proteobacteria	Alphaproteobacteria	Rhodobacterales	Rhodobacteraceae	Unassigned
358	2	0	0	0	76	19	0	0	1	2	0	0	Proteobacteria	Alphaproteobacteria	Rhodobacterales	Rhodobacteraceae	Unassigned
782	31	38	0	0	17	4	0	0	8	14	0	0	Proteobacteria	Epsilonproteobacteria	Campylobacterales	Campylobacteraceae	Arcobacter

Table S12. Abundant OTUs (≥ 50 sequences) exclusive to sediment (Rhiz: Rhizosphere; End: Endosphere).

	H. portulacoides				*Sal.ramossisima*				*Sar. perennis*								
	**Aquaponic**		**Sediment**		**Aquaponic**		**Sediment**		**Aquaponic**		**Sediment**						
**OTU**	**Rhiz**	**End**	**Rhiz**	**End**	**Rhiz**	**End**	**Rhiz**	**End**	**Rhiz**	**End**	**Rhiz**	**End**	**Phylum**	**Class**	**Order**	**Family**	**Genus**
22	0	0	50	62	0	0	0	1	0	0	8	1	Proteobacteria	Gammaproteobacteria	Xanthomonadales	Xanthomonadaceae	Dokdonella
27	0	0	7	85	0	0	0	0	0	0	1	0	Proteobacteria	Deltaproteobacteria	Myxococcales	Unassigned	Unassigned
42	0	0	7	13	0	0	0	0	0	0	22	45	Bacteroidetes	[Saprospirae]	[Saprospirales]	Saprospiraceae	Lewinella
58	0	0	11	4	0	0	0	0	0	0	27	29	Proteobacteria	Gammaproteobacteria	Unassigned	Unassigned	Unassigned
79	0	0	4	10	0	0	0	0	0	0	31	14	Bacteroidetes	[Saprospirae]	[Saprospirales]	Saprospiraceae	Lewinella
